# QTL Analysis Using SNP Markers Developed by Next-Generation Sequencing for Identification of Candidate Genes Controlling 4-Methylthio-3-Butenyl Glucosinolate Contents in Roots of Radish, *Raphanus sativus* L

**DOI:** 10.1371/journal.pone.0053541

**Published:** 2013-01-07

**Authors:** Zhongwei Zou, Masahiko Ishida, Feng Li, Tomohiro Kakizaki, Sho Suzuki, Hiroyasu Kitashiba, Takeshi Nishio

**Affiliations:** 1 Graduate School of Agricultural Science, Tohoku University, Sendai, Miyagi, Japan; 2 NARO Institute of Vegetable and Tea Science, Tsu, Mie, Japan; New Mexico State University, United States of America

## Abstract

SNP markers for QTL analysis of 4-MTB-GSL contents in radish roots were developed by determining nucleotide sequences of bulked PCR products using a next-generation sequencer. DNA fragments were amplified from two radish lines by multiplex PCR with six primer pairs, and those amplified by 2,880 primer pairs were mixed and sequenced. By assembling sequence data, 1,953 SNPs in 750 DNA fragments, 437 of which have been previously mapped in a linkage map, were identified. A linkage map of nine linkage groups was constructed with 188 markers, and five QTLs were detected in two F_2_ populations, three of them accounting for more than 50% of the total phenotypic variance being repeatedly detected. In the identified QTL regions, nine SNP markers were newly produced. By synteny analysis of the QTLs regions with *Arabidopsis thaliana* and *Brassica rapa* genome sequences, three candidate genes were selected, i.e., *RsMAM3* for production of aliphatic glucosinolates linked to GSL-QTL-4, *RsIPMDH1* for leucine biosynthesis showing strong co-expression with glucosinolate biosynthesis genes linked to GSL-QTL-2, and *RsBCAT4* for branched-chain amino acid aminotransferase linked to GSL-QTL-1. Nucleotide sequences and expression of these genes suggested their possible function in 4MTB-GSL biosynthesis in radish roots.

## Introduction

Glucosinolates, sulfur-rich plant secondary metabolites derived from amino acids found in Brassicaceae and related families, are important ingredients determining the flavor and taste of Brassicaceae vegetables and have an anti-carcinogenic effect in animals [1]. Glucosinolates have three moieties, i.e., beta-thioglucose, sulfonated oxime, and a variable aglycone side chain derived from an alpha-amino acid. More than 120 glucosinolates are classified into three groups, i.e., aliphatic, aromatic, and indolic glucosinolates, based on the distinct side chain [2], [3].

Recent studies using doubled haploid populations obtained from F_1_ and BC_4_F_1_ plants in oilseed *Brassica juncea* have revealed some major and minor quantitative trait loci (QTLs) for seed glucosinolate contents [4]. Fine mapping has revealed candidates for the genes controlling seed glucosinolate contents such as *GSL-Elong*, *Myb 28*, *GSL-ALK*, and *GSL-PRO* located in QTL regions in *B. juncea* [5]. An integrated linkage map containing amplified fragment length polymorphism (AFLP) markers and simple sequence repeat (SSR) markers has been constructed for QTL analysis of the contents of eight different glucosinolate compounds in leaves of *Brassica rapa* using a doubled haploid (DH) population, and 16 loci controlling aliphatic glucosinolate accumulation, three loci controlling total indolic glucosinolate contents, and three other loci regulating aromatic glucosinolate contents have been identified [6]. Many important genes participating in the three phases of the glucosinolate biosynthesis pathway, i.e., side chain elongation of precursor amino acids, formation of the core glucosinolate structure, and side-chain decoration, have been identified in *Aarbidopsis thaliana* and *B. rapa* 5,7,8. Branched-chain aminotransferase (BCAT) is involved in elongation of methionine, and methylthioalkylmalate synthase (MAM1 and MAM3) is involved in the methionine chain elongation pathway for isopropylmalate synthesis [9]–[13]. Core structure formation of glucosinolate is accomplished in five steps via oxidation of aldoxime by CYP79 and CYP83 families followed by C-S lyase, S-glucosyltransferase, and sulfortransferase [14]–[16]. Isopropylmalate dehydrogenase (IPMDH1) has been identified in the biosynthesis of both glucosinolate and leucine [10], [17], [18].

Radish (*Raphanus sativus* L., 2*n* = 2x = 18) is an important root vegetable in the family Brassicaceae and a common crop in East Asia. Genomic research on *R. sativus* has not progressed as much as that of *B. rapa*. Several genetic linkage maps of *R. sativus* have been constructed using restriction fragment length polymorphism (RFLP) [19]. AFLP [20]–[22]. SSR [23], and single nucleotide polymorphism (SNP) markers [23], and some of these have been applied to analysis of QTLs for shape and color of roots, flowering time [25], and disease and pest resistances [20], [22].

Radish roots contain 4-methylthio-3-butenyl glucosinolate (4MTB-GSL) with the common name of glucoraphasatin as a characteristic common glucosinolate [26] and 4MTB-GSL content is an important characteristic of radish cultivars [27]. Although the genes responsible for glucosinolate contents in *Brassica* seeds have been intensively studied, genetic analysis of glucosinolate contents in radish roots has not been reported. In genetic analysis of some traits influenced by many other factors, use of closely related parental lines is desirable. Since glucosinolate contents in radish roots are influenced by growing conditions and growth stages [28], parental lines having the same cultivation period are suitable for producing F_2_ populations for genetic analysis. However, for QTL analysis, it is required to construct a linkage map composed of DNA markers without a large gap, and closely related parental lines are not suitable for developing many DNA markers to cover a linkage map. In the present study, we analyzed QTLs for root glucosinolate contents in radish using two F_2_ populations obtained from a cross between a high 4MTB-GSL inbred line and a low 4MTB-GSL inbred line, both of which are Japanese daikon cultivars having thick roots. For this study, we developed SNP markers using sequence data of bulked PCR products obtained by next-generation sequencing to cover a linkage map. Three QTLs were repeatedly detected, and SNP markers in these QTL regions were developed. Furthermore, three candidate genes were mapped in the QTL regions, and nucleotide sequences and expression of these genes were analyzed.

## Materials and Methods

### Plant Materials

An inbred line, ‘TBS-2-5-3-2- (3)’ (‘TBS’ hereafter), derived from a radish F_1_ hybrid cultivar ‘Taibyousoubutori’, which is a major radish cultivar with a green shoulder in Japan, and an inbred line, ‘AZ26H-24-6-5- (3)’ (‘AZ26H’ hereafter), derived from a pungent local cultivar of Akita Prefecture in Japan, ‘Karamijidaikon’, were used as seed and pollen parents. Roots of ‘TBS’ contain lower amounts of glucosinolates than those of ‘AZ26H’. For QTL analysis, two populations of 220 and 180 F_2_ plants obtained by self-pollination of F_1_ between ‘TBS’ and ‘AZ26H’ were cultivated in a plastic greenhouse of the NARO Institute of Vegetable and Tea Science, Kusawa 360, Tsu, Mie, Japan, for four months during autumn-spring in 2009–2010 and 2010–2011, respectively.

### Analysis of Glucosinolate Contents

A simple, rapid method using a common solvent, methanol/water = 80:20 (V/V), at room temperature was applied to the extraction of crude glucosinolate from the powder of freeze-dried radish roots. Quantification of 4-methylthio-3-butenyl glucosinolate and other types of glucosinolates was done using HPLC [29], [30].

### SNP Identification

In our previous study [24], 2,880 primer pairs were designed for specific amplification of coding regions of genes containing 3′-untranslated regions. These primer pairs were divided into 480 groups by MultiPLX 2.0 (six primer pairs in each group) (**Table S1**) [31]. Genomic DNAs of ‘TBS’ and ‘AZ26H’ were prepared by a modified cetyltrimethyl ammonium bromide (CTAB) method [32]. Multiplex PCR was conducted in a 10-µl reaction mixture with 20 ng of plant genomic DNA, 10 pmol of each primer mixed as a group, 0.5 unit of *TaKaRa Ex Taq*® DNA polymerase (Takara Biomedicals, Japan), 1 × Ex Taq buffer, and 0.2 mM dNTP each. Thermal cycling conditions were as follows: 94°C, 2-min denaturation, 40 cycles of 94°C for 30 sec, 57°C for 30 sec, and 72°C for 1 min, and a final extension at 72°C for 1 min. All the PCR products of each line were collected and mixed in one tube and then digested with six enzymes separately (*Alu*I, *Mse*I, *Hae*III, *Mbo*I, *Msp*I, *Afa*I, New England Biolabs). The digestion products were again mixed and purified using ethanol precipitation. Sequences were determined using Illumina GAIIx. Using the software Bowtie (http://bowtie-bio.sourceforge.net/index.shtml), the obtained sequence reads of ‘TBS’ and ‘AZ26H’ were respectively mapped to reference sequences previously determined by the Sangar method to produce SAM files [24], which were then analyzed by the program SAMtools (http://samtools.sourceforge.net/) to identify SNPs.

### SNP Analysis of F_2_ Plants

Genomic DNAs from 189 and 174 plants of the two F_2_ populations, which were prepared by the modified CTAB method, were analyzed by a modified dot-blot-SNP genotyping method, the MPMP-dot-blot-SNP method, following Li et al. [24] First, 30 primer pairs of SNP markers were separated into six groups by MultiPLX 2.0 (five primer pairs per group). Next, the probe hybridization conditions were predicted as described by Shiokai et al. [33] based on Tm values estimated by the DINAMelt web server (http://mafold.rna.albany.edu/?q=DINAMelt/hybird2). Multiplex PCR was performed in a 10-µl reaction mixture with 3.5 ng of plant genomic DNA, 10 pmol of each primer mixture, 1x KAPATaq Extra Buffer (without Mg^2+^), 1.75 mM of MgCl_2_, 0.3 mM of each dNTP, and 0.25 U of DNA polymerase (KAPATaq Extra). Thermal cycling conditions were as follows: 94°C, 1 min denaturation, 40 cycles of 94°C for 30 sec, 56°C for 30 sec, and 72°C for 1 min, and a final extension at 72°C for 1 min. Amplified PCR products were denatured in a solution of 0.4 N NaOH and 10 mM EDTA and then dot-blotted onto a nylon membrane (Nytran, Pall, NY, USA) using a Multi-pin Blotter (Atto, Tokyo, Japan). DNA fragments on the nylon membrane were hybridized with unlabeled allele-specific oligonucleotides having bridge sequences [34]. Allele-specific signals were detected by hybridization of digoxigenin-labeled oliginucleotides having sequences complementary to the bridge sequences according to Shiokai et al. [33] One SCAR (sequence characterized amplified region) marker was analyzed using 3% agarose gel.

### QTL Analysis

A linkage map was constructed using the JoinMap 4.0 software (Kyazma B.V., Wageningen, the Netherlands) for each population [35]. Linked loci were grouped into nine linkage groups (LGs) at high logarithm of odds (LOD) values (≥6). The Kosambi mapping function was used to convert recombination values into map distances. QTL analysis was performed applying a composite interval-mapping analysis with Windows QTL Cartographer v2.5 [36]. The LOD thresholds for QTL significance were determined by a permutation test (1000 replications) with a genome-wide significance level *P*  = 0.05.

### Nucleotide Sequence Analysis of Candidate Genes in Glucosinolate Biosynthesis Pathway

By BLASTN analysis of SNP markers in QTL regions with sequences of *A. thaliana* and *B. rapa,* homologues in the QTL regions were applied to prediction of genes controlling glucosinolate content [7]. Primers were designed to amplify genes of *CYP83A1*, *CYP79F1*, *BAT5*, *BCAT4*, *MAM3*, *MAM1*, and *IPMDH1* from genomic DNA in ‘TBS’ and ‘AZ26H’ (**Table S2**). The amplification products were purified by UltraCleanTM15 DNA Purification Kit (MO BIO, USA) and were cloned into pGEM-T Easy Vector (Promega). Sequences of six clones for each product were determined with a DNA sequencer (CEQ2000, Beckman Coulter). The sequences were aligned using SEQUENCHER version 4.7 (Gene Codes Corporation, MI, USA). After comparing nucleotide sequences between the parental lines, probes were designed to map these genes on the linkage map using the F_2_ population. Exons and introns of these genes were inferred by BLAST analysis with EST sequences of *R. sativus* (http://blast.ncbi.nlm.nih.gov/).

### Expression Analysis of Candidate Genes

RNA was extracted from 30 mg roots of ‘TBS’ and ‘AZ26H’ four months after sowing using the SV Total RNA Isolation System (Promega Corp.). One microgram of total RNA from each sample was reverse-transcribed in a 22 µl reaction mixture using SuperScript^TM^III Reverse Transcriptase First-strand cDNA Synthesis (Invitrogen). RT-PCR was performed with specific primers designed from exon regions of *BCAT4*, *MAM3*, *CYP83A1*, *CYP79F1*, *MAM1*, and *IPMDH1* by 30 cycles of 94°C for 30 sec, 58°C for 30 sec, and 72°C for 30 sec. Real-time PCR was performed to quantify mRNAs of *MAM3*, *IPMDH1*, and *BCAT4* using a LightCycler (Roche Diagnostics) with SYBR Premix EX Taq (Takara Biomedicals). Specific primer sets were designed for real time PCR and *Actin* was used as an internal control (**Table S2**). Thermal cycling conditions were as follows: 30 cycles of 95°C for 5 sec, 60°C for 20 sec, and 72°C for 15 sec.

## Results and Discussion

### Phenotypic Variation

**Figure 1:**
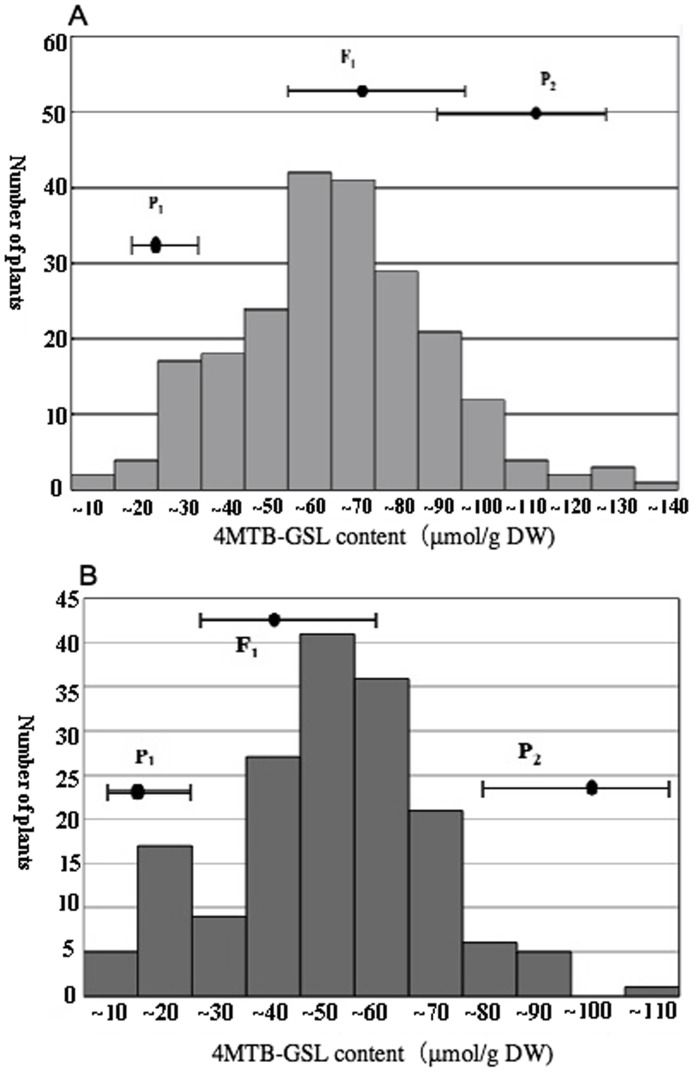
Distribution of 4MTB-GSL contents in the two F2 populations obtained by crossing ‘TBS’ and ‘AZ26H’. (A) F2 population grown in 2010, (B) F2 population grown in 2011.

The contents of 4MTB-GSL of P_1_ (‘TBS’) ranged from 24.4 to 40.8 µmol/g DW and those of P_2_ (‘AZ26H’) ranged from 96.3 to 137.7 µmol/g DW. Average contents of 4MTB-GSL of P_1_ and P_2_ were 30.5 and 120.0 µmol/g DW, respectively. The percentage of 4MTB-GSL in the total glucosinolates was more than 96%. The 4MTB-GSL contents in radish roots of the F_2_ plants harvested in 2010 were distributed from 5.5 to 130.6 µmol/g DW with an average of 60.7 µmol/g DW (**Figure 1A**). The frequency distribution of 4MTB-GSL contents in the F_2_ population showed a continuous, bell-shaped distribution, the contents from 50 µmol/g DW to 100 µmol/g DW accounting for 76.8%. The 4MTB-GSL contents of the F_2_ plants harvested in 2011 ranged from 6.6 to 103.6 µmol/g DW and the average content was 45.6 (µmol/g DW) (**Figure 1B**). Two inbred parental lines contained significantly different 4MTB-GSL content in radish roots that provided ideal material for QTL analysis.

### Production of SNP Markers by Nucleotide Sequencing of Bulked PCR Products

Nucleotide sequencing of multiplex PCR products amplified with 2,880 primer pairs using an Illumina sequencer determined sequences of 2,301 and 2,328 fragments of ‘TBS’ and ‘AZ26H’, respectively. The numbers of read bases of ‘TBS’ and ‘AZ26H’ were 175 Mb and 140 Mb, respectively. Comparison of sequence data of 1,777 fragments between ‘TBS’ and ‘AZ26H’ revealed 2,655 possible SNPs. Among them, SNPs with more than five repetitive reads were regarded as credible SNPs, and 1,953 SNPs in 750 DNA fragments (**Table 1** **, S1**), 437 of which had been previously mapped in a linkage map, were identified. Among them, 133 SNPs were selected as markers, because probes of dot-blot-SNP markers for these SNPs have already been produced (**Table S3**) [24]. Twenty-five markers were screened by analysis of the polymorphism between the parental lines from the EST-SNP markers on the high-density linkage map of our previous study (**Table S4**) [24]. To fill gaps in a linkage map, 30 newly identified SNPs in DNA fragments, which had been mapped previously, were used for developing probes of dot-blot-SNP markers. SNPs identified by new-generation sequencing enabled efficient screening of SNP markers for the parental lines used for producing a segregating population. Furthermore, new SNPs can be identified for developing oligonucleotide probes that had been positioned in gap regions of a linkage map.

**Table 1:** SNPs between the parental lines identified by next generation sequencing.

Parental lines	Primer sets used	Totalamplicons	Number of SNPs with readdepth more than five	Total length with read depth more than five	Frequency	Amplicons having SNPs with read depth more than five
TBS	2,880	2,301	1,952^a^	151,839	1/77.8	729
AZ26H	2,880	2,328	2,656 a	203,267	1/76.5	970
Between lines	–	–	1,953	–	–	750

a The sequence data of 'Aokubi' were used as references for SNP identification.

Numbers of SNPs with ‘Aokubi’ are shown.

Next-generation sequencing technologies have been applied to gene discovery, transcript quantification, marker production, and so on [37]. In *Brassica napus*, for example, about 40,000 SNPs have been discovered by Solexa-based transcript sequencing of two cultivars, and reads have been analyzed by mapping them onto a reference database of unique genes retrieved from publication archives [38]. The number of SNP markers identified by next-generation sequencing of bulked PCR production by 2880 primer pairs, i.e., 750, was more than sufficient for QTL analysis, and 1,152 primer pairs might be sufficient for producing SNP markers only for QTL analysis. The method used in the present study is simpler and more efficient for producing SNP markers than transcript sequencing. Furthermore, primer pairs used for multiplex PCR in sequence analysis can be used for producing SNP markers. The primer mixture for multiplex PCR would be useful for identification of SNPs between other parental lines for QTL analysis of radish and *Brassica*.

### Linkage Map Construction of Radish for QTL Analysis

**Figure 2:**
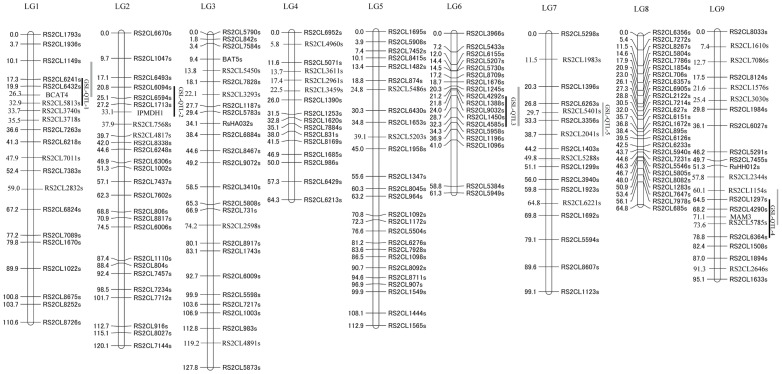
Linkage map of SNP markers showing polymorphism between ‘TBS’ and ‘AZ26H’. Black bars and gray bars indicate QTL regions of 4MTB-GSL contents detected in 2010 and 2011, respectively.

Using 188 dot-blot-SNP markers (**Table S3, S4**), 189 F_2_ plants harvested in 2010 were genotyped. Finally, the 188 SNP markers were mapped on linkage groups. A linkage map with nine linkage groups (LGs) spanned a total length of 856.0 cM with individual group sizes ranging from 61.3 cM (LG6) to 127.8 cM (LG3), and the average distance between neighboring markers was 4.37 cM (**Figure 2**). The marker positions in this map were in accordance with those in the radish map published by Li et al. [24].

For the analysis of the 174 F_2_ plants harvested in 2011, a linkage map was first constructed using 118 markers without use of markers closely linked with other markers. The final map consisted of nine LGs with 127 SNPs and one SCAR locus. The total length was 849.4 cM with an average distance of 6.58 cM between neighboring loci.

### QTL Analysis of Glucosinolate Contents in Radish Roots

Four significant QTLs for 4MTB-GSL contents in radish roots were detected on LG1, LG2, LG6, and LG9 by analysis of the F_2_ population harvested in 2010, and four QTLs were also detected on LG1, LG6, LG7, and LG9 by analysis of the F_2_ population harvested in 2011 (**Figure 2**). LOD threshold values for significant QTLs in 2010 and in 2011 were determined to be 4.90 and 3.40, respectively, by the permutation test (**Table 2**). Among these five QTLs, GSL-QTL-1 on LG1, GSL-QTL-3 on LG6, and GSL-QTL-4 on LG9 were detected in both populations. In the population of 2010, these three QTLs explained more than 50% of the total phenotypic variance, with the GSL-QTL-3 on LG6 having the highest contribution value, i.e., 29.50%. By contrast, GSL-QTL-3 explained the small phenotypic variance, i.e., 16.72%, in 2011. ‘AZ26H’ alleles of GSL-QTL-3 and GSL-QTL-4 played major roles in increasing 4MTB-GSL contents. GSL-QTL-1 located between makers RS2CL6241 and RS2CL7263 on LG1 had a reverse additive effect, which explained the total phenotypic variances of 13.39% in 2010 and 36.7% in 2011. GSL-QTL-2 and GSL-QTL-5 explained the total phenotypic variances of 14.05% and 11.09%, respectively, but were detected only in one year. The average content of 4MTB-GSL in the F_2_ population harvested in 2010 was higher than that harvested in 2011 (**Figure 1**), suggesting that environmental factors, e.g., temperature, moisture, and harvesting time, have considerable effects on 4MTB-GSL contents. Therefore, they may have influenced the detection of QTLs in different years.

**Table 2:** QTL analysis of 4MTB-GSL contents in radish roots.

QTL	Linkage group	Nearest marker	2010 F_2_ population			2011 F_2_ population		
			LOD	Additve^a^ effect	Variance explained (%)	LOD	Additveeffect	Variance explained (%)
GSL-QTL-1	LG1	RS2CL6432s	5.87	7.94	13.39	19.1	11.84	36.70
GSL-QTL-2	LG2	RS2CL6594s	5.62	−5.62	14.05	1.54^b^	−4.27^b^	3.20^b^
GSL-QTL-3	LG6	RS2CL4585s	5.19	−16.98	29.50	3.62	−10.53	16.72
GSL-QTL-4	LG9	RS2CL4290s	7.36	−8.21	13.28	4.09	−9.54	11.09
GSL-QTL-5	LG7	RS2CL3356s	1.83^b^	0.31^b^	3.31b	3.85	−9.19	11.30
Threshold value			4.90			3.40		

a, Additive effects of ‘TBS’ alleles are shown.

b, Not significant.

Seventeen QTLs have been identified as controling seed glucosinolates and four have been revealed to play major roles in glucosinolate accumulation of *B. juncea* by analysis using an F_1_DH population. Among them, only three major QTLs have been repeatedly detected in a BC_4_DH population [3], [4]. In *B. rapa*, QTL analyses have identified 16 loci controlling aliphatic glucosinolate accumulation in leaves, three loci controlling total indolic glucosinolate contents, and three loci regulating aromatic glucosinolate contents [6]. In the present study, five QTLs associated with 4MTB-GSL contents were identified in the two F_2_ populations in radish roots, and three of them, i.e., GSL-QTL-1, GSL-QTL-3 and GSL-QTL-4, were repeatedly detected. Although there is a possibility of the presence of more QTLs controlling total glucosinolates in radish as shown in *B. rapa* leaves, for only the 4MTB-GSL contents, which were found to constitut more than 96% of the total glucosinolate content, these five QTL are considered to play major roles.

To understand the additive effect of the two reproducible QTLs (GSL-QTL-3 and GSL-QTL-4), the F_2_ plants were categorized by marker genotypes at each QTL, and the 4MTB-GSL contents were compared. The genotypes of BB-BB (genotype of GSL-QTL-3 - genotype of GSL-QTL-4), BB-AB, and AB-BB, in which A is a ‘TBS’ allele and B is an ‘AZ26H’ allele, showed significantly higher contents of glucosinolates than those of BB-AA, AA-BB, and AA-AA in the two populations (**Table 3**). In a combination of GSL-QTL-1 (positive additive effect of ‘TBS’ alleles) and GSL-QTL-3 (negative additive value effect of ‘TBS’ alleles), BB-AA (genotype of GSL-QTL-1 - genotype of GSL-QTL-3), BB-BB, and BB-AB showed lower glucosinolate contents than those of AA-AB, AA-BB, and AA-AA (**Table S5).** This indicates that the allele at GSL-QTL-1 from ‘AZ26H’ acts to decrease the 4-MTB-GSL contents and that the alleles at GSL-QTL-3 and GSL-QTL-4 from ‘AZ26H’ act to increase the contents. However, the heterozygous genotypes of GSL-QTL-1, i.e., AB-BB or AB-AB, showed higher glucosinolate contents than the homologous genotypes, i.e., AA-BB and BB-BB or AA-AB and BB-AB. This may be due to a heterosis effect of GSL-QTL-1 on 4MTB-GSL biosynthesis.

**Table 3:** Comparison of 4MTB-GSL contents between different genotypes of SNP markers in GSL-QTL-3 and GSL-QTL-4.

Group number	Marker genotype		2010 F_2_ population		2011 F_2_ population	
	RS2CL4585s inGSL-QTL-3	RS2CL4290s inGSL-QTL-4	Number of plants	4MTB-GSL content (µmol/g DW)	Number of plants	4MTB-GSL content(µmol/g DW)
1	BB	BB	6	78.3±5.14 a	9	74.1±3.24 a
2	BB	AB	19	65.3±4.32 a	14	68.5±5.12 ab
3	AB	BB	12	59.6±3.01 a	23	59.2±3.54 ab
4	AB	AB	25	53.7±2.32 ab	46	52.5±2.84 ab
5	AA	AB	18	51.2±3.12 ab	17	42.4±3.26 bc
6	AB	AA	10	46.5±4.09 ab	14	47.5±2.53 b
7	BB	AA	5	44.3±5.07 b	13	44.9±4.72 bc
8	AA	BB	8	41.0±4.31 b	9	38.1±4.08 c
9	AA	AA	7	36.4±6.23 b	10	35.0±5.24 c

Note: Values followed by the same letter within each experiment were not significantly different at the 5% level by Tukey's multiple comparison test.

DNA markers in GSL-QTL-1, -3, and -4 are considered to be useful for marker-assisted selection of radish. As radish for a salad or other fresh dishes, radish cultivars having low content of 4-MTB-GSL are preferred, while cultivars having high content of 4-MTB-GSL are required as a spice or a functional food. Since dot-blot-SNP analysis is suitable for SNP genotyping of a large number of plants [34], the dot-blot-SNP markers used for detection of GSL-QTL-1, -3, and -4 and SNPs of candidate genes in the QTL regions (discussed later) can be used in radish breeding.

### Sequence Variation of Glucosinolate Biosynthesis Genes

Search for the SNP markers in the QTL regions were conducted by BLAST using the genome sequence of *A. thaliana* and *B. rapa*. New SNP markers were added to the QTL regions by selecting markers having SNPs between ‘TBS’ and ‘AZ26H’, which have been previously mapped in these regions. Three SNP markers were selected for GSL-QTL-1. Two markers were added to GSL-QTL-2, GSL-QTL-4, and GSL-QTL-5, respectively (**Table 4**). In the linkage map, SNP markers Rs2CL1297s and Rs2CL1508s of GSL-QTL-4 were shown to have high homology with At5g11670 and At5g24810, respectively [24]. In this region, *MAM3* (At5g23020, Bra013009/013011/029356/021947) was selected as a candidate gene by comparison with the *B. rapa* genome [40]. Moreover, *IPMDH1* (At5g14200, Bra023450) and *BCAT4* (At3g19170, Bra02248/Bra001716) were found in syntenic regions with GSL-QTL-2 (At5g05260-At5g18380) and GSL-QTL-1 (At3g21770-At3g22600). No candidate gene was detected in GSL-QTL-3, which accounted for the largest contribution to phenotypic variance, and GSL-QTL-5 by synteny between *R. sativus* and *B. rapa.* The glucosinolate biosynthesis pathway is a complex network. In previous studies, more than fifty genes have been found to be involved in glucosinolate biosynthesis, including transcription factors, core structure formation, secondary modification, and core-substrate pathways [7], [42]. There may be other genes controlling 4MTB-GSL contents or several genes co-regulating the 4MTB-GSL biosynthesis in this locus.

**Table 4:** Newly developed markers in QTL regions.

Linkagegroup	QTLregion	Markername	Forward primer (5'-3')	Reverse primer (5'-3')	TBS-SCR27	AZ26H-SCR52	Position(cM)
LG1	GSL-QTL-1	RS2CL5813	ATGGCAACCAGCTTACCAGTCT	AACCAAGTCCAAACTTGCCATC	CTAGTGCCACCGCCGCA	CTAGTGCCGCCGCAGCA	32.9
		RS2CL3740	TGGGACAAGCTCTGGACTATCA	AACGTGAGCTTCACCAACTCAT	TTTGAAGGTGCTGGAGA	TTTGAAGGCGCTGGAGA	33.7
		RS2CL3718	CAGTTTTGAGGCAAGTTTGTGC	TCAAGTTCTGCTCAGGGGAGAT	CTTTGGATCATTGCAGC	CTTTGGATTATTGCAGC	35.5
LG2	GSL-QTL-2	RS2CL7568	ATTGCATCTCCTTCCACTCCAT	GCTTAGGCATTGCGTTTCTAGC	GAAGCCGTAGCCCACGG	GAAGCCGTTGCCCACGG	37.9
		RS2CL4817	CTCGTCTGCGCTTATGGTTATG	ACTAACGTTTGCCCCTGTCAAT	CTTAAGTACCATTTTGC	CTTAAGTATCATTTTGC	39.7
LG9	GSL-QTL-4	RS2CL5785	AGATTGTGATGTGGGCTGAGAA	TCTCGTTTAGCAACTCCACTGC	AAGGGTATCGGAAGGTT	AAGGGTATAGGAAGGTT	73.6
		RS2CL2646	AACATAACATGGGACGTTCTGC	GAAACAGGGGAGAAACAAGAGG	TTTGCAATCACCTCGTA	TTTGCAATTACCTCGTA	91.3
LG7	GSL-QTL-5	RS2CL2041	GCCCAGTCCTGTTCTTGAGATT	TAATATGGGTGGCCTCTGCTCT	GTGATCGTATTCAAAAA	GTGATCGTTTTCAAAAA	38.7
		RS2CL5401	ACATCAGAACGTGGAACAATGC	TTGAAACCGAGAAGAGCTGGAT	TTTCCCTGATGATGTGG	TTTCCCTGGTGATGTGG	29.7

Note: The oligonucleotide probes were designed as bridge probes (Shiokai et al. 2010). Sequences excluding the bridge sequence are shown. A sequence, TATATTTACATTCGCAATTAAGAGGCTTCGT designated as SCR-27, and a sequence, TATATTCCCTCCGTCAGCGGATC designated as SCR-52, were added to allele-specific sequences of TBS and AZ26H, respectively.

To confirm effects of these candidate genes on 4MTB-GSL content of radish roots, three other important genes, i.e., *MAM1* for side-chain formation, and *CYP83A1* and *CYP79F1* for core structure formation, were also analyzed (**Table S2**) [10], [17], [18], [39]. Sequences of these genes were determined and exons were inferred by BLASTN of EST sequences of *R. sativus* (http://blast.ncbi.nlm.nih.gov/) listed in **Table 5**. Comparison of *R. sativus* sequences of these genes, here named *RsMAM3*, *RsIPMDH1, RsBCAT4, RsMAM1, RsCYP83A1,* and *RsCYP79F1,* with *A. thaliana* and *B. rapa* sequences revealed that sequences of *RsMAM3, RsMAM1, RsCYP83A1,* and *RsCYP79F1* showed more than 85% nucleotide identities in both exons and introns with homologous sequences in *A. thaliana* and *B. rapa.* However, genomic sequences including exons and introns of *RsIPMDH1 and RsBCAT4* showed lower identities with homologous sequences in *A. thaliana* and *B. rapa.* Even exon sequences of *RsIPMDH1 and RsBCAT4* showed low nucleotide identities between *R. sativus* and *A. thaliana*, i.e., 61% and 74%, respectively, and between *R. sativus* and *B. rapa*, i.e., 68% and 73%, respectively. In all six of these genes, the allocation of exons and introns was almost the same between *R. sativus* and *A. thaliana*.

**Table 5:** Sequence analysis of glucosinolate biosynthesis genes in *R. sativus.*

Gene name	Position	Length (bp)		Number ofSNPs in exons	Number of indelsin exons	Number of SNPsin introns	Number of indels in introns
		TBS	AZ26H				
*RsMAM3*	GSL-QTL-4	1620	1621	1	0	0	0
*RsIPMDH1*	GSL-QTL-2	1781	2285	1	0	5	3
*RsBCAT4*	GSL-QTL-1	1952	1963	0	0	8	7
*RsCYP79F1*	Not mapped	968	968	0	0	0	0
*RsCYP83A1*	Not mapped	1624	1623	0	0	0	1
*RsMAM1*	Not mapped	2512	2513	0	1	5	2

**Figure 3:**
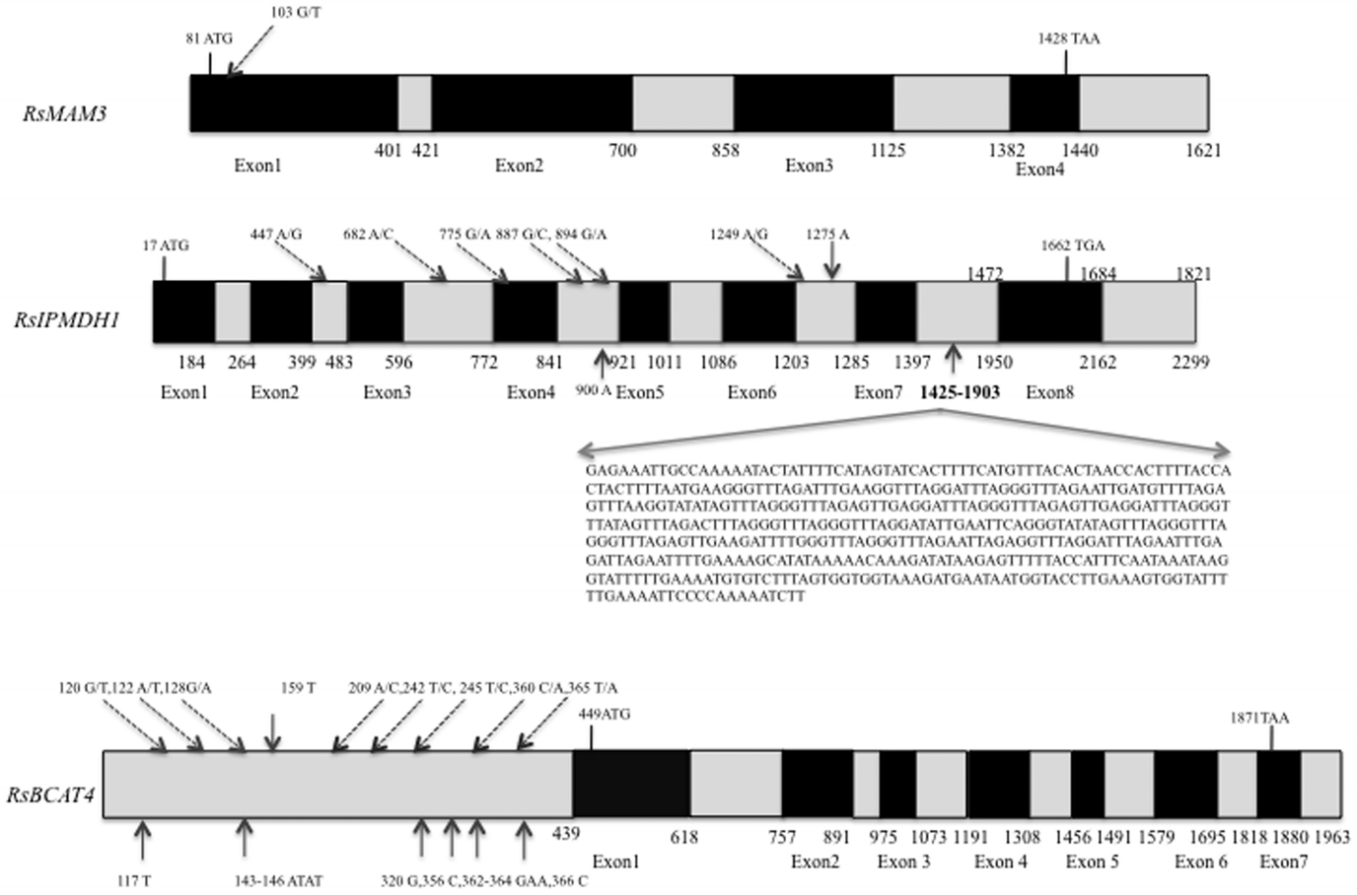
Nucletide polymorphisms of *RsMAM3*, *RsIPMDH1*, and RsBCAT4 between ‘TBS’ and ‘AZ26H’. The black and gray boxes indicate exons and introns, respectively. The black arrows show indels. The positions of dashed arrows indicate SNP sites and nucleotide variations. The numbers under the boxes indicate the start and stop sites of exons.

Comparison of the sequences between ‘TBS’ and ‘AZ26H’ showed *RsMAM3* to contain one SNP in an exon region, *RsIPMDH1* to have 478 bp insertion in ‘AZ26H’ besides six SNPs and two single nucleotide indels in exons and introns, and *RsBCAT4* to contain nine SNPs and six indels in the intron regions (**Table 5** **,** **Figure 3**). *RsCYP83A1* had one insertion in an intron region between parental lines, and *RsMAM1* had five SNPs and three indels throughout intron and exon regions. There was no variation in *RsCYP79F1* between the parental lines (**Table 5** **, Figure S1**). SNPs from *RsMAM3* and *RsBCAT4* were used for designing probes for genotyping of the F_2_ population. *RsMAM3* and *RsBCAT4* were found to be linked to the GSL-QTL-4 and GSL-QTL-1 regions, respectively. Based on 478 bp insertion, a SCAR marker was designed for genotype analysis of *RsIPMDH1* and was revealed to be linked to GSL-QTL-2 (**Figure 2**). *RsCYP83A1* and *RsMAM1* were not linked to the map, possibly due to distorted segregation ratios, i.e., 153:35 and 47:68:73, respectively. High similarity of *RsCYP79F1* sequences between ‘TBS’ and ‘AZ26H’ did not allow the design of a probe for genotyping. *RsMAM3, RsIPMDH1* and *RsBCAT4* were considered to be candidate genes which regulate the 4MTB-GSL content in radish roots.

### Expression Analysis of Candidate Genes for Enzymes of the Glucosinolate Biosynthesis Pathway

**Figure 4:**
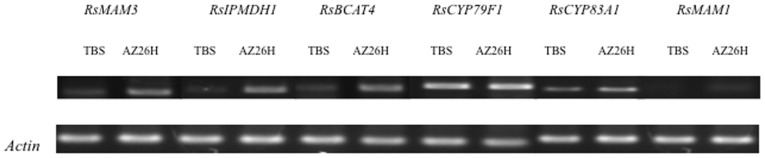
RT-PCR analyis of candidate gene transcripts in ‘TBS’ and ‘AZ26H’. RNAs were extracted from radish roots. *Actin* was used as a control to demonstrate equal RNA loading.

**Figure 5:**
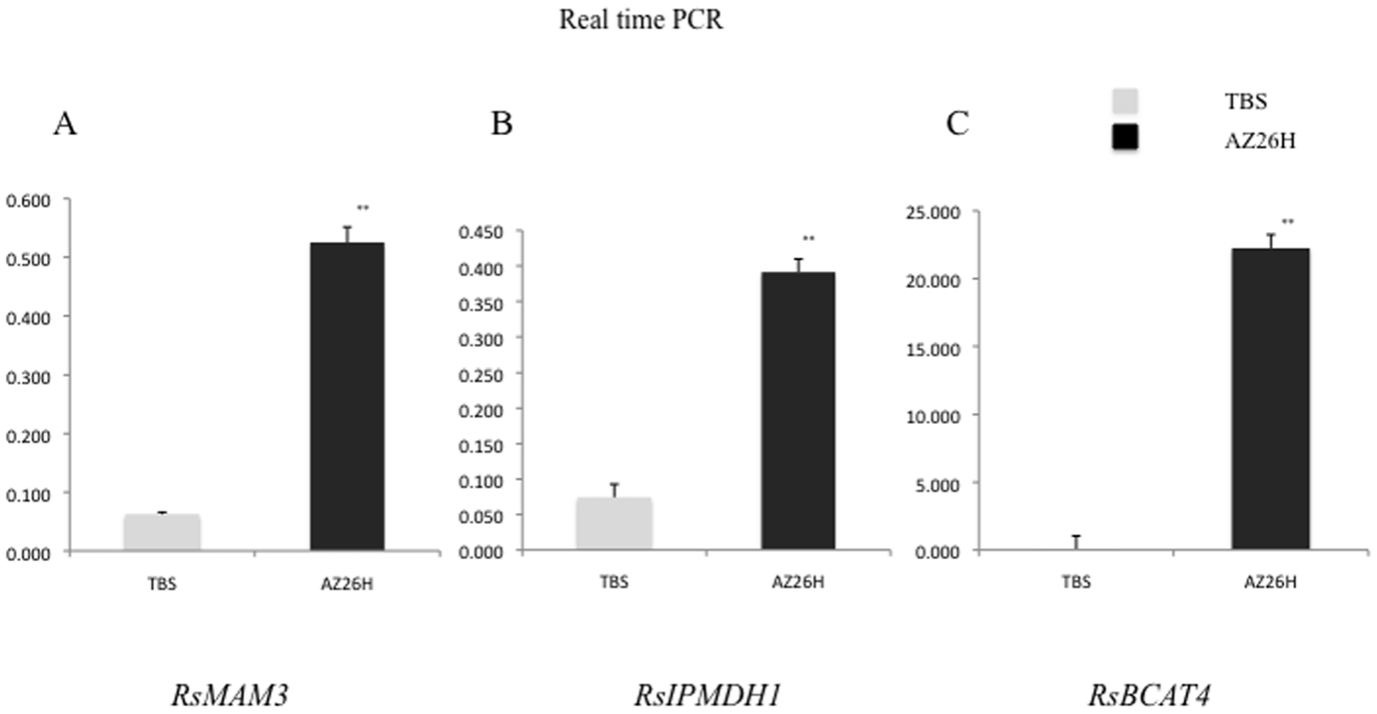
Gene expression analyses of parental lines by real-time PCR. (A) *RsMAM3,* (B) *RsIPMDH1,* (C) *RsBCAT4*. Gray and black bars indicate parental lines ‘TBS’ and ‘AZ26H’. Significant differences (P<0.01, LSD-t test) between the parental lines are indicated by asterisks.

We performed RT-PCR of six related genes using specific primers (**Table S2**) and compared gene expression levels between the parental lines in roots. Expression levels of *RsMAM3, RsIPMDH1* and *RsBCAT4* were higher in ‘AZ26H’ than those of ‘TBS’. However, *CYP83A1* and *MAM1* showed no expression difference although sequence variations were observed between the parental lines. The expression level of *CYP79F1* was also similar between the parental lines (**Figure 4**). Real-time PCR was employed to delineate detailed expression profiles of *RsMAM3, RsIPMDH1* and *RsBCAT4*. All these genes showed significantly higher expression levels in ‘AZ26H’ than those in ‘TBS’ (**Figure 5**).

*MAM3* catalyzes the condensation reaction of all side-chain elongation cycles. *MAM3* contributes to the production of all aliphatic glucosinolates but primarily to that of glucosinolates derived from one, five, and six elongation cycles [11], [12], [41]. *RsMAM3* was strongly expressed in the higher 4MTB-GSL content line (‘AZ26H’). 4MTB-GSL is one of the aliphatic glucosinolates (**Figure 4** **,** **5A**) [27]. One nonsynonymous SNP (G-T) in the first exon changes the amino acid from leucine to phenylalanine in the coding region (**Figure 4**). QTL results of 4MTB-GSL contents in radish roots suggested that *RsMAM3* plays a role in the difference of aliphatic glucosinolate contents between ‘AZ26H’ and ‘TBS’.

*IPMDH1* has been identified based on strong co-expression with glucosinolate biosynthesis and leucine biosynthesis, and a knockdown mutant of *IPMDH1* has been shown to cause a decrease in glucosinolate accumulation [7]. Mutation of *IPMDH1* leads to a significant reduction in the level of free leucine and of glucosinlates with side chains of four or more carbons in *A. thaliana* [18]. IPMDH2 and IPMDH3 are predicted enzymes that share a similar function with IPMDH1 [9]. The expression of *RsIPMDH1* was significantly higher in ‘AZ26H’ than that in ‘TBS’, implying that *RsIPMDH1* may play a co-regulation in the 4MTB-GSL biosynthesis pathway with other genes (**Figure 4** **,** **5B**).

*BCAT4* deaminates methionine to produce 2-Oxo-4-methylthio-thiobutanoic acid, which is processed to 2-Oxo-6-methylthio-thiohexanoic acid, and transaminates 2-Oxo-6-methylthio-thiohexanoic acid to produce dihomomethionine, which is an intermediate of the pathway to 4-MTB-GSL. A *BCAT4* knockdown mutant, *bcat4,* has been revealed to cause an approximately 50% reduction in aliphatic glucosinolates of leaves and to increase levels of free methionine, S-methyl-methionine, and the transport form of methionine, indicating that *BCAT4* regulates the first deamination of side chain elongation of the glucosinolate biosynthesis pathway [41]. Higher expression of *RsBCAT4* in ‘AZ26H’ than that in ‘TBS’ may suggest that *RsBCAT4* acts to increase 4MTB-GSL content of the ‘AZ26H’ allele (**Figure 4** **,** **5C**). However, the additive effect of the ‘TBS’ allele in GSL-QTL-1 was positive, suggesting that the ‘TBS’ allele increases 4-MTB-GSL content. Since the nucleotide sequence of *RsBCAT4* is highly different from that of *A. thaliana BCAT4*, the function of *RsBCAT4* might be different from that of *A. thaliana BCAT4*. Another possibility of this discrepancy may be the presence of another gene enhancing glucosinolate biosynthesis in ‘TBS’ roots. Delimitation of a GSL-QTL-1 region is required to identify the gene responsible for 4-MTB-GSL contents.

*CYP79F1* converts all chain-elongated Met-derivatives, and *CYP83A1* converts aliphatic aldoximes [43]–[46]. Amino acid chain elongation occurs, in which additional methylene groups are inserted into the side chain (up to six in *A. thaliana*). The condensation step of the cycle is considered to be critical for side chain variation and is catalyzed by a methythioalkymalate synthase enzyme. *MAM1* catalyzes the condensation of side-chain variation in the first three elongation cycles and is involved in the production of glucosinolates derived from two elongation cycles [11], [13], [47]. Although there were sequence variations in *RsCYP83A1* and *RsMAM1*, the expression levels were similar between ‘TBS’ and ‘AZ26H’. Sequences of *RsCYP79F1* were the same between the parental lines and there was no significant difference in the expression level between them (**Table 5** **,** **Figure 4**). These genes may not contribute to the difference of 4MTB-GSL contents between ‘TBS’ and ‘AZ26H’.

### Conclusions

Using the sequence data obtained by next-generation sequencing of bulked PCR products, a large number of SNP markers were developed for QTL map construction. Five QTLs were detected and three of them were repeeatedly detected. By analyzing synteny of the QTL regions with *A. thaliana* and *B. rapa*, three candidate genes were identified. Sequence and expression analyses indicate that they may be responsible for the difference of 4MTB-GSL contents between ‘TBS’ and ‘AZ26H’.

## Supporting Information

Figure S1 **Nucletide polymorphsims of** ***RsCYP79F1, RsCYP83A1,*** **and** ***RsMAM1*** **between ‘TBS’ and ‘AZ26H’.** The black and gray boxes indicate exons and introns, respectively. The black arrows show indels. The positions of dashed arrows indicate SNP sites and nucleotide variations. The numbers under the boxes indicate the start and stop sites of exons.
(TIF)

Table S1 **Primer pair groups for multiplex PCR and amplicons with SNPs detected between ‘TBS’ and ‘AZ26H’ by next generation sequencing.**
(PDF)

Table S2 **Sequences of primer pairs used for genomic DNA amplification, RT-PCR, and real-time PCR of glucosinolate biosynthesis genes.**
(PDF)

Table S3 **Sequences of primer pairs and oligonucleotide probes of SNP markers and the conditions of hybridization and washing.**
(PDF)

Table S4 **Dot-blot-SNP markers previously mapped with newly identified SNPs.**
(PDF)

Table S5 **Comparison of 4MTB-GSL contents between different genotypes of SNP markers in GSL-QTL-1 and GSL-QTL-3.**
(PDF)
