# Supplementary figures and images for: QTL Analysis Using SNP Markers Developed by Next-Generation Sequencing for Identification of Candidate Genes Controlling 4-Methylthio-3-Butenyl Glucosinolate Contents in Roots of Radish, Raphanus sativus L
Source: PLoS One. 2013 Jan 7;8(1):e53541. doi: 10.1371/journal.pone.0053541 (PMC3538544; doi:10.1371/journal.pone.0053541)

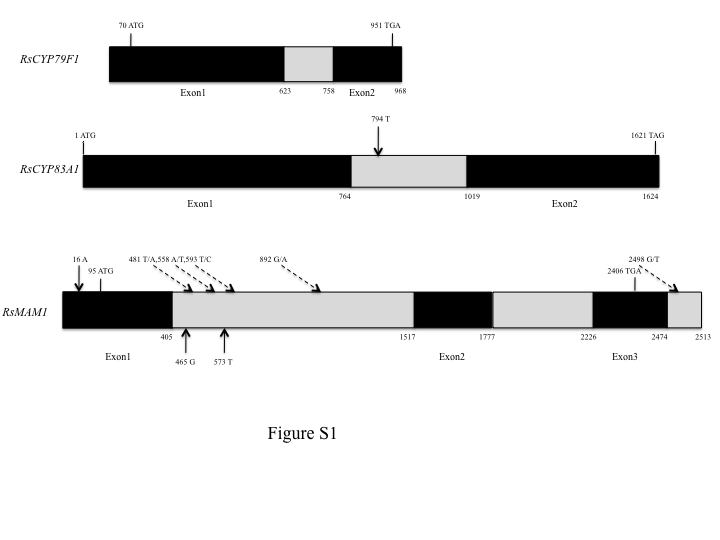

Supplement: Figure S1 — Nucletide polymorphsims of RsCYP79F1, RsCYP83A1, and RsMAM1 between ‘TBS’ and ‘AZ26H’. The black and gray boxes indicate exons and introns, respectively. The black arrows show indels. The positions of dashed arrows indicate SNP sites and nucleotide variations. The numbers under the boxes indicate the start and stop sites of exons. (TIF) [file pone.0053541.s001.tif]
