# Supplementary material for: QTL Analysis Using SNP Markers Developed by Next-Generation Sequencing for Identification of Candidate Genes Controlling 4-Methylthio-3-Butenyl Glucosinolate Contents in Roots of Radish, Raphanus sativus L
Source: PLoS One. 2013 Jan 7;8(1):e53541. doi: 10.1371/journal.pone.0053541 (PMC3538544; doi:10.1371/journal.pone.0053541)
Supplement: Table S1 — Primer pair groups for multiplex PCR and amplicons with SNPs detected between ‘TBS’ and ‘AZ26H’ by next generation sequencing. (PDF) [file pone.0053541.s002.pdf]

Table S1. Primer pair groups for multiplex PCR and amplicons with SNPs detected between 'TBS' and 'AZ26H' by next generation sequencing

Group	Primer name	Forward primer (5'-3')	Reverse primer (5'-3')	Amplified		SNPs	SNP marker name
				TBS	AZ26		
1	CL1039	TCTCCGCTGGGTTATAGGGTTA	CATCGGGTTCCAGAGATTCTTC	+	+	-	-
	CL1466	AGGTCGGTTTCTGAGGAAGATG	ACCCATCAGAGATTGCAAGACA	+	+	+	RS2CL1046s
	CL2312	GCTGGGGTAGGATCATCAAGAA	GGTAGATCCCAACTCCGTTTTG	+	+	-	-
	CL2360	CATCAGCAGCTTGATTCTCCAG	CAATGGAAGTGGAAGGGAGAGT	+	+	-	-
	CL5208	GACGCAAATGTAAGACGGGTTT	TACTGCTATCAAACACCGTTGG	+	+	+	RS2CL5208s
	CL6595	ACTGGTGGAAGGTTCGTCGTAT	GTTCGGAATTAACACGGTCGAT	-	-	-	-
2	CL1018	CGTCCACTGACTTTGACGATGT	ATATAACACGGGCCTCATTGCT	+	+	-	-
	CL1400	CTAGAACGGCTGGCTGATGATA	ACCATGAAAGGGTTCGAGTGTT	+	+	-	-
	CL2376	GATACCTTGCCTTCCTTGGAGA	CTACTCGTTTCTTTCGCAATGG	+	-	-	-
	CL3487	AACCGGTAGCGAAGTTCATCAT	ACTGATGAGAAGCCGAGTCAGA	+	+	-	-
	CL5083	ACGGAGTTTGAGGAACAGAAGG	TCCTTCCGAGAATGCCTAACTC	-	-	-	-
	CL5899	TCTACGACATTGGACCTCAGGA	TACAGAGGAGGGAACCATGTGA	+	+	-	-
3	CL1037	CAGCATGGAAAATATGGGGAAC	AAAAGCATCTACTCGGCTCCA	-	-	-	-
	CL1114	AGCCGTCATGGGTTTTCTACAG	TGGGACACTGAAACGAAGAAGA	+	-	-	-
	CL2526	CGACACCATTTGCAGATAAAGC	CAAACAACCAGAGAGCGAGAGA	+	+	+	RS2CL2526s
	CL3231	GAAGAAGAAAGGACCCATCGTG	TCCATTGATCGTAGTCCACTCA	+	+	+	RS2CL3231s
	CL6382	AGGTTTCTTCCCCTTCCGTTTA	CGAGATGGAAGAGTCGGTTTCT	+	+	+	RS2CL6382s
	CL6865	ACTCCATCGTTAAACCCCCAAT	CGTTGTCGAATGTGAGCTCTTT	-	-	-	-
4	CL3164	AATGAGGCGAAGAGAGCAAGAC	TTGCTGTGCACATACACAAACC	+	+	+	RS2CL3164s
	CL3221	AGATGGCAAGTCTCCTTCCAAA	GTGAACGTCAAGGAAGTTGTGG	+	+	+	RS2CL3221s
	CL3557	GAAGGCGAGAAGGGAAGCTTAT	AACCTCCAGGGATGATAGCAAG	+	+	-	-
	CL3828	CATCAAGCCTAACGACACCATC	AAACCGAGTAGCGAACCAAAAG	-	-	-	-
	CL4411	GACCAGGTTCTTCCACAAGGAT	CAGACCGTAGACAACGATGGAA	+	+	+	RS2CL4411s
	CL6770	GGAGAAGAGATGCAACACAACC	ACAAACCACCATATGCTCAACC	+	+	+	RS2CL6770s
5	CL1078	AGACCAATGCTTTCAACCGTCT	CCCTTTCCAACAAGCTAACGAC	+	+	-	-
	CL2426	TTGTCCAGAGCATCTTTTGCAG	TATCCATTACATTCGCGTGGTC	+	+	-	-
	CL2671	AATGCAAACACTCTGCGTCATC	TTGTCCTGAAACACGTCGAACT	+	+	-	-
	CL3025	CCCAATTGCATCGTGAAGAAG	CCATCACACCACCCCAATTA	-	-	-	-
	CL3917	GGGCCTAACGTTCAGTGGAATA	AAGCCACCAACACATGTACGTT	+	+	+	RS2CL3917s
	CL6291	GAGAAACAAGGCATGTCACCAG	AATGGGCCAGCAACAATAACTC	-	-	-	-
6	CL1200	CCCTTCCTCAGAGTTGGTTTTG	GATGATGTCTTCGCCGATGTTA	-	+	-	-
	CL2414	TGCTTCAGGGAGATGCTTGATA	CATCCATAGCGGATCAACGA	+	+	-	-
	CL3252	ATAAACCCTAAATCCGGGAGGA	CTTCCATGATCCCTGGAAAGAC	+	+	-	-
	CL4014	GCTCGTGAGTTGCTGAAACTTG	TGGTAGAACCACCAACAAGGAA	+	+	-	-
	CL5305	GAAGAGGATGAGGCTTTTTGGA	TCAGGAACCCTTGACAAAAGAC	-	-	-	-
	CL6494	TGCTTCATGCAAGCCTTAGAAC	TGTTGAGAGCAGCAGCATTACA	+	+	+	RS2CL6494s
7	CL1218	CGGTCATCAATACGCTCATCAT	ATGTGCTCGTTGACGATTCAGT	-	-	-	-
	CL2758	AGAACGTTGAGGTACCAACCAA	TCTTTACTCGTCAGCCTTCTCG	+	+	-	-
	CL3107	TGGACGGATTGACTATGGAGAA	AAACCCAAAAGAGGGTCAAAGC	+	+	+	RS2CL3107s
	CL3335	ACACAGACAAAGCAAAGGCAAG	CATTAGAGGCAACGGGAAGAAC	-	+	-	-
	CL4802	AAAGAAGGGCTGCAAGAAGATG	GCTTGAGCAGCAATCAAATCAG	+	+	+	RS2CL4802s
	CL5855	GTTCTTCTCATCAGCGCTCAAA	TCTGTTCTGTTCCACCGACTCT	-	-	-	-
8	CL1495	CATGGACGATCCATACTCATCA	TTGCCATTACAGGCTTCACATC	+	+	-	-
	CL1591	TTCCTTCACCCCTCCACAAT	CGGTGCAGTAGACAAGGATGAA	+	+	-	-
	CL1004	CGATTCAACATGGGAGTCTCTT	GAGATCCACATCACATGCTTCA	-	-	-	-
	CL1005	AACCAATGGCTTCCACACTTCT	AACCCTGAAAAGCTCAGTCACC	-	-	-	-
	CL1009	TTCGCTAGTTGGAACTTGCTTG	GGATGACATCGGATGATGCTAA	-	-	-	-
	CL1011	GAAGCCCTAAAAGCCGATCTCT	CTTGAGAACAACCGCAAATACG	+	+	+	RS2CL1011s
9	CL2635	AAAGGATGAGGACCATGCAACT	CTTTACCCACACGTGCATCATT	+	+	-	-
	CL2961	CTGGCCTATCCTGCTTTGCTAT	AGCTGCATAGATCTGTCCGTCA	+	+	+	RS2CL2961s
	CL3574	TCACCACACTCAATGTCTGTCA	CCAAGAAATCCAACCGCATA	-	+	-	-
	CL4553	TAGGGATGACTATGACCGAGCA	CTTTTCCTGGAGGGATGACAAC	-	-	-	-
	CL999	ATCGTGGGCGAACAGAAAGTAT	CATGCATACGTCGGTCTTTAGC	-	-	-	-
	CL1008	CAAGAGGAGGAAACCCATGTTC	TACGGCCTTGTCGACCTTTAAT	-	-	-	-
10	CL1023	CAACACCTTGATTTGCCAAGAC	GTCACCACCCCTTTTGTTTGTA	-	-	-	-
	CL1026	GCTTCTCGTTCCAGTTCTCACA	TCCAAGTGGGTTGATGATCTTG	-	-	-	-
	CL1087	TCCGAAATTCTAGCCTCCATGT	TCGCTGTGAAGAAAGTGACACA	+	+	-	-
	CL1134	CGTTTGATCTCAGCAGTGGAAC	AAAAGCAGGAAAGAGCTCATGG	+	+	-	-
	CL1176	TATGGACACCATCCCAACAAAC	AACCATTGAAGAGGCCAGAGAG	-	-	-	-
	CL1177	AGTGGATTTCTTGCGGTGGTAT	CCGTAAAAATCGCTTAGCATCC	+	+	-	-
11	CL1079	AAGCGAGGAAGTCGACAAAGTC	GCTCCAGCAACAATCTCAAGAA	-	-	-	-
	CL1112	TTGCTCTTCGTCTACGATTCCA	GGCAAACTGATTGAACTTGTGC	+	+	-	RS2CL1112s
	CL1113	GGAGAGCATGAGACAAGGAATG	CCCTTGACTTTATGTGCTCCTG	-	-	-	-
	CL1135	TACAAGTACCGGCCATAGGTGA	GCATGCTGAAAGATTCTCTGTG	-	-	-	-
	CL1150	GCAGAATCGAAAAAGGAACTGG	ACAAAAGGCAGAGAGACACACG	-	-	-	-
	CL1181	TGACGTAGCAAAGGTGAGAAGC	ACAAAGGATGCAAACACACAGC	+	-	-	-
12	CL1019	TGTTCTTCTCGCCATAAACCAC	CACTCACATTCGATCGGGAATA	-	+	-	-

	CL1032	AGCTCTCTCTGATGGCTGCTTT	CCTCACGACGTGTGAAGAAAAC	-	-	-	-
	CL1065	GTGACCCCTCAATGGTGAAGTT	CACAACCTTACATCCGATCCAT	-	-		
	CL1072	AGACGGGATACTTCAGGCAGTT	TCGCAGTCAACATCTCTGAACA	+	+	-	-
	CL1080	CCGAGCTCTTTACTCCCTTTGA	TGAGTCTGATGACGAGGAGCTT	-	-	-	-
	CL1111	TTGCACACAGGTGATCAAACAG	GTCATGGTAACCAACCAGCATC	-	-	-	-
13	CL1014	TTATCTCCATCGCTGTCGTCAT	CAGCCATACCATCCACAACAAC	-	-	-	-
	CL1033	TTGTCATCTCGAAGCCTGATGT	TACAAAGAGGACCAACGGGAAG	-	-	-	-
	CL1042	GCTGTGTATCGGTGCTTCAATC	TCCCTTTCGATATCCACCAACT	-	-	-	-
	CL1062	CTGGACAATGTAGGGATGTGGA	CGCCAACAAGGCATATGATAGA	-	-	-	-
	CL1152	ATGTGCTACTCCCACTTCAACG	AGCTTCTCTTCACTTGGCACTG	-	-	-	-
	CL1173	CGCTGGATTTATCTCCTCTGCT	ATCAGAATGGCAGACGAACTTG	-	-	-	-
14	CL1012	CACGGATTTGTGTACCGTGA	ACAGAAATCCAAAGGGGAACG	-	-	-	-
	CL1038	CTCGATCTTGCAAAGAGGGATT	CCTCCAAGATGACAATGAGCAC	-	-	-	-
	CL1125	CTTTCGTGCAGAGACAGGAAGA	CAAGAGTTCCCATCACATGGAG	+	+	-	-
	CL1148	CCCACTTGTTGTTGGTTCAAGA	CGAAGTTACCGGACCCAATATC	-	-	-	-
	CL1153	TGAGAAGGAGAGGCAAGCAGAT	CTGGCTCGCCTGTGTTATACAT	+	+	-	-
	CL1160	TCACATGTGCTACTCCCACTTC	AGCTTTCTTTCACTTGGCACTG	-	-	-	-
15	CL1052	AGTGCTCAAGAACGACGACAAG	CATCACACAACAGATGGAGGAA	-	-	-	-
	CL1081	CCTTGCACCAACTCAGACAGAA	GAACACTTCTCCCCTCCGTAAA	-	-	-	-
	CL1094	CTCCCGATCCAAACTCTTCTTC	GCCGAGAAGCTTGACGATATTT	-	-	-	-
	CL1108	ACTAATTTTGACGCGGTGGTG	GACAAGAAAGAGGCTCCCACAT	-	-	-	-
	CL1171	AACAGTGACCAGCTTCCCATCT	AGGAACCCAGTTGGCATACACT	-	-	-	-
	CL1179	GTAGTGAATGATGGCAGCGTTT	CTTCTTGCTGCTATGCGCTTTA	+	+	+	RS2CL1179s
16	CL1223	AACCCTAGCTTGGTTCATGCTC	CTCACTTTCCGGGAACAAAGTT	-	-	-	-
	CL1264	CACTCTCTCTCGATCCGATTCA	CAATCTTCGGAATCTGCAAGTG	-	-	-	-
	CL1281	CCAATGATGTCAACGAGCTTTC	GATTTCAGAGGCAGTGTCAACG	+	+	-	-
	CL1316	AACCCCTTCTTCGTCCTCTTCT	TTGCCCTTTTCAGAAACCTCTC	-	-	-	-
	CL1350	GTAGTGGATTTGGCTCACCTTG	CGAGATCTCTCATGAGGCAATG	-	-	-	-
	CL1351	CTCACTTTTGGCGCTTTCTCTT	GCGCAAAGTGTGTCTCTTTCAA	-	-	-	-
17	CL1261	TTGTCTCCTGTGCCTTTTGTGT	GGTACAAGGTTCGCTTTCTTGG	+	+	+	RS2CL1261s
	CL1280	ACCGGTAGTCAGTGCAACATCA	GATTGTGATGACGTTTGGGTTG	-	-	-	-
	CL1288	TGCATCGAGATGGTCTATTGCT	GCAGTTCACTCGAAAGGAGGTT	-	-	-	-
	CL1317	AAAGGATATTGCATCGGGTGAG	GTTGCTTCGTCTTTGCTCTCCT	-	-	-	-
	CL1322	CACAGGTTTGGTCGCAGAACTA	GGAGAAGAGGTTGTTGGAGGAA	+	-	-	-
	CL1324	CCTGCCTTATGCATCTCAGCTA	TTCATCTTTGCAGCTTCTCTCG	-	-	-	-
18	CL1183	TAAAGGTGTGATCCCAATGCAC	AAACGGTATGACCAACTCAGGA	+	+	+	RS2CL1183s
	CL1194	AAATAGAGCTGCGGTCTTCAGG	TAACATGCCGGCTTCACATTAG	+	+	-	-
	CL1201	CAGCTAACAAGGGGCAGAAACT	GGAGGCTGCTGATCAAGAAGAT	+	+	-	-
	CL1204	TCCCAAATCTCCTTACGAGTGG	GCAAAGCACACAACAGAGGAAC	+	+	-	-
	CL1268	AGCTACAAGCTCAAGCTGCTCA	GCCTCTAAAGTGCGAAACACAA	-	-	-	-
	CL1332	TTGGATGGCGTCAAATATGG	AATCGGATGCTCAGCTTCTACG	-	-	-	-
19	CL1190	AGTGGCAGATATATGCGCTGTT	CAGAGCTGAGGAAAAGGAGGAA	-	-	-	-
	CL1222	TGCGAAGGCTACGAACTTAGAA	GCCTCAATTGTTTGTCTCTTGC	+	+	-	-
	CL1230	TAACCAGAACACAGCCATCTCG	ATGTCGCTGGTTTACGTTCAGA	-	-	-	-
	CL1242	CGCAAACAGAGAGCGTTACAA	TGATGGTTCTCGCCATCATAGT	+	+	-	-
	CL1321	ATACAACCATGACCTGCCTTGA	TTTGTCTGGATCACCACTGAGC	-	-	-	-
	CL1334	GTCAACCCCAAAGCCATAATGT	AAGGATCGGAACTCGCTGATAG	-	-	-	-
20	CL1225	GTTGGAATCAGCGGTAAGATCC	TCCTTTCAATGACACCTTGTGG	+	-	-	-
	CL1251	ATGGAGAGTCAGCAATGACCAA	CAAACATGACTCGGCCGTAATA	-	-	-	-
	CL1258	ATGGAGATCATCATCACCTTCG	CGAAACCAAACCAAACACCA	-	-	-	-
	CL1291	CCGTTGAGGAGAAACAGAGCTT	GGATAGATCAAACATGGCAACG	-	-	-	-
	CL1310	GTTGCCTTTTCTCTCGCTTGAT	AAAGCGGTTAAAGATGGAGCAG	-	-	-	-
	CL1354	TTGGGCCACTCAAGTGATATTG	CCATCCATCACAAACACAAACC	-	-	-	-
21	CL1206	GTCGAAAACCCTGAATGCTTCT	GAAGGAGGAAGAAAGGCGATTT	-	+	-	-
	CL1259	ACTGCGATCTTGAACAATACCC	GCCACGTGTTACTTGTTCGGTA	+	+	+	RS2CL1259s
	CL1273	GGGAAAACAACAACCACACCTT	TCAGTGGGATCACTCTGCTTGT	-	-	-	-
	CL1293	CACAGCCAAAACAGAGACGAAT	GCAAAGCCAACAAGAGAGGATT	-	-	-	-
	CL1295	AGAGGAACCGGTGTTTACCAGA	AAAGTCAACCGCAGCTCACA	+	+	-	-
	CL1306	ATGCATCCAGAAGCTTGACAAC	GTTCAAATCCGGACAGTCACAG	-	-	-	-
22	CL1363	ATCCCACCCTCAGATTTTACGA	ATCAGGATCAGAAGGGAACCAA	-	-	-	-
	CL1398	GGCGTTACCTTTCTCGATGAGT	TTCCACCTAAGCCTCACACAAA	+	-	-	-
	CL1410	ACGAGTCATTCCGACAACTTCC	CAAAGTACGGACGAGATGGATG	+	+	-	-
	CL1419	TTACCAAGCAATCATGCTGGAG	GACATGAGCTGTTTTTCCTGCT	-	-	-	-
	CL1489	TGTTCAGCGATCACAGACAAGA	CAGAGAACAAGAACTCCCCACA	-	-	-	-
	CL1497	ACGAGGTGTTTGAGTTCCCATT	CAATCTCACACACATGCACACA	-	+	-	-
23	CL1362	TGTGGAGGTGGGATAATGACAG	ATAAGCATTCTGAGCCACACCA	+	+	-	-
	CL1377	GAGCAGTTTTGGCAGGAGAGTT	AGAGGAAAGAAGGAGGCAATGA	-	-	-	-
	CL1380	AGTCTTTTGGAGAGCCCAATCA	CTTTTGGTCGTCTTCGTCCTCT	-	-	-	-
	CL1417	GTTGGTAGCAGCTCTTGCGTTT	TGTCACATACGGTCTCTTGCAC	-	-	-	-
	CL1477	ATTCTTCTACTCGCGCTCGTTT	TTCCCAATAGCCAATCAGGTCT	-	-	-	-

	CL1528	TATAGGCATAGGGTTCGGCAAT	GAGCTTTTGATTGATCCGGGTA	+	+	-	-
24	CL1418	ACCATTGCAGGATTCTCAACCT	GCCTAATGGGTTATTGCCATTG	-	-	-	-
	CL1427	CCAATCCAATCGTTCTCTCCTC	ATCAAGTAGGCAACGCTGTTCA	-	-	-	-
	CL1445	ATTGGGTCTGCGACAGTTGTTA	AAGGTTTATCCTCACGCGACAT	-	-	-	-
	CL1491	GGGGATGGAAGGTTTGAGGTAT	AAGAAGATGGAAGGCAGAGCAA	-	-	-	-
	CL1504	AGAGTCAACCGTATGTGGAGCA	GCTTCGAAACATCTGATGGGTA	-	-	-	-
	CL1512	CGTCGGACACGTATGTGATCTT	CGAAAATCACTCCCAAGGAAAC	-	-	-	-
25	CL1370	CTTTGAAAGCGGAGAAGGAGAA	AGCGTTTACACTTGCCAAACTG	+	+	+	RS2CL1370s
	CL1446	CAGAAACTTTGGAGCTTGAGCA	CTTTGTCCCTTGTTGTCGCATA	-	-	-	-
	CL1451	CCTCCCCTCCTTAACTTACAA	CTGATCCGTTGCTCTCTTCCTT	-	-	-	-
	CL1516	TGCTTGAAATGTGTTGCCTCTC	GGATTCTTTGCTGTCTGCATTG	-	-	-	-
	CL1524	AGGACCATACCAGGGAATCTGA	TCGTGTGAAAGACACTCTGCAA	-	-	-	-
	CL1533	TCACCTGATTGCCTAACAGCTC	AGAACCTCTGGTCATGGCTTTC	-	-	-	-
26	CL1392	GGAAAATCAGCAACCGTTATCC	CGCTTTATAATCGAAGCGTGAC	-	-	-	-
	CL1458	CAGCACGATGGGATCTATTGAC	CAAGGCCATCTCTTTCTCCAGT	-	-	-	-
	CL1438	CGAATTTGGATCCACGTTTACC	AGCACAAGATGGATCAAACCAG	-	-	-	-
	CL1448	AACACACACCTGATGCATTTCC	GACTTTGTGGTGCCTCTCGAAT	+	+	-	-
	CL1434	AACACTGTTTCCGATCGAGTTG	GATTCATTGCACCTTGCAGAAG	-	-	-	-
	CL1527	AGAAGCGGTTTACAAGCCAAAC	AACCGCTGAAAGAAACGAACTC	-	-	-	-
27	CL1536	AGCTCCCCTCGAAGCAAGTTAT	ATCCGAAGACACTCCCTTCATC	-	-	-	-
	CL1554	AGTCCAATCGGTCTGCAGATTT	ACAGATACGAGCTGTGGTGCAT	-	-	-	-
	CL1559	GATCCAGAAACCCGTCATTCTC	ATTCTTACTCCAGGCGAAGCTG	-	-	-	-
	CL1597	GCTTTTCCTCTTCACTGCGTCT	CCTTATCAGCAAGACGAACGAC	-	-	-	-
	CL1673	TATCGTCATCTGGTTCGTGAGC	AAGCAAAAGGAAGCCGTACTTG	-	-	-	-
	CL1679	CGTCAAGAGCTTCTTTGGCTTT	GCTAAAATGGCTGCGAAGAATC	-	-	-	-
28	CL1551	GGAGGAAGACGTATTGGTTTCG	TTATTTCAAGCAACGGGGAGAG	+	+	+	RS2CL1551s
	CL1583	AATCCGAGAGAAACCTCGACAG	ATGGTCTTGGCAGAACTGAACA	-	-	-	-
	CL1587	CCATTGAAGCCCAACTAAGGAA	CGAGCCAATCCAAGTATCAACA	-	-	-	-
	CL1625	AAGTAAACCCTGGTTCGCAGAG	AAGAGAAGCAGCAGAGTCGTGA	+	+	-	-
	CL1635	TGAGGAAGCTGTGAAAGTGGAG	CCAAATGATGGTTACGTGGTCT	+	+	-	-
	CL1669	CAGCCATAGCTGAAGTCGAAGA	GGTCGTCCTGGATACATTGAAA	-	-	-	-
29	CL1542	GGTCGCATTGCACACAAGTTAT	AAATCGTCCTTTTGCTCACCAC	-	-	-	-
	CL1562	TCAGGTTCATCGAGAACAAACG	TTCTTCGTGCTGCTAAGACGAT	-	-	-	-
	CL1568	TTGGACTTTACGACCTCGGTTT	AAGCAAACGATGCCACTCATAG	+	+	+	RS2CL1568s
	CL1572	AGACCCAAACATGTGGGAGAAT	CGAAAACTTAGATGCCTTCGAG	-	+	-	-
	CL1574	GGACACATTTCACAACCACCAC	TCTCGTCAACGGTAGTTTTTCC	-	-	-	-
	CL1606	TACCCAAAAGAACGCCATAACC	GCTACAGATCCCAACACCGATT	-	-	-	-
30	CL1628	GGCACGAAAACCAGTCATCAT	GCAAAGAGACCGAGTTCCACAT	-	-	-	-
	CL1640	GCTCTCTTCTCACAGACCCAAA	CAACCACCCAAGAGCAAAGAAT	-	-	-	-
	CL1643	CAGCGAAACCACAAACACAAAG	GGAGGCTCACCAAACCTTAGAA	-	-	-	-
	CL1684	ACAATAGCGTCTTTGAGCGTGA	TTCCACAAAGCTTCCTCCTTTC	-	-	-	-
	CL1687	AAGCCAAGTCGGATCACACATA	CGATGATCACTCGTCGTTTCTC	-	+	-	-
	CL1688	ACAGCAGATACGTACCGGTGAA	AAGAAGCAGTACGGAAAAGCTG	-	-	-	-
31	CL1546	AGCCTCTCCACCACATTTGTTT	GCAAGTTTCTTTCGCTGTCTGA	-	-	-	-
	CL1556	AGAGCAAAGACGCATCTGACAC	TGAACAAACAACCATCCCTGTC	-	-	-	-
	CL1584	CAGCTCAGGAGAACAACTCCAA	TGTGTTCGAGCCTGAGATGTTT	-	-	-	-
	CL1599	AACAAGAGTTTGCCGACGAGTT	GAGACTCTGCGCCAACAATATG	-	-	-	-
	CL1602	AGGCAAGCAGAGATGAGGAGTT	GGTCAAATCCGAAACCGTACAC	+	-	-	-
	CL1657	GAACGTGTGAACGACTCAAAGG	GGAGCCATGAGAACAATGATGA	-	-	-	-
32	CL1718	CCTCAGGCATTGTCATGTCTTC	GAATAGTCCCCAACCTCATTGC	-	-	-	-
	CL1725	GTGAATGAAATGGAGGAGTTCG	CCAACAGAAAACGTGTGACATC	-	-	-	-
	CL1757	CCTGGAGGAAGCCTTTGATATT	AATCGTAAGGTGGCTTCTGGAG	-	-	-	-
	CL1832	ACAAGAGGCTCCTCGAAACTTG	CTGCCTCTTGGGAGATTACCAT	-	-	-	-
	CL1846	TCCATAGGCAACTCAAGACCAG	AAGGGAAAAGCAGTTCAAGACG	+	+	+	RS2CL1846s
	CL1863	CAGTAACGTCAAGCCCACTGAT	TTGAAGGTCGAGAAGATTGGTG	+	+	+	RS2CL1863s
33	CL1789	GTTTCCGAGACCTTCCGGTTAT	ATCATCAAAGGGTGAACGGAGT	+	+	-	-
	CL1816	TGCTCGAGCTGCTACTATTGCT	CAAGGGCCTATATTCGAGGATG	+	+	+	RS2CL1816s
	CL1868	TTCCTAGCATGGAAGCAACAGA	CAAAAGAGTGTCCACCGTCAAA	+	+	+	RS2CL1868s
	CL1872	TGGAGGAAGGACAGATCAACAA	GGGCAGTCAAATCTCAAATCTC	+	+	-	-
	CL1882	AAGCGGTGAAGATTGGTATCGT	TCCCAAAATGCCTAGAACCCTA	-	-	-	-
	CL1897	GAGCTGCTGAAGGAGTTTGGTT	ACAACTTGGCAAAGCATCCA	+	+	+	RS2CL1897s
34	CL1690	AGATTCTTGAACCCCTTTCACG	TGCCTCTGCCATTCTATGAGTC	+	+	-	-
	CL1724	TATCGGCAATGCTCTTGATGTC	TCCAATCCACACGTCCAGTAAC	-	-	-	-
	CL1749	TGCAAGAAGATGGTGTTCGAGT	CGAGAATGAACACCAAGCAAAG	-	-	-	-
	CL1774	GCTGGGATTGAAGGAGAAGTTG	ACAAACTCATTTCCCCCAGAGA	-	-	-	-
	CL1878	AGCAATGGTTCCCTTGATGAG	ATAAAGTGGGCAGCATCAACAG	+	+	-	-
	CL1896	GAGGTCAGAATCATGCCCTTTC	GAAATGGACTTCCCCAAGAGAA	-	-	-	-
35	CL1717	TCGGATCACACAGAAGGAGCTA	TTGAGCTAAAAGCTGACCCTTC	-	-	-	-
	CL1719	CCAACACCGTCGAGTCTAAGAA	GGAGCAAAGGGATCAGCATTAG	-	-	-	-
	CL1731	AAAGGAGGAGATGGACTGGTGA	GATTACACCGCCAATGAAACG	+	+	+	RS2CL1731s

	CL1739	AAGGAGGAGACGCGTTGTCTAT	TGTTCAGCGTCTCTCACCTGAT	-	-	-	-
	CL1809	AACATGGGCTTCTGGAGACACT	TAAACCGGCCTTACCAACATTC	+	+	-	-
	CL1824	GGAACTTCCCTCGAGAGTCAAA	AAACTTCAGTTCAGGGCATGG	+	+	-	-
36	CL1746	GCATACTTGGCAAATGCAGAGA	GCTGATGTTGACCCTCGTTATG	+	+	+	RS2CL1746s
	CL1748	GGCACTCCTCAGCTTATGACAA	TAGACGGGGAAAACAAAAGGAC	-	-	-	-
	CL1767	AAGGACCAGACTTGTTGGGAAA	CCACACAGTACACAAAGCCACA	+	+	-	-
	CL1822	ACAGTTTGCTGAGCTACGCACT	CTTACACAATGCAACCGTGGAT	-	-	-	-
	CL1850	CGAGTCACACGATGACTTACGA	GGTCACGGCTTGTTTGAGAAA	+	+	+	RS2CL1850s
	CL1861	AGGGTTAGGGTTCTTTGGAGGA	AGACAAAGTTTCGGCACATACG	+	+	-	-
37	CL1776	TTTAGGCACTGGATCCCTTCTC	TCTAGCCAAGCTGGTGTTTTTG	-	-	-	-
	CL1813	GCATACACGGTCCCGTTATCTT	AAATGCTCCGACTAGCGATTTC	+	-	-	-
	CL1819	TCTTCAGCCTTCTGAATTGCAC	CATGGCTGCTGCTACATCTTCT	+	+	+	RS2CL1819s
	CL1842	TCCTTGTTACGTTGCTGGCTTA	AGCCACCACCCATATTTAAACG	-	-	-	-
	CL1871	CCACTCAACGGACTGAGAATCA	AGTTGGCCGGAAAACTCTGA	+	-	-	-
	CL1873	GTAAAACCCCTTGCTCTCCTCA	CGGGGAAAGGTGAAAAGTACAC	+	+	+	RS2CL1873s
38	CL1928	GCGTATTCATCTCACAGCGAAT	TTGCTTGCAGGACGCATATTAG	-	-	-	-
	CL1960	AGCTGGGCTACCAGAGTTTTTG	AGCAGAAGACGTAAATGGATGG	+	+	+	RS2CL1960s
	CL1994	GCCTGGAAAAGTAGTTGGGTCA	CAACGTCAAAGGACAAAGGTCA	+	+	+	RS2CL1994s
	CL2008	GCCCAAGTCACCAAACCTTAAC	ACGACAAACGGACAAAGAAAGG	-	-	-	-
	CL2032	TTCCAGTGTCTCTCTGGGTTGT	ACAGCAGCTCTGTTGGTGTTTC	+	+	+	RS2CL2032s
	CL2043	TCTTCCTTGGTGGAGCTTGAAT	TGGACGGTTAAGATGACGAGAA	+	+	+	RS2CL2034s
39	CL1947	GATTGACGAGAACCGTACTGGA	CTCGATCGGATGGTACAAACAA	+	+	-	-
	CL1982	CTTTTTCCCAGTGAAAGCTTGG	AAGTTGTGCCTGAACCTGAACC	+	+	-	-
	CL2005	CCTATTTAGGAAGGCCATGGAA	CAGCACCACTTGTGTCAGAGAA	-	-	-	-
	CL2016	ACAACAACTTAGGCGGAGGATG	ATGAAAGCTAACCCGAAAGACG	-	-	-	-
	CL2044	TCTGATCTGAACAACCCGAGAA	TTTACGACTCCAGGGGAGAAAC	-	-	-	-
	CL2064	AGCGATAGGGAAGCTCATGTTC	CACGGTCCACCACTTTGAGTAA	-	-	-	-
40	CL1931	CTAGATCCTTTGCAAGCCCATC	ACTCCATCAGCTCCTTCGTCTT	-	-	-	-
	CL1946	CCTCAATGGTCTGGTTCGAGTT	TCGAGAGACCCTGCTCAAGTTA	+	+	+	RS2CL1946s
	CL1988	GACCAATAGAACCCCAAGCAAG	CTTTGCTCTCACCCACAACAAC	-	-	-	-
	CL2018	TCAACCAGGCTCGAGATTCATA	TGCTCCTAGAGCGGTTATCGTT	-	-	-	-
	CL2071	AAGGGAGAATCATCATCCGTTC	TGCGTCACCTTAGAGCTGTTTC	-	-	-	-
	CL2085	TCAGGCTTATATCCTCCGCTGT	AATCGAGTTCTTGCCTTTGACC	-	-	-	-
41	CL1949	TCGGACATTTCACATGGAGTTC	GACTTCCTCCGCATCTGAAACT	+	+	-	-
	CL1950	ACATGTGCTTGACACGGAAAGT	CCCCAACAGAAACTCATCACTG	+	+	+	RS2CL1950s
	CL1961	AGTTGCCTCGTAATCAGCTTCC	GGCAGTCGTTTCGTCTTCTTCT	-	-	-	-
	CL1972	TCTGAACCCAACTGCTCTTTCA	CAACATCGCTTTTAACCGTGAG	-	-	-	-
	CL1990	TCAGCCCAAAGAAAGAAAGACC	AGAGGTGCTGATGTCGCTGATA	+	-	-	-
	CL2025	GCATTCATTTCCTCTGCATCG	GGCTCGAGCAAGAATTAACCAA	+	-	-	-
42	CL1927	AGCAGAAAGGTTTGCAAAGGAC	TCTCAACCCAATCAAGCCTACA	-	-	-	-
	CL2014	CTCAGCAGCCTAAGCTTTTCGT	ACAACACTCGAAGACGGTTTCA	-	-	-	-
	CL2024	AGATAATCAGCCTCCGGTGAAG	GCTCGATTTGAGTCACACCAAC	-	-	-	-
	CL2029	TTATCTCCCATACGCTCCATGA	GCAATGACACTGCTGGCAATAC	-	-	-	-
	CL2072	CCAAAATCGGCGAGAAAGAG	CGTCAATCTCAGGCAACTCAAG	-	-	-	-
	CL2073	TTCTTGGACCCATGTACAGCTC	TTCATGACAAGGAACGTCTCTG	-	-	-	-
43	CL1901	CCAGGTAAAAGCAGTCCAAAGG	CATGAACCCAAACGAGCTAGAA	-	-	-	-
	CL1937	GATCCAACCAACCAGTTTCTCC	CGCGACCATATTCAGTGTTCTC	-	-	-	-
	CL1998	AAGCTTCCACTCAACTTCACGA	TTTACTCCGACCCACTTCCCTA	+	-	-	-
	CL2009	AAGGGAGCAGGATCAGAGAATG	ACACGTAGAAGCACTGCGAAAG	+	+	-	-
	CL2046	GCCATCGTCTTCGACCTTTACT	TCACTGCTGCAACTGATCGTAA	+	+	-	-
	CL2061	GACTTTGTTGTATGGCGGGTAT	CTCTGGTTTTCACTCGTTCTCC	-	-	-	-
44	CL2118	AGATCTTGCCCGTTGTCAGATT	GCCATCAGCTACCAAACACAAC	-	-	-	-
	CL2134	CTTCCCTTTCCCTTTAGCAGGT	GCTGTAAAGTTTTCGCCGAGAC	-	-	-	-
	CL2136	CCCTAGCTTCTTTCCTTCGACA	AGATGGTTCTTCGGTCCTCGTA	-	-	-	-
	CL2162	GGAACTCTTCAAAGCCATTTGC	CCCTCACCTCCTTTGTGTCTTT	-	-	-	-
	CL2190	AAGATCGGAGGCTACGACATTC	ACACCAAGACCATCAGCATCAG	-	-	-	-
	CL2191	GATCAGGGACAATCCCATTAGC	AGCCTTCAGTGTGCTAGGTTCA	+	+	-	-
45	CL2090	CAATGCTCAGCAACCAAAGC	AACACAAGGGTGGAAAACGAAC	+	+	-	-
	CL2169	TTCTTCTCTGCAGGCATTATCG	CTGTCTCTTGTTTGGCGATGTT	-	-	-	-
	CL2241	GAGCTGATCAGGCCAGAGAAAT	CTCCAAGATTGCCTTCGAGTTT	-	-	-	-
	CL2256	AAGGCATTCATAACTGGTGCAG	GTTTCTTCTGCAGCCTCCTTTT	-	-	-	-
	CL2261	GGGATCACAGTTCAAAGGGAAA	AACGCGAGTAGACAGCAAATGA	-	-	-	-
	CL2265	GCATATTACAAACCGCAAGCAG	GAGAAATCGACGACGAGAACAA	+	+	-	-
46	CL2088	CTGTAACTGGACAGCCAAATCG	ATGAAGAGGCTGATGGTGATGA	+	+	+	RS2CL2088s
	CL2156	GGAGCCTTGTCCTCAGAAAAGA	GAAACGTGTGGAGAACCAGAGA	+	+	+	RS2CL2156s
	CL2175	CAAAGCAGGTTGTGTCCTTTTG	ATGATCAGGAATGGCCAAGAGT	-	-	-	-
	CL2202	GACGATAAATTCCGCAACCTTC	GCTGTTGTCACTTTGGTCTCCA	-	-	-	-
	CL2237	GGTGTGGTGCTACTTGAAATGC	TATAGCTCCCAGGACCGAAATC	-	-	-	-
	CL2253	TACACCAGCCAGCTTTAATCCA	TACTATCTACGTCGCCGCCTTT	-	-	-	-
47	CL2104	GGAAAGAATCGAAGCAGTGGAT	TAGAGATGATGGAAGCCCCTTA	-	-	-	-

	CL2112	AATCGATATGGATCAGCCCAGT	GATGGTTAATCCGATTGGTGTG	-	-	-	-
	CL2148	ACCATACAGGCTTTGGAGGCTA	GGCGTTTACACGACGAATGAT	+	+	+	RS2CL2148s
	CL2201	AGGAGGAAACGAAGAAGCATTG	GGAAGAGTGAACATTCCACGAA	+	+	-	-
	CL2234	CAACAACAGGACAAATGGGTGT	ACTCTGTTGGTTTGTGCCTTCA	-	-	-	-
	CL2273	AGCCTTTTCTGTGGGTGGTTAG	CAAAGCAAACAAGAGGTGGACA	-	-	-	-
48	CL2110	GAAGAAATCGGCGGAACCTAAT	GAACAAGGGTTCCACACAACAA	-	+	-	-
	CL2132	AACAGAGCAAAGGGACTGGTGT	ACAAATGGAGAAGCCAGTGACC	+	+	-	-
	CL2193	TTGGGTCTGAGCTTTGATTCAC	CCCAACTCAGCCCTTGAATTAT	+	+	+	RS2CL2193s
	CL2223	GATGCTGTTCCTCAACACCTTG	GCATGCATTCCAAAGATACTGC	+	+	-	-
	CL2224	CACTTCCACACCACAAGACACA	GGAACAAGTCGGATCTGAAGGA	-	-	-	-
	CL2262	TTCCTCTTCGCCTTCTTTTCTC	ACCCGGTTTCCAAAAACCAT	-	-	-	-
49	CL2161	CCAAATCTCAAGCGACCAAA	CGCGGGTAGAATATTGGAGAAC	+	+	-	-
	CL2164	TTTCCCAGGGACTAAAACCAGA	GTCCTCTTTCCGTTCCAAACAC	+	+	+	RS2CL2164s
	CL2168	AACCTGGAAGCTAGAGCAAAGG	ACACGTTCACCAGCCTCTAATC	+	+	-	-
	CL2185	ATGCAACCTCCATTCCATTACC	ATGGGAAGAATCCAGCCTGTTA	-	-	-	-
	CL2222	CTTGAAGAGCTCCCAAAGTGCT	TCTGACAATCAACCCCTTCTCA	-	-	-	-
	CL2248	TGTTTTCAGTCACTCGCAGACC	TGTGATCAAAGTGGCAATCTCC	-	-	-	-
50	CL2295	CCCCATCAGGTGAATAAGGAAG	ATCCGAGGTGAGAAAACTCAGG	-	-	-	-
	CL2306	GATTGGTTTGGAGCTAGGGTTG	TCCTGCTATGGCTCCAGGTATT	-	-	-	-
	CL2382	GATGGACTTTGACGAGGAGCTT	CTCCCTCTTGGGCTTAGATTCA	-	-	-	-
	CL2401	AAACTTAGGCTCTGCCCTTTCC	AGGAATGGATGTTGACGAGGAT	-	+	-	-
	CL2458	GTTTGCCTGCTTTGTCTTCCTC	GATCATGGCAGCAACTTTGAAC	-	-	-	-
	CL2479	CACATATCGCCTCTCCTGAAAA	CTTCTGGATTTAACGGGCATGT	-	-	-	-
51	CL2285	CTACGACCGATGCAGTTGCTAA	CACCTCGGTTTGAGCTTCTTCT	+	+	+	RS2CL2285s
	CL2359	AAGAACAGGATCACGATCACCA	GCAAAAGAATGCACCAGCTTAG	+	+	-	-
	CL2392	CAGACCAGACATGGGTCACATT	CCCAACAGGAATGAGAGTTCA	-	-	-	-
	CL2400	TGGGAACTTTGTTTCCCTTCTC	GTTTAAAGCCCTTGCTTCTCCA	+	-	-	-
	CL2413	CAGGTTGCGTTTGTGACTTTCT	CAGAAACAGTGGGTTGAAGCAG	+	+	+	RS2CL2413s
	CL2422	CCGATCAAGATCTCCAGTGAAA	TACAACATGCTGGAGTGCCTGT	-	-	-	-
52	CL2298	ATCTGCGCTTCACCGTAAAGTT	TGGTTCCTTCTTCACATCATCG	-	-	-	-
	CL2351	CATCAGAAGATGCCATGAGAGG	TCATGGAGCAGAGGAAACGTAA	+	+	+	RS2CL2351s
	CL2364	GCAAAGGGGGCAGACTTTTATT	CAGCTTTGTATCCGTCGTTGAA	-	-	-	-
	CL2407	ATTGGCCCAGAGGGAAGATAGT	TTTGACCATAACTGTGGCGTTC	+	+	+	RS2CL2407s
	CL2436	CTGTCCACAATCGGCGATAA	CCACAGAGTGATCCCTGTTTCA	-	-	-	-
	CL2469	GGGTTTGAAGCACTCTTCTTCC	GAAGTTGCAAATGGAGGAGCTT	-	-	-	-
53	CL2279	TCATCATCACTGGAGGACGAGT	ACGGTTTTTGGAGTCAGTCACA	-	-	-	-
	CL2315	AAGCTAAAACTTGGCTGCTTCC	TGCCGGTGAGAATAGAGGTGTA	-	-	-	-
	CL2342	GCGTTACCAGGATCTGATGAAA	GATGCTGATCAAACGCACAAAC	-	-	-	-
	CL2434	ACGAAAGCCTACACCGAGATTT	AGTTGTAGCCATTGCAAGCAGA	-	-	-	-
	CL2453	CCACAGCTACAAAAGAGGCTGA	AACGCGGTTTTGCTCAACTT	+	+	-	-
	CL2474	ATGCATCTTCCCTGGCTTTAGA	ATCCCACTGAACCAACAAGAGG	-	-	-	-
54	CL2318	TGCAGATGTGACTTATGGAGCA	CACACACACATAATCCCCCAAT	-	-	-	-
	CL2345	CTCTCGTCGCCATTGAGTTATG	ACGCCAAAGTCTTCTCGTCTCT	-	-	-	-
	CL2371	CACCAGTCAAGCAAACAGAGGT	CTCTCGCCAAAGTTCCTTTGAT	+	+	+	RS2CL2371s
	CL2388	GGATTTAAACCCAAAGGCACTG	ATTCGTGGGTCCATCCTCATAG	+	+	+	RS2CL2388s
	CL2435	TCGTTCTCGTCAAGCCCTCTAT	AACCTCAAACCGGAAACTCAAG	-	-	-	-
	CL2446	TCATTCCCTCCAACCTCTCTCT	CTTTCTGTCCAGCTCCTTGGAA	-	+	-	-
55	CL2274	TCGTCTTCAGGCTTCTTTCCAT	AAAGGTAGCAACGGAGGAATCA	-	-	-	-
	CL2358	CCATCAACATCCATTGCCTCTA	GCAGGTTGTAAAACCATGTGGA	-	-	-	-
	CL2377	GGGGAGAGTCAAACACCATTTC	TGAAACCGCTAATACCGAGTCA	-	-	-	-
	CL2432	GTAGGAGACGAACGCTCATCAA	AATCCAGAGGAGGCTATTGCAG	+	+	+	RS2CL2432s
	CL2438	TAGATGTGCACGAGCAAGAAGG	CAAAACGGAACAGGGTTCAAAG	-	-	-	-
	CL2467	TAGAAAACCCTAGGCCAAACCA	CGAGGAGCTCAAGAAGGTTTTC	-	-	-	-
56	CL2567	GATGACTTTCCAGCTGCGTCTA	ACGTGCGGAAGAACTGAAGAAC	+	+	-	-
	CL2579	GCACTGCTTTGGTCACGTTTAG	AGGAATTTCACACGGCTCTCTC	-	-	-	-
	CL2594	GATCTCCCCCGAGCAAAAAT	CATCAATCATGGTGGGAGCA	-	-	-	-
	CL2598	GTTTGGAGCTCGAGGTAAGGAA	GTAACAACATTGGGAGCGAGTG	+	+	+	RS2CL2598s
	CL2625	GGCTGAATGCAACATCTTCAAC	ACCCTGGAGAGGTTAACCGAAT	-	-	-	-
	CL2696	CTTCCAACCATCAACAGGATCA	CAAGCATGGTTGGTTCTTTCAG	-	-	-	-
57	CL2520	GGATGGGGAGAGTTAATGTGGA	ATGGCTCTTGGTTTAGCTCTCG	-	-	-	-
	CL2542	GGGAGAAACAGGAGATGCAAAC	GGCTTGTGGAAAATCTCTTGCT	+	+	-	-
	CL2559	TCTCGTGCCCGTATGTATGTTC	ACAAGGATAACGACACCCCAAG	-	-	-	-
	CL2580	TTGCTACGCAGGTCCTATGTTC	CGCCCAATAAGACAAGTCCAA	+	+	+	RS2CL2580s
	CL2584	GCATGAAGAGTGGAAGGAGGAT	CATCTTCTGAACGGTCCCTCTT	-	-	-	-
	CL2614	AGCTACAACGGTATGGCTGGAT	CGCCTTTGCTCAGTTTAAGTGT	-	+	-	-
58	CL2482	GGAGGTCCAAGCCTAAGAAAGA	GCACTCAACCCCCAAGATTAAC	-	-	-	-
	CL2489	CTATCAGGCGATTCCAAGAACA	CAACGACATGCTCTGATCCTTC	-	+	-	-
	CL2491	GCGAGTAAACAGGCTGTGAAAA	CAGTGCTTGAGCAACGAAGAGT	-	-	-	-
	CL2588	AGTTATAGACCCGGCGTTCTCA	CTTCAACCCATTGATCCGTACA	-	-	-	-
	CL2590	CACGTAGGGCATTTAGCTTGAA	AGGTCACCACAGGTGAACAATG	-	-	-	-

	CL2646	AACATAACATGGGACGTTCTGC	GAAACAGGGGAGAAACAAGAGG	+	+	+	RS2CL2646s
59	CL2496	AACGAAACAGAATGTCCCGAGT	TGTTGATACATCCCAAGCAACC	+	+	+	RS2CL2496s
	CL2505	ATCCTTCTCGGCCTTGTTGTTA	CTCAGACGTCATTTCTCGGAGT	-	-	-	-
	CL2551	TCAGGCCATTCCACTTTACTCA	AACCCTCTTCGAAACCAGACAA	-	-	-	-
	CL2583	ACGGACAAGAGAGCAAGGGATA	AGGATTAAGCAGCTATGGTGGA	-	-	-	-
	CL2621	CGAGAACTACGCTCAAAACTGG	TCACAAACCCCACAAACACTTC	-	-	-	-
	CL2640	CCAGCTCGATGGTTACATTTCA	AAATGCAAAGTGCTGCAGAGAG	-	-	-	-
60	CL2486	AGATGGCGACACACAATGTTCT	TCCAGATTCGTGAGAGAACTGC	-	-	-	-
	CL2499	AGCTGCAAACTTGCCTCTGTT	GGCATTGGTTACCTCAGTCACA	-	-	-	-
	CL2511	TATCCTCCCGAAGACGGAATAA	GGAGGAAAGTAAGGATGCCAAG	-	-	-	-
	CL2519	TTCCAGCGTTGATGAAGCTAAC	CATGTAGCGATGGTGGTGAAAT	-	-	-	-
	CL2622	GATCAATACCCCTGTGGCTTTC	GATCAGACGAAGCGTATGAACG	-	-	-	-
	CL2633	TAGCAAATCCAGGTTCTGTTGG	AGCTTTGTTCATGATCGGTTCC	-	-	-	-
61	CL2484	CTCTGCAATCACCACATTCTCC	GGATGGAAACACAAGGCAAAGT	-	-	-	-
	CL2523	TAAGACCGTGGAATTGGTAAGC	TCCTAAATCCATCTGGGACTCG	-	-	-	-
	CL2586	CGTTCTCCATGAACTCAGCTTG	TCGAGATCCTTCCCCAAATTC	+	+	-	-
	CL2627	GAAGATGGTCGTGTCGTTGTTC	TGGAAAACAACTCCCAGTACCC	-	-	-	-
	CL2638	ACGCACTTGTTGGCTATTGAGA	TGATACCTGGCTCCTTCAACAA	+	+	-	-
	CL2675	CACAAGCAAAATTCCAGACGAC	CGAATCACAGTGTGTTGCTCAG	-	-	-	-
62	CL2744	TGCGGTTGTAAGCAGCACTTAT	TGCTTACCAACACGGTTCTACG	+	+	-	-
	CL2749	AAGGAGCTGGTGAAGAAAGCAG	CATACCTGCTTTGGTCGTTTTG	+	+	+	RS2CL2749s
	CL2801	TGTCCTCTCAGCATCCTCTCAA	TAACACTGCGGTGGTGTCTTCT	+	+	-	-
	CL2822	ATTCCTCATTGGATCCATCTCC	TGCAGCGTCAGATCTCTGAATA	-	-	-	-
	CL2830	AGCCGTGGAAAGGGAAAGTTAT	CGACGAGTCTCTCAAGTTCCAA	+	+	-	-
	CL2884	TCATCGATGGTGTGTCTTTGTG	AGACACAGCTCAAATGCATGGT	-	-	-	-
63	CL2724	TCTTGTAAGAGCAGGGAAAGCA	GAAAAGCTGCAGAAAGAGCTTG	-	-	-	-
	CL2815	TTCTCCACTGAGGTGTGGTGTT	TCTGCTGAAGATTTCTCCGTTG	-	-	-	-
	CL2821	GATCTTGACCGCTTCACCTTTC	TCAAGGCTGGTGTCTACAAGGA	-	-	-	-
	CL2858	GGTGGCATGGGATTTTGAGTAT	TTGCCTCTGCATTTCATTCC	-	-	-	-
	CL2862	TACATAAACGAGCCAGGCACAG	GCATAAGCAGGGAGAGGTCATT	-	-	-	-
	CL2919	TACAAGAAGGCAGGGTGAGAGA	ATACCACAACAGTCCAACCACA	+	+	-	-
64	CL2747	CAATAATTACGTTCCGCAGCTC	CAGCCAGCTCATACGACAAATG	-	-	-	-
	CL2775	GGAATCAACTACCAGCCTCCAA	TTGCTTGAGAGAAGAAGGTGGT	+	+	-	-
	CL2795	CAGCACGGAAACCAAGTTGTAG	CCTTCACCGAGCTTCTCAGATT	-	-	-	-
	CL2799	TGTTCCTACGAGCAAACTCAGC	AAGCCTTGGCAGAAAAACTCTG	-	-	-	-
	CL2831	CAGTTGAGATGATCCCACATGC	TGTTCTGGTCTAGGCATGATCC	-	-	-	-
	CL2895	AAGCTATCCATGTACGGCACCT	AGCAACACCAAACAAGGAACTG	-	-	-	-
65	CL2706	CAGGCGAGGTACGTTTCTCATT	CACACCAACATAAACCGGAACA	-	-	-	-
	CL2725	ATCCGAATATATCGTGGCCTTG	ACAATCTGAACGCAAGAGTGGA	-	-	-	-
	CL2841	TGTCCAAAACTCTCAAGCCAGA	GAACGCATTGAGTTTCCTTTCC	-	-	-	-
	CL2861	ACCGAACCGTCTGTAAACACAA	CTGCAAACTCGAAGATCTCACC	-	-	-	-
	CL2881	TCTCGTTCTTCAGCGAGAGAGA	AGAAGCTCTGTATCCAGCGAGA	-	-	-	-
	CL2909	AAGTGCCGTTAGTGACACTTGG	AACAAAGTGGAGGCAAAGGATG	+	+	-	-
66	CL2701	ACCCATCACACACTCCTCTCAA	CGAGACACCATCTTCGTTCTTG	-	-	-	-
	CL2730	CTTCAAGACAAGCCACACCAAC	TCCTCGGAACGCTATGCTTTAT	+	+	+	RS2CL2730s
	CL2759	CCATTTTCAAGCTCATCTGGTG	AGGGAGGAAAGCAGAGAAGGAT	-	-	-	-
	CL2788	GAAATCAGCCGTTTCAGTAACC	CTCTCTCCTGCTCCAAAGGAAT	-	-	-	-
	CL2798	TGACCTCAAATGGAAGGTGTGT	GGTTTGGTTAAACCGATCTTGC	+	+	+	RS2CL2798s
	CL2871	CTTCGTCTCCAGCAAATGAGAA	CCGCTCCTTTGAGAAAGCTAAA	-	-	-	-
67	CL2735	CCCTAAAAACCCGAGATTCCA	CTTTAGGAGACGGATCGGTTGT	+	+	+	RS2CL2735s
	CL2745	AACCCAAGATGACAGGAGAGGA	CAAAGCAAATCGAAGCCACA	-	-	-	-
	CL2771	AGGAGTTTGAAAAGGGCAACAC	CACTAATCCGTGGAAGATGACG	-	-	-	-
	CL2811	CAAGCACTCAAACCCATCTCTG	ATCAAAACCCTTCTCTCCACCA	-	-	-	-
	CL2859	CACATCAATCATGCAGCAGAAG	GCCTGCATCTCCACATTGACTA	-	-	-	-
	CL2908	TAGTAGGCTGGACCAACATCCA	ACTCTCGTCTTTCAACCCCACT	-	-	-	-
68	CL2710	AAGAACACGACATCCGAGACAA	AGCCCATGTAACGAAATCCAAG	-	-	-	-
	CL2729	GGGAAATGAGTTTGGTTTCTGG	CTAGTGGATCAAGCAGGCCTTT	-	-	-	-
	CL2781	GAAACGGAGGCAAAGGATGTAG	ACAATCTGCAGAGAGAGGCAAA	+	+	+	RS2CL2781s
	CL2832	AGCTGGTGAAGGTGAGGTTCTT	GATCCTCCATGTCCAAGCTTTC	+	+	+	RS2CL2832s
	CL2860	GGGTGGCATTGTAGCTTCTGTT	TGGTACACTGATGATGGTGGTG	+	+	-	-
	CL2891	CTTTCCGTCTCAAAACTCCAAG	GATTCCCACCGATACGAAAGAG	-	-	-	-
69	CL2934	TCCCTGAGTCAACGTGTCTTTC	GGATCCGAAAACGTCTTGTTCT	-	-	-	-
	CL2983	ACTGGTGTGCCTTGTGATGTCT	TTTCTTTGGCGAGTCGGTCTAT	-	-	-	-
	CL3039	ATCTCGGTGTACATCTGCTCCA	TTTTGTCTCCTCTTCGGCTGAT	-	-	-	-
	CL3097	AACCTCGCATGGGTTCATAAGT	GTTTCACGTCTTCATCGCATTC	-	-	-	-
	CL3117	AGTCAAACTCCTCGTGATGCTG	CTGTTAACGATCGGCTTCTCCT	+	+	-	-
	CL3127	ATTGAGTGTGGTGTCACGGAAC	GGAGCTTGATCTCTCCCTTGAA	+	+	+	RS2CL3127s
70	CL2935	AAGATCCTTGTCAATGCCTGGT	GTTATTTCAATCAGCGCACCAC	-	-	-	-
	CL3000	TGACAATGACAGGAGGAGCAGT	CAAAAGAAGCCTCAAACCGTCT	-	-	-	-
	CL3017	CCGGAGAAACCGAGAAACTTTA	ACGAGTTGTAATGGACCAACGA	-	-	-	-

	CL3067	AAGAAAGGCGTAAGCAACGAAG	TGCTAACCCTGAGTGGATGAAA	-	-	-	-
	CL3074	TTAGATTCCACGGTTGGGAGAT	CAAACCCAACAAACCACAGAAG	-	-	-	-
	CL3155	CGTACTTGCCGTAGTTGTCGAA	TCCAACTCCAGAGGAAAGCAAT	-	-	-	-
71	CL2943	ACAGGAGTCGTTGGAAAAGGAG	CCCATATCACAGGTCGCTACAA	-	-	-	-
	CL2994	GTGGGTTAATGGGACCAGGTTA	CATCACACGTTCACAAGACGAC	+	+	-	-
	CL3005	TACTTCTCGTCGTCGCCCTTAT	CTCCTTTGAACGCATCAACATC	-	-	-	-
	CL3013	GGAAAGCCAAAAGTAGCAAAGG	CATACATACCATGCATGCACCA	-	-	-	-
	CL3113	ACAAAACAGAGAGCGAGTCGAA	CACCATCACCATGTTGGTCTTT	-	-	-	-
	CL3154	TGGTGGAAACACGATTAGATGC	GGCACCATTTGTTCAAACAGC	-	-	-	-
72	CL2929	GCCTCACAAGATGTCAAGATCA	TGACGAAGGCGAAGAAGAAAGT	-	-	-	-
	CL2963	ATCCCTCCCCCAATCTTATTGT	GCAACAAGAGATCCTCGATTCA	-	-	-	-
	CL2981	CCGAAGCTCAAAAAGCTTCATC	CGTTGTGCGTTAGGAGAAGAGA	-	-	-	-
	CL2985	CAGCATAAGCAACGAGATCACC	GGTTATCCACGAAATCCCGTTA	+	+	-	-
	CL3006	ACAACAACCTCATCTCCCGTTT	TCATGGAGGAACCCATCATAGA	+	+	+	RS2CL3006s
	CL3015	CAGCTGTTGTGGGTATTGGAAA	TCCTTGGTTGATCTGGAACTGA	+	+	+	RS2CL3015s
73	CL2950	TAATCCTCATCCACTTCGCAGA	AAACCCTAGCTTTCTCCGTCTC	-	-	-	-
	CL2965	TGCCATCGGTTTGAGTCACTT	AAGCCAAAGCAGAAGCAATGTC	-	-	-	-
	CL2969	GGGACAGTCCTCAACAAGTGAA	TCCATCATTAAGTCCACGACCA	+	+	-	-
	CL2980	GGGAACCTGTTCCATATGGTGT	TACAAATCTCAAGCTCCGTCCA	+	+	-	-
	CL3082	TGACTTACGGGACATCAAAACC	AGATGAAATGTGTCCAGCCTTG	+	+	-	-
	CL3126	AACCGGTTTACTCGGTTCACTC	TGCAGCCAGAGAAGGATATGTT	-	+	-	-
74	CL2744	TGCGGTTGTAAGCAGCACTTAT	TGCTTACCAACACGGTTCTACG	+	+	-	-
	CL2880	TGGAGAAACCACCCTACACTCA	GCACACACACTGCATAGGATGA	-	-	-	-
	CL3027	AGAGGAAGTGGATCCAAACGAG	TTTTCCTCAGGCTCATCCTTCT	+	+	+	RS2CL3027s
	CL3559	TTGGAAGAAGTGCAGGAGAACA	CCTCTGGGATTTTTCATCAAGC	-	-	-	-
	CL3612	TCTCTCATAAACCCCCTTCACC	ACGTCGTGAGAGACTTTTCGTG	+	+	-	-
	CL3708	AATAACATCAACCAGCGAGAGC	TTGGAGGTGTGCTAGGGTATCA	-	-	-	-
75	CL2948	AGTCCGACCATCTTTGTCTGTG	AGTCTACGTTCTTGGGGTTGGA	-	-	-	-
	CL3032	CAGCAGACATCCCTTGTTTCTG	GGGAAAGACTGTGGTATGAGCA	-	-	-	-
	CL3116	CAAAGCGGTTCATGGTGTTTAG	GGATGGTGATCCAGGGATTTTA	+	+	-	-
	CL3119	CTTGAACATATTCCGGGGAGTC	AACTCGGAATCCAGAGATGCAG	-	-	-	-
	CL3394	CCTCACCATAGTGGAAAGATGG	AAAGTCCCATGTTTGGTCGTAG	-	-	-	-
	CL3489	AACACACTGTGGATGGAACTCG	CACAGGGTTCACAGAGGATTTG	+	+	+	RS2CL3489s
76	CL3180	AGGCCAAACGTAAAAGCTATCG	TTACACCGACGACTGCAAAGAT	-	-	-	-
	CL3263	ATCATGATCACAATGGCTCCAC	CTTGGAGGGATGAGGGAAAATA	+	+	-	-
	CL3301	ATCACACACGGTAACCACAAGC	AAAGCGAATCCACCAGGTTCTA	+	+	-	-
	CL3318	TGCGACTGATAAAGGATGAGGA	ACCCCCAAGTAAACCAAAACCT	-	-	-	-
	CL3324	GTATTGGAATTTAGCGCCCATC	AGAAGATCCAGCATGCAGAACA	+	+	-	-
	CL3325	TGGAGAATGGCTGTGAAGAAGA	AACAGAGCCTTTTGGCAGAGAC	+	+	-	-
77	CL3230	CGAATTGCAAGCGGATACTATG	ATTGAGCGTTGATAGTCGCGTA	-	-	-	-
	CL3261	GAGGAGGACACTGCAATCTTGA	GCGTTTGATATGTCCGTCAGAA	+	+	+	RS2CL3261s
	CL3333	ATCCCTGCTTGATGTGGAATCT	ACCACGTATGGGACATGCATTA	+	+	-	-
	CL3341	GGGATATGGTATGCAAACAGGA	ACAAAGAGGAACAGCCTTTTGG	-	-	-	-
	CL3356	CGGCGGTATATTAAATGGAGGA	CATAGAATTGACCGTGGGAACA	+	+	+	RS2CL3356s
	CL3387	GGTAAGAAGGCGACAGCTTTTC	GCTGGAGTGACAACTGACTGAA	-	-	-	-
78	CL3208	AAACCTCCGGTGTCTAGGAGTT	AGAACACACATAGCACGCCATT	-	-	-	-
	CL3279	TAAGCGTTCACGACTCATCCAT	GGTTCTCCTGAGCGTCAAAAAG	+	+	-	-
	CL3283	TGTATCGGTTGAGTTGGGTAGG	TTCTGGACACTCACTCCAGGTT	-	-	-	-
	CL3286	GGAAGGCTCCAAGATCCTTGTT	GCCAAGTGATCGGCTTCATTAT	+	+	-	-
	CL3326	CTCCTCAGCCTTGTAAACAGCA	ACGCAGGAAGGGAAGGTTTTAT	-	-	-	-
	CL3360	TCGACACGAGTTCTGGTCTCAT	GTAAAAGGTTTGCACGCATCTG	-	-	-	-
79	CL3202	AACACCGTACCAACCAAATCCT	GCCAAATCCTCAGTTCCAAATC	-	-	-	-
	CL3291	ACGACGAAGGATACCTCCAGAT	TCAGCAAAACGACACAGATTCC	-	-	-	-
	CL3293	AAAACTTGTAGTGCCGGTGACA	ACCCACAAACAACAAGTGGACA	+	+	+	RS2CL3293s
	CL3339	GAGTTTAAGCCGGAGAGGTTCA	TAGACCAGCTTCCTCTGTGGAT	+	+	+	RS2CL3339s
	CL3345	CCAAACGTGGTGGGATTCATA	CAAGGCCCAAGAGAAAGAGAAA	+	+	-	-
	CL3347	ATCTTTGTGAACACGAGGCGTA	AAGAAGTTACCATGCTGCCACA	+	+	-	-
80	CL3197	ACTGCCACAAACCGAAGTCTTT	CAGCTGAAACAGAACCTCGTTG	-	-	-	-
	CL3234	CGTAAAGAAGGGGGTGTAATCG	CAACCAAAACGACTCTCACTGC	-	-	-	-
	CL3258	CTCTGGTCTCGGATTTGGTTTC	TCGAGATGTATTCCGATCGTGT	+	+	-	-
	CL3317	GCAGTTTGTGAACATGGAGGTC	GGCTCGAAGTCTCGACATAAAC	+	+	-	-
	CL3352	AAAACCGGGATTGAGTTGACAG	TTCCTCGTCGATACAAGTTTGC	+	+	-	-
	CL3362	TGAGAGCTGTGGTTGAAGAAGG	CACATCAACCCCAACAGAGAGT	+	+	-	-
81	CL3160	AGATGCTCGTTCTTGACCCTTC	AACCAGAAAAACAAGGCAGAGG	-	-	-	-
	CL3183	TCCTGAACGTCCAGAACAAGAA	GACAAGGCATTGTGAAGGAAAG	+	+	-	-
	CL3216	ACGGAGAAGCAAGGAAAGTACG	TCCTCTCGACCAGAAGTTTTCC	+	+	+	RS2CL3216s
	CL3306	TACTTGGTATTTTCCGGCCAAC	TCTGGTATTGTCGGTGACTTCG	+	+	-	-
	CL3313	CCGATGATTCGTCCGTGTTA	CGAACTGCAATCAGCCATTC	+	+	+	RS2CL3313s
	CL3351	CAAACACCCCACTGCTTGAGTA	TCCCTTCTGACAGCTCAATCAA	-	-	-	-
82	CL3411	TCGTCGTAGGTAAATGGGAGGT	CCATGGCTGCTAAACTTGTGTC	+	+	-	-

	CL3492	CAAAGATGGCCTCAACTTCAAC	GACAACATCGCGAAAGAATCAG	+	+	+	RS2CL3429s
	CL3516	GCTGTCTTCCATGACTGCTTTG	TATCATGCACGCGTGACAAAC	+	+	-	-
	CL3531	CCAAAAAGGTATGGGATTGAGC	TCTCCCAGAGGAGAAAGAAGCA	-	-	-	-
	CL3575	AATCAGCGACCAGAGATCATCA	TCACTAGCTTGCCTGAAAGTGG	+	+	-	-
	CL3656	AAGCTTACTTCCTGCCGAGAAA	AGGAAGCATTTCGTCGAACAAC	-	-	-	-
83	CL3404	GTTTCATAGCAGCAGGTTGACG	TCATTCGTTTGCCTCTCTCTCA	+	+	-	-
	CL3444	TAGGGTTTCTTCCATCCACGAT	ACCACCACCAGTATCATCCTCA	+	+	-	-
	CL3534	TTCTCTGTAGCTGGGGCCTTTA	CATTCCTTGCACTGGCAAAA	-	-	-	-
	CL3579	AGGTATTTTCCAGACGGGTCAA	CCATCACAAGATTCGATTCCAC	-	-	-	-
	CL3640	ACTGGGCTCAATCTCCCAGTAA	TGCTGGGATTGTAGCTGTTGAT	+	+	+	RS2CL3640s
	CL3650	GAAAGGGGATGCTTTGTTGTTC	CGTGCACTTTCTTGCGTTACTA	+	+	-	-
84	CL3414	TCTCAAAAGAAGGCCCAGAATC	TGGGGTGATAAGTCTCGTTTCA	-	+	-	-
	CL3431	CGACACTAGTCGTTTTCGCACT	AAACACTAAGGCTGCTCGGTTC	+	+	+	RS2CL3431s
	CL3476	GTTGCAGACATGAACCCTGCTA	GGCCAAATTCGATTTCTGAGAG	+	+	-	-
	CL3532	ATTAGAAACCAAACGGGGAGGT	GATGTCCTCATGGGTGACAAGA	-	-	-	-
	CL3554	TGAGAGGAACTGGTTGAGGAGA	TACACAAGAGAGGGGACAACGA	+	+	-	-
	CL3605	GAAGATGGTGTATGCCAGCTCA	ACAGCTTATCCCAAGCCAGAGT	+	+	-	-
85	CL3455	CTCAAAGATCTTCGTCGTCGTC	TTGTGGTTGATCTACAGCAGCA	+	+	-	-
	CL3466	CGGTAACGTGGGATATGGAAAT	CATCAAGAAGTGCCTGACCAAC	+	+	+	RS2CL3466s
	CL3473	TCGCAGCTAATAGCCTCTCCTT	GGCCTTTGAAATGGTCGATAAG	+	+	-	-
	CL3599	GTGGAAACGCATGAGAAGAGTG	CCACCAACCAAAACCTTACAAC	+	+	-	-
	CL3609	CCTTTAACCCGAGGTGTGAATC	AAGTCGCGTTTGGTCTTCTACC	-	-	-	-
	CL3618	AGCTGAAAGAAACGGCAAAGAG	CGTCCAGCATCAAATTCAGAAG	-	-	-	-
86	CL3400	GAAGAACTTGCCGAGGGAACTA	GTTTCAGCAAACCAACGACAAC	+	+	-	-
	CL3416	TCTTAACCGACCGCCTCATATC	TTGTGGCTGTGTTGAAGCTGTA	+	+	+	RS2CL3416s
	CL3460	TTGGGGTCTTGATAGCAATCTC	TGACTGGTTGGAGAGAACATCC	+	+	-	-
	CL3520	AGGCTGCAGAACGCTTATCTCT	TTGAGTAAAAACCCCCACCAAC	-	-	-	-
	CL3600	AAAGATGCAAGTCACCCTCCAT	GTTTGTTGCTGGAGCTTCTGTG	+	+	-	-
	CL3628	AGGATGATGATGAGCAAGGTGA	AGAGCAAGGATCAAACCACCTC	-	-	-	-
87	CL3395	GGTTTCAGTTCCAAGGCAATTC	GTAACATAAAGCCGGGCCATAA	+	-	-	-
	CL3397	GACTGTGAAAGCGCAGATATGG	TGATAAGCCCCTTTCAGGAAGA	+	+	+	RS2CL3397s
	CL3415	CAAGAACCACAGGGAACAAGTG	CTCGATACAAAGGAGTCGCAAA	+	+	-	-
	CL3420	TTTGAGTACGCGTTCTTTGGAC	GCCGCTGTACATTGCTAACATC	-	+	-	-
	CL3433	TTAACCAGCTAACCGGTCCAAT	ACAGCAAAAGGTTATCGGTTCC	-	-	-	-
	CL3525	CCCGTTGCGTTTAGAGTTCAGT	AGTGGGAACAGGAGGAGAAGAA	+	-	-	-
88	CL3703	TTTCCCTCCATTTCAGGACCTA	TCTCGTTCTCACGTTCTCATGC	+	+	+	RS2CL3703s
	CL3794	AAGCTGAAACCGTCGGAGATAA	AACCTGCTGGTTCCATCTCAGT	-	+	-	-
	CL3834	AAGCTTGTGCAGAGAGGTGTTG	GCAACTCGTGAACGCTAATCTC	-	-	-	-
	CL3855	CAGATGAGACGTTCTTGCTCGT	TTACACAGTCCCCTACCGTCAA	+	+	-	-
	CL3879	ATAGAATGTCGTTGTGGGCAGA	ATTCGGGGAGGTTTCTTGAAGT	+	+	+	RS2CL3879s
	CL3951	GAACGGCCATTTTCCATCTTAC	TGGTACGCGTTTGGTAGAAGAA	-	+	-	-
89	CL3657	TATGGAGTTTCAACGGATGCAC	GTGGGTAACATTCACGTGCTTT	+	+	-	-
	CL3662	AATGGGTGGCTGCAGTTTTATC	AAGGCATCATCACCCCAATATC	-	+	-	-
	CL3709	ATCAAGACTTTTTCGGCCTCAC	GGAATGGATGGGAAACTAGTGG	+	+	-	-
	CL3763	CATTGCGGCTCTGATAGTGAAC	AGCATTCAAAGGGACAACATCC	+	+	-	-
	CL3771	ACGCTAAGAAGTTGGGAGGAGA	GAGCATTCTCTTGTTCGGTCAT	+	+	-	-
	CL3830	TCCGATCAGTGGTGAGTTTACG	AACAGAAGCGACGAGATCTTCC	+	+	+	RS2CL3830s
90	CL3677	TGATGTTATCAGCCAACCATCC	AACGTTGGAAACTTCCAGATCC	-	-	-	-
	CL3748	CCTTTCTTACCCTGCCTTTTGA	ACCCCTTTAGTTCTGGCAAACA	+	+	-	-
	CL3784	GGGATTCATTTGTCAGCACAAG	AAGCACGACGAAAACACACACT	+	+	-	-
	CL3789	TACTCAGCCTGGAGAATGCAAC	ATCACTCTAGCCATCGATTCCA	+	+	+	RS2CL3789s
	CL3804	CCCGAATGGAGAAAGAAAAGG	TTCATCGGATCACTGAAGCAAC	-	-	-	-
	CL3849	ACATCATGAGACCGACAACAGG	CTTGCCTCTAAACCCGAAAGAA	+	+	-	-
91	CL3666	TTTCATCTTCAGCAGCGTTAGC	TTTCGGAGAAGGGTTTGAGAAG	-	-	-	-
	CL3682	ATCCCCTTCCTTCATCTGAGTG	CACCAGGTACACGTCATCATCA	+	+	-	-
	CL3701	AGAATGCCTAGGGTCAGATTCG	GTTGGAAGGCAACAAAATGG	+	+	+	RS2CL3701s
	CL3768	GAAGCGACAACAGTTCCGATAA	TGCATGGGTTCTTTCTGATACC	+	+	+	RS2CL3768s
	CL3843	GAAAAGGCAGAGGCTGAGAAAA	TTTCAGGACTGGTCTGCAAAG	-	-	-	-
	CL3954	AGGATGTTGCAAGCGATCTGT	ACTGAAGAGAGTGGCAAGGTGA	-	-	-	-
92	CL3679	CACTTGATGGGCTCAAGAAGAA	AAGCCCAATGATGGAAGACAAG	+	+	+	RS2CL3679s
	CL3699	ATAAAGCTGACCAGATGGGAGA	GTACATGGAAAGCATGCAACAG	+	+	+	RS2CL3699s
	CL3726	GATTCATGCCTGCTGCAATATC	TGGTTAGGCTGGCCTCAATTAT	-	-	-	-
	CL3737	TGGAGATTGTTAGGTTCGCAGT	ACAAGCTTTGGGGAGAACAAGA	+	+	-	-
	CL3791	GGTTCGAGCAATTCAGTTGTGA	GAAACAACCCCTTTTGGGATTC	+	+	+	RS2CL3791s
	CL3950	AGCCTATGGAGCTCACCACATA	GCCATCGATAGCTTGATAGGAC	+	+	-	-
93	CL3743	AGGAAGACCGAACCGATACAAA	ATCCTGACGTGGAAACCAAACT	+	+	-	-
	CL3809	AGTCGCAAATCTTTGGACCTGT	TAACGGAAAGAGACCACCGAAT	-	-	-	-
	CL3845	AGAAGGCAGATGAAGTTGTTGC	GCAAGACAACAGTGGTGAGAAA	+	+	-	-
	CL3860	GGTCCCTCAGATTCCACAAACT	GCGCCGTGATTCACAATATAAG	+	+	+	RS2CL3860s
	CL3911	CATTTGAAGATCGTCGGACCTA	CATACCTGGAAAAGGCAAAACC	-	-	-	-

	CL3937	TTGAGAAAGAGAAGGCCAAAGG	CACTAGATTCCTGCCCACAACA	-	-	-	-
94	CL4065	ATCAATCCGAGTAGGACGTGAC	GGGGTCAAATAAAAGGTCAAGG	-	+	-	-
	CL4094	TCCATCATCCTCTACACCTCCA	TCACCTTAACTTTCGCCTCTGC	-	+	-	-
	CL4101	TCGTTGTCAGCAGACTTCACAA	AGCCTGCAGTAGGGTCAAATTC	+	+	-	-
	CL4113	CCACATATTTGGGCTAGCTTGA	CATTTACGCCACCAAAGGAAGT	+	+	-	-
	CL4128	ATGTGTACCCCTTGCGACAAT	ATCCTCTCCTCATTCACCCAAA	-	-	-	-
	CL4132	GGTTTCTTTTGCTGCCTTGTGT	TAAGAACTGCGAGTTCCGTGTC	+	+	-	-
95	CL3958	AGGGAGAAGCAACCAATTTGAG	GTTGCACACATGACACACAAAG	+	+	-	-
	CL4000	TCTCAAGATTATGGACCCGTGA	GAAGTCAATGAACGCGAATGAG	+	+	-	-
	CL4062	CTTGACATGGAACGTGATGGA	GAAAACCGAAAAAGACGCTCAG	+	+	-	-
	CL4063	AGGACAGAGACAATGACGATGG	GTCCGTACTGAAAACACGATGG	-	-	-	-
	CL4123	ATCCGGTTTACTTCCTCCTCCT	TGAGTCCCTCAAGACCTCAACA	-	-	-	-
	CL4152	GAAGCAACTACAAAGCGTGGTG	ACTAAACGACACCGAGACCGTA	+	+	+	RS2CL4123s
96	CL3978	CATCGTCAGGAAGAATTGTCCA	CCAGCGCTTGTGTTCTGTTCTA	-	-	-	-
	CL3986	AGAAAGCGTACAGCAGCAACAA	GCATAGCGAAGGTTTGTGTCTT	+	+	+	RS2CL3986s
	CL4055	GATTAACATGGCGGCTTGTCTT	CAAAGCCGAGATCAGTGAGAAG	-	-	-	-
	CL4100	ATAGGCCACAGGTGGTTTCAAT	AGGAAGGAGCTGAAAGCCAAGT	+	+	-	-
	CL4153	TAAACGGAGCGTCACGAGACTA	TTGCAACCTTACATGTGTGTGC	+	+	-	-
	CL4154	GCTGCAAACCCTCAGAACAAT	CAGCACTGCGTGTCATAAAGAA	-	-	-	-
97	CL3959	TTTCCTCTTCTCCGGTATGTCC	CGCAGATCGAAAAGGAGAGACT	+	+	-	-
	CL3972	CGCTATAGCTTGCGGTTACACA	TTTACACAACACGGCAAGAAGC	+	+	+	RS2CL3972s
	CL4002	CCCACTGAAACCCTAATCATCC	TGTAAACCTCCGAGCAGACGTA	+	+	-	-
	CL4072	TCTTTGACGCCTCAGTGATTTG	AGCTAGAAGACGGGACAACCTT	+	+	+	RS2CL4072s
	CL4116	CCACGAGTTTCCACCTCTCTTT	AACACCGTACTTGATGGCAATG	+	+	+	RS2CL4116s
	CL4151	GGTCATTCATCATCATCGTCGT	CAGCCATGGAAATGAGAGACAC	-	+	-	-
98	CL3989	CAAACAAGGGAAACACACCGTA	AGTTGTTCACGGGTTGCAAGT	-	-	-	-
	CL3998	CTAATGCGGGAATTGTGCTC	CAAACAAGGGAAACACACCGTA	+	+	-	-
	CL4007	TCCTGCAAACTCCCATACTCAA	ATGGCTGCTCTAAAAGGCAAAG	-	-	-	-
	CL4036	GGAATCAACAAGTGCGACGA	GACCGTTGCTTAAATCTGAAGG	+	+	-	-
	CL4091	GGCTTGCTCCATTCTCTCTCTA	AACACGATCGCTACGTCTCTGA	+	+	+	RS2CL4091s
	CL4102	GGTGGCTATGAAAACGAAGGAA	TGCGTCCTAGAACCAGAAAACC	-	-	-	-
99	CL4187	GAAGTTGTGGCTCTACCGCTTT	AAACAAAACGCACACACTCCAC	+	+	+	RS2CL4187s
	CL4210	ACACGTGGAGATGTGGATCAGA	GGCAAAATCAGCAGTTTCTCG	+	+	-	-
	CL4231	GGCTCGTGATCAACAGTCATCT	GAGCTTCTGTTGCTTCGGTTCT	+	+	+	RS2CL4231s
	CL4241	AAGGAAACCCATTCAGACGATG	CCAACCAAGCTGACCAGTCATA	+	+	-	-
	CL4255	TTGACATAAGATCCGCAGAAGG	CGAACATGCTCCACAGTCTTTC	-	-	-	-
	CL4309	CATCACAAGATTCCTCCCTCAA	GATACCATCTACCGTTCGCACA	+	+	+	RS2CL4309s
100	CL4240	GCAATCAAAACAGCAAGTGGAG	CCTCGTAATGCGTTTGAATCAC	-	-	-	-
	CL4287	ATGCTTTCAGGCTTTGAACGAC	GTGCTTCTTCTTCGGGATGTTC	-	-	-	-
	CL4290	CAGAGTCGCTAACCCTTTGACA	ACCCGAGAAAACTGCGTACTTC	+	+	+	RS2CL4290s
	CL4317	GGAGATTTGATCCTCCGAAATG	CCGAGGTACCAAATGTCGATCT	+	+	+	RS2CL4317s
	CL4381	GCAATTGTCTCAGAGCTGTTCC	AGGCGCGGATTATCATCTAGTC	+	+	-	-
	CL4417	AAACAGAGAGCTTAGGCGGAGA	AGTGGTCATGTCGCTGATGAGT	+	+	+	RS2CL4417s
101	CL4168	CGATGAACCAGTTGAAGACCAA	GCGTTTTCTCAAACACTCACCA	-	-	-	-
	CL4192	TGAGATTCGAGACAGCCAGAAC	GCCGTATGAAGATGAAGCATTG	+	+	-	-
	CL4204	AAAGGAGAAAATGACGGTGGAG	TCTCGTCGCTGAATATCTGAGG	+	+	-	-
	CL4232	AAATCAGGGGGTGCTCTAATCC	GGGTGCAAAAGAGCCAATGT	-	-	-	-
	CL4410	CGCCACATTCACTAAAACGGTA	TCGAGAAATCCATGGTCTGGTA	+	+	-	-
	CL4415	GTTATCAAACGTGACCCGCATA	CTTCAAGAGTAGGACCGAACCA	+	-	-	-
102	CL4228	AGAAAGGGATGGGATATTGCAG	AGACACAAACCAAAGCCCTTGT	-	-	-	-
	CL4244	AAACCTGCCCCTGATTACTTCC	CAAACGGGTCATGTGAAACTCT	-	-	-	-
	CL4254	GCCCATTTTCAATTCGAGGTAG	CAACGTAACGCAACAGATCCTC	+	-	-	-
	CL4292	TCACCTGTGAGTCAAACCCAAC	GTTTCTGCATGGGAAGCGTAAT	+	+	+	RS2CL4292s
	CL4357	TCAGACAGGAGCGAAGTTATCG	CACCAGCAAACTCTAGGCAACA	+	+	+	RS2CL4357s
	CL4362	GCTTATTGCTCTCCCGATCCTA	CCTTAACCTTCTCCTCCGTTGA	-	-	-	-
103	CL4251	TGCGTAAAGCAGGATACAATGG	GTTGCGTTTTCAGAGAATGGTG	+	+	-	-
	CL4294	ACGTGACAAGACACTCGACGTT	TAAAAGCCCTTACAGGCGAAAG	+	+	+	RS2CL4294s
	CL4343	TAGCATTCCTCGTGTTCTCAGC	CCAATCCATCTCTCCAGGTTTC	-	-	-	-
	CL4350	CAACTCCAGCTTTGCATTCTTG	TTGGCCGGTTCGATTTAGTAAG	-	-	-	-
	CL4366	AAGCCGAGAAACAGCAAAAGAC	TTGTGGATGGTTCGGTAGAATG	-	-	-	-
	CL4393	AATCGCCGTGAATAGGTATGTG	TGCAGGTGCATTTCCCATTA	-	-	-	-
104	CL4213	GATGAAGATCAAGAAGCCACCA	GCTGCTCTCAGCTGTTTCACTT	+	+	-	-
	CL4214	GAGCGGGAAAATCCAACATT	CACCGAAAAAGAGTTCAACCTG	+	+	-	-
	CL4313	CGCATAAGCGATCACGTAGAAG	ACCGATAAAGGATCTGGTCGAA	-	+	-	-
	CL4319	CCGACAAATCCGGAGATTACAT	TATGGCAATAGCACACATGACG	+	+	-	-
	CL4374	CGATTTGGAAGGAAACGTCTG	GATAAAGGGTGGGGTAGTTCCA	-	-	-	-
	CL4419	GTCCCAAAAGCTTTCGTCTTTG	AATCGTTACATACGCCACATGC	-	-	-	-
105	CL4457	TGTGTCCGGAGAGATCCAGTTA	TAGTTGTCTTCCCCATTGCTGA	-	-	-	-
	CL4491	AACCAGCCTCAGATGGTGTAAG	GGCAGAAATAACACTTGCGATG	-	+	-	-
	CL4540	CCAGCTATTGAACCGTTTGACA	CGGAAACCCCAGTACTATGCTA	+	+	-	-

	CL4559	AGATAGTGCAGCGATCACCAGA	TGCAACAGAGACATTGGTTACG	-	-	-	-
	CL4698	TCTTCTAATTCCCGGCGATATG	CACCAAAGAGAAACAGGGAAGC	-	-	-	-
	CL4716	ACCAAGGAAAGAGTGGAAGTCG	CACGCCAAGCTTTCTTCTTCTT	-	-	-	-
106	CL4514	TCCTCAGGCTCAACATTCTCTG	CAGAACCACAAACCCTTCTCAA	+	+	-	-
	CL4519	GAGAGGCATCCGTTTGGACTAT	CCCTCGAATTCCATTCATCAGT	-	-	-	-
	CL4632	GATCATGTCCTGGCATATTGGA	GGATCAGTCATTCCACGAACAA	+	+	+	RS2CL4632s
	CL4654	TCATCGGATCTCAGTTCATGCT	GCCTTCCTCTTCTTTTGCATGT	-	-	-	-
	CL4660	TGCAGTGGAGTTGGCTATTTTG	CGAATCTGCACACACATCACTC	-	-	-	-
	CL4686	CTTCAGCCACCTCTCATTCCTT	AGCCATCCCTAGGCTAAAACAA	+	+	-	-
107	CL4468	GCTCGGTTTCTTCTTTCTCTCG	ATTCGATGAACCTCCCTTGTGT	-	-	-	-
	CL4511	TGTAGCGGATTTCCGTTGTGTA	ACCAATCATGATGAGCCCTAGC	-	-	-	-
	CL4518	TCTTTCGCATTTCTCTCTCTCC	GCTAAAGTGACGTGAGCACAGA	-	-	-	-
	CL4552	GAAGCAACTACAAAGCGTGGTG	CGAAGCAGAACCAAATGACAAC	-	+	-	-
	CL4662	GGAACTGTTGGAGGAGCTTTTG	TGAGCACACACACTGCATAGGA	-	-	-	-
108	CL4431	TGTTGAATATGATCCGGTCAGC	GGATGCAGTTTTTGTTCCTTCC	-	-	-	-
	CL4452	CACACAGTGATCAACCAACCAC	GTTGGAACGACTCCTCTCGATT	-	-	-	-
	CL4466	TGAGAGGTCTGCAAAAGACCAA	TCCCTCGTGACCACAAGTTAGA	-	-	-	-
	CL4546	CTGATGAGAATGCGAAAGAAGC	ATAACTCAATTGGGACCGAACC	-	-	-	-
	CL4669	GTGATGGATCAGCTCAAGGTGT	CGTACAAGACCCCCATTTATTG	-	-	-	-
	CL4706	GCAGAGAATGCAAAGCCACTAA	CGAGCAAAAGTTTCTCCAAAGC	+	+	+	RS2CL4706s
109	CL4512	AGAAAACAGCTCTCCCTGATGG	CGGTTTCTTTTTCCTCTTGTCC	-	-	-	-
	CL4550	CACATCCATAGCTCTCGAAGGA	TGTTCTCCACCGTCTACCTTTG	+	+	+	RS2CL4550s
	CL4568	GCCACTCAAATTAACGTCTTCC	CATCATCATCATCCATCGACTC	-	-	-	-
	CL4589	GCATGAAGAGAGATTCGGGTTT	GCGGTCAAGTTCCACTTTTCTT	-	-	-	-
	CL4648	ACTCTCGTTCACCACCACGTAA	CGCATACGCAACATCATTGTC	+	-	-	-
	CL4649	TTCTTTGCAGACAGACAGCTTG	CCTGAAGGGTTTACTCTGTTGG	+	+	+	RS2CL4649s
110	CL4525	GCCACTAACAAATCCAGACACG	AGCCCTAAAAGCAGAGAGAGCA	-	-	-	-
	CL4536	ATGAACAGGGAGGCTAGGGTTT	TGGCAGTGAGAGAGACGGTAAA	+	+	-	-
	CL4594	ACCACCTCAACCAGATGAAACA	GCTCAAGTCTCAACCTCAGCAA	-	-	-	-
	CL4647	ATCCAGAGGACACAGCAAGTCA	TGGCTAGAACATGGAGAAACGA	+	+	+	RS2CL4647s
	CL4685	ATGGGAGGATTCCCTCACTATG	CCAACGTCAAAATTGCCAAC	-	-	-	-
	CL4714	GCAGAAGAAGAAGTGTGGAGCA	TAACGAAACGAGAACCAGTCCA	-	-	-	-
111	CL4795	CCACTTGCTTTGTCTGTCCACT	TGTTACCCTCCTGCTCCTTGAT	+	-	-	-
	CL4804	CGCTAGAAGGTTGATCTTGTCC	CATGGAAACATCTGGAGATTGG	-	-	-	-
	CL4808	TGTTACTCCGTGCTCTCGTTCT	AGATACTTGGCCCAACTGAACC	-	-	-	-
	CL4850	GTTTCCCTCTCTGCGTTTCAAT	GAAGGTTATGAAGGTCGCTTGG	-	-	-	-
	CL4886	CGTTCATCTCTCCATGAACTGC	CATTTCGTTTCTGAACGTCGTC	-	-	-	-
	CL4906	CGTCTGAAACCAAAGACACTGG	GGACTGGTTACAGTTTCGTGGA	+	+	+	RS2CL4906s
112	CL4744	GCTATATCATGGCATGGGTTCA	GTGAGCAACAAGAGTTCGCCTA	-	-	-	-
	CL4824	TTGTCTTGACGGTGGGAGTCTA	CACAACATAATGGCACGCAGTA	-	-	-	-
	CL4827	AAGACAAGGCAAAGAGGCAAAG	CTGGGCATCCGAGTAAACAAAT	-	-	-	-
	CL4831	CGAGAAAGCAGCAGAAGTAGCA	GCTTTCGGAGGACATTGGAATA	-	-	-	-
	CL4834	TCCTGGAAACAGAGGAATGTGA	CATCAGGCAGATTTTTGGACAG	-	+	-	-
	CL4860	CTTGAAAGGTGGAGAAGGAGGA	AAACGAAACCGGAGAATCTGAC	-	-	-	-
113	CL4740	GAAGGAGACCAACCCATCACAT	GAGGAGGACAACTCAAGCACAA	-	-	-	-
	CL4817	CTCGTCTGCGCTTATGGTTATG	ACTAACGTTTGCCCCTGTCAAT	+	+	+	RS2CL4817s
	CL4840	TCCTGGGAGGATGACAGCTATT	CTATGAACTGCTGGTGGTTTGC	+	+	-	-
	CL4858	ACGACAATGGATCACCCAGTTA	CGGTAAAGAGCGGAGCTAACAT	-	-	-	-
	CL4902	AAATGCGAAGAAGCTCGAAGAG	GCATGATCACAACACTCACACC	-	-	-	-
	CL4932	GGTCATAAAAGCTTCCCGAGTG	ATAGCATCCACACACCATCGAC	+	+	-	-
114	CL4724	GAAGGTTCCAAGTCCGTGAAAT	CAAAGGTCTCCAGGATCTCCAA	+	+	-	-
	CL4736	GAGCAATCTCAAGTACCCAGCA	GAAATGAGAACGCTCGGCTAAT	-	-	-	-
	CL4781	AGAAACCCATCGAAGGAAGACA	TGAGTCACCAAGACCCTAGCAA	-	-	-	-
	CL4816	GCTGTTTCAGTGCTCCCACATA	GAACCGAATCCCAATTGAACC	-	-	-	-
	CL4863	AAAAGCTTCCCTTGCTGATGTG	CACGAACAACAAACTCCCATTG	-	-	-	-
	CL4903	ACCCAACTTTCTCAAGCAGACC	TGAACTGACTGCATATCGGAAC	+	+	+	RS2CL4903s
115	CL4762	GTGTGGAATCCATGGGAAAAG	GTCTCTTGTCTTGATCCGCAAA	+	+	-	-
	CL4800	GATCCATTGGCTGGTTCTCTTC	GGTTTCCTCATCAATCCAAAGC	-	-	-	-
	CL4823	ACTCATTGAAGGCGACTGAGAA	GCAGGCATCACTTTACAGCTTC	-	-	-	-
	CL4843	CCCATTTGGGATGGCTACTTAC	GGTGCTGAGGCACAAAAAGTTA	+	+	-	-
	CL4854	GTGAAGAAGGACGAGAACGTGA	ACAACCCCTAAAGACCGAAACA	-	-	-	-
	CL4875	AGCTTCAATAGGGTTGCTCTGC	CGCAACTCTTTTTGCAGTCTTG	-	-	-	-
116	CL5001	TTCCAGTGTGGTGGATATGGTT	GTTTGTCAAGTGGGTGATGATG	-	-	-	-
	CL5036	TTCAACAAGGCAGTATCCGAGA	CGTAACCCAACCGAAAAGAATC	-	-	-	-
	CL5046	ATGAACAGCGGAGCAGATATGA	ACACAAGGATCTGGAGCTGACA	-	-	-	-
	CL5116	TTGCTACGTTCAATGGCTTCTG	GGAATCCAAAGCTGTGAAATGG	+	+	+	RS2CL5116s
	CL5133	AGAGAGAGATGGGTGGAGGAAA	TTGCAGGATAGCCGACTCAATA	-	-	-	-
	CL5184	AATAACCCCAGAAAACCCCAAC	ATCCGACTCCTCGTTCTCGTAA	-	-	-	-
117	CL4953	CACTCATTCTCCGGCTACCTTT	CAACAAGAAGTCGAGACGTTGG	-	-	-	-
	CL5007	GTGTGTCGGCTGTGGAAATAAA	ATCCTGCAATTAGGTTCGTGGT	+	+	-	-

	CL5012	ACGCAGTGGGACATGCTTTT	GCTCAATAGCCTCGGATGATAA	+	+	-	-
	CL5078	ATCATTTCCCAGTCCACCAAGT	GCTTCTTCAGGTGAACCCAAGT	+	+	+	RS2CL5078s
	CL5098	GTATCCTTTCGGTATGCCTTGC	GCACACCGCAGAAAGATTCTAA	+	+	-	-
	CL5121	AAGTTCCCTTCGGTTTTTAGCC	AACCGTCCTCATCGTTAAGCTC	+	+	+	RS2CL5121s
118	CL5026	AGTGTGATTCCGCAGTGGAGTA	ACTCTTGCGTTCTAGGCCAATC	-	-	-	-
	CL5047	GGTGTACATGGGCCTGTTGTTA	TGACTCACATACTGCCGGTTCT	-	-	-	-
	CL5049	CCTCAGCAAAGGAGAGAAATCA	CTCGGAAAAAGATGGTGAGGAC	-	-	-	-
	CL5105	GATCCCGACAAGAAACCTTCAG	GTGCCATAGAGATCCCCCAATA	-	-	-	-
	CL5201	AGTAAGACACCGAGCGAGGAAA	CTTCCTAGAGACGATGCCGAAT	-	-	-	-
	CL5216	CCGTATTATGGGCGTTACGTTT	GCAGCGCACTTGAACTCTATCT	-	-	-	-
119	CL4951	ACCAGAGTCATTTGGGTCGTTT	AGCTTGGCTGCTTGAATCTTTC	+	+	-	-
	CL5063	TGGTTGTGGAGAAGGATCAAGA	CGCAAACATAAACTCACGATGC	-	-	-	-
	CL5077	GGTGTGTTTACAGGACCATGTG	CCTCCTTGATGAGAGGATTGAA	+	+	-	-
	CL5134	CTCCCATGGCTAAGAGGTTTTG	GTTGGAAATGTGGAGAATGCTG	-	-	-	-
	CL5154	GATGATGTTCTTTCGCCCTTTC	AATCCCACACATGCATCATCTC	+	+	-	-
	CL5178	ATCCATCAAACCAGGAATGGTC	CATAAGCCTTCCCATCTCCATC	+	+	-	-
120	CL5223	CTACCAAGGTTTGGGAACGAGT	ACGAAGCTGGCTGTTACGGTAT	+	+	+	RS2CL5223s
	CL5400	CGTTGATGCTGACAAAGACAAC	GTCCATTTTGGTTGGTTGGTG	-	-	-	-
	CL5411	GGGCAGAACTGGTGTTCTGTAA	CAACAAACACAAGGTTGGAAGC	+	+	-	-
	CL5429	TTCCTGATGAATCCGGTAGAGG	AGACCATCTAACTTCCCGCTTG	+	+	+	RS2CL5429s
	CL5461	GTGGATCTGCATGGACTATTGG	AGACGCGAGTAGAGATTGTTGC	-	-	-	-
	CL5583	TTCACCCTGAGACCATAGAAGG	TTGCCTTGTCCATCCTCAAAG	-	-	-	-
121	CL5244	CCAACCTGAAACGGTGTGATTA	TCTGCTGTGCGTACTGTCAAAA	-	-	-	-
	CL5284	AGACGTGTGACCAGGCAGATTA	ACTAGACATGAAAGCCCGAACG	-	-	-	-
	CL5302	CAACCATGTGAGAGGCGTTCTA	CACGCAAGCTAACTACAAGCAA	+	+	+	RS2CL5302s
	CL5384	CTACTGCGACGAAAACAACAGG	ACTAAGACCCAACCCAAGAGCA	+	+	+	RS2CL5384s
	CL5453	GGATGGGGAAGTGAACGAAGTA	ACAAACTCCTAACGGCCCAGTA	+	+	-	-
	CL5650	TCAAAGAACACAAGGAGCTGGA	CAATGCTTCCACACCTCTGAAC	-	-	-	-
122	CL5274	GGTTTCATCTTCGGAGTGCTTC	CACGCAAGCAACTAAAACACAC	-	-	-	-
	CL5440	CCTGAACCCAAGCAAGAAAGAG	GTTGATGCGATCTTGGTTTACG	-	-	-	-
	CL5447	ATCAAGCGGCTATTTCTCATCC	AGATTTGGCACATGCCAGTAGA	+	+	-	-
	CL5459	AGGACTACATCAAGAGGCAGCA	CGTCTTGGTGCTTTGTGCTT	-	-	-	-
	CL5480	CCGTTCAGATCAATCGTCTCAG	GGAACACGAGCAAATCCAGATA	-	-	-	-
	CL5524	AGCAAGCAAACCTCCCAAACTA	GAAGCCTTCCCACAAAACAGAG	+	-	-	-
123	CL5271	GAAAATCTCGCGAACATCCTCT	ATTCTTTCGAGATCGACGAAGC	-	-	-	-
	CL5357	GCGACTGCCTGAAAATCAATCT	TGCTTTCTCTAGCTGCGGTTAC	+	+	-	-
	CL5450	TAATCCAAGTGGCTCTGCTCTG	CAAACCCAATCTGAATCCTCCT	+	+	+	RS2CL5450s
	CL5488	GTCCATGGGTAGGATGCAAAAT	CAGCCTTTCTAACCAACGACAA	+	+	-	-
	CL5545	TACGCGGTTCAAGTGATGAACT	TGCTCTGCTCCTTTGTCTTCAC	-	-	-	-
	CL5551	TTATTGGGACTTGGTGAGCTTG	GATTCACTGAAAACCGGGAAAC	+	+	-	-
124	CL5312	CTACGAGCCAACCGATGAAAAT	CGTATCCCAGAAACACCAAAAG	+	+	+	RS2CL5312s
	CL5347	GGATGTCAAAATGCATCGGTAG	GGGCATTTTCTCTACCCCTTCT	+	+	-	-
	CL5471	CATCAGGAGAAGGACGGAAGAT	AGCTGCTCGAGTAGACGTTCAA	+	+	-	-
	CL5484	ACAGAGGATCCATACAGCACCA	TCTCCACCGTACGTGTTGATTT	+	+	+	RS2CL5484s
	CL5486	CCCCACACCCAAATTTATACCA	AAGACGAGGAGGAAGCAGAAGA	+	+	+	RS2CL5486s
	CL5586	TGCAACACCATCAAAGAGGAAG	CATCTAAACCCGCTTTGCTTTC	+	+	-	-
125	CL5239	TCATATTCGGCGGCACTAGTTA	AAGCGTCAAGTTTTCCCACAGT	+	+	-	-
	CL5294	TCATCTAAAATCCACCCCTCGT	TTGTTGCGTCTGCTCCTTATGT	-	-	-	-
	CL5329	ATTCTTGGAGGGCAGCTTTACC	GCAAAGCGAGAAGAAAAACTGG	-	-	-	-
	CL5439	AGGCTTTTGCAGCCAGAGATAC	AACCACCCCCACAAGTATATGG	-	-	-	-
	CL5563	TGGTCACCAACAAAGTCTCAGG	CACAACACACGAGATTCTGCAA	+	+	-	-
	CL5658	GAATTGGGACACACGATGCTT	ATAGTTAACTCGCGACCCTTGG	+	-	-	-
126	CL5707	GCCGTTGCTGTAAATAACTTGG	GGGAAAAAGCCAACCAACACT	-	-	-	-
	CL5729	ACAGAGACACGAAACCCAATGA	GAGAAACGAGGAAGGCAGTCTA	+	+	-	-
	CL5824	TGTGAAAGCACACTCTTTGGTG	CTGATGTTACACAAGCCGCAAT	+	-	-	-
	CL5825	TTTTGTCAGCAGGTGTGTGATG	ATGTCCTGAGCTGCCATTCTTT	+	+	+	RS2CL5825s
	CL5877	CCACTTTGTGGTTGCGAGTTAT	TTTCTACCTGTTCCCTGGAGGT	+	+	-	-
	CL5950	CGCCTAAAATCCTCCTCCTTCT	GATCTCATCCCTTGCGCTACTT	+	+	-	-
127	CL5739	CCTAAAATCGTGGCCGTAACAT	TCACATCTCCTCATCACCATCC	+	+	-	-
	CL5762	GATTTCGCGCTACAACAAAGTG	AAGCACTTGTGGCTTCTTGTGT	-	-	-	-
	CL5798	GAAATCCACGTCGAAAAGGAAC	CATAAACACACACACCCCCAAT	-	-	-	-
	CL5802	AAGAGCAAGACTCACCAAGACG	GCTTTCACCAACATTGTTCACG	-	-	-	-
	CL5896	GATCGTTCGGTTTCTTCTCTCG	GCCTCACACAAACAGAGCAAAC	-	-	-	-
	CL5958	CATAACAAGCCAGTGCAAAGGT	ATTGCAGACATCTGGCTTTCCT	+	+	+	RS2CL5958s
128	CL5758	GGTGAAATCACAGCTGCAAGAC	GGGAACCATGTACACACCTCAA	+	-	-	-
	CL5781	TGATGAAGAAACTAGCGGTGGA	ATAACAAAGAACCGTCCCAAGG	-	-	-	-
	CL5792	TGTATCCGCGAATGACTAATCG	TTAGAGAAACCGGAAGGAACCA	+	+	-	-
	CL5828	CCAAACTGAGCAAGAACTTGGA	CCAAATCCCAAAGGGTAAAAGG	-	-	-	-
	CL5867	ATGGCGACTTTCTAGGGTTTCA	CAAACACTCGCACCACAAATCT	+	+	-	-
	CL5891	GCCATCATTGGTAAAGGAGGAG	TGGTCTTATGCCCAGCAAACTA	+	+	-	-

129	CL5719	GCAGTCAATGAAGGAACCTTTG	TGAGTGACTGCTCATCGGAACT	-	-	-	-
	CL5775	AGAGGAATACCTTGGTGGAAGC	GATGAAAAAGGGGAGGCAAAGT	-	-	-	-
	CL5785	AGATTGTGATGTGGGCTGAGAA	TCTCGTTTAGCAACTCCACTGC	+	+	+	RS2CL5785s
	CL5794	AAACGTCGGGAAAGACTCGATA	GTACAAAACCAGACACCGTGGA	-	-	-	-
	CL5832	GTTTCTGCGAATCCTGCTCTCT	GTGCACCTATCAAACCACCTGA	+	+	+	RS2CL5832s
	CL5841	AGCAGCTCAAAGGGGTGTTTAG	TACGACGCTCTAAGGCTGCTAT	+	+	+	RS2CL5841s
130	CL5672	AGATGGATATGGGGATCAATCG	CCCCAAACATAATAAGCCAAGC	+	+	-	-
	CL5831	TCCAAACCCATCAAGACAAGTG	AACGTACCCGAAACTGTGCTTT	-	-	-	-
	CL5857	GCGAAATCCGTCTACACCAAGT	ACCAGAACCATCGCTAACCAAG	+	+	+	RS2CL5857s
	CL5860	GCGTGTGGTGCATCAAGATACT	TGTCTCCACAAAGCTCCCTTTT	+	+	-	-
	CL5895	GATCATGAGGATGATGGCAAGA	TGGGGGAAGAGTATCTCAAAGA	+	+	+	RS2CL5895s
	CL5949	TGGAGAAACCGAAGAAGAGGAC	AGGTGAAATGCGAAGGTGAATC	+	+	+	RS2CL5949s
131	CL5727	CTCCGCTGCTCGTGAATATCTA	ACCATTTACCGCATAACCCTTG	-	-	-	-
	CL5733	CAAGAGAACCGAGCCAAGAAAG	GACAAATGGGTACAAAGGCACA	-	-	-	-
	CL5811	TGATCAGAAGAGAGCGTCATGG	GAGGAGGTCTTCAACAAACAGG	+	+	+	RS2CL5811s
	CL5868	GGGCCAGGGTTAATGTTATTTG	GTGGTCCGATGATCGATTGTAA	-	-	-	-
	CL5890	TTGTGGTGACTTTTGCTCTGGT	AGCTTTGATCGTCCTTCTCCAC	+	+	+	RS2CL5890s
	CL5948	TTGGTCGTGGCATAGAAACAAC	AGCAGTTGGGAAAAACAGGCTA	+	+	-	-
132	CL6045	ATTCTTCTCCTGCAGAGGGTGT	TCTTCAGTCGCTTTCGTAGTCG	+	+	-	-
	CL6129	GATTGGCAGTTGTGGCTAATGT	CGATGATGAGAAGGGTAACGAG	+	+	-	-
	CL6131	GCTCATAAATCGGCATGTAGCA	AGGCGTATTGAGGCGGATATAA	+	+	-	-
	CL6133	CGAACTGAATCAGCATCAAAGG	GGAGCCCTTTTACCTCATCAAA	-	-	-	-
	CL6134	AGCTCGAACCCGAAGATGTTTA	GAAAGGCGAATGGAAATACAGG	-	-	-	-
	CL6139	TACAGCATTGGGGTTGTGTTCT	AGCAAACCAAAAGAAGCTCAGG	+	+	+	RS2CL6139s
133	CL5990	ACCCCGAAACGTTTAGACCAT	ATCCACAAATCTTCAGGGCTTC	+	+	-	-
	CL6037	TGGGTTCTGAAACTTCTTGCTG	ACCAAAACCTGAGCGAGATTGT	+	+	-	-
	CL6070	CTCACTTTGGTTCCTTCGACCT	TTCACCTTGAGCTCTCTCCTCA	+	+	+	RS2CL6070s
	CL6124	CTGGCCACTTGGATTTCTTCTT	GCCCGAGTGTCAATATTTTTGC	+	+	-	-
	CL6192	CTTAGGCCATTTGGGATTGATG	GCTTGTTTCATGGTTAGGCAAC	-	-	-	-
	CL6221	ACCCTAATTTCATCCCCCATTC	CACCCACTGACCCAAAACATTA	+	+	+	RS2CL6221s
134	CL6004	AAGAGACAAGCCCACGAATCAT	GTCCTAAAGACCCATCGCAATC	+	-	-	-
	CL6043	CTGGTCGGAGATCAACACGATA	TCTCTCTCCCATCTCGTCAGAA	-	-	-	-
	CL6048	GCAGCGTCCTAAGAACGATACA	TGATGCACACAAATCGAACC	-	-	-	-
	CL6084	GGTTGCTAGGCACTATTCGACA	CTGAATGGCAAGAAGACCACAA	-	-	-	-
	CL6088	ACAGAAGAGAATGCCAGCAAGA	ACAGACGCGACACTACACCAAT	+	+	-	-
	CL6242	CTGTGAAGCCCAAGGCTAAAGT	GGGGTTTCAAGAAGAAACCAGA	-	-	-	-
135	CL5963	CCGCATGTGAAGAAATTAGCTG	CCAATGAGAAAAAGGAGCCAAC	-	-	-	-
	CL6109	AGGGAAAGAAGTTGCAGAGGAA	GTATGTTTGAGAAGCGCCCAAT	+	+	-	-
	CL6217	ATGGAGGAAGATGGAGTGGATG	CCAGAAGCAAGAGAGAATCCAA	+	+	+	RS2CL6217s
	CL6227	GCGTTGCCTGTTTATCAGAGTG	TCAAACCACCACCAGACTCTTC	+	+	-	-
	CL6244	CTATGTCGATAATGCCGGTGAA	TGTGATCTTAACGGCGATGGT	+	+	-	-
	CL6255	GTACGCTGAAGTGATGCAAAGC	CCAAATACGCAGGTTTCCTTCT	+	+	-	-
136	CL5993	ATGGACATCTCATGGCTGGTAA	GCCTTATGAGCGACCAAAGAGT	+	+	-	-
	CL6066	AGCTGAACATGTGAGGAACTCG	TGAATCTACCGGAGTCCTTTGG	-	-	-	-
	CL6123	AGATAAGCGCGTTCTGGTTGAT	AGCAAGTGGGAAGAAAGATCCA	+	+	+	RS2CL6123s
	CL6191	TAGGATTGGCTGGTCAACAAGA	ACCATGGTTGGTTTGTCAACTG	+	+	-	-
	CL6220	GCAAGGGGATAGCAAAGAGACT	TTTAAGACCACAAGCGCCACTA	+	+	-	-
	CL6226	GAACAAGGGCTCTTCCACTGAT	CTTCGCATCATGCCCTTTTT	-	-	-	-
137	CL6072	AGAGGCGTTACGACTGCTCAAT	GCCAATCAACAGAACTTTGAGG	+	+	-	-
	CL6075	CACCATCGGACAAAGACACAAG	CTGCAATGAACCTTGCAAACTC	+	+	+	RS2CL7075s
	CL6110	CGAGAAAGGCTATGAGTGCAAC	TTCAGCAACGTACAACACATGC	-	-	-	-
	CL6167	CTGCGCCATTCAAATACAGACT	GCGATCAGACCAAGCATCAATA	-	-	-	-
	CL6248	GGAGGAGCCAAGACAATAATGG	AGTTGCGTGGAACAAGACAAGA	+	+	+	RS2CL6248s
	CL6259	ACAAATACCGACATGACCATCG	CCCCTTATATTTGGCCATTGAC	-	-	-	-
138	CL6294	TTGCAAACGCTCTTCTCTATCG	ACCCTCGTGCTAGTCACATGAA	+	+	+	RS2CL6294s
	CL6304	GGTGTTTGGTTGGTCTGTCATC	TCTCCAGATATAACCGCAGCAA	+	+	+	RS2CL6304s
	CL6368	TTTCTCGTTACGTTCCTGTTGG	TCGACTGCCCACAACTTAGAAA	+	+	+	RS2CL6368s
	CL6379	TACTCTCAAAGCGGCATACCTG	AAAGAGGGCCATCCATCATAAG	-	-	-	-
	CL6408	TTTGACCGATGTCTTCTCAGGA	AAAGCATCCCAGTGAAGGAGAG	+	+	-	-
	CL6485	AGAGGCGATTACCTCTTGTTCG	AAAAGCTGTCATCCCCACACTT	-	+	-	-
139	CL6377	AATGGAGTTTCCGTGGAGAGAA	AGAGCCCGAAAATAAAGGGAGA	-	-	-	-
	CL6418	GCTTCAGCTGGAGTATCAGGAA	CAGAATCACACACGGTGAGAAA	+	+	+	RS2CL6418s
	CL6451	GGTATCATGTCCGTTCACGAAG	TCCTTTGCAGTCAAGGGACTTT	-	-	-	-
	CL6479	TGTTTCACCTCAAAACGACACC	CAAAAAGGACCCACACAAGAAG	+	+	+	RS2CL6479s
	CL6571	GGGGAGAGGTCTGATTTTGAGA	TCATTTCCTACCCAACCTTGCT	-	-	-	-
	CL6688	CCTGGTCTCATCACATTTTGGA	CGGTCGTGACTATCCAAACAAA	+	+	-	-
140	CL6391	AGGATGACATTCCTGCTTCCTC	AATAACGGAGAGACCCGCAATA	+	+	+	RS2CL6391s
	CL6532	CGGAGGTAAAGAACCTTGTCGT	GATTTTCCAACTGCCGAAGAC	+	+	-	-
	CL6665	GAGTGCCCTGTTTCTTGTGATG	CGTAGAAACCCAACAGGAAAGG	+	+	+	RS2CL6665s
	CL6676	GTGATCAACGCTGTGATGGAAG	AGAAGGACAAGCGATTTCTGTG	-	-	-	-

	CL6683	GAAGAAAGTCGAAATGCGTGTG	GATTCCACGCAAACTCTCAATG	-	-	-	-
	CL6689	TGATTTTCTAGCGGCCTGGTAT	CGGAGAATATGGGGATCTGAAA	+	+	+	RS2CL6689s
141	CL6276	CATTTACTCGTACGGGATGTGC	CCATGTTTGAAACCAGCTACGA	+	+	+	RS2CL6276s
	CL6278	ATATCCTTCCACGCTTGCTCTC	TGACGCTATCACCACGAGAGAT	-	-	-	-
	CL6322	CAACACATCAGCAACTGAACGA	TAGCCCCAAACAATCTGAACTC	+	+	-	-
	CL6354	CAGAAGCCTTTCTTCCCACCTA	TTACAATGCCTTAACCGCTGTG	+	+	-	-
	CL6586	GTCAACAGAGGAGTTCGTCCAA	TCTTCTGCCACAATAGGAACCA	+	+	-	-
	CL6623	ACCTTCGCCAAATCTCTCTGAC	GGCGAGGTTTACGAGAAACTGT	-	-	-	-
142	CL6352	TCTACACGTCGGGTGCATATCT	TCAGAGCTCCAAGAATCCAAGA	+	+	+	RS2CL6352s
	CL6369	ATCAGCTGCGACATCTCTTCCT	TGTTGCAGCTTGAGTAACAGCA	+	+	+	RS2CL6369s
	CL6399	ACAGAGGCAGAGCTTCAAGACA	GGTTATCCTCACTTTGCCATCA	+	+	-	-
	CL6424	ACGGTGATATCCCTGACGAAGT	AGCTTCGACACCACCCTAATCT	+	+	-	-
	CL6464	GCATTTAGCGACAAAAGCAGAG	GCCTGAAGCACGTTTTGACTTT	+	+	-	-
	CL6598	GGTCACTTTCAATGGGTTGGAT	ACGAGCCACTCCTTTTGATCTC	-	-	-	-
143	CL6287	TGTGGTTCAGATCGGTCTGTTT	CCTCCAGTGACATTGGTTCAAG	+	+	-	-
	CL6355	CACCGGACACTTTTTACTGCAA	ATCACTCGCTATCAGCCCAATA	-	-	-	-
	CL6465	GAGTTGGAGACTTCGTTCATGC	GGCCAGTAATTCAGCACTCACA	+	-	-	-
	CL6511	TACCAAGGCCACAAAGAAGACA	CCACACACCATCGTGACTGTAA	+	+	+	RS2CL6511s
	CL6716	GTCCGCATACCCAAATCAACTT	CCTGGGAATGTTACCAAGCAAT	+	+	+	RS2CL6716s
	CL6727	AGTTCAAGGATGTGCACGGATA	AAACGTGGATACACACCATTCC	+	+	-	-
144	CL6444	CAATAGTCAACGACTCGACGTG	GAATACGAAACAGAGGGGGAGA	+	+	+	RS2CL6444s
	CL6478	TGACTTCACTGGAGTGGGTGTT	CTCGCCTGTCAGTGTCATCATT	-	-	-	-
	CL6509	AAGATGAAGGGCCCCAAAGTAT	GGCTAAGCGCAGTTCTTTGAAT	+	+	-	-
	CL6536	ACGTGAGTTCTTGCGAGTACGA	GGGAAACATGCCATGGAAATAC	+	+	-	-
	CL6615	GTATTGAGCAGCGGTGTAGCAT	TCCCTGAGAACCGTACTTGAGA	+	+	-	-
	CL6666	CTGGAGAATGGTCAGGATGATG	AATGAGACAACTCCACCAACGA	-	-	-	-
145	CL6796	CAAAGAACACTGAGAGCGTGGA	GTCCCCCATTTGTAAAGGGTTT	+	+	-	-
	CL6883	CAACTGTGGAAGGTGTTTTTGC	GGCTGAACAACCAGGAACTATG	-	-	-	-
	CL6968	AGCGAGCTTTTTCAGCTCAAGT	CATAACAACCATCCCCCAATTC	+	+	-	-
	CL7018	GACGAGAAGTTTGAACGGGTTT	GAGATTGGGTTTCTTCGGTTTG	-	-	-	-
	CL7111	GATGTGTATGGGTTTGGTGTGG	CACCACATTCACAAGGCATTTC	-	-	-	-
	CL7121	TTGGTGGTGTCAGAAGACGAGT	GACCGGTTCGGTCATTGTAGTT	-	+	-	-
146	CL6772	GGAAAGTGATAGTGGCGGAATC	GTGTCCTCACAAGCCAGAGAAA	-	-	-	-
	CL6800	GGAGAATCCCATTCCATCAAGA	CCATTGAGCTTGGCGTATACAA	+	+	+	RS2CL6772s
	CL6911	CTTATCTGGAACGCGGTTGTCT	CCTTCCCCCACACTCTAACAAA	+	+	+	RS2CL6911s
	CL7009	CATCTCAAACCCCTCAAAGACC	TCAATGTCTCCACCTCATTGCT	-	-	-	-
	CL7081	CTGGACAGCATCAACAAACCAC	CGGTGAAAACAAAGGGCTTAAC	+	+	+	RS2CL7081s
	CL7137	AGCATCCAAAGAACGACACAAG	CAATCAAGGGCCCATAGCATA	+	-	-	-
147	CL6806	CAAGGAATGCGATAGGAGGTGT	TCATAGGCCACTCTCCGTTTTT	-	-	-	-
	CL6809	GGTTGTTTACGACGAGACGACA	CCCTGGCGAGTTTATTTGAATC	-	-	-	-
	CL6938	GCATGCACCCTTTCGATTAGAT	TTGCGTGTCAATAAGGAGCTGT	+	+	-	-
	CL6991	TTGATGGAGATTGTGGATACGG	CTCTGTAACCAGTTTGGCAGCA	+	+	+	RS2CL6911s
	CL7077	AGTGATTGGCGTGGTAATCTCA	TTGAGCATCTTCAGATCCGGTA	-	-	-	-
	CL7168	GACAAGATGGGTGATGCAGAGT	TGGTTCTGATCTCTTGTTGTGC	-	-	-	-
148	CL6791	CAGTGTTTGAGCAAGGAGCATC	TTCTTCTCGGGAGAGCAAAATC	+	+	+	RS2CL6791s
	CL6815	CAAAGGAGACTCGATCAAAGCA	CGTCAATTTTCACTGCGGAAC	+	+	-	-
	CL6914	CAAAGGACGACAATCACCAGTC	GTCTGGACCAAATGCAAACTCA	+	+	-	-
	CL6957	AGACTTGGGAACGACGAGTACA	GCTAACGACAGAGTTGCAAACA	+	+	-	-
	CL7084	GCTAGTGGAACCACTGCAAAAA	CATTCTTGTTCGCCGATTCAG	+	+	-	-
	CL7085	CGATAGACCCAAACGCAAAGAT	AGCAAGCATTGCCCTTTGTAAG	+	+	-	-
149	CL6803	TGAACATCTCACGGGCATAATC	TGGATCCTTTCTTCAGCCTAGC	+	+	-	-
	CL6976	GGAAACGAAAAGGGTCTATCCA	CGCTTTATAATCCAAGCGTGAC	+	+	+	RS2CL6976s
	CL7073	AACCGAAGCTTCCACAAGGATA	ATTCTCACAGCGCAGACACATT	-	-	-	-
	CL7124	ACCACCTCTACCACCATTGCTT	CCAAGAAGAGGACCTTCAGCAT	-	-	-	-
	CL7127	CCTGAGAACATGAGGAGTTTCG	GGGATTGAAGCTGACCAAAAAC	+	+	+	RS2CL7127s
	CL7158	TGATCAAATCAGCGGTTCCTC	GCAAAACCACACTGACACACAA	-	-	-	-
150	CL6785	GAGGATAAAATTGCGGAGCTGT	GTATTTCTTGTCGCGCGATGTA	-	+	-	-
	CL6837	ATTGGCTGATCTCAACAAGCTG	CTTACTGCATTCCCTGGTGAAA	+	+	+	RS2CL6837s
	CL6910	TGTTCTGGAAGAAAGACGGATG	TGGGCAAGAGACGACGTAATAA	+	+	+	RS2CL6910s
	CL6982	GAAGAAGCTCCCGTCGAAGTAA	AAGAGAGGGTGATGCAAAAAGG	+	+	+	RS2CL6982s
	CL7138	CGTCTGTATGGACCCGTGATTA	TTGGTTTGTATCGAGAGGCTGA	-	-	-	-
	CL7142	GGGAACTTTTCTGGAACACGAG	CCTTAGCAGCCCAGTGTTTCTT	-	-	-	-
151	CL7188	ATTGAGACATGGCTTCGTGTGT	CCAAAAGCTGAACCCCTAAACT	-	-	-	-
	CL7193	TGGGTTTGTCATGGCAACTATC	TATCGCAGAAAATCACCAGCTC	+	+	-	-
	CL7194	TTGCTCTTCTCCTCTCATCACC	ATAGGGTGGCCCTGGTTCTAAT	+	+	-	-
	CL7228	CCCACATTTTCTTCTTCGATCC	ATTAGCGATGGTTTCGACAGTG	+	+	-	-
	CL7230	GAGACGATGATGAGCTCAAGGA	TATGGCTCTCTAGTACGCAGCA	+	+	+	RS2CL7230s
	CL7259	AAAACGTTTACCCTGCCCCTAT	TCAATGCTTAGAGCGGTTGAGA	-	-	-	-
152	CL7201	TTGTCGGAAGCTAGGAGGGATA	GAAAACGTAGGGCTGGAAAATG	+	+	+	RS2CL7201s
	CL7208	ATGTGCCTTAGCTGTGTTCTGC	GGGGAGGATTTGATCTTTGCTT	+	+	+	RS2CL7208s

	CL7240	CCAAGATGGTCCCACAAGCTAT	ATGGCTCAGAATGATACGGTGA	+	+	+	RS2CL7240s
	CL7249	GCAAGCTCTGAATCTCTCTTGC	ACAGTATTGTTCCACGCAAACG	+	+	-	-
	CL7253	TAAGTTGAACCTCTCGCCTCCT	TCCCTACCTCGGCTTCTTCTAA	-	+	-	-
	CL7284	CTCTCAAGCGGTACAGAAACGA	ACCAAACAGGAGCTGTGAAACA	+	+	-	-
153	CL7301	GATCCCGGAGTACGTTGAGTTT	CTACGACAACACTTGGCATGAA	+	+	-	-
	CL7302	CTCTTGAGAGCAAACGCAGAAA	GAAGAACCCATTGTCCAAAAGC	+	+	-	-
	CL7327	CGTTGTCTCACGTGACCTGTTT	ACAGGTTGGCCCCTTTTTACA	+	+	-	-
	CL7397	TTCCGGCTTAAGCATTCCTCTA	TTCACAGTTGCATCATCAGTCC	+	+	-	-
	CL7408	GTGCAAAGCATGACTCAGAGGT	GTGTGCATCTAAGAAGCCTGGA	-	-	-	-
	CL7442	ACACAAAACCAATCAGCCACAC	TCTTCATCGTCACTTCCACGTT	+	+	+	RS2CL7442s
154	CL7358	CTCTAGGGATTGCGGATAGCAT	GTTGAACAAGGGAAGCGAAAAC	+	+	+	RS2CL7358s
	CL7378	GAGGCAATACACATGGGACAAG	AAGCTGCACAATAACAGGGTGA	+	+	+	RS2CL7378s
	CL7419	CGAACAAGGAAGGCTACTCTCA	TACACAACTTAGCGAGGCATGA	+	+	-	-
	CL7432	ATGCACAATCGTGTGGGAGATA	CACACATATAAGTCGGCGACAG	+	+	+	RS2CL7432s
	CL7461	GCACAATCATGAGCTACCAACC	AAGCTGGAAACGAGACGAAAAC	+	+	-	-
	CL7467	GAGTCCTCTTCACGCTTTTTGG	TGTCCGGTCAGCTTTTTAACCT	+	+	+	RS2CL7467s
155	CL7351	TAGGTACTTTGGGGGAAGAGGA	CCGACCAAGAAAACTGAAAACC	-	-	-	-
	CL7398	AAAGCAGAGGCCTACCATGTTT	ACCAAACAAGTGGCTGTTCTGA	-	-	-	-
	CL7406	CTTTTCGACTCCATCGTTAGCC	GGGGCAAGAACAGAGAATGAGT	+	+	+	RS2CL7398s
	CL7417	TATAGTTCCCAGCTGCCACAAA	CTCACCGCGAATATGACGATAA	+	+	+	RS2CL7406s
	CL7449	GGATAATCAAGCCGAACCAGAT	CAAAAGACCCCTCCAAGAGAGA	+	+	+	RS2CL7449s
	CL7471	GGACATCTCTTCTCGCCTCTTT	CTGTGCATAAGCCATTGAGTCC	-	-	-	-
156	CL7289	CGTGTATGAGAAGGGGAGGAAT	ATCAAGGCCTTCTGCAAAACC	+	+	-	-
	CL7311	GGGCAGAACTGGTGTTCTGTAA	TTGCCCTCAAAGAGTCATCAAC	-	-	-	-
	CL7403	ACTCTGTGGACCAGGTGAAACA	TTAACAACCGTGACCACGAAAC	-	-	-	-
	CL7413	ACCAGCGATTCGTTTTCTCTCT	TCTGTGGCACTTCCTTCTTCAC	+	+	-	-
	CL7415	GATCCGAAATCTAGAGGCATGG	TGCAATCACACGTTCACAAGAC	+	+	-	-
	CL7473	CGGTTACTGATGATTTGGGAGA	GTGACCGCACTGCTGTTTGTAT	+	+	-	-
157	CL7387	TGATCTCAATCTCTGGTGGTCA	AAAAGGGGAGGCAGAGAAACAT	-	-	-	-
	CL7396	TCCACAGACTGCTGAAAGGAAG	CAGAAACGAGGGAAACAAGTCA	-	-	-	-
	CL7414	CCAGCTGGTGCAGAGAACAATA	ACCGTTGAACCGTGGATCTT	+	+	+	RS2CL7414s
	CL7425	ATTAGCGAGGAGGGAGATGATG	ATGCTGAAACCGAATCTGAACC	+	+	-	-
	CL7460	CAAGGAAGAAGACGGAATCGAC	TTGACCATACCACACACCTTGG	+	+	-	-
	CL7464	GAAGGATACTTTGCATGGTCGT	TAGCCGCAAAAGAACCCTTAAC	+	+	-	-
158	CL7486	CAAAAGAAGCGAGAACCCTAGC	AATGAAAGTCGGACCTTGTGGT	-	-	-	-
	CL7501	CTCCCATCACTTAGGGACCAAT	TCATCTGCCTTGAGACTTGGAA	+	+	-	-
	CL7533	CCACCCCTTTGTTTCTTTTCTC	AAGTACCAAGCGTGGAAAGGAG	+	+	+	RS2CL7533s
	CL7538	AGGCTGTTTCATGTTCCCTTTC	CTCCTCCTCATCATCATCATCG	+	+	+	RS2CL7538s
	CL7679	GTCGAAGTATGGAACACCAACG	TCGTCTCCATGTTCTCCTGAAA	+	+	+	RS2CL7679s
	CL7691	TCTTAAGAACGCGATTGACTGG	CGGACAAACCGCTAACATCATA	+	+	-	-
159	CL7541	ACCGATCTGCTATGCTCACTTG	CATGGCTTAGCGAAAAGTCACA	+	+	+	RS2CL7541s
	CL7572	AATGGAGAACTCGCCCAGATAC	AATCGAGGATGCTTGGAGAGAG	+	+	+	RS2CL7572s
	CL7624	TAATGACGACAAGGCGAAGAGA	ATGCTTCAGGCACCCAAGTAAT	+	+	+	RS2CL7624s
	CL7665	GGCTAAGAAAAGCTTCGACCTG	GATAACAGAAGGAAGGGGGAAA	+	+	-	-
	CL7713	AGGCTTGACGACCCGTCTATAA	ACCCGACATTAAAACCAGAACC	-	-	-	-
	CL7735	GAAGGTTGCTTTGTTCGGAGAT	TCACCAAGTCACCCTCAAAAGA	+	+	-	-
160	CL7586	AGGAGCCTTCAGAGGTCAACAA	GAAGCGTATGTCGCTTTCTCAA	-	-	-	-
	CL7587	AACAATCCATGGAGAAGGTGCT	CCTCTTCTTCGTCGCTGAACTT	+	+	+	RS2CL7587s
	CL7601	ATAGATCATGCCTGTGGAGCAA	ACCATAACGATCCCACGAGTCT	-	+	-	-
	CL7675	CAGAACAAGTTCCCTCACACGA	CTTGGCCTTGGCAACAAAAG	+	+	-	-
	CL7692	TTTCTCCCAGTGTGTTTTGTGG	CTTGATGGAGACGACAAGGATG	+	+	-	-
	CL7712	ACGAAGCTGCTCTTCGGTTTAG	AGGTAACCGGTTTCGCTAACTG	+	+	+	RS2CL7712s
161	CL7506	TATGGCAACAGAAGCTTGAGGA	AGGTGAAACAAACGCACATAGC	+	+	-	-
	CL7549	GATCTTATGAAGCCAGGGGAGA	CGCCCTAAAACGGACTGAAATA	+	+	+	RS2CL7549s
	CL7655	CCACCCGACAACATACTACGAA	TCTCAAACAGAACAACGCCTTC	+	+	+	RS2CL7655s
	CL7717	CAAAGGTAAAGCTCGAGTGCAA	TAGAAGATGACGTCAGGGACGA	+	+	+	RS2CL7717s
	CL7754	TGCAGGAGTCTCGGTTTTGATA	TTCTGCCCCCTACTTTTCACTT	+	+	+	RS2CL7754s
	CL7778	TGCACACATTACAGTCGCTACG	TGCAGGCTCCATATAACCTCAA	-	-	-	-
162	CL7566	CCTCGAACATACGATTTCTTGC	GTGTTCCTAAGGCTTTCACTGG	+	+	-	-
	CL7592	TCCAAATGAGCTTCCGCTAATC	GAAGTAGCAGCTTGCGAATCCT	-	-	-	-
	CL7622	TCAACATCTCCAGCTCCTTCTG	TCAACCTTCCTTCCATCGCTAC	+	+	-	-
	CL7649	TGTCCCTGAGAGCTTTGACTTG	TGGCTAAGCTTGTGGATGGATA	+	+	-	-
	CL7700	TCACGACACACACTCATCAACC	GCTCGTTCAGTAGGCTTCTTGT	+	+	-	-
	CL7702	GGAGCCCAGAAAAACCCTAAAA	GCGTGGTACATTTTCCTCAAGA	-	-	-	-
163	CL7559	GCTTTGGTATGAGGAACCCTTG	AACCTTCCCCCAAATCTCATCT	-	+	-	-
	CL7569	TGCTATTTGGTGAAGGGAAAGG	CTTCAGACGAATTTCCAGATGC	+	+	+	RS2CL7569s
	CL7598	AGTGGAAACAAAACCTGCGAGT	CTTCCCATTGAAAACCCTGTGT	-	-	-	-
	CL7671	CGTTTAAAGCAAGCCACCTCTT	CGACTGCCTGAAAATCAATCTG	+	+	-	-
	CL7677	ACGGAGACAAGAAAGACGAACC	ACCTGCTCCAGCTCTGTTCTTT	-	-	-	-
	CL7728	CGCGGAGATGAAACCGTTAT	CTCTCAGATTTGCGGAAAAAGC	-	+	-	-

164	CL7786	GGCTTTCCTGAAGTGGTACAGA	ACCTAGAGGTGTTTTCCCCTGA	+	+	+	RS2CL7786s
	CL7789	TGATATCCTCGGTTCTGACCAA	AGACACTAACCCATGCCCTTGT	+	+	-	-
	CL7824	GAGTGTATGGGATGGCAAGTCA	CCCCATCTCCACCATCTTCTTA	+	+	+	RS2CL7824s
	CL7837	AAGATGCGGATTATGCAGTGG	AACATCGTCGTTGCGTATTCAC	+	+	+	RS2CL7837s
	CL7893	ACCGTGAACATAAAACCCAACC	AAAATCGATGGGCCTCACATAG	+	+	+	RS2CL7893s
	CL7896	TCTTGTTGACGCGATTGTTG	CACGCATTCACACACACACAT	-	-	-	-
165	CL7817	CGTCTGTATCAAAACGCCTCCT	TTGGGCGTTAGCATAGCAAGA	+	+	-	-
	CL7849	TGGACTTCGCCAGTTCTACAAA	AGCCACCGAGTAAACATGAACA	+	+	+	RS2CL7849s
	CL7866	GAGAAGATCAACCCCAAACTCC	TGCTCACAGCTTCCCAATACAT	+	+	-	-
	CL7874	CTGAGACCTCCCTGAGGAAAAA	TTTCCCATAAACCGCTCTCACT	+	+	+	RS2CL7874s
	CL7884	ATCCCACCGCATATTCTCTCTC	GCTTTAAGGACAGCGCCTTTAG	+	+	+	RS2CL7884s
	CL7902	CTTCAGATCAGCCTTCCCAACT	CCAAGAAGTTGAACAGCATGGA	+	+	-	-
166	CL7810	GACCAGGAGGACCATATGCTTT	AGTAAATCCCCGAAACGGAAGT	+	+	-	-
	CL7834	TAACTCTTCTGGAAACGCGAAC	CAATGCTTCGGAAGGAAGAAGA	+	+	-	-
	CL7839	ATACAAGGCGATAAGCGACGA	GGAAGAGGCAAATGTTTTGGAG	-	+	-	-
	CL7852	AACAAGGGGGAAGAAGTTGTTG	ATAACATCACAGGGCAGGAACA	+	+	+	RS2CL7852s
	CL7900	CAAGGCTTTTCTCCCACTGTCT	GCGAGCTTGTTGATGACTCTGT	+	+	+	RS2CL7900s
	CL7907	ACTTCTGGAAGGGACTCAACCA	GTAAATGGCAAAGTTGGGGTCT	+	+	+	RS2CL7907s
167	CL7779	TCTCGAAGAGAAGCTGAAATCG	AAAGCTTCGAACATGTCCACAC	+	+	-	-
	CL7791	TCTTCAAGGAACACACTGCAAG	TTGAGAGGTACCTAACCGGAGA	+	+	-	-
	CL7800	CCAATAGAACCCCAAGCAAGAA	ATCTCCCAGAAAAACCCCAACT	+	+	-	-
	CL7803	CTTTTCGCGTGCCACTTATCTA	ACGCATTGCATGAATCGAAC	+	-	-	-
	CL7828	TCTTCCAGATGGCAGTAGCTCA	ACTTCACGGAAGGCTTCAGATT	+	+	+	RS2CL7828s
	CL7908	GAGTAACGTCGAGAACGATGGA	GGCCATTTGTTACTTGGTTTCC	+	+	-	-
168	CL7953	CCGTCTCAACAAATAGCTTCCA	AGGTCTGTGCCCAGAAAATGAT	+	+	-	-
	CL7992	TGTCTCTCTGCTTCCATGTCGT	GTATCTCCGGCGGTTATTTCTC	+	+	-	-
	CL8032	GATTGACTTGGCGAAGATGGT	TGGTGGGTGTCAAAAAGACAAC	-	-	-	-
	CL8058	ACTGTATGCGTCAAGCCATGAG	AGTTCATCATGGCATCTCATCC	+	+	+	RS2CL8058s
	CL8077	AAGTCCAGCGTCTGTGACATTG	CCAGAACGAATTTGGGTTTCAC	-	-	-	-
	CL8180	AATGAAGCTTCTAGCGGAGGTG	AGATCACCGCTTTTCTCCTTTG	+	+	+	RS2CL8180s
169	CL7912	GGCAATACGTTTCACTTTAGGC	GTGGAAGTGGTCGACAATCTGA	+	+	-	-
	CL7976	CAGGAAGCTTCTCCTCGTCAAT	ACAAGAACGAACAAAGGGTGGT	+	+	-	-
	CL8011	TTCACTACGCTGCTCAAGGTTC	CTTGATCAGCTTCGGTGTCATC	+	+	-	-
	CL8143	GGATAGATCAAACATGGCAACG	TAGGAGCCGGGTTAGGGTTATT	-	-	-	-
	CL8167	ATGGTGAATTTACGCCGTCAC	CGGGGGTATGATTCATCATCTT	+	+	-	-
	CL8170	ACCACCAGATAAAAGCCAGCTC	CAAGTGGTGGCTGTGGAGAATA	+	+	-	-
170	CL7921	CCAGAGGGAGCGGTGATAATAA	TTTAAACACCCCATCCTCATCC	-	-	-	-
	CL7936	AAAACATAGGGATCGCTTCACC	CTTATCCTTGCCACCACCAAAC	+	+	+	RS2CL7936s
	CL7968	ACAAGACGCATCAATGTCACCT	GAAACCCCCTTAGCCTCTTTTG	+	+	+	RS2CL7968s
	CL7975	GAAAACGTGTGTGAGAGGGAGA	AGCAACAACGCAGGTCTGAATA	+	+	+	RS2CL7975s
	CL7982	CGAGAAACCAAACCCTAAGTGG	GGGCTTCACAAGAAACCAAACT	+	+	-	-
	CL8042	GCCAAAGCTTTACTCCCAAGTG	CATGCCCCAAGAAGAAGAAGAA	-	-	-	-
171	CL7951	GGGATGTCTGAAATTGGACCAT	CTGGTGTTGGTGTTGATGTTGA	+	+	+	RS2CL7951s
	CL7974	TTAACCCAAAGCCGTCTCTCTC	AAGGAGTGCTCGTCAGAAAAGG	+	+	-	-
	CL8008	TGGAGCCAAGAAAGTGATGTGT	CAAGCAATGTGTTGAGCCAAAG	+	+	+	RS2CL8008s
	CL8037	TCATCTCTGCTCCTGAACTTCG	ACAAATGCAATCCCTTGAGGAC	-	-	-	-
	CL8124	ATTGGTCAGCTAGGTTCGCTTC	GTTCAATCGTGTGATGCGTTG	+	+	+	RS2CL8124s
	CL8144	AGAATGCACCGATGCAGAAGTA	TGAACCAAACAACATGGGTCTC	+	+	-	-
172	CL7978	TCCGATTCTCCTGTTCCTTCTC	CCGAGAAGCTTGACAATGTGAG	+	+	+	RS2CL7978s
	CL8020	TCACACAATCACAGCTCCCTTT	TCTCCTCTCTGCTTTGTCTCCA	-	+	-	-
	CL8045	TGGAGGGACTAATGAGATTTGG	GAAGAAACGGTGGAGGCATAAG	+	+	+	RS2CL8045s
	CL8056	CTGGTTTGACTTTGGGGTGAAT	CGGAGTTCTCATCTTCAGGACA	+	+	-	-
	CL8057	CGAAATGGAGAGGCTAAACTCG	TCCCTCTGAGAAGATAGGCAGA	+	+	-	-
	CL8075	ATTTCCATCTTGTCCTCCCAGA	ATGGAAGCTTGGACGTGAGATT	+	+	+	RS2CL8075s
173	CL7916	ATTGTACAACCGGAAAGGCAGT	TAGTAACCGTGGATACGCAGGA	+	+	+	RS2CL7916s
	CL7973	CTTCTCCAAAGTTCCTCCGTTG	AACAGAACTGTGAGTGGGCGTA	+	-	-	-
	CL7997	CGAGAGACGAAAGATGAAGACG	AGGAAGTTACGGATCTCGATGG	+	+	+	RS2CL7997s
	CL8027	ATGGTGCGAAGAAGAGAAAACC	TCGAGAAAGCTGATGAAGATGC	+	+	+	RS2CL8027s
	CL8102	AGGTCTCCCTCGCCTTAAAAAC	GATGCAAGAAGTGAGCAGGAAA	+	+	+	RS2CL8102s
	CL8104	TACGAGACGAAATGGGATCTGA	CTGGGTCCATAAACGCAGAGAT	-	-	-	-
174	CL8207	GTTCCATATTAGCAGCCCTTGC	GAGTGCGGGAAGAGTTTGATTT	+	+	-	-
	CL8334	ATTTCTGCGACTAGGCTTGGAG	CGGTCATTGACTTCGACAAGAA	+	+	-	-
	CL8338	AGCTGCGTGGACTAGAAGATCA	TGCCATGTTCCTTAATGGTGTC	+	+	+	RS2CL8338s
	CL8371	CACAAGACCAAAACCCTCACTG	CTGTAGGTTTGGGATCATCGAA	-	-	-	-
	CL8380	CTGAACTGGCATCTTCACTTGG	GGCTTATCACCTTGCTTGATTG	+	+	-	-
	CL8392	GCTGGACGAAGTGGGTTAACTA	AAACCTATACAGCCCAGGCAAA	+	+	-	-
175	CL8283	CGTTAACTGAAAGCCACTCGAA	CATTAGGTTCATCCGCGTGTAA	+	+	-	-
	CL8332	GTCATGAAGAAGACGCAACACC	CCTCAACGTGGAAAACATAACG	+	+	+	RS2CL8332s
	CL8361	GTACAGCGATCGGGTTTGTAGT	ATCTTTGTGGGTCTTGTCACCA	+	+	-	-
	CL8374	AGCTTTTGAGAGGAGGGATTGA	TCGCTTGAAGAATTGCTGCTAC	+	+	+	RS2CL8374s

	CL8375	GGCAAGGATTTCACTAGGGTTG	CACCACCACTTATTGCAGACAC	+	+	-	-
	CL8382	CACGAAAACAGACGTGAAGTTG	CCATTGTCATCTTCCCTCATTG	+	-	-	-
176	CL8185	ACAAAATCGGGTCAGGTTGTCT	TCATCACCTAGGCATGTTACCG	+	-	-	RS2CL8185s
	CL8197	TTTTACCACTCCTCATCGCTCA	TTGTCGTCTCTCCACCCAAAA	+	+	+	RS2CL8197s
	CL8211	ATGTGATGCAGGCAGCTAAAAC	AAGACGCAACAAAAGGGACAAC	+	+	-	-
	CL8276	TGAGGAATATGCAGGGGAGACT	CATTCCTGATCAGCTCCACAAC	+	-	-	-
	CL8294	TCCTTGGTACAAGGGGAGGATA	CTCGGCAATAAACGGGTAAAAC	+	+	+	RS2CL8294s
	CL8347	CTGGAGTCGGTTGGATGATGTA	CCTGGGCTCACAAAGTGAATCT	-	-	-	-
177	CL8183	GATAGCTGCAGGTGCAACACTA	GGAACCAACATTCAGACAAAGG	+	+	-	-
	CL8198	ATCTTCCACCAAAGCACTTCGT	GGTGAAGAGCAAAGGCAAATCT	+	+	+	RS2CL8198s
	CL8213	ACGTGGAACTGATTGGGAAGTT	TGAGTTAGCTGCGACATCATCA	+	+	-	-
	CL8214	GCCAGGATATCAAATCGGCTTA	TCCTACGTAACACGAACCAAGC	-	-	-	-
	CL8300	TGAATGGGATGTTGCTATGGAG	CCACGCACAAATAAATGGAGAC	-	-	-	-
	CL8311	AGCCGTTGTTGAAAAAGAGAGG	GTTTATGGGGATTGGCAATGAG	+	+	-	-
178	CL8284	TTGCTTGCTTCTGTGCTTTCTC	CAGCCGGCTTACAACAGATAGA	+	+	-	-
	CL8289	GAGACAGAACCGGAGTTGAACA	CCAATCTCCTAGCACCACCAAT	-	-	-	-
	CL8370	CGACCAATCACTCCAAGTTCAC	CAACCAAACACCATCCTTCATC	+	+	+	RS2CL8370s
	CL8399	CACAAACGGAACGAGAGATCAG	AGGCTTGTTATTGCTCCATGCT	-	-	-	-
	CL8436	CCTCCTCTTCCTCTTCGTTCAA	GCTGCTCCTCCATAGGTTGATT	-	+	-	-
	CL8449	TGGGTCAAGACCAAACTTAGCC	GACAAAACCATGCATGTCACAG	+	+	+	RS2CL8449s
179	CL8509	AGGGTGAGGAAGCTGAGAAAGA	AAATGTAAAACCGGAGGGTGGT	+	+	-	-
	CL8633	ACGATGCATCATACCGAGCTTA	CCCTCTAAAATCCAAACCAACG	+	+	-	-
	CL8676	ATTCTGTGCATGTATGGGAGGA	TGGGGGAAGAGAAACAAAGAGA	-	-	-	-
	CL8711	CGTGGAAGATGTACCAAGAAGG	AAGGAAGACGTGGAAATGGAAG	+	+	+	RS2CL8711s
	CL8744	GAAAGATGAAAGAGGCGGTCAC	AGATGGATGTGTTGGGTCACAG	+	+	+	RS2CL8744s
	CL8764	GATGTCGACGAAGAAGATCGTG	CAGGATGAGTTCGAAGAGGGTA	+	+	+	RS2CL8764s
180	CL8460	AGCTCGACACTCTCATCCACAA	AAACCTTTCTTCATCCCCAAGC	+	+	+	RS2CL8460s
	CL8531	AGGAGTCAGCAAATGCATGAGA	AATGACCAGAAGCCTCTTCCAG	-	-	-	-
	CL8594	ACATGCACTAACGCCTTTTGTC	AGATGTTATAAACCGGCCAACG	+	+	+	RS2CL8594s
	CL8607	GCTTCCGCAGATCCTTGAAT	TCCAGTGTTTGCAATGTCCA	-	+	+	RS2CL8607s
	CL8671	AAGAAGTTGCAGGAGAGGTTGC	ATCTCATAAAGCGGGCTCATCT	+	+	-	-
	CL8726	GAAAGAGCCGTTGAAGAACCTG	TGTTGCTGTGTGTTTTCCTCCT	+	+	+	RS2CL8726
181	CL8500	GCCTCTTCGTGTGATTGTTCAG	AATCTTTCGGCGAGATGCTAAG	+	+	-	-
	CL8523	GGCACTGGTGGAACTTCTTCTT	GTACACAACAATGGCGTCACCT	-	-	-	-
	CL8556	GCATTGCTTACCCTGGAAGAGT	AATTGCTAGAACCAACCCAGGA	+	+	+	RS2CL8556s
	CL8566	TCCTGTCAGAATGGAAGGTTCA	CCCATCCCAATTTACTGTGGTT	+	+	-	-
	CL8609	AAACGGAAGGGACTATTTCGAG	GCTCCGGGAGAAAATAACAGAC	+	+	+	RS2CL8609s
	CL8749	ATGGTTCACACAGGCTCAGGTA	CCAACAAACAAGAACCGAGACA	-	+	-	-
182	CL8453	ATCCGAGGATTGTGACAGTGTG	ATGGATCTCAGGTTCCCTCAAA	-	+	-	-
	CL8576	GAGCAAAGAGGAGGATGTGCTT	CCATATAGGCAAAACCGCAGA	+	+	+	RS2CL8576s
	CL8606	TTGCCTTAGGCCTCAGAAATC	TCCGGTCAAACCAGCTTTGTAT	-	-	-	-
	CL8658	GCCTGCGAAGAGGAAGAACTAT	TAAGAAACGTCTGAACCGAACC	+	+	+	RS2CL8568s
	CL8670	ACGACATCAAGCTAGCGAACAG	CCCAAGTGTAAGGAAAGCGAAC	+	-	-	-
	CL8729	ATGGCTGGCTACGACAAAGAAG	TTAGACAGCAGCAGTGGTGACA	+	+	-	-
183	CL8504	ACCAACAAGCTTCTCGCTAACC	CTGCCTATTCGCTTCTTCCTTC	+	+	-	-
	CL8506	GACTCGGGAGATATTAGCAACG	AAAGGCACCTACCAAGAAATGC	+	+	-	-
	CL8533	AGTGGAGGCATGAGGTTTCAAT	TAACATAAGCCGGGCCAATAAC	+	+	+	RS2CL8533s
	CL8562	ACTTGGGCTGCTTGCTTCATAC	ATGCAGCGTACGTGCTTATCAT	+	+	+	RS2CL8562s
	CL8733	TCCAAACACATTGGGGTGTTAC	ACATTGCAGGACTTCACCATTC	+	+	-	-
	CL8737	AGTTGATCCGGAGAAGATGGAG	CAGCTCACACTCGGAACAAAAT	+	+	+	RS2CL8737s
184	CL8597	TCAACTGGCGGTTACGTCAATA	GGGAGTTCGAATTCAAAGCAGA	-	-	-	-
	CL8630	TCACTGCTGTCTCAAACCCATT	CTTGCAAAAGGCTATGGTGCTT	+	+	-	-
	CL8674	TAAATGTCTCCGACGCTCACTC	GAGTTTTCCCATCAGACCCAAC	+	+	-	-
	CL8675	ACTGAAGGCTTGGTTGAAGAGG	GGCGTTGTGTACAAGCTCAATC	+	+	+	RS2CL8675s
	CL8688	CAATGCTACCACCGAATCAGTC	GCCACGAGGAAAGAAGAAAAGA	-	-	-	-
	CL8724	TCGGTACTCCTTCCCATCATCT	TTAACCACCCAGCGATCTGA	-	-	-	-
185	CL8787	AGAAGTGCCAGAAGGAGATGGA	TCCGGAAATTCATTCTGTGC	-	-	-	-
	CL8815	ACGAGAAGTTTGGGATCGATGT	CCAAAGTAAAAGCCCCTGCTAA	-	-	-	-
	CL8973	CAACCATTCGGTGGTACAAACA	GAATGATGCTAGGAACCCCATT	-	-	-	-
	CL8977	GAGTTTGATTGATGCCTGGTCA	CACAAACCGAAGTAAACGCAAC	+	+	+	RS2CL8977s
	CL9001	TATCTCCTCTTCTCCCCCACAA	GGGTACAAAACACAAGGCAAAC	+	+	-	-
	CL9017	TTCTGGAGGGCCGTTACATTAC	TGCTAACAGCTCACCTTGAACC	+	+	-	-
186	CL8976	TTAGAGGACGGATGTACCACGA	ACATAACGCAGTCTCTGCTCCA	+	+	+	RS2CL8976s
	CL9002	AAGAACGGAAAAGGAGATGCAG	CATTAGCTTGGGCGACAAAA	+	+	-	-
	CL9004	CGAGTTCCCGATTCTTCTTGTT	TATGACGACGATGAGCGAAACT	-	-	-	-
	CL9006	CTGCTGAGATCGAAACAGAAGG	ACTTCAGCGCACAACATGAGAT	+	+	+	RS2CL9006s
	CL9024	TTGCAACAGTTGTTCAGGGAGT	GCAATCCTTGTTGGGGGTAGTA	+	+	-	-
	CL9046	CGAGGAAGAGTTTCCGGATCTA	CCTGCAGAAACCACAAATCAAC	-	-	-	-
187	CL8767	GATAAGGCGCTTCGTTGTTACC	ACTACGCGCAGGTTAGGATTGT	-	-	-	-
	CL8789	TCGAATTCTTTGGAGGAGAGTG	TTTGCCTTTGTCTTCCTCTTCC	+	-	-	-

	CL9000	CAAGGCATTGCTCTTTCAGATG	TTCAGGACTGAGAACCACACTG	+	+	+	RS2CL9000s
	CL9026	GAACGTGAAGCCACTCAGAAAA	ATGAGTCGCCATCGAGTTGTAA	+	+	-	-
	CL9032	AGATTGGCAACTGATCGTCGT	GGGCTCAAGCAGAAGTCAAAT	+	+	+	RS2CL9032s
	CL9042	CGTGTGACAAGTTTGACCCTTC	ATCCAACAGCTTCCACTCTTCC	+	+	-	-
188	CL8765	ACGGTCTCTTAACCTGGCACAT	ATGGAGTGGTTCACATACACGA	+	+	-	-
	CL8809	GAGCAACGACGAGAAACAGAAG	CACCACATGCACAGCAAACAT	+	-	-	-
	CL8975	TGGGTGTCTGCTTGATAAGACC	TCTCCATTGAAGGACGCTTAGA	+	-	-	-
	CL8984	GCACCAGCAGTTAAACGAAGAA	GAGCTCAAGAATCACCGAACAA	-	-	-	-
	CL8999	GAAAGAAGCCAAGGGAATCTGA	TCTTCAGCGCATGGTAAATCTG	-	+	-	-
	CL9049	CAAAGCACGGTGTTAACGGTAG	CACTCATAGCAACATGCCAACA	+	+	-	-
189	CL8850	CAAACACAGAAGGGTTCATTGC	AGAATCAGCAACAAGCTCCCTA	+	+	+	RS2CL8850s
	CL8978	TGGAAGCTTACCACGTCGTTTA	CACAGAAAGCCAGCTAAGCAAA	-	-	-	-
	CL8986	AAGTATGGGCGATGGCTATCAT	TGTGAGGACTGACTGTGCCTTT	+	+	-	-
	CL8998	TCCGACTGCAAGTTAGGTCTCA	AAACTCCTCCGTGATTGACGTT	+	+	+	RS2CL8998s
	CL9005	TTAGCAGATGCGGTGGTCATT	AAACCAGGCTTCCTAGGAGAAA	+	+	-	-
	CL9029	CCGACCCCTTAATGGTTGATTA	TTCTCACAGTTCGGCGAAGTTA	-	-	-	-
190	CL891	CGCTCAGAAACTCTGTCGATTC	TCTGCTACCAAACGGGAAGATT	+	+	-	-
	CL904	GCATTGGGTTTTGACGGTTTAC	GCATCTGGTTCTCGCAGTCTAA	+	+	+	RS2CL904s
	CL991	TCACGTGCTTCCTGTCTTTGAT	ACGTGCGGTGATGAGGTATGTA	+	+	+	RS2CL991s
	CL996	GTTTTATGCCCTTTGCTCCAC	TTGGTGGGATTCTCACTTCCTT	+	+	+	RS2CL996s
	CL1049	TACATGGCTCCTTCTCTCGATG	CATCTCGAAGATGGGCTTCTCT	+	+	-	-
	CL1104	CTTCTCCAAAGTTCCTCCGTTG	AGCTCTCTTCCTTTCTGGGTTC	+	-	-	-
191	CL9057	AATGCCGGATGAGCATAGAGTC	GTCAATTACCAAACGGCGATCT	+	+	-	-
	CL9066	CAAAGGAGCTGCCTAAGGCTAA	CGACATTCTCCAGGAAAGACAA	+	+	-	-
	CL736	GGCTTTCCGTTTTGATCCTT	GACTTGAAGGAAGTGCGTGAAC	-	-	-	-
	CL849	AATGGGAGTACACCCGAAGAAT	GTCGGTTTGTCTGCTGGTTT	+	+	-	-
	CL1000	AACACTGGTTTACCCGACCCTA	ACATCATGGAGCAGAAAGTTGG	-	-	-	-
	CL1036	TCTCCATCTTCCTCATCGTCTT	TGCCTCCACCAAACTTGCTA	+	+	+	RS2CL1036s
192	CL738	CCCCTAAACCTCTTTTGAATGC	TCAGGATTATGCCAAGCTCTCA	-	-	-	-
	CL794	TGTCCCAAAGCTTTCAGTGCTA	GAAAGGAAACAGAGTCGGTGGT	+	+	+	RS2CL794s
	CL835	AAACCTTGAAACCCAGATGGAG	TCAACGGTCACGATTCTCTCTC	+	+	+	RS2CL835s
	CL887	TGAAGGCCGAGGATTTCTTTAC	ACCTTGCTTCGAGAAACCTCAC	+	+	-	-
	CL997	GATTTGTCCAATGCTGTCAAGG	TGTTGTCAGGAAGAGCTCCAAA	-	-	-	-
	CL1061	CACCTTGTGAGTGAAGGCAAGT	CGATCATTGCGATCACAGAACT	+	+	+	RS2CL1061s
193	CL771	TAACGCCATCTATGGAGCTGAA	ACCTCTCAACCATCCACACTTG	+	+	-	-
	CL792	GCCTACGTTTAGCCATTGTTTG	TGATTACTGTGGAGAGCCCATC	+	+	-	-
	CL839	CACCGTGATCTAAGGAGAGCAA	GTGAAGAATCAATGGTGGACGA	+	+	-	-
	CL995	GTTTCTTCTGATGGAAGCGTGA	ACCTTGGCACCCAAGTCAGTAT	+	+	-	-
	CL1060	TGAGCAGAGTTACGGATCTGGT	ACTCCCTTCCTCTTGTTCCTCA	+	+	-	-
	CL1103	GTGGTGCAGGACAAATCTCAAA	AGATTGCTCACAACGGAAGGAT	+	+	+	RS2CL1103s
194	CL867	ACTCAAATCCTTCACCGGACTC	AAAGCGATGGCGTAGTCGTAAT	+	+	-	-
	CL1046	CTTGCTTTCTCTCCACGTGTGT	GATCCACATTCCTCACCACAAA	+	+	+	RS2CL1046s
	CL1053	GCCTGGAGAAGCAGTAACCATT	TAATCAACTGCTTCCACCACCA	+	+	+	RS2CL1053s
	CL1054	CAGATTCCTCCAAATCCACTCC	GGGGAAACATCAACACTGCTCT	+	+	-	-
	CL1091	TAGGTCTGGCACCAAGGAGTTT	TGCCTTCCTCATCCTTCTTCTC	+	+	-	-
	CL1106	AACTGTGCGGAAATGAGTGAAC	ATTCCGATCCAGTTTCTCTTGC	+	+	+	RS2CL1106s
195	CL9072	CCTTCACCATCTCATTTCATCG	CGAGCATAGCATTTGAGAGCAT	+	+	+	RS2CL9072s
	CL713	TCCCTTTCACCGCTAGTTTCTC	TTGGGAGTCTTGTCAAGTGGAA	-	-	-	-
	CL859	TTGCTCCGTCTCTTATGGCTTT	CAGTGCAACAAGTGGGCTATTC	+	+	+	RS2CL859s
	CL893	ATCATGCTTCACTGCTGTTGGT	TGGAGCTTTGGATGAAAGTGTG	-	-	-	-
	CL968	CTCCAAGCTTTCTGGAGGACAT	CAACGGGCACTTTTCTCTTGTA	-	-	-	-
	CL973	AACCACCTTCGGAGAAGATTTG	CCAACAACATTCCTTGGTCAGA	+	-	-	-
196	CL1126	CTTTCGTGCAGAGACAGGAAGA	CCATCACATGGAGTCATCATCA	+	+	-	-
	CL1137	ATTCCAGCAGCTTTGATCCTTC	CCCAACACAAATGCTGCTAAAC	+	+	-	-
	CL1202	ACGGTTAAACATGTTGGAGGTG	GGAGCGAGAAATTCACAGAAAG	+	+	+	RS2CL1202s
	CL1275	CCAAGAACCGACCAATGAGAAT	TGCAGCTAAGCACTCTTTAGGG	+	+	+	RS2CL1275s
	CL1285	AGACATTAGTCCGGAAGGCAAA	AGCTACGAACATGGGTCAAACC	+	+	-	-
	CL1318	GCCGGTCCAACTAAAAAGGAT	AGCAAAGAGGAGGAGGAGAAGA	+	-	-	-
197	CL1107	TGGTCCAGCTCCAGAAAGTGTA	TTGCTCGCTCTCGAATTGTAAC	-	+	-	-
	CL1165	AGAGCAACTTGGTGTTCCGTTT	GGTGGGAATCCTTGAGGAAA	-	-	-	-
	CL1188	AAAAGCATTGCCGTAGAGGAGA	TATAAGTGCGCTCGCTGTCATT	+	+	+	RS2CL1188s
	CL1198	CTTTGATCCTTCTCGTGTCAGC	TGAGGAGGTATCGACCTTCCAT	+	+	-	-
	CL1227	CTCCCTATGGCCCTAAGCTTTT	GAAGGCCTTAAGCTGCAAGGTA	+	-	-	-
	CL1229	CTCAACCAAACCGTATCCACAA	ACAACACGGAGGGTAAACTGGT	+	+	+	RS2CL1229s
198	CL1196	ACTGCCTTATCGCTTCCCTCTT	CGTTGCTCGAGGAGAGAATCTT	+	+	+	RS2CL1196s
	CL1232	AGCCTGGTAAGCATCTCCAAAA	TCAGGTTTGAATCACTGCAAGG	+	+	-	-
	CL1248	TCCAATGGATCCCTTTCTTCTC	GCACGATCTGCACTCGTAGATT	+	+	+	RS2CL1248s
	CL1308	TTCATCCCGGGAAGTAGTATGC	CTGATCTTTCTCGGTTCGGTTT	-	-	-	-
	CL1319	TATCCTTCAACGGGTCTCCAAT	ATCCCTGGACTCATGGAAGGTA	+	+	-	-
	CL1325	GGGAGTACTCGACGAAGCTTTT	TAAAGTCGGAAGGGTTTTGAGG	+	+	-	-

199	CL1186	CGGCTCTTCAATCTAAGGTGGT	CATCACCACCAGAAAGCACTTC	+	+	-	-
	CL1236	TGGATGATCTTCTCTCCGTCAG	CCAAGACCAAAGCCTAATCCAG	+	+	+	RS2CL1236s
	CL1270	GATGAAGAACCCCGAGAAGAGA	GCTTCATCTTCACATTGGCAAC	-	-	-	-
	CL1287	AACGACCCGAAACTCACATCTT	TTCAGTGGGAAAGTGGTTGAGA	-	-	-	-
	CL1311	AAGGCAAAGCTACCGTTCTCTG	GGAGGGTCTTCTTGTCTTCCAA	+	+	-	-
	CL1328	CTCTAAACCCACCGCAAACAAT	TAGCGCAACCAATGTCAGAATC	+	+	-	-
200	CL1146	CCACTTGCTGTTTCCAAAGTTC	GAGATGAGCGCTCTTGTTGAGA	-	+	-	-
	CL1156	TCAAGCCTTCCACGGTTTAGAT	GTTGCTATGCTTTCCACAATCG	+	+	-	-
	CL1182	TCCAACTTCGTACACACACACG	AAATGGCTAGGGTCCAAGGTCT	+	+	-	-
	CL1192	TAACCATCCTCCTCTTCGCAAT	GATCGCTGGTTTTGAGGACTCT	+	+	+	RS2CL1192s
	CL1279	CGGGAGGCATAACCATAAAGAA	TATCATCACCGAGTTTGGATGC	+	+	+	RS2CL1297s
	CL1312	AACGTAAGCCTTCTTCGTTCCA	AGTGAAGTCAGGCCAAGCTTTC	+	+	-	-
201	CL1155	GAGATAGCCATTCCACCCATTT	TACCTGTATGCTTCTCGCTGGA	-	-	-	-
	CL1168	AGTGAAGAATTTGGCCACTCC	CAATCATCTCAGAAGGCTCGAC	+	+	-	-
	CL1170	AATACCAGCTTCACGTGTCTGC	AGATCCCACTGATGCAACAAGA	+	+	-	-
	CL1225	TTTCTTCCTCTGAACCACAACG	TCTGTGAGCACGGGTTACAAAT	+	-	-	-
	CL1228	TCTCCTCTTCCTCTTTCCGATT	AGTCGTTGACTTTCTCCGCAAC	+	+	-	-
	CL1334	GTCAACCCCAAAGCCATAATGT	AAGGATCGGAACTCGCTGATAG	-	-	-	-
202	CL1346	CGAAGCTGGGGTTGATTATCTC	ATGACGAGAAGGGAGGTTGAAG	+	+	-	-
	CL1353	AAAGAACCGATGGTGATTGGAC	ATCCCATCTGCACCTAGCTGTT	+	+	+	RS2CL1353s
	CL1433	AGCAAGCATCGATGGGTTTAGT	CCCAGTCTTGATTTAGCCATCC	-	+	-	-
	CL1476	GTCCGAGAAAAAGTCGGTTTTG	AGCCAGGACTCAGCGTTATCAT	+	+	-	-
	CL1486	CTGCTAGCCTTGTTTCCCTTGT	CAGGAAGCAAGGAATGCTCTTT	-	-	-	-
	CL1605	GAAGTTGATCTCTGCGGCTGTA	ACTGAAGCCGGTATCCTTCTTG	+	+	-	-
203	CL1382	CAGCCCCTGAGTAATAGCATTG	GATGGAGGGAAGAGGTCTGAAA	+	+	+	RS2CL1382s
	CL1532	TTATGGTGGTGTTGATGGTTGC	GCAGTTACATCAAAACCCGAAG	+	+	+	RS2CL1532s
	CL1544	GAAAACCGTGGAAGAAAGAGGA	AGCCGGGAATGAATACTTCTCA	-	-	-	-
	CL1585	AAAGGGGGAGCTAACAGGTTTC	CCCAGAATCAACAATTGCTACG	-	+	-	-
	CL1610	TACGAGAGACACGACGTGATGA	CAAACGAATACACATGCCCAGT	+	+	+	RS2CL1610s
	CL1697	CCATGTCCTGTAAGGCCTTTGT	GGGAAGGGGTAGTAGCGAAAAA	+	+	-	-
204	CL1391	GGGAAGCACTTGCATAATCAGA	ATGTTGCGTCCGTTGCTAAAG	-	-	-	-
	CL1429	GACAAGCGTGGTTACACACCAT	CCAAGGACAAGGTCAAAGTCAA	+	+	-	-
	CL1479	TTCGCGGATTCCACTACCTTAT	CAGTGACAAGGATCAGCGAGAT	-	-	-	-
	CL1492	GGGGATGGAAGGTTTGAGGTAT	AAGAAGATGGAAGGCAGAGCAA	-	-	-	-
	CL1636	ACGTTTCCTTGAACCGCTATGT	ATTAAGAAACGAGGTGGGCAAC	+	+	+	RS2CL1636s
	CL1682	CCATCAGAGCAACAATGGAGTC	GGCAAGCACGTCTTTAGAGGAA	+	+	-	-
205	CL1393	TCCTCTTGGCTTCTTTTTCAGC	ACGTGATCATCTCCTCAGGTCA	+	+	-	-
	CL1405	GGTCAGTTATCGACATGCTTGG	CATTGCTCCCACCAAATCTACA	-	+	-	-
	CL1414	TTCCAGGCTCGGTTGGTATATT	CCCATTTTTACATGGGATCTCG	-	-	-	-
	CL1437	AGCAAAATGAGCAGAGCACAAG	TAACATGCTGCCTCCAGCTATC	+	+	+	RS2CL1437s
	CL1449	ACCAGGACTCTTCCCACAAAAC	AGAGATTTAGGGTGGCCAACAA	-	-	-	-
	CL1675	AAACATCTTGGCTCGGATCTCT	TTAATGAGAATCCACCGTGTCC	+	+	-	-
206	CL1378	ACACTGCGCTTTACCACTGAAA	TTTATCTGCGCTTCCCAAGTCT	+	+	-	-
	CL1422	CCGACTCCTTGTGGAACAAAA	AGACATGAGACCCACAGGGATA	+	+	+	RS2CL1422s
	CL1471	GGAAAATGCAGAAGCCATTACC	TCTCAACGCCATCTCTCAAAAC	-	-	-	-
	CL1552	GGAGGAAGACGTATTGGTTTCG	GAGAATCTTCCTGCTCCTCCAA	+	+	+	RS2CL1552s
	CL1555	GACATTGACACGATCATCACCA	CATTTGTGGAAAGTGACCCATC	-	-	-	-
	CL1701	TTGTTGCCTTGATAACCACGTC	GCACGGATTGCAAGTTTAACAG	+	+	+	RS2CL1701s
207	CL1360	TCTCTTAACGACGGTTCCACTT	AGCATCTTCCCTCTTTGCAATC	-	+	-	-
	CL1483	GGGATGATCGATTACGACAACA	CCTCAGGGGAAATGAAAGGATA	+	+	-	-
	CL1576	GAGTTCAATGCATCGAGCAGAT	GTCCTCCTGTTTTCTCCTCCAA	+	+	+	RS2CL1576s
	CL1600	TGAAGGACATGGGGAGTGAA	AGCTTCTTTAGCCTGTGCTGCT	-	-	-	-
	CL1623	TGTTGACATTGCTGAAGGAACC	TATTGTCCAAGCTTCACGATGG	+	+	+	RS2CL1623s
	CL1642	CCAGGAATCGACCAATGAGAAT	TATGCGGCTAAGCACTCTTTTG	+	+	-	-
208	CL1712	TGGAGTGAGATGATGGATTTGG	TATTGCCGCCAGAGAGAGAAA	+	+	-	-
	CL1740	TGTGAGATCTTGGCGATCGTAT	GAGCAAACCAGTAGGCATTTCC	+	+	-	-
	CL1756	GATCTTGTCATCCGAGACGATG	TCTTCTCCCCAAGGAAGTTACG	+	+	-	-
	CL1795	GGACGACTGTTAGCAGCAGAAA	CCATACCGAACAAACGTGTCAT	+	+	-	-
	CL1915	CCACCGAAAAGCTTTGACACTT	CACCGGTTTACCCACCAAAAT	+	+	-	-
	CL2036	GTTGGTACGATCCCTCCAATGT	TATCCAAGCATTGTTGGTGTCC	+	-	-	-
209	CL1715	TTCTTGGTGGCTGTATGGTTG	ATCTCACACCGCTTACACCAAT	+	+	+	RS2CL1715s
	CL1726	TTAGTTCCGAGACGGGAAAATC	ATGACGCAGTGGTGGATGTAAC	+	+	+	RS2CL1726s
	CL1831	GTGGATGTCATGGGTTACAGGA	CCGTGGAAGGAGACAAGACTTT	+	+	+	RS2CL1831s
	CL1920	ACACTTTGTGCCTGGTCTCTCA	TCAGTGGAGTGGAAATGAAGGA	+	+	+	RS2CL1920s
	CL1963	TGGAGAAGGAGATGTCGATGAA	TCTTTCAAGAACTGGTGCTGCT	+	+	+	RS2CL1963s
	CL1983	GATGTTTTGGACGGTTCAAGTG	TCGGAAGCTTTAGCTCTCCATC	+	+	+	RS2CL1983s
210	CL1735	TTCTGCTTCAAGGGAAGTCTCC	CGTGGATTCGCAGAGACATAAG	+	+	-	-
	CL1828	CGTCCACTAAGGAAAAGGAGGA	ACCAAGTGGCGACGAAATTAAC	+	+	-	-
	CL1940	CGTCCTTCCTGACATGTTCAAA	AAGCTTCTCTCCACTGGGAATG	+	+	-	-
	CL1948	GCTCATGATTGATCGGCTACAC	TCTTCTTTCCCTCTGGGCTATG	-	-	-	-

	CL1956	GCAGCTGCAACTTTCCACTTTA	CTCAACTGCGGAAAGAGACAGA	+	+	-	-
	CL1989	CTGGTTTTGCTGGGGATGAT	AAGGGAGAGCACAGCCTGAATA	+	+	-	-
211	CL1733	ACTCCAACTCCATCCCTAACCA	CAGAAGGAAACTGAGGCCACTT	+	+	-	-
	CL1758	CCTCGGGAACATCTTCTCAACT	TAGGAGCTTCGGCGATTCTAGT	-	-	-	-
	CL1854	CAACATCTCCTCCTGAAGCTGA	TTCTCTGAGCTGATCCAGTTGC	+	+	+	RS2CL1854s
	CL1855	TCTCCTCCTCGCCTACAAAATC	CCCAAACGTGTAAGAGACACCA	+	+	-	-
	CL1903	CGCCATTGTAGTCCACCTCATA	AACTCCAGCTACGACCGAAATC	+	-	-	-
	CL1904	GTTACACCAAAAGCCCATTTCC	GCAACGAGTCTCAGATGCATTA	+	+	-	-
212	CL1723	ATCTTCCTGCTTGGAGCTCTTG	ATGACGCAGTGGTGGATGTAAC	+	+	-	-
	CL1755	CCACGGCAACTGAATTACTGAG	ATCATCTCGTGGCTTTTCACCT	-	-	-	-
	CL1845	AGATGGGATCCATTTCACAGC	TGACAACCGTCATTGTACACCA	+	+	-	-
	CL1880	AGAGGGGTTTTGGATTGACGTA	ATCCCCAACACTTTCACTGTCA	-	-	-	-
	CL1885	TCCGTTTCCTAGCTTCATCTCC	CGCTCAACTACGAAGCTCACAT	+	+	+	RS2CL1885s
	CL1992	ACCAAACCAGAGAAGGCATCAT	GGAGTAGCACGGAAAGGAAAAA	-	+	-	-
213	CL1734	CTTTCAAAACCCTGACCAGCTT	GAAGATTCTGCTGCACTCCTGA	+	+	+	RS2CL1734s
	CL1736	GAGAAAAAGCCAGGAGGAGGAT	TCTACGACAACATCCCAGCAGT	+	+	+	RS2CL1736s
	CL1792	CTTCGATCTTCCTCCAACGTCT	TGACAACGTGACTCCCAAGTCT	-	-	-	-
	CL1875	ACATCCCTGAAGGCCAAAGTTA	AATCAGTGGCGGATGAGCTT	+	+	+	RS2CL1875s
	CL1964	GAGAACATCGAGAGCAAGATGC	TGAGTGAGCAGTGGGATAAGGA	-	-	-	-
	CL1968	GAACCGTCGTAAGTGGGTGATT	ATCAGCGCAAGCAAACATTC	+	+	+	RS2CL1968s
214	CL2091	TAGCGTTTGCGTCTGGATTGTA	TCTGGAGTGGATTTGAGAAGCA	-	-	-	-
	CL2103	ATGCACGAGTTTCAGACAAAGG	CGCACGCATTCAAGAGTTTATC	+	+	+	RS2CL2103s
	CL2115	TTCACAGCATCTCAACAGCTCA	GGGAAGAGGAAGCAATGGAATA	-	-	-	-
	CL2123	TGTGGATGGATCGTCCTACTTG	GAAGGTATCGATGATGGTGGTG	+	+	+	RS2CL2123s
	CL2187	AAAACCCGACGGTGTCAAAC	TGGAAAACCTTTAAGGCGGTAG	-	+	-	-
	CL2291	CATAGCAACTCAGGCAAAGTGC	CCGGTTAAAGATGAAACCGAAG	+	+	-	-
215	CL2041	GCCCAGTCCTGTTCTTGAGATT	TAATATGGGTGGCCTCTGCTCT	+	+	+	RS2CL2041s
	CL2070	TTGTGCAGTACGACACTGAGGA	AGACTTGTCTGCAGCCTCCTTT	-	-	-	-
	CL2142	CTTCTCGGGGTATTACGTTTCG	GAAGACAGATCCTGCAGCTCAA	+	+	-	-
	CL2166	AATACAGGCCGTTCTCGTTTGT	CAGGCGATCTTGTTTTTCACAG	-	-	-	-
	CL2195	CCCACAATGAAAGACATCTCCA	GCAATTACATCACTGCCGTTTC	-	-	-	-
	CL2308	GAGACTGCATCTGGATTTGGTG	TTCACAGGAAGAAACCATGACC	+	+	-	-
216	CL2094	AGAGGATAAAATGGCGAGCAAG	GCGTTGGGCATGTTACATTT	+	+	-	-
	CL2100	AAAGTTCCTCTGTGCTCCGTTC	GGCTCGTGACTGCTGATTCTAA	+	+	-	-
	CL2149	GTGAAAGGAATGGCCTTGAATC	TTCTGTCAGCCTTGCACCTCTA	+	+	-	-
	CL2207	TTCGACAATTGGCTTCTTCTCC	CCGACCAGATTCAGTTCTCAAG	+	+	-	-
	CL2316	ACGGGTTACTCAACCTCGAAAG	AGACCTTCAAGCACACAACACA	+	+	-	-
	CL2321	TTGCATACTTGGTTACCCGATG	GAGCATCATGGCATAGGTCAAA	-	-	-	-
217	CL2125	TTGGAATCACCAGATTCACCAC	TAGCGACTGGAGCCTATTTTGA	-	+	-	-
	CL2140	TCCGGAGATGAAGCTAGGAAAC	AACCATCATCTCAAGCCCCTTA	+	+	+	RS2CL2140s
	CL2151	CAAGGGTAGAGCCGTAAAAAGG	AAGGAAGAGCCACTCTCGACAT	+	+	+	RS2CL2151s
	CL2174	TAGACTGGTTGTTTTCGGACCA	GACGAGGCTCAAAGGAGTGTTA	-	-	-	-
	CL2205	CGTTAACGGGTTCGATTACCAC	TGACAGCACCTTTCTCTCTCCA	+	+	+	RS2CL2205s
	CL2257	TGACATGGAAATGGATGAGTCC	AACGGAAAGTTCTGCAAAGACC	+	+	-	-
218	CL2054	GCACAAGAAACCCCAAACCTAA	ACGGCTGAGATTGAGAGCTTGT	-	-	-	-
	CL2114	TACGCCAAATCTTTTCCCAGAC	TGGTTCTTCTGGCTTATCACGA	-	-	-	-
	CL2122	AAATCCTCTGATCCCATCAACG	GCTTACAACACCTCGTTTCACG	+	+	+	RS2CL2122s
	CL2130	CGAGTTTCAAAACCCGAGTAGC	CGATCTGAGCGTTAAGAGCAAG	+	+	+	RS2CL2130s
	CL2275	CGAAGATAGCAAAGCTTGTGGA	GCTTGCCTTACTCGACAGCATA	+	+	-	-
	CL2320	TGGTATTGTTACGGCAAGATGG	GTGGGTACTCCGGGAATAACTT	+	+	-	-
219	CL2042	AAACGGAAGAAAGCAGCAGAAC	ACCAGGTAACCCCAGCTAACAA	+	+	-	-
	CL2047	CATCAAACTTGAACGTGGGAAC	GAAACCAGGTGCTCCTCACAAT	+	+	+	RS2CL2047s
	CL2086	GGTTACACTTCAATGGCGTCTC	GACGAAAGCAATGTCGAGAAGA	+	+	-	-
	CL2117	GCGACAAATCATACCCTCACTG	TTGAAATCGTGTGGCTGTATCC	+	+	+	RS2CL2117s
	CL2278	GGGTTGCAGAAGAAGCGACTAA	ATTGTCCGAAGAGGTTGGAGAT	-	-	-	-
	CL2300	ACAACGACTGAACCAGAATCCA	GTTTGGAGATCCGTAAGCAAGC	+	+	+	RS2CL2300s
220	CL2415	CTGCATTACCTTCACGTCTTCC	CGCTCTCATGAACCGATAATCT	+	+	+	RS2CL2415s
	CL2500	GCATTACTGAGGTTTCCTCCAA	CTGGAACCAATGCAACAAGAAC	+	+	+	RS2CL2500s
	CL2517	ATGCTCCTCCAATGGCTAAGAA	GACTCAGACGACGACAACGATT	+	+	+	RS2CL2517s
	CL2532	ATGGCAACACCTGTAACCGATA	CACTCGGAGGTTTTGACACTTG	+	-	-	-
	CL2616	AGAGACGGCTCCTTGATATGCT	TCATCCAAAACATGGCTACTCG	-	+	-	-
	CL2664	AGGACATAGTCCATCGCTCACA	TTCATGGCCAACTCTGTTATGG	-	-	-	-
221	CL2344	AACTGGTACGCGTTTGACAATG	AACAGGGCATGAGGATTTGTCT	+	+	+	RS2CL2344s
	CL2403	ACAGTTGTTCAGGGAGCATTGA	TGAGCATGAGTTGTCTGGGTCT	+	+	-	-
	CL2568	TCGTTTCCACTCCAAAGGTTCT	GGTGTTCTCAGCATCACGTCTT	-	-	-	-
	CL2576	AGATTGCGTTGAAGTGTTAGGC	GGGAAACTTCACACTTCGTTTC	+	+	-	-
	CL2693	CCAAGCTAGGTAAAAGCGAGGA	AATACGCAGTTACGGGTCGAGT	+	+	+	RS2CL2693s
	CL2708	GTTTGTGCCTTTGTGGCTCTAA	GTGGGTCGACTGGTTTGTTTAT	+	+	+	RS2CL2708s
222	CL2361	TCTAGATAACCACGTGGCGTTG	CACGGATAGCAAGCTTCACAGT	-	-	-	-
	CL2369	CCGGAATCTCTCCTCAAGACAA	AAACCAGGGCCTCTAGCAACTT	+	+	-	-

	CL2404	CTCTTTCACGCGAGCAGATT	TTTAAGCCTAACGGCGGAGT	+	+	-	-
	CL2539	TGATTCTCACCCTCACACCATC	AGATGCCAATCTCAGAGGAAGC	-	-	-	-
	CL2581	ATGGAGCAGAAGGGAACAAGAG	GTTCTCGTCACCGCTTTCATTT	-	+	-	-
	CL2713	ACCAACCATTCCAGATCCTCAT	AGGACTTGCCTATCAGCAGCAT	+	+	+	RS2CL2713s
223	CL2327	TCAGCTCGATCAGTAACGGAAA	GCCCAAGTCTTATCCCATTCAC	+	+	-	-
	CL2395	ATCAAGCCACTCAAGCCAAAG	ACGGTTTTCACAGGGACTAAGC	-	-	-	-
	CL2398	CCGACAAACACAACAACAACCT	TGCACGAAGAGCTCAAAGAAAC	+	+	+	RS2CL2398s
	CL2575	TCGTTCAACATGGTTCATAGCC	CCCCTGTCTCCAAATGCAATA	-	-	-	-
	CL2618	GACGTCGGGATGAAGAAGAAAG	GGAACTTAAACTTCGCCCTCCT	+	+	-	-
	CL2649	ATAAGCGATCAAGGGGTGGTAA	TAAGCCTTGGCTAAACCTTGGA	+	+	-	-
224	CL2556	GCATAGTAAACGTGTGCCAACC	CCTAAGCTTCCCGTTTTTGCTA	+	+	-	-
	CL2570	AACCGTGAGCTCGAAGTAATCC	CCCATGCATTGTTAGCTACAGG	+	+	+	RS2CL2570s
	CL2571	ACGTTGAGCAGAGACCAATACG	GCGATCATGGTATCTTCAGCAG	+	+	-	-
	CL2645	TACGGATCGGGTCAAATAAACC	CAAGATGGGACTCCTCACAAGA	-	-	-	-
	CL2647	ACCTTTCCAGTACCCCAAGACA	ACTTGGCTCAAAGCCTTGACAT	-	-	-	-
	CL2712	TTTCGTCTAAGGGTGCCTAACC	TCTCGCTTTAACACCAACGGTA	+	+	-	-
225	CL2328	GATGATCCGACTTGTGCATAGC	AAGACGACGTGAAGAACCTTCC	-	-	-	-
	CL2405	TCACTGCCACTACTTGCAAAGC	AGCGAGTGCATCAGAACGTTTA	+	+	+	RS2CL2405s
	CL2521	CAATGCTGTCAATGGGAAAGAC	ACAACGGAGGGTGACTTATGGT	-	-	-	-
	CL2540	TCAATGCTCAAATGTCCTACCG	AAGAAGAAAGGGTTTGGGAAGC	-	-	-	-
	CL2547	TATGGGAGTAGACAACGGCTTG	TAAGTGTGGGATCACCGCATAG	+	+	-	-
	CL2683	CTCCACTTCCTCCTTGCTTCAT	ATACAGAGGAAGGACCCGTCAA	+	+	-	-
226	CL2773	ATGGGAAACAGTTTACGGTGCT	GGCCGAACAATTCTTCTCTGAC	-	-	-	-
	CL2794	AACACATCACTGCGTGCAACTA	TCCCTGGATACCCTGACAACAT	+	+	+	RS2CL2794s
	CL2805	TTAACCGAGAAACCAACCCATC	TTTGCTGCTGGTCTTAAAGCTG	-	-	-	-
	CL2842	CTTCGTGACTGCGTTTGTCTCT	GTTGCCTTGCTCTGAGTCTGAA	-	-	-	-
	CL2883	CATTGCTATCACAATGGGTGGT	CCGGCTCTGTCGAGATATTCTT	-	-	-	-
	CL2922	CGGAGAACAATGGATCTGAGTG	CTCCCGCTTTTCGTTCTGTAGT	+	+	-	-
227	CL2803	TCTCTTTCTTACCTCCCCAAGC	CATCAAGAAATGTGCAGCTGTG	-	-	-	-
	CL2869	AAATGTTCCTACCCCAGCCATA	GCCCATTTTTAAACCCATCG	-	-	-	-
	CL2875	CTTCTCCCCTCCGACAAAATCT	TATACAACCGGTGGCGTAGTTG	-	-	-	-
	CL2887	CACTGCTCCCAAAAAGGAAACT	CCGTTGTGATTTCTGGTTCTTG	+	+	-	-
	CL2930	TTCGGTTTCGATTCTCCTCTTC	TGATTAACCGGAAGGCTCCTAA	-	-	-	-
	CL2931	AGTCATACTCAACCGTCCATCG	CAGAGCCAGTTTGAGGACCAAT	+	+	+	RS2CL2931s
228	CL2734	GGGATTTTTATCCCGGAGTTTC	AGTAGCATGACCAGGATCAGCA	-	-	-	-
	CL2756	AGTTTTGGGTGATTGGAGGTGT	CAAAAGGCAGTTCTGCGAAAG	+	+	-	-
	CL2845	AACCAAGCAGCTTAATCGGTTC	TTGCCTGAAACAGTTCTCCAAG	-	+	-	-
	CL2863	AGCTTTCAAGACTCCCCACAGA	ATGTTTCTCGGAACCTCTGCTC	-	-	-	-
	CL2901	AGTATGGTGCAAAGAAGCTCCA	GACCAAGAATGTCAACCACCAA	-	+	-	-
	CL2927	ACAGTCACTGAACATCCCAACG	CGTTGTGTCAACTCAAGAACGA	+	+	-	-
229	CL2857	CTTTCTTGATCAACGCATACCC	ATCAGGGAGATAACCCTGCAAA	-	+	-	-
	CL2916	ACGAGTTTCAAAAAGGGAGCAG	GTAGAAGATGCCTTTGGCGTTT	+	+	+	RS2CL2916s
	CL2923	CGGCTTCACGTAAAGAGACAGA	CTTGTGTTGCACACTCAAAGGA	+	+	+	RS2CL2923s
	CL2926	GGCGGTTTCTTCAGGGAAGTAT	CAAAGCCACAAAAGCAACTCAC	-	+	-	-
	CL2988	CCATCGTCATAGGATTGCTCAC	GGCACATGACCATCTGAATCTC	-	-	-	-
	CL2998	TTCACGGTTGGTGTAAGGCTAA	CCTCACCGTGGTGACAATACAT	-	-	-	-
230	CL3030	AGCTTGCTAACCTGGTGGTTGT	ATCTGGTCCCAGTGAGATGGAT	+	+	+	RS2CL3030s
	CL3071	TCCAAGAAAGCTACGACCAATG	CGACTTTAGGGGACTTGGAATG	+	+	+	RS2CL3071s
	CL3112	TCATGGCCTTAAGCTTCTCCTC	GTGAGGCTGTTCGTTGACATCT	-	-	-	-
	CL3133	AGCAGCTGATCTCGCAAAAAC	GCATTGTATGCATCCGCTACTT	+	+	-	-
	CL3255	CGGTCAAGTGGTATGCGTTTTA	AAACACATGCTCCTCTCCTGTG	-	-	-	-
	CL3259	CGTTGTGAAAGGTCTCGACTTG	TTTGAAGGCTGTCCGATAACAG	+	+	+	RS2CL3259s
231	CL3016	TGTTTCCATCTGGAGCTGATGT	GTTTCTAGCTTCGCAGCGTTTT	-	-	-	-
	CL3070	TCTTCAAGTGGTCAGGACGAGA	TCTTGAAACACCAGGGAGTGAA	-	-	-	-
	CL3138	ACAGAGAAAGCCTCCATCTTCG	GCTTTTTGCTTAGCCCACAAGT	-	-	-	-
	CL3144	TCGACGTTGGTGCTGTATCAAT	TTCTACTTCTTGCTTGCCTTCG	-	-	-	-
	CL3210	TGTTCCCAAGGATAAGGAAAGG	GACAATGCCGGGAAAACAAC	-	+	-	-
	CL3241	ATCGTCATCTCCAAGGCTCTTC	TGCGGAAATGGTTCTTGTAGTC	-	-	-	-
232	CL3001	AAGCCTAGGTCCAGCATTGAAC	GGGTGATACAAAGACACCCACA	+	+	+	RS2CL3001s
	CL3018	GAAGCTGCAGAATCATTGGAAG	TACCATCGCTACCTTTGTCACG	+	+	-	-
	CL3060	TATGGAGGGGCTCTTAAAGCTG	TATCCTCTCGTGCTCACCTGAT	-	+	-	-
	CL3091	CCTGATCCAGATGGGAGAAGAA	TGCCACAACTCTGATCGAAAAC	-	-	-	-
	CL3186	GATGACCACAACCGTCAGGTTA	TCTCATTGTCGGACGACATCTT	+	+	-	-
	CL3232	ATGCTGACTGCGAAAGCAAGT	AAGTCTTCCCAAGACCAAGGTT	-	-	-	-
233	CL3040	AGCGTTTGCAGGAATCATAGGT	AGCTCGGTCAAGAAACTCTGCT	+	+	+	RS2CL3040s
	CL3061	CACACTATCGGATTCGCACATT	TAACACGTGAACAATCCCTTCG	+	-	-	-
	CL3102	GGACAGTGAAAGTTCCCGTCTT	ATGAAGATGAGGCAATGCACAC	+	+	+	RS2CL3102s
	CL3118	CAACGTCCGGCTGATAATGA	CATCAAATCTCCAAGGTTGCTC	-	+	-	-
	CL3166	GTAATTCCGCTGGGTCAATCTC	AAAGCTTCTTCCATCTCGTTGC	+	+	-	-
	CL3171	TAGCCCTAATCTCATGGGTGGT	GATCGCCGAAACCCAATAGTAA	+	+	-	-

234	CL3050	CCCATTGTCTCCTCTTTGCTTT	TTCGTACTTCTGGGTGAGACCA	+	+	+	RS2CL3050s
	CL3084	ATTGTTCAGCACAACCCATCAC	CGAACCAGTCATCCCAATTCTT	+	+	-	-
	CL3124	GCTTCACAGCGACAAGTCATTT	ACTCTCATCCCTCGCTTTTGTT	+	+	-	-
	CL3148	TGGTTCATGTGGAGAGATCACA	ATGTCCATCTCCTGCTTGTTGA	+	+	+	RS2CL3148s
	CL3200	GCAGTTAGACGAGCTGCAGAAA	TACGGATTGGATCAGGGAGTTT	-	-	-	-
	CL3226	CATCCGACGATAGAATGGTGAG	CTACATCCCATGCCGGTTAAGT	+	+	-	-
235	CL2999	CGAATTGCAAGCGGATACTATG	TCACGCACACACACATACAATG	-	-	-	-
	CL3012	ATTCATTCGACCACAGAGCAAG	ATATTAAGCGGCCATGGGAAC	-	-	-	-
	CL3049	TCTCAATAGCAGGGATGCCTTT	AGTTTATTGGTCTCCGGGTTCA	-	-	-	-
	CL3052	TGTCTAGCTTTCCGAGAATCCA	GGGCACCAAGTTTTGGAGTAAA	-	-	-	-
	CL3077	GCTTCACCACTTCATTCATTCG	CAAGTCCTCTGCCACAGAAAGA	+	+	+	RS2CL3077s
	CL3268	GGAGATGCGAGAAGTGAGTGAA	CAGTTCGGTTCAAAATCCCAAG	-	-	-	-
236	CL3329	CTCTCCACAATCCACAATCCAC	GTTCCTGAAGCCAACGCTTAAC	+	+	+	RS2CL3329s
	CL3342	AAGGAACAGCTGAGCCATTAGC	CCACAATCCACGACTTCTATGG	+	+	+	RS2CL3342s
	CL3346	TTGGATACGATGACGGTACTCA	CACTTATTCAAAGGTCGCCAGA	+	+	-	-
	CL3418	ATGGATTCAACGGGACCTAGAG	AAGCAGTGATCCTTCACAAACC	+	+	-	-
	CL3494	GGGCAAAGGTAAAGCTTGAGTG	AGATGACATCAGGGACGATTCC	+	+	-	-
	CL3495	TCAGAAGCATTTGTTGCAGAGC	ACAGTGATGCTGTTTTCGATGG	-	-	-	-
237	CL3365	AGCTGGATCCAATCAGTGCTTT	CCCCGTATCGTTTACCTTCAAC	+	+	+	RS2CL3365s
	CL3366	TCTGCTTATACCGTCCATGTGC	GCCAGTAAAGGGAGCCATCTAA	+	+	-	-
	CL3429	ATTTGCGGAGTCGTCGTCTT	AACAACGACTTCGGGTTTAGGA	+	+	-	-
	CL3430	TTCCCCTCTTTGTGTCCTGTTT	GGAGGAGAAGAACCACTTTCCA	-	+	-	-
	CL3459	GCCCTTGTAGCTTTTGAAGGAC	ATTCCGCTTGCTAAGGAAGGT	+	+	+	RS2CL3459s
	CL3486	TCTTGTTGGTGTTGTGGTCGAT	TTTCTTGAGGCATGAAGCTCTG	+	+	-	-
238	CL3284	GTGTGGCTTGAGCAAGAGCTAA	CAGCTGCTATGGCTTCATTGTC	-	-	-	-
	CL3289	CTGGAACAACCAATCTCAACCA	CGCCTCTAACATTGGGTTTAGG	+	+	+	RS2CL3289s
	CL3332	AACATCAACGGTGCAAGTCATC	AACTCCGAAAGATCCCGACATA	-	-	-	-
	CL3353	CTGTTGTGGGAAATGGAACAAG	GTTTTGTTGACGCAATCTCTCG	+	+	-	-
	CL3445	GCATGCAACTGCTAGTCTCGTT	TTGTGGGAACAGCTTCCCTTAG	+	+	-	-
	CL3464	TCTGTCCAGCTTGGAGATTTTG	AGCGGACAGTCTTGTGAATACG	-	-	-	-
239	CL3314	CGGATTGCAGAGTTTGACTCAC	CCCATACAACAGCTTCCTGCTT	+	+	-	-
	CL3384	TGGGTTATCTTGTTCGTTGCAC	TGTAGGAAGAAGCTTCCCGTTT	-	-	-	-
	CL3396	GAAGCAAAGAGAATCCCGATCA	GTCTGCTGCCTGAAATGGTATG	+	+	+	RS2CL3396s
	CL3410	CGATCAACAACGTCTCCTTGTC	CATATCTTAACGCCGTCCATCC	+	+	+	RS2CL3410s
	CL3419	GGACATAATGCTGGGGATGAAT	TGCTCCACTTGAGGAAAATCTG	+	+	+	RS2CL3419s
	CL3480	TTCCTCGTCTTCAGACCACAGT	GGACTGGGCTGTTCATTCTTTC	-	-	-	-
240	CL3334	ATGTCGGTCACGGTTATGGTTA	AGAACTCCTGGGATCCTTTGTG	-	-	-	-
	CL3343	GGATGGTAACGAGGTTGCAAAT	ATCCCATTCTCGACTTCCTCAA	+	+	-	-
	CL3406	TGGGTCACTCGTTTCTATACCC	GAAAATGATGAGCCCGTCGTA	+	+	+	RS2CL3406s
	CL3421	ATTTGCAGCGCCTGTTGTAG	AGTGTGCAAACAGCAAGCAG	-	-	-	-
	CL3472	CAGGCAAGTAGACTGGATTGGT	CACCGCCAGAATCAGTAAACTC	-	-	-	-
	CL3483	CCCTGAGCAGTTGAAGATGATG	AGCACCAAGTCCTCTGTTTTCC	+	+	-	-
241	CL3308	ACGTGACAGAAGGCTCAGAGAA	TCAGGCTCCATGACTACATTCC	+	+	+	RS2CL3308s
	CL3309	GTCGTGTTGCAAGAAAGAATGG	CCAACACAACAGCACCTGGTAT	+	+	-	-
	CL3359	CCTGAGAGGAACCGGATTGTTA	GCGTATCCAGCGAAAAGAAGTT	+	+	+	RS2CL3359s
	CL3458	GATAGACGAGGACGCCAAAAGT	ACCAGTGGGAACAAGAACACCT	+	+	+	RS2CL3458s
	CL3470	TCACCATCCAACCGCATTTAC	CAACGGGGACAAGGATAAAGAA	+	+	+	RS2CL3470s
	CL3518	ATCCCGAGGAAAGAAAGATGCT	CAACCGCCGCTAATCTCAGTAT	+	+	-	-
242	CL3536	AAAGCTGGAACACCAAGACCAC	CCATCACCAAGAAGATGAGACG	+	+	-	-
	CL3544	GGATCCACGAAAACCCACTAAA	TAACTCCGGGGACAACGTTAAT	+	+	-	-
	CL3567	TTTGGCAGTTCTCAACGTCAGT	GATTGATATATTGGCGGCCTTC	+	+	-	-
	CL3603	GAGAACAAGGCTGATGTCGATG	TCTTCTGCTGCTCAAGTTTTGG	+	+	+	RS2CL3603s
	CL3626	GAGCTCAAGAGTTTTGCGTGTG	GCTTCGGTGTAGTCTTGCTTGA	-	-	-	-
	CL3718	CAGTTTTGAGGCAAGTTTGTGC	TCAAGTTCTGCTCAGGGGAGAT	+	+	+	RS2CL3718s
243	CL3523	GAAGTCATGACCAGATCGATGG	GACCTTGACAAAAACGCTACCA	+	+	+	RS2CL3523s
	CL3543	TTAAGGGTCTTCCTTCCATGCT	GAGGTCAAGATGGAGCAAGGTT	+	+	-	-
	CL3581	TCCTCTGATGTTGTTCCTGTGC	TTACACATTTCCCCACCTTGTG	+	+	+	RS2CL3581s
	CL3641	TGCGCAATACTTACCTGGAAGA	CGTCAAGAGTCCAGTTGCTGTT	+	+	-	-
	CL3695	AGGTATTGGAGCTTGCCTTTGA	ATTATCCAGATGCGGAACAGGT	+	+	-	-
	CL3835	CATGTTCTCAGGGAACTGCTTG	AGTGCATTGTGAGAGTGCTTCC	+	+	+	RS2CL3835s
244	CL3585	TCTCGGTCCATAGGAGAACACA	CCAACGCGTAGTTCAGGTTTTT	+	+	-	-
	CL3611	AATATCGAAAGCACCAGGCTTC	CTTCTTAACCGGAGCAATGACC	+	+	+	RS2CL3611s
	CL3614	ACCGGTATGTACATGCAACAGC	TCCATTCCCCGTAATAATCACC	+	+	-	-
	CL3644	GGGATAGATTGCAATGACGTG	AAATCACGAACACACGATGAGG	+	+	-	-
	CL3660	AAGAGGAGAGAATGGCTCAGGA	CAACAGCAAAGGCAAAGTCTGT	-	+	-	-
	CL3755	CATTCCAGAATCGGAACCAATC	TAAGGTGTGGCAAATGAGCAGT	+	+	-	-
245	CL3592	GCTGATCAACTTCCATCTCCAA	GATATGGGTGATGGTTGGGTTT	+	+	-	-
	CL3689	AACCTCCACAAAAACCTCATCC	AGGAGCATCATCAGGGGAAT	+	+	+	RS2CL3689s
	CL3739	TGGTGCAGAGTTAGAGGAATGG	ACACCGGGAATCACTTGTCA	+	+	-	-
	CL3762	CGCCGCTAAGGAAAGAAGTAGT	TCAGACGGTCTCGAAGTAGGAA	+	+	-	-

	CL3770	CTTTCGCTTTTGCTCTGTTCAC	CGGGAACAAGATGACGTTTACA	+	+	-	-
	CL3798	TGACAAAAGGCTTGAAGTGGTG	AGGCTCGCAGTAAAGGTGTTGT	+	+	-	-
246	CL3548	CTCTCTACGCGTTTCAGCGTTA	TGAATCCATTCTCGACGAGGTA	+	+	-	-
	CL3584	GGTTGCTGAGGAGGAGTCATTT	CACCGTGCAGAAGAAAGAGTGT	+	+	+	RS2CL3584s
	CL3606	CCGCTACCTCTAACCCGTTATT	ATCGACGACGTTACCAGCTTCT	+	+	+	RS2CL3606s
	CL3664	ACCGTCTCATCGATATCCCAGT	TCATCCATAGGAAGAGCAGCAA	+	+	+	RS2CL3664s
	CL3734	GTTGCCGTTGAAAGAGTTGAGA	AATCTTCCCACTCAAGCTGTCC	+	+	-	-
	CL3740	TGGGACAAGCTCTGGACTATCA	AACGTGAGCTTCACCAACTCAT	+	+	+	RS2CL3740s
247	CL3624	CATGTTCCTTCAAATCCCTGTG	AATATCCAGTTCGATGCACCAG	+	+	+	RS2CL3624s
	CL3633	AGCTACAACCACCACCGTCAAT	ACTCGTCTCAAAACCGACAGAA	+	+	-	-
	CL3692	TGTTTCCCTGTAGGCATTGACA	CACGAGAAACCACGATAAGCTG	+	+	+	RS2CL3692s
	CL3747	TGGACCGATCCATCTTTCTTTC	ACTTGCTCTTGCCACAACACAT	-	-	-	-
	CL3783	TCTTGACCATCTTTCTCCACCA	TGGAAGAAACAACAGAGGAGGA	+	+	-	-
	CL3824	CATTGTTCCACCTAACCAACGA	GATGTTGCGTTACGCTCTGGTA	+	+	-	-
248	CL3910	TGCAATGATCGGTCTCTCCATA	CCAACGCAAGTGCAGCTAATAC	-	-	-	-
	CL3940	AGATTATGAGTGGGGACGCAAG	GAAGAAACCACCGTTGCTAACC	+	+	+	RS2CL3940s
	CL3960	AAGAGTCTAGGGCTTTCCACGA	AGGTGGCGTGAAGACGTAAAAG	+	+	+	RS2CL3960s
	CL3975	GACACAACTCAAAAAGCGGTGA	TTGGTCATTTCTTGCCGTGA	+	+	-	-
	CL4012	ACCCCAAAATCAGGATCTACGA	GGTGACCATGAGCATCCTTACA	+	+	+	RS2CL4012s
	CL4015	AAATGTGGTGACCTGTTCGAGA	AGAAAACAGCAGGCTGGTTAGG	+	+	+	RS2CL4015s
249	CL3846	AAAAGGCTGAGGAGGAAGCAA	TCAATTGCAGGAACTTGAGAGC	+	+	-	-
	CL3858	CTGCAACTATTCTGGCATCCAA	AGTTTCCGCAGATGAGTGCATA	+	+	+	RS2CL3838s
	CL3892	GAGTGTCGAATGTGGGAATCTC	GGATCTTGCTTGATTCATGGAG	+	+	+	RS2CL3892s
	CL3925	AATAAGCCTCCTGGTGGGAACT	GCACCATTACCACTTCCCGTAT	+	+	-	-
	CL3966	TGGTTGCTGACAAAGTCATCGT	TCGTTGCTGAGGCTCTTGTAGA	+	+	+	RS2CL3966
	CL3997	GTTTGCGTGGTACGTCTTTTGA	AGATGGCTATAAACCGCGAATC	+	+	+	RS2CL3997s
250	CL3848	AGCCCATCCAAACTCATTCTCT	ATCATGCACTTGGAGATGGAAG	+	+	-	-
	CL3876	CGCACAAGGAGGGAGATACTTT	CGGCTTTCCAATGTAACCTCTT	-	-	-	-
	CL3933	TGAATTCAGTAGGAGGGAATGG	ATGCTTCTGAGATCCTCCAGGT	-	-	-	-
	CL3962	GGGTAAGATTGTGGAAGGCTTG	TCCATTAGCCAAGATGAGATCC	+	+	+	RS2CL3962s
	CL3977	AGCGATGAACACAACCTAATGC	CTGGTGGAGAAAGTCCTCTTTG	+	-	-	-
	CL3984	CACTTCGTCCCACGAAATCA	GAAGAGCCAAAAGCTGAAGAGC	+	+	-	-
251	CL3857	ACCCTCCAACTCTTCACTCCAT	TTTGGAGTGGATGAACCAGCTA	+	+	-	-
	CL3870	CGGTGGAGAATGTGCAAGATAA	TGGAATCAACTACCAACCTCCA	+	+	-	-
	CL3873	CCAACTACAGGCTCATCGACAT	CTTCATCAACCCAAAAGCAGTG	+	+	+	RS2CL3873s
	CL3929	CCCACCCTCATGCTTTATAACC	ATGCTCATGTACGCAGGATGTT	+	+	-	-
	CL4004	GGTCGTGTAATTGGCACACGTA	TCTTCAAAGACTTGGGAGCACA	+	+	+	RS2CL4004s
	CL4019	TCCCTGAGCTAACCCAACAGAT	TCTTCCTCTTCGTCCTCCTCAT	+	+	-	-
252	CL3836	AGAGGCTTCGTCCTTCATGAGT	TGGGAAAGATGTTCCTCGTG	+	+	+	RS2CL3836s
	CL3890	TGGGGTAGAGCAGGAATAAGAA	GACCGTAGAGCGCAGGTATTTT	-	-	-	-
	CL3894	AGAGGTTACGAACGGAAGGAAA	ATCCCCATGCCTATTCTCTTCA	+	+	+	RS2CL3894s
	CL3898	CAAGTGTCTTCACAGGCCATCT	AAAGAACCAGAACCAAGGACCA	+	+	-	-
	CL3899	CTTCTCTCTCGATCCCCAATTC	GATCGGAGAAAGATTCGTCGAG	+	+	-	-
	CL3921	CAGTTTTGGAAAACGGTGACTG	CGAAAGACGGAAGTGGTCAGTA	+	+	-	-
253	CL4057	ATTCGAATATCGCGCATCGT	CATCCGATCATGAACCGTACAA	+	+	+	RS2CL4057s
	CL4082	TGGTCAGTACAAAGGGCATAGC	ACAGAAGGTTAAGGTCGCTTCG	+	+	+	RS2CL4082s
	CL4121	TTTGAAGAGCTTGGTTCCCTTG	GGCAAGTTTGGCTGCCTATTT	+	+	-	-
	CL4126	GCATAGGTTCCGGTGATTCTTC	TTCCCTCAACTGCTTCCCTATC	+	+	+	RS2CL4126s
	CL4163	TTCCGTCCATAGCCAACTAACA	CCAAGAAACCTCGACTGGGTAA	-	-	-	-
	CL4170	TTGCTCTCTTCTTCACCTCCAC	AGTTCCAGGATTCAACGACCAT	+	+	-	-
254	CL4067	ACCGAATAGCCCTTGTCTTCAC	TCTTCAAAGAGAGAACGCGAGA	+	+	-	-
	CL4074	GAAACTCAAAGAGCAGCAAGCA	AACTGGCAGTGGTCTTTCACCT	-	-	-	-
	CL4098	TTTGAGTCGCCCATTTTCTACC	TATGTCTCCATCTCCGGACTCA	+	+	-	-
	CL4162	CAAATGGAGTGGGGTTTGTGT	CGACAATCCTCCGATCGATACT	-	+	-	-
	CL4169	GGGAGAGGAAGAGGTGGTTTTA	GCTAAACCGGGATAATGTCAGA	+	+	+	RS2CL4169s
	CL4189	CCTCCTGTTATCTGCGCATTTT	AATCAGAGATTAGCGACGGTCA	+	+	-	-
255	CL4044	AGCCCTAATCTCTCGACCAAAA	CTTGGGATTGGCTGATATGATG	+	+	+	RS2CL4044s
	CL4058	CACAAGAAGCAATCAGGTGGAC	TCCAAAGCTGTTGATCTGACCT	-	-	-	-
	CL4092	ACGTCGCTAGCAGTAACGTTTG	ATCGGAAGTGGCGTTGTCATA	+	+	-	-
	CL4096	CAGATGACCTCTTCTCCGGAAT	CATCATCCCAGGAGGGAAATTA	+	+	-	-
	CL4136	GAAAGAGTTCGCGGGAAAGATA	TGATCACCGAGATTAACGACGA	+	+	-	-
	CL4178	GAAAAATGGGTCTCAAGGCTTC	GCGAAACACGGTTGTGATGTAT	+	+	+	RS2CL4178s
256	CL4022	ATCCTCCAAAGAAGCAGCTCAC	TCGACAACTCCCACAATGATCT	-	-	-	-
	CL4034	GACAACAAGGTTTTAGCCGAGA	AAGCCCGTAAATCCACATCATC	-	-	-	-
	CL4099	ATCTGAAGCGTACTGAGCAACG	TGGCTTCACTAAACGCTACTGC	+	+	-	-
	CL4120	AGGAAGATAGGTTCGGCATGAA	GGCAATACTTCTGCCTCCATTT	+	+	-	-
	CL4165	AGTGAAAGAGCAGGACTGCAAC	TTACTAGCCGGAACGTTCACAA	+	+	-	-
	CL4190	GGGATATAAGAAGCGGGACAGA	AGAAGAGGTCTGACGAATCAGC	+	+	-	-
257	CL4033	ATCTCGATGTCCAGAGCTCCAT	CAGCAGAAGTTGTTCTCCATGA	+	+	+	RS2CL4033s
	CL4059	AGGTGTACAACGCAGAGCCTAA	CGTTCACAGAACTGGTCCAAAG	+	+	+	RS2CL4059s

	CL4107	AGTTTCAATTCCGCGTCTCTCT	TCTTGACCAGCAGTATCCCAAA	+	+	-	-
	CL4118	TGTCTTCCATGTGAATCCAACC	TGAATCTCTCACGCAGGTTTTC	-	+	-	-
	CL4119	ATCCAGCTTAGGCACACTTGCT	CGCGTCTCTCGTCTCTAAATCA	+	+	+	RS2CL4119s
	CL4161	AGCTCCTCTCACCTCCAAATCA	GAAAAGTCTCCCTCTTGCTGCT	+	+	+	RS2CL4161s
258	CL4201	TCTTTCCTACTCCTCGCAACTC	CGTATGATTCAGAGCAGCATCC	-	-	-	-
	CL4233	TTCTTGTCTTCGTGCGCATT	TTTTGGGCAACACCCTTCAT	+	+	-	-
	CL4248	GAAACGTGTTCTTGAAGCATGG	ATCGAGAGCGCTAGCTTTGTGT	+	+	+	RS2CL4248s
	CL4265	ACTTGGTACTCCGGCTCACATT	CCACAGTCGTATTTCTGTCCAA	+	+	-	-
	CL4267	CATCAAAGCCACACACAGATCC	GTTGGTGATCTCGCAACTCAAG	-	-	-	-
	CL4289	ATTCTCCGACCGTATGTTCCAC	AACCTAGGAAGCATCCCAACAA	+	+	-	-
259	CL4205	CCTTACATTCAAACCGCTCCAC	GCCATAATAGCCATCGAAGTGA	-	-	-	-
	CL4212	GGGTCATTCCCTCCATATTCTT	ATTACAGAACCTTCCGGTTGGA	-	-	-	-
	CL4308	ACAAACCCGTCTGAAGAGGAAC	GATGATGGTGAGCACAGTGTTG	+	+	-	-
	CL4326	AAAGAAGCCTTGCTTCCTCTGA	GACATGGCACATACAATCAGGA	+	-	-	-
	CL4349	TGGAAAAAACGCTATAGCAGCAG	CTCAGCTCCTCGTTCTCCATTT	-	-	-	-
	CL4386	ACAACTTGTGTGATTGGGAACG	TCCAACATTGTAGCCTGACCAC	+	+	-	-
260	CL4224	TCCCCAGCTATCACATTTTCCT	GTGGACGAGAATTCCAGCTTTA	+	+	+	RS2CL4224s
	CL4236	GCAACATCACCAACATCATCAG	TTAGCCAGTTCCACCAAGAACA	+	+	-	-
	CL4311	TAATGTCGAACAACGGATCGAG	AAGACATCCACGGGGTTAGAGA	+	+	+	RS2CL4311s
	CL4376	AAATGGTTGCATCTGCGTTC	TATGCTTCCAGTCAATGCACCT	+	+	+	RS2CL4376s
	CL4391	TCATCAAACAACGACGACGAC	TGCCTCTCTTCTCTTCTTGACC	+	+	-	-
	CL4397	GCTCACGAAGCTTTACGACATC	GATCATCCGGAGAAAAGCAAAC	+	+	-	-
261	CL4193	GAATGTCGTCCACTCTCCATGA	AACACACGCCGTACCAACTTTT	-	-	-	-
	CL4242	TCTCGAAGAGAAGCTGAAATCG	CCCCACAAGCAAACCACTTTAT	+	+	-	-
	CL4264	GCGACTTCACTTGGGATCTTCT	CGCTTGGTATTGAAACACTTGG	+	+	-	-
	CL4282	ACTAAACCCTGGTGGTGTTTCC	CATCAGATCAGCCATCATGACA	+	+	-	-
	CL4286	GGAGTTCACTCTGGAACAGCAA	ACCATCATCATCTTGCAGCAGT	+	+	-	-
	CL4299	TTCGCTAGGCCTGACTCATCTT	TTCTTGGACCTCTCTTCCTCCA	-	-	-	-
262	CL4219	CTTTGGCCCTTTCAACATGAC	ATTGTAGGAAATGCGGAACTGG	+	+	-	-
	CL4229	AAAGCGACGTCGTTCTCATAGC	CAGCTTCGAACTTTGCAGACAC	+	+	+	RS2CL4229s
	CL4281	GCATCGATGGAATCTACTGTGC	CGGAAAAGGAGCGAATGACTTA	-	-	-	-
	CL4285	GGTTCCAGGGGGAATAAGGATA	GTCACAGGCAACCTGATCAAAC	+	+	+	RS2CL4285s
	CL4307	AGGAGGAACTGAGCTGATGGAT	AGAGAAGGTGGGGAAACTGTTG	+	+	+	RS2CL4307s
	CL4339	CTTTGTGTTTCCGTTTCTGTGC	AAGGCAGGGATCAACTTCAAGA	+	+	-	-
263	CL4412	ACTCCGGACATTTTCTTCCTCA	CGGTAAAATCCTGGAGCAATCT	-	-	-	-
	CL4445	TCGAATGAGGTGAGATTGGAGA	CTCCTAAAAACGCTGTTGCAGA	+	+	-	-
	CL4523	AATGAATTCTTCTGGGGTGCTC	CTTGCTCTTGTCACCAAGCTCT	+	+	-	-
	CL4593	AGCATCTCGAAGATCCACTTCC	CATTTTCCCTCTTTCCCCTTTC	+	+	+	RS2CL4593s
	CL4676	AGCCGAGATCGATTGTGTGATA	GCATGTAGCGAGTTGGTCCTAT	+	+	-	-
	CL4701	AAAGGTGCGTTTACGGTAGCAG	ACTCCTGTGAGGCCAAAACAA	+	+	+	RS2CL4701s
264	CL4453	ACCTTACTTGGCTTCCATCGAC	GGCAGCTTTAGCTTCCAGACTT	+	+	-	-
	CL4464	ATGCAATCGCATTCTCTGGTC	AGCAACCCCAAAGATTTGTGAC	+	+	-	-
	CL4474	TTAAAGGGCTTGGTGAAGAAGG	GGCCTTGAATCATCTCACCTTT	-	+	-	-
	CL4521	GGCTATCGGTTCTTTCGATCTG	CTCCCATCTTTACCCAAAACCA	+	+	+	RS2CL4521s
	CL4707	TAGACGCAGCTCTCTTGCAATC	CACGTCCCACACGTTAACAATC	+	+	-	-
	CL4752	CCAAAGTGGTTCAACGAGTCAA	CACCACCAGAACTACGTTCCAT	+	+	-	-
265	CL4441	GGAAAGGACACGACTTTGAGGT	AGACTCCGCTTCTCATCTTTCC	+	+	-	-
	CL4442	CCAATCGTTCACCGTGATCTTA	ACACGTTTCTTGCGTTACAAGC	+	+	-	-
	CL4585	GACATTGAACCCAATGGTGCTA	GGTTCGAAGCCTATCTCACACA	+	+	+	RS2CL4585s
	CL4588	TAGACGTCCGTTTCCCTTTCTG	CACAATATCTGCAATCCGGTGT	+	+	-	-
	CL4596	TGGAGAGCTACTCAAGGGCATT	GCCTTTTGATGGACTGATGTTG	+	+	-	-
	CL4609	ATCTACCATGGAGGTCGAGAGA	ACCTCCCTGTAATCTGGAGGAT	-	-	-	-
266	CL4436	GAACGAGGCTATTTCAGCCAAA	TGTCGGCCGAGTACTGATTAAG	+	+	+	RS2CL4436s
	CL4471	CAAGACTCATGAACACGCACAT	CCATCCTCCAACATCTCCTTTT	+	+	-	-
	CL4489	GCTATGCCCTTCCTCATGCTAT	CGGTGATCTCTTTGCTCATACG	+	+	+	RS2CL4489s
	CL4624	GCCGTACCTACCGTTTTTACGA	GAAGCTTGAAGCGAGACGGTAT	+	+	+	RS2CL4624s
	CL4680	ATGGGAACAAGCCAGCAATAGT	TGGTCTCAGGTTTGAGGATTCA	+	+	+	RS2CL4680s
	CL4729	GCGAGTTTCGAGTTCCTGTCTT	ATCCAACTTGCCACACATCAAC	+	+	+	RS2CL4729s
267	CL4556	TCTTGAAGCAGAACAAGGCAGT	TCTTGAAACGTTCCTCGAGCTT	+	+	+	RS2CL4556s
	CL4557	ACCTTCTCTCCGCCTATGAAGA	GTGTCATCATCGCTACCGTCTA	-	-	-	-
	CL4579	CAACTTACGGTGAGGACTTTCG	AAGGGGCTTACAACTTGGTCAT	+	+	+	RS2CL4579s
	CL4635	GTTCACGGAAACCTCAAATCCT	CATTCCGAATTTATCCCTGTCC	-	-	-	-
	CL4639	CGCCAAGAAATCAACTTCCTTC	CCTTCCTCAACTCCTTGGAACA	+	+	-	-
	CL4653	GCGTTCAGAGCAAAACACAAAC	TAGAGAACGAGCTCACACCTCA	+	+	-	-
268	CL4424	CTGCAGCTAACGAAGCAAAGAA	TACGTGTGTCCTTCTTGCCAAT	+	+	-	-
	CL4493	ACCGAAGGAACAAAGAGACTCG	CCAAATCACGAGGACTGACAAC	+	+	+	RS2CL4493s
	CL4543	TAGCCAACACGTTGGTGATGTT	GATAAAACGCATCAGCGATCTG	+	+	-	-
	CL4567	AGTATCTTATGGCGGACGAGGA	CCAACTCTTCCACCGATTTCTC	+	+	+	RS2CL4567s
	CL4657	GGAGTCAGGTTAAACCCAAACG	AAGAGAATCTCGCACCACACTG	-	-	-	-
	CL4688	ATCATCTAGAACAGGCCCGAAA	GGGTCACGATCACACTTCTTCA	+	+	-	-

269	CL4825	TTCTCAAGTGACGGTGAAAGGA	GATTCTCGTCGCTGAACAGTTG	+	+	-	-
	CL4841	AAGCTTCTCTCCCATGTGGAAC	CGCTAGGATCAGAAGGAGCAAT	-	-	-	-
	CL4855	GATGAAACTGCTCGTGGAAATG	AGAACACTTGGTCGTCTGTCCA	-	-	-	-
	CL4882	ACGTTGTTTGTGGCGGTAGTTA	CGGTTTAAACGTGGTGTGTCTC	+	+	-	-
	CL4891	TTCGTCCTCTGCTTCGTACTCA	CAGTTGTATCCACCAACGGAGA	+	+	+	RS2CL4891s
	CL4951	ACCAGAGTCATTTGGGTCGTTT	AGCTTGGCTGCTTGAATCTTTC	+	+	-	-
270	CL4773	TGCAGTGTAGTAGCAGCATCCA	TAGGATTCGCATAAGCTCAACG	+	+	-	-
	CL4813	CCCAAAAGCTGATCCCTGTAAT	AATGTATCCGCCGTCATCAACT	-	-	-	-
	CL4917	CCTGAGTTCTGGGATTTGTCTG	ACGTGGGAAACAAGGAAGAAAG	-	-	-	-
	CL4941	CCTGGTTCGATGGAAGAAGAAG	TATCAGAGATGGGAACCAGTCC	+	+	-	-
	CL4970	ACAACTTCAATGGTGGTGGTGA	ACCCATTGCTTCCTGAGACTGT	-	-	-	-
	CL4985	CCTCCAAGCTCACAAACTCTCT	GGAAAGGGGGTCTAGGTTTGAA	+	+	+	RS2CL4985s
271	CL4789	AGATGGTCCAGGAAGCAGAAAG	GTAATCCATGCGTGTCTCAACC	+	+	+	RS2CL4789s
	CL4794	GCAGTGAAATTCCGTTCTGTTG	AGCAGCCTTTTGAAGAGCAGAT	+	+	-	-
	CL4935	ACATTGGCCTGACTTCTCTTCC	GCTATCGCCGGAGTTGTTACTT	+	+	-	-
	CL4956	TGCAGACACCACGGATAGAGAT	AGTCTCGCAATTGCCTAGCTTC	+	+	-	-
	CL4975	GGGGATTGATGTGTTCCAGAAT	TACAAATGGCGGTCGATAGAGA	+	+	+	RS2CL4975s
	CL5065	AACCTGAGCAGCAACTTCAGTG	GGGACAGAGTTAACCGAAAGGA	+	+	-	-
272	CL4868	TGACATCACCAAGTACGATGGA	CAAGGGCCTTAAGAAAGAGCTG	+	+	+	RS2CL4868s
	CL4931	GTTCCATTCCCAAAGAACCAAC	GCACACTGCAGGAACACCTTTA	+	+	-	-
	CL4952	ATAGCGGAAGAGAGCGTTTCAC	TCCCAATGCTATCCACTCACTG	+	+	+	RS2CL4952s
	CL4960	TGGTCGTCTTGAAGTCCATGAG	CATAAGGGTTTGTTGTCGTTGC	+	+	+	RS2CL4960s
	CL5027	ATCTTGGAGTTGGCAGAGAACG	GGATTGACCGAATTGAAGCTCT	+	+	-	-
	CL5060	CATAGCAGTCCATAACGCAAGC	CACCACCCGAGTCAAGAAAGTA	-	-	-	-
273	CL4777	GGGTACTTCCACCACCACATTT	TCACCAGCTCCGATATCTTCAG	+	+	-	-
	CL4845	ATCGAAAGCAAAGGGCTAAACC	TGTCAACGCAAGCAAACATC	-	-	-	-
	CL4918	ATGTGTTGGACCCAGGTCTCTT	GCGTAAGAAGTCACCATCAACG	+	+	-	-
	CL5031	ACTCCGGGAAGTGTTAGCAAAG	CGCTTCATCACATGAGTCATCA	-	-	-	-
	CL5033	TGCCATCTGATATTCGTCAAGC	TCTCTCTTCCGAGACTTGTCCA	+	+	-	-
	CL5066	TTGGTGATTGCTACGCAAGAAG	TAAAGCTTTGTGTCGAGGGTCA	+	+	+	RS2CL5066s
274	CL4809	GGCGCCTTATGTGAAGAAAGAC	CTGTGGTTCCCCATTTTCATCT	-	-	-	-
	CL4874	ACTGAAGAGGAGCACGCTTTTT	AAGCAAAACCGAACCTCTCAAG	+	+	+	RS2CL4974s
	CL4933	CTTGAAACTCTGTGTGGCCAAG	AACTGTTTGACGCCAGATACGA	+	+	+	RS2CL4933s
	CL4947	CTAATGGATTCATGCCCCTTTC	GAACAAATTTCTGCAGCACACC	-	-	-	-
	CL5017	CTCGCTTGTCCGAGATCCTATT	TGGTCGAGAAACGACAAGTACG	+	+	-	-
	CL5071	GTTTCATCACCAGCAGCAACTC	AGCATCCAATAACCGAGAGAGC	+	+	+	RS2CL5071s
275	CL5085	CCTCGGTTCTTCTCATCCAAGT	GATCAACCGGTTTAGGAACCTC	+	+	-	-
	CL5126	TTCTTCGCCAGCAACAAGTG	TCGAAAGGAAACGTCAACACTG	+	+	-	-
	CL5127	ACGGTTTCGAGAAAGAAGTGCT	ACAATCAGGACCCCAAAGAAGA	+	+	+	RS2CL5127s
	CL5174	TAGTCGACGATTTCATGGGAAG	CACTCATTTCACCACTCAACCA	+	+	-	-
	CL5197	ATCGCAATCTTCTTCGTTCGTC	TTAACACGGGCATGGAGCTT	+	+	+	RS2CL5197s
	CL5203	GAAGGCAGGGGTAAAAGGACTA	TGCAAGCTACTGGGATTTCATC	+	+	+	RS2CL5203s
276	CL5094	TGTTAGCAGACCCCAAGTTCCT	ACAGCAGCCGTCTTAAGCATAG	+	+	+	RS2CL5094s
	CL5101	GAAGCCGAGATGAGAGAAGTGA	TCCGTTTTCCTACTCCAACAGA	+	+	-	-
	CL5186	GCTGGAGCTGAAGTGGTTGAAT	GCTTTTTGCCTGCCACTAACAT	+	+	+	RS2CL5186s
	CL5211	GGCTCGTGGAGAGTATTTTGGT	GAAAAGGTACCAAAGCGCAAAC	-	+	-	-
	CL5218	CATTGAGATGGTTCGGTTTGTC	GAAGACTGCCACCGATGATTTT	+	+	-	-
	CL5252	CCAAAGCTCTGTCCCATTAACA	CTCGCGTGATAAGCATCAAAAC	-	+	-	-
277	CL5086	ACAAAGTTCCACCGGAAACCTA	TTCACTTGTCGCATTTCTCCAG	+	+	-	-
	CL5172	TCCCATATCGAAGGCAAGCTAT	AACGCATACACTTGTCCCCAGT	-	-	-	-
	CL5212	GAAGATGCTTGAATACGCACCA	GTTGAAGACTCCTTCGGATCGT	+	+	-	-
	CL5213	CAACGACTATGCCGACTTTGAC	TATAAACACAGCCAAGCGTCGT	+	+	-	-
	CL5232	ATGTCGTTAGGGTTCGCAAAAG	GGTGATATCCTGATGACCGATG	+	-	-	-
	CL5288	GTTCAACTCCAGGGGAGATGTT	CTCCATAATCCCACCATCAAAG	+	+	+	RS2CL5288s
278	CL5090	TGATTCACCTGAGGATGCTGTT	GTTGAAATCCTGCACTGCTTTG	+	+	-	-
	CL5102	AATGGTCGGCTTCATTGGTATC	AGTAAACAGAGCACACCGCAGA	+	+	-	-
	CL5209	TGGTTGTGCAAGCAAGAAGC	TACTGCTATCAAACACCGTTGG	+	+	+	RS2CL5209s
	CL5269	GTGCCTCGATTCGATTCAGTT	TTGGAGCTTATCGATTCCCTGT	+	-	-	-
	CL5270	TTTCTAAGCGCGTGTTCTTCAG	AGCGACGTTCTTGGACATGTTA	+	+	+	RS2CL5270s
	CL5291	AAAAACCGATCCCGACACACT	GGTTTCTCATCTGTTCCTGCAA	+	+	+	RS2CL5291s
279	CL5118	CTGCTGAAGCCAAAGGATTGA	TCATGTCACAAGCAAAGGAAGC	+	+	+	RS2CL5118s
	CL5132	ATGTTCAGTGTGCCTTGGTGAC	CTTTACAGGACCCAACACAAGC	-	-	-	-
	CL5140	CCAATGTCTTCTCACGTTTTCG	CTCCCTCCTCGTTTATCATTCA	+	+	-	-
	CL5198	TTTGAAGAAGGAAGGCTGTGTG	GAACTTCCATCTCTCCGAAAGG	+	+	+	RS2CL5198s
	CL5254	TCCCATGTTCCCTAACAAAACC	ACCCGAGAATGCTCACTCTTTC	+	+	+	RS2CL5254s
	CL5289	ACTGTCTCCATGAACCATCACC	AACATGACCCTCTTGAGACACG	+	+	-	-
280	CL5073	AGCAGCCAGAATAGCAATAGCC	ATATTCGTTGCGGCTTATCCAG	+	+	-	-
	CL5146	AGCAACAGTTTCACAGGCCTTA	GACCTTGAAGATGAAGCATCCA	-	-	-	-
	CL5153	CACAAACGGTGTATCGGTATTG	CGCATAGATAATCTCCCGAACC	-	-	-	-
	CL5207	CGGTAAGGAGAAGAAGCCTGAA	AAAGCAACTGCGACTTGGAACT	+	+	+	RS2CL5207s

	CL5217	ACACTTCTTACACCCGACAGCA	AGCAGACATGCAACACACACAC	+	+	+	RS2CL5217s
	CL5231	AACACGTAGTTTGCGTGCCTTA	CGACGAAGCGATGGTAGTGTAA	+	+	-	-
281	CL5092	ATCTTTCGGGGTGGTGGATT	AGAGAGCCTAAATGGCTGGAGA	+	+	+	RS2CL5092s
	CL5152	GACGTGGCTGCGATTATGTACT	ATCAACATGAGGAAGAGCCACA	-	-	-	-
	CL5214	GCCGTATTCACAACAAGCAATG	AATGGTCCGAAGCAAGAAACAC	+	+	-	-
	CL5215	TCAGGACATGGGCTTTTGACTA	AAACTCCCCTTTGCCGTTATCT	+	+	-	-
	CL5226	AAGATCGCTGGGATCAAGATGT	AATCATAAGTCTGGCGCAAACC	+	+	+	RS2CL5226s
	CL5276	TGAGAACGTAGGAGGCAAAATG	ACTCGTGAGCAACCACAACAAC	+	+	+	RS2CL5276s
282	CL5318	TTATATGCTCCCAGGTTGGTGA	ACTTTGAGAATCCAACGGAAGC	-	-	-	-
	CL5401	ACATCAGAACGTGGAACAATGC	TTGAAACCGAGAAGAGCTGGAT	+	+	+	RS2CL5401s
	CL5432	TCAGGCTTCGTCGAGTACATTC	AGCTCTCATAGCAACACCATCG	+	+	+	RS2CL5432s
	CL5474	ACGTACAAGCGGAGAATGTGAC	TAGACGAATTCATCCGCAAGAG	+	+	-	-
	CL5501	TCCGGAGACTCATCATCACTTC	ATCTTTTTGGGGATTCCCTAGC	-	+	-	-
	CL5518	AGAAAGCCACTTGGAGAAATCG	GATGATTCTTGCTCCTCCAAGG	-	-	-	-
283	CL5310	CAACGAGAATCCAGATGCTGAG	TTCAAGACCAGTCCCATAAGCA	+	-	-	-
	CL5344	GTCCACGTTTTTCCACCGTTAT	TCAAGAAGCAACCTCGAGAGAA	+	+	+	RS2CL5344s
	CL5368	TTCTCTGGACCATCCCTGTTCT	TATCCTTGGAAGGAGCAGAAGC	-	-	-	-
	CL5403	ATTGGAGCATTTCAAGCTACCG	TCCAGCTACGTCTCTGCAAGTA	-	-	-	-
	CL5452	TGCGTGCATACAGATAGTTCCA	TCTAACTCGGTCCAAATCTTCG	+	+	-	-
	CL5470	GCGACAGTTCTATCGTGAAACC	GACCAGAGTCGAAAACAACACC	+	+	+	RS2CL5470s
284	CL5313	ACAACTCGCCAAGCCTGTTAGT	TTGAGGAGGTGGTGATTCTCTG	+	+	+	RS2CL5313s
	CL5376	GCCTTGAAGCACTTTTACATGG	CATGACACACAAACATCCAACC	+	+	-	-
	CL5387	CCTTCTCTCGGCTAAGCAAGAA	TCAGGTTTTGGGGGTATATTGG	-	+	-	-
	CL5408	CGAGTAAGCAACTTCGTTGTGG	TCTTGTGATAAAACGCGTCAGC	+	+	-	-
	CL5422	ATCCTCTCTCCGATCATCAACC	ATCGGCGCATGTGATTGTTA	+	+	-	-
	CL5456	ATCTTCATGGTGGAAGCATCTG	GGAAACTTGATGTCGCCAAAG	+	+	-	-
285	CL5292	AGCCGTTGCAGAGTACCTTTCT	AACGTTTCCACAGGGATTGC	+	+	+	RS2CL5292s
	CL5298	TACTCGACAAATCCGGGAGAAT	CCTTTGGCCATTAGGTTGTGTT	+	+	+	RS2CL5298s
	CL5331	AAAGGAACTGGCAGAAACAAGC	TCCAACTGAGATTCAAGCTCCT	-	-	-	-
	CL5413	CAACGTCCGGCTGATAATGA	CATCAAATCTCCAAGGTTGCTC	+	+	-	-
	CL5490	CGTCCGTTTGCTTCTTCTTCTT	TGATGAAGTTTCCGGTGTTCTG	+	+	+	RS2CL5490s
	CL5516	ATTATCATCCACGGCGTCAAC	CATTCTCTGAACAAGCCCTTCA	+	+	-	-
286	CL5378	TCTTCCCGTGGAAAGGATATTG	ACCTTCAACACCTTCGTCCATT	+	+	+	RS2CL5378s
	CL5433	CCCATGAGAAAGAATCATCCAG	CGCTCTCTTTCTCTTTGTGTCG	+	+	+	RS2CL5433s
	CL5458	ATCCGAGTGTTACGGGTAAGGA	AAGACCCATCGTCTTGTTGGAC	+	+	-	-
	CL5472	ACCACAACCGTGGTAAATCAGA	TGCTTCTCCTCCACATGAATTG	+	-	-	-
	CL5504	GACAAACGGTATGACCCCAAAC	TCGCATCTATTGCTCTTTCAGC	+	+	+	RS2CL5504s
	CL5513	ATAGCACTAAACTGCCCCAAGC	TCACCAACCCAAAACTACATGC	+	+	-	-
287	CL5347	GGATGTCAAAATGCATCGGTAG	GGGCATTTTCTCTACCCCTTCT	+	+	-	-
	CL5366	AGTTAGTTCCCCCATGGCTTCT	CTCAAGGGCGTTTTCAGGTTT	-	+	-	-
	CL5372	AGTGTTCCAAGGAGAAGCGAAC	CTTCACTCTAACCGAACCACCA	+	+	-	-
	CL5425	GCCACATTTCAAAGACCAACCT	CATTTGAAAACTCTGCGTACCG	+	+	-	-
	CL5446	ATTCGAGAGCATCTGAGGATCA	TAACCCACTGAAGAGCCTAGCA	+	+	-	-
	CL5529	TGGCTGCCATGTAACAGAAACT	TGACTCAGCTGTGATGATCGTG	-	-	-	-
288	CL5573	ATGTGCATGGACAATCGCTTAG	CATTCCTTTGAGAGGGAGGCTA	+	+	-	-
	CL5691	GTCGTGGATATCAAAGCATCCA	TTTCTGGACCCGGTGAAAAG	+	+	-	-
	CL5699	TTAATGAGGCTGCTTGATGGAG	AGAAAGGATGCCGCTAGTTTTG	+	+	+	RS2CL5699s
	CL5774	GGTTGGATCATTCTCGCTTGT	GCTTGAGTTCGTGCCATAAAAC	+	+	-	-
	CL5787	GCACGGAGAAACAGCAATGAT	CTCCGATCTCTTCAATCTTCCA	+	+	-	-
	CL5805	CGTGGTTAGCACGGAAAACATA	CGGTGCAAAGTCATCCTAGAAG	+	+	+	RS2CL5805s
289	CL5575	TTACCATCACTCGAGCGTCTTC	GGCTTCCGGATAATCTTTAGGG	-	-	-	-
	CL5679	TACTTCACCGTTACAGGCGAGA	GAGGTGAAACTGGTGATGATGG	-	+	-	-
	CL5702	TTGCAATGTACTCCCAAAGGTC	CTTGAGCTTGGATTTGTCGATG	+	+	-	-
	CL5708	GCGCTTCGAATGAATCTCTCTT	AGCTGAATCACTTGCAGCTCCT	+	+	-	-
	CL5730	CGATGAAAAGCAACGATGAGAC	CATGGTTTTATACTCGCGGAAC	+	+	+	RS2CL5730s
	CL5790	GTGGAGAGATGAACGGTTTCCT	GACATTGTCTTCATCCCCTGGT	+	+	+	RS2CL5790s
290	CL5533	TCCATTGACTCCCCTTGTATCA	GAATGCTCTCTCTTTCCGCAGT	+	+	-	-
	CL5606	TGCAGTCGATTCTTGAGAAAGC	ATTGTCTCCACGATGCCTCTTC	-	-	-	-
	CL5615	TCTCCGAGGAAGAAACTCTCGT	TTGTGATGAAGCTTCTGGGACT	+	+	-	-
	CL5714	ACACCAGTGACGACAACACCTT	CCAGGTGGATAACAGCTAGGAA	-	-	-	-
	CL5728	CACAATAAGAAGCCATGGAGGA	ATAGCATCCACACACCATCGAC	+	+	+	RS2CL5728s
	CL5813	ATGGCAACCAGCTTACCAGTCT	AACCAAGTCCAAACTTGCCATC	+	+	+	RS2CL5813s
291	CL5574	TGGAATCTTTGTGGTCTGTGTG	CCCCCATTTCTTGATCCTAAAG	+	+	-	-
	CL5589	CGAAGAGCTGAAGATCTGCTTG	CGCGACGTTTTGGTATAGAAGA	-	-	-	-
	CL5598	CCCCAATACCCTATTTCCGTCT	TGGCTTCACAGATTGGGTCATA	+	+	-	-
	CL5642	ATCTCGAGCCATTGCGAGATAC	TCCCACCACCGGTTCATATT	+	+	-	-
	CL5646	CTTGAACCACCCCTCTGTTTTC	CACAACGAATGGCCTATTACCA	+	+	-	-
	CL5769	GGAGGCAAAACAATCCTTCATC	ATCAACGAAGTGAGCCCAGAA	-	-	-	-
292	CL5532	GATGGGAGAAGCTCCAATGAGA	ACCACCATGTTCACTAGCCACA	-	-	-	-
	CL5667	ATGTCCGAAGAACAAAGGAGGA	GGATGTATTCACACCACCGTGA	+	+	+	RS2CL5667s

	CL5681	TCAACTACGACAAGAGCCGAAA	CGCTGTCGGTATTCCATCATTA	+	+	+	RS2CL5681s
	CL5717	ATTTCAACTGCGGCTTAAGGTC	GAAGCAAATCATGCAAGACTCC	+	+	-	-
	CL5754	AACCATTCCCCTCTCCTAAACC	AAAGGAACTCGGAGACTTCAGC	+	+	-	-
	CL5768	CCACAGCTTTGAAGTTGTCAGG	CCACAACCGAACCTTAACCCTA	+	+	+	RS2CL5768s
293	CL5535	GTGGATGATGTTCCCATACGAG	GACACGGTCGGTGCAATAGTTA	+	+	-	-
	CL5542	AAATGAGGAGGCGATGGAGATA	CACAACCCCACCATATCATCAC	+	+	-	-
	CL5593	TCCATCTCTTCACCCACTTCTG	TGTCGATTCCGACATGGTTATC	+	+	-	-
	CL5736	TGCGACTACGGAAACAAGAAGA	CATGGATCACAACCTTCCTGAG	+	+	+	RS2CL5736s
	CL5773	TGTTCAAGAACAGTTGGCGAAG	GTTCATCTTTCGTTGCGTTCTC	+	+	+	RS2CL5773s
	CL5783	GAACAACATGGGACGAGATTGA	TATGCGGCTAAGCACTCTTTTG	+	+	+	RS2CL5783s
294	CL5821	CAAAGTTTGGAAACTGGGAAGG	CTAAGGTTTGGTTTGGGCTTTG	+	+	-	-
	CL5856	GAAAGGAGCATTGGAAGGACAC	GTAATGGTGTGGCGGCATATAA	+	+	-	-
	CL5872	GTGATGAGAAGTTACGGGGACA	AGAGGAGATCGTTGAGGAATGC	-	-	-	-
	CL5889	GGCGAACAACAGTATCACCATC	GCTGACGCTGACTTTGAAAGAA	-	-	-	-
	CL5928	AAATTCACTCAAGGGTGCAAGG	CATTCCCGCTGAATAGGAAATC	+	+	+	RS2CL5928s
	CL6024	AAGACAGGCGTGAAAGGACTCT	GGAGGATTTGGAGCCATGTAAC	-	-	-	-
295	CL5849	GGGGTTCCTCAGATCAATGTGT	GAGGAAAAGGCAGATCACGTT	+	+	-	-
	CL5892	ACGCCATTCCTTCCTTGTCTAC	GACCGACATGTTGAAGGAACAG	+	+	+	RS2CL5892s
	CL5893	GTGTTTTCGACTGCGGTTGTT	ACATTGGACTCACCAACCAAAC	+	+	+	RS2CL5893s
	CL6006	AACCATCATCACCCTCTCCATC	CCCGAAGACAAGACTAGCGAAT	+	+	+	RS2CL6006s
	CL6014	AGCAAATGGCAGTGGCCTTA	GTATTGATCGGTGCATTTCTCG	+	+	-	-
	CL6078	GTACTACTTGCACGGGTTTTGG	TAGTTCTGTGGCTTGGTTTCCA	-	-	-	-
296	CL5945	TGTGATCCACCATACGGAGTTC	TACCAACCTGGCAAGAATCAGA	+	+	-	-
	CL5972	GGAACCATGGAAGCAAGAGAAA	CGATAGTTCCATCAAAGGCACA	+	+	+	RS2CL5972s
	CL5997	GCTTGGGAAAGCATTTTTGC	GATTCGGCCCAAAAACAAAG	-	-	-	-
	CL6027	TACCTTCCATCGGTGAGAACAG	CAGTCAGAAGAGGAACCCGAAT	+	+	+	RS2CL6027s
	CL6036	AGACAGGGGAACCCCAATAGTT	GCAGCCAAACATTGTCCACTT	+	+	-	-
	CL6141	ATCTCCCTCTCGCCAAACTCTT	CTCGTTGTGGATGGAGAAGATG	+	+	+	RS2CL6141s
297	CL5851	ATATGCAAAGGGACGGACTTGT	AAAAACCTGGGAAGTGTTGTGC	+	+	+	RS2CL5851s
	CL5880	CCATAAAGTCGCTGCTAAGCTC	TGAAGCTCATGACCCTCGTATG	+	+	-	-
	CL6009	TGTGAGCAAGGTTACCGTCTTG	TTACCATGGCTTCCTCATCTTG	+	+	+	RS2CL6009s
	CL6099	CGGTAGGTTATGGTTCCCGTTA	TCAGTACTGTCGTCACCGCTTT	-	-	-	-
	CL6108	AAACCAATCTTCCCCCTTCACT	CATCAGAGTAACCGCATCCAGA	-	+	-	-
	CL6126	GGTTTGTGTTTGAGACCGAGAA	AGACTGTTCAGACACCGAGCAA	+	+	+	RS2CL6126s
298	CL5848	GAGTCAAAAGAGTGCCCTGTCA	CGGTCATTGTTTTCTGCTCGTA	-	-	-	-
	CL5879	TGATGCTGAAAGACCTGGAGTC	ACCAGACTGTTTCTTCCCAAGC	+	+	-	-
	CL5907	ACTTTTTATCGTCCGGGATGTG	ACAAAAACCCCCAGGAAGAGAT	+	+	+	RS2CL5907s
	CL6032	CCTAACATGAGCTCGGTGAAGA	CATTTTAACTGCTTCGGGTTCC	-	-	-	-
	CL6090	ATCCGAACAAAGAAACGTGGAC	TCTTCGTCTTTAAACGGCTTCG	-	-	-	-
	CL6103	TCCCTAACTTCATCCTCCATCA	GAACTAGCGAATCTGTCGTGGA	+	+	-	-
299	CL5861	ACGCTCCGACTCAATAGCATCT	TTCGGACAACAAGATGAGGAGA	+	+	-	-
	CL5863	CAGATCAATCCGATGAACGTGT	CCAGTTGTGATGGTGTGAAGAA	+	+	+	RS2CL5863s
	CL5874	TCGCTTGACTCCAAACTCTCAA	ACAGACAGCTTCGCCAGTATGA	+	+	-	-
	CL5985	CGGTGAAATCGATTCGTAGC	AAACAGCGGTATATGCTTCCTG	+	+	+	RS2CL5985s
	CL6017	GGAATTGAGCCGACACAGTTTA	TCTCCCGTCCTGGTTTTTACTC	-	-	-	-
	CL6125	GTTCAACGATTCCCTCTGGA	CCTCATGATCTCAACTCCGTTC	+	+	-	-
300	CL6186	GCGTTTTGGAGATGAGCCTAGA	CAGTTTCCAGCATCGTTTGTTG	-	+	-	-
	CL6263	GTTCTGTTGCGTCGTTTCCA	TGGATCAAAGATGAAGGACTCG	+	+	+	RS2CL6263s
	CL6265	CCAACTCAACCAGAAGCTTTCA	TGGAAGTGGGCAAAGGATTACT	+	+	+	RS2CL6265s
	CL6300	CATGCAAAGACCCATCACAAGT	AAGCAAGTCATCGGGAATAGGA	+	+	-	-
	CL6346	TTCACCTTGAAAGGAGCCTCTG	CTGAGGAGGAAGAAGCACCAAA	+	+	+	RS2CL6346s
	CL6356	TCAGTACGGAAGACCGCTGTTA	TGCTAGTCGGGAAAGTGTCAAA	+	+	+	RS2CL6356s
301	CL6235	CTCGACATCGAATGCTTCTCAT	TCTTCTCCATCAGCTTCGACTG	+	+	-	-
	CL6317	CGGGGAAAGTGTATATGCAACA	GTATGACACCTCGGACATTTCG	-	-	-	-
	CL6332	TCATCTCTCCTTGCGTCTTCTG	GTAGCGGCAGACAAGAACTTCA	+	+	-	-
	CL6393	CTGAACCAACAAGCTTGGACAC	TTTCCTAGCGAGACAGATGCAA	+	+	+	RS2CL6393s
	CL6417	AATGCCTCCACAAGAAGAAAGC	TGGACTTGACCTTCCTTCATCA	+	+	-	-
	CL6419	CGCAAATCCTACCAAGACCTTC	ATCGAGGAGGTCTGGATTCTCA	-	-	-	-
302	CL6199	GAGGCATGAGACGCATTATGAA	AAACGCCATTACTTGGTAGTCG	+	+	-	-
	CL6206	TGCCACTCGTAAAGGTATGTGG	AGTCAACCACCTTTGCACATGA	-	-	-	-
	CL6303	ACTCGAGCCCTACTTGCATTGT	GGATTCTGGAACCTGAACCAAC	+	+	-	-
	CL6331	TTTGGATGCTGGTGTGAACAG	GCTGCAATATCCAAAAGCCTAC	+	+	+	RS2CL6331s
	CL6349	TATTCACTGCACATGGGTGACA	AATAGCCAGTAACGCCACCAAA	+	+	+	RS2CL6349s
	CL6413	GTGAAGGCTCAGATATGGGACA	GTTCCTTGCCCTGGAAGATTA	+	+	-	-
303	CL6183	CGATATCGATTGCCCTCTCTCT	ATGGTCAAAGGAACACCAGGTT	+	-	-	-
	CL6200	GGTTGGAAAGCAATTGGTGAAC	GGTTCGACACACAAAGAAACCA	-	-	-	-
	CL6286	ATGTCCGAAGAACAAAGGAGGA	CACCACAATTCGTACCCAAAAC	+	+	+	RS2CL6286s
	CL6320	GTCAGCAGGCATGGAAAAAGTA	ATAGAAGCGGAGGACATCAGGT	+	-	-	-
	CL6364	AAATACACTCAAGGGTGCAAGG	AGAGCTGCTCACTGTGGCTAAA	+	+	+	RS2CL6364s
	CL6367	GGCAAACACAAGGGAGGATTAC	ATGCGGTAATGCAAATGCTG	-	-	-	-

304	CL6151	GGACAAGATCGGGAAAAATGG	CTTCTCGACCGGAGTCTGATTT	+	+	+	RS2CL6151s
	CL6161	GCTTGACAAGGTTACATGCTGA	AAGAGCAAGTTAGCGGTGTTCA	+	+	-	-
	CL6218	CTCCGATAATCCTTCCATCGTC	ATCCTCTTCATTGGTTCGGGTA	+	+	+	RS2CL6218s
	CL6262	TCGTGTAAGGACTCGATCAACA	AAGCTTTGGCTCTAACCGGAGT	+	+	+	RS2CL6262s
	CL6389	TTTGAGAGGAACGAACTCACGA	CAAGCATGCCCTCTCTTCTTCT	+	+	-	-
	CL6395	GCTAGCATTGCAGAGCCTTCTT	CAGATGGTATATGGGACGCTCT	+	+	-	-
305	CL6179	TGTCCGAGTAACAGAGGAGGAA	ACATCACACTCCGTACCCAAAA	-	-	-	-
	CL6195	GCTGCTCTTGATCTTGGTCTCA	GTTGTGCTCATTGTCTCCTTGC	+	+	+	RS2CL6195s
	CL6247	GGTTTACCATTGGAGGAACCAG	AAGAGGTTGTTGTTCCGGGTAA	+	+	+	RS2CL6247s
	CL6306	TTCCTCGGGATCTCCTCAATAG	TCCTGTTTGACGTGCAGAAATC	+	+	+	RS2CL6306s
	CL6325	TCTCTCGACCGAAGACAGAGAA	TCCTCGCTAATCCCTTATGTCC	-	+	-	-
	CL6327	AACAGCCAACTTCATCTTCGTG	TCGAGCATATGACGAGCTTCTT	+	+	-	-
306	CL6438	GGAGGGTGTGTGTTTGATTCCT	AGAACTCCTCGTAGCTGTTGGA	+	+	+	RS2CL6438s
	CL6482	CGGAAAAGCCGAAGTTGTTTAC	TTCCACTCGCCTTTAACAGGAT	+	+	+	RS2CL6482s
	CL6557	CAAATACCAAAGTGGTGGGTCT	TGGCATATGGTATGGTTCACTG	-	+	-	-
	CL6590	GTCTTCATTGGAGCCTCTGGAT	ACCGAGGCTCTTTCTTCTATCG	+	+	-	-
	CL6596	CCGCTTTCAATTTCTCCAGGTA	ACAGCCTTTTTGATCTCCATCG	-	-	-	-
	CL6854	TTGGAGGAGCTGAAGAAGTTTG	TAGTTGCAAGGCAAGTTTGGTG	+	+	-	-
307	CL6453	ACCGATCTGATTCGAAGAAAGG	CTTGCGTCACACACATTCACAT	+	+	+	RS2CL6453s
	CL6460	CCAGGTCTCACCAACAACACAT	CTTCTTGGGTTGGAACTTGGTT	+	+	+	RS2CL6460s
	CL6469	GATGGCATGAACTTGGAAGGAT	AATGCTCCATACCATCCTCCTG	+	-	-	-
	CL6496	TGTGTCTCTGCATTCAAGTTCG	AGATAACTGGGCGTGCCAAT	-	-	-	-
	CL6533	TCGGCTGATATTAACTCCACCA	AGTCGACAGATCCTTTGCTTCC	+	+	-	-
	CL6853	GCAATCTGAGGTCGATGCTATG	TGCAACTCCGTCTCCTTATCAA	+	+	-	-
308	CL6481	GAGCCGTATCTCCCTTTGAATG	CTCCGAAACCTCGTCCATTATC	-	-	-	-
	CL6493	TGACTCGCCTTTGAAGACTTTG	CTTTCAACAGCACCATCTGAGC	+	+	+	RS2CL6493s
	CL6546	GCTGGCTCAACACAAGACCATA	TTTCTTCGCATACTCCTTCACC	+	+	-	-
	CL6559	CAAACCAATCTCATACCCACCA	TTGCTGACTTCAGGAGCAAGAG	+	+	+	RS2CL6559s
	CL6565	CATCCGTGCACAGACAGTTACA	AGAAGCAACCTCGAAAGAAGGA	+	+	-	-
	CL6859	GCCTTTTGACAACGGTAGAACC	TACCGAACAGCTCCAACTTTCC	+	+	-	-
309	CL6430	TCGAGGTACTAACTGGGAAGCA	TATAGCAAAGAAGACGCCGAGA	+	+	+	RS2CL6430s
	CL6454	AATGTTCCACTCGAGGCATGTA	GATGATCAGCATGGAGAAGGTC	+	+	+	RS2CL6454s
	CL6505	TTCTCTCGCGAACAAAGATAGC	CGCAAAGACAGAGAAAGCAGAG	+	+	-	-
	CL6521	ACAGCAGGAAACCACTTCACAA	AGGTCACTTGATGTGCGAAAAG	+	+	-	-
	CL6552	GTGTGAGAAGAAGCAACGCAGA	CTGCTACATTGTTGGCAGAAGC	-	-	-	-
	CL6603	TGGGAGGAGGATGATAAAGAGC	TTGTGATTGTCACACCTTGCAG	+	+	-	-
310	CL6429	CAAATAGAAAACCACCCACGAC	GGAAACCTGTTGCGTCGACTAT	+	+	+	RS2CL6429
	CL6432	AAGTCCAAACACATCTCCAAGG	CGACTGACAGGAGGAGTTTGAA	+	+	+	RS2CL6432s
	CL6459	AATCAACGGGGACAGAAACAGT	AAATTCCAGAGACTCGCACTCA	-	-	-	-
	CL6550	GTCTTCTCCGATCCGATTCTTC	GAGTAAGAGCCAACGCCATAGA	+	+	-	-
	CL6556	TCGCTGTGACTCACCAAACA	TTCATAGCCAGATCGGTCCAA	+	+	-	-
	CL6567	CGGTTCTGAAACCAGGATTCTT	CTGATGTAGCGTTTTGGATTGC	-	-	-	-
311	CL6872	CTGAAGAACTGGGTGAGGCATT	ACATCGAAAGACATAGCCAACG	+	-	-	-
	CL6944	TAAGGTGACCAGGAGCACAACT	GGGTTGCTAAGCTTTCTTCCAA	-	-	-	-
	CL6956	TGCCCAAGTTGGTAGGAGATTT	ATGGGAGAGTGGTTTGAAGGAG	+	+	+	RS2CL6956s
	CL6999	GAATGTAACGGGGACGAGATTC	AACCGTGAAGCTTCGAGAAAAC	-	-	-	-
	CL7007	TTGAAGGTTCTCTTGGGAACTG	AACGGCCTAGGATCCTTGAAA	-	-	-	-
	CL7011	AAGCGTAAAGATGCGGTCACTA	GTTTCGCCTTGTCGACTTCATT	+	+	+	RS2CL7011s
312	CL6869	GAGATCCCATGGCGAATAAAGA	AACGAACTTGGCCCAGAAAC	-	-	-	-
	CL6923	AAGGTGGATGGTAGATGTGGAC	GACATCACTTGACCATGAATCG	-	-	-	-
	CL6924	CCAAGGAAGAGGATTCTTCAGG	TGGTTGCTAGAAACAGCTCTCG	+	+	-	-
	CL6969	TTCTTCTCGGTCTCCTCTGCAT	GTGTTCTGTTCCATTGCCACTC	+	+	+	RS2CL6969s
	CL6979	TGTAGGTTTCCTCATCGCTTTG	CGAATCAGGCTCAAAACTCTCA	-	+	-	-
	CL7000	TTGATTGTGGAGAAAGCACCAG	TCGATTCTCACAGCTCCACTCT	-	-	-	-
313	CL6884	ACGACCAAGTTTCTAGCGAAGC	CCTTCTGTCTCCCACAAAAGAA	+	+	+	RS2CL6884s
	CL6885	AGGACACTCGATCAAACGCATA	TGTTGACATCGAGATCAGTTGG	+	+	-	-
	CL6937	GCATGCACCCTTTCGATTAGAT	CTCATTTAGGGCTTGGGCTTT	+	+	+	RS2CL6937s
	CL6948	AGTTAACGAGCAACCCTCCAAA	CGGAACTAACAAACGGAACAAC	+	+	+	RS2CL6948s
	CL6958	GAAACTGGCTGCTCCTTACGAT	GGATGACTGCAAAATGGTCTGA	+	+	+	RS2CL6958s
	CL6966	AGGCATTCTGGATGGTACTCGT	GAAGATGCATTCTTTGGGGAAG	+	+	-	-
314	CL6862	TAGAGAAGGGAAAGGCAGGTGA	AGTCATGTCTTGGTTTGGGTGA	+	+	+	RS2CL6862s
	CL6900	GAGCAGGAATCGATCTTGTCAG	GAAATGGCACTGAGAAATGCAC	-	-	-	-
	CL6905	TCGGAGGATTGTGGTTGTTATG	GAAAACGGCGAAGGATACTGAT	+	+	+	RS2CL6905s
	CL6940	CTCCAGAAGTTCGTTGAAGTGG	CCAGCACGCAGTCTTGAATTAC	+	+	-	-
	CL6990	CCATCGCTAAGCCCTTTCTTTA	TTCTTGATGAGGATGGACTGGA	-	-	-	-
	CL6992	GCGCGAGAGAACTTTCAAACAT	GGAGATGGGTTGCAGAGAAACT	+	+	+	RS2CL6992s
315	CL6863	TTCAATCTCAGTGCATGGTCCT	GCCCCAAGGAAAAACAGAAACT	-	-	-	-
	CL6920	AACCTGCATCGGGAAGTACA	CAACCAAAGCTAACATCCATCC	-	-	-	-
	CL6929	GCCAAGTGGAAGAAACAATGG	TCAGCTTCTTGAAGTCCATCCA	+	+	-	-
	CL7003	AACTCAACCGCTGCTTCACATA	CTGATTCCGGCATGTACGACTA	+	+	-	-

	CL7006	GGACAACACAAAAACCCTTTCC	CGGAGACGAAAACGAGCATA	+	+	-	-
	CL7023	TCCGTGTGGAATGCAAAGACTA	AAGAATTCACCACGAACGAACC	+	+	-	-
316	CL6874	GCGTGGAATTGACGAATCAGT	TGTGAATGTCTTGCGGTAACCT	+	+	+	RS2CL6874s
	CL6922	GGTTGGGAGTATTTCAGGTCCA	GAAAGGAATTGCAGGGGTTATG	+	+	-	-
	CL6947	CTCCAAGAACTCATCCCACAGA	CTGAGAAGGGAGATGCTCGAAT	-	-	-	-
	CL6952	GAAAGGGAAAGAAAGGAACGTG	AACCAGACTCAGTGTTGTGTCG	+	+	+	RS2CL6952s
	CL6953	AAAGGAGTGCTGAGTGATGCTG	TCAAAAGCTGGAGACATTGGTG	-	-	-	-
	CL6965	CCGCTTCTAAAATTCCTCTCCA	CGGAATCAATCTTGTTGCTACG	+	+	-	-
317	CL7024	CCACTTGGGCTAATGTGGTTTC	CTCGTGGACTCTTTTCCCATCT	-	-	-	-
	CL7037	GTTGAATATGGAAGCGGTTCAG	AATCTTGGTCAACGGTCCATCT	+	+	+	RS2CL7037s
	CL7080	AAGTAATGGCGACATCGGTTG	GAAAGCTTTTGACGGTCTTGC	+	+	-	-
	CL7086	AGTGTTTGGGGAAGAGAAGGTG	TCTGCTCGACGTTCTTTCTCAG	+	+	+	RS2CL7086s
	CL7180	GGTGCTGATGCTCAGGTTGATA	GTTGCTTAAGCTTTGCAGTGGA	-	-	-	-
	CL7183	CGATCCTAAAGGAGGCAGGATT	CAACGAAGCTTTGCCCTTATTC	-	-	-	-
318	CL7048	CATGCATAGGGGCCAAGTTT	TTTAGTTCCTTGGTTGCTGCTG	+	+	-	-
	CL7070	AAGAATTCCACACCATGGCTTC	CTCATCTTGTTCGTCGATTGCT	-	+	-	-
	CL7089	TTCCAGCCTTCTCTCGAAACTC	TTGTCCATACAAGGCAAGTCGT	+	+	+	RS2CL7089s
	CL7128	GAGGTTCGTTTGTTCTTGAGGA	GAATCAATGCAAAGTGCAGACC	+	+	+	RS2CL7128s
	CL7173	TCAGCATCTGTGTCATCCATTG	GATTATCTCGGAACAGGGCATT	+	+	+	RS2CL7173s
	CL7186	ATACAAGAGCCAGACGTGCAGA	CCTTAGCCGAAGCAATGAGAAT	-	-	-	-
319	CL7041	AAGCAGGTAAAGACTCCGTGCT	CAAGAACAAGGCCCAGAACACT	-	-	-	-
	CL7056	AGCTGAAGCCTTACATCCCTGA	AGTCAAGCTTAACCGGGTATCG	-	-	-	-
	CL7063	ATGAGGACTTACATGGGTCACG	TCTGGTTGTCCAAACTCTGTGC	+	+	+	RS2CL7963s
	CL7115	TTTGAACAAAGACGCGGACA	TCATGTCACCAGCGACAGTAAA	-	-	-	-
	CL7153	GTCCGAAAATTCAGAGCATGAG	TGAAGGTACTTCAAACCCTCCA	+	+	+	RS2CL7153s
	CL7164	TCTTGGGAGCTATGTGAAGCAA	CTTCATGGGTGCTTTTGATGAC	-	-	-	-
320	CL7033	AAGGAGAATCCTTCCGAAGCTC	TCAACAGACGGCTCAGACTCAT	+	-	-	-
	CL7040	TACATCTACGGGCAGCGTTCTA	CCTTCAGCAGCAAGAAACACTC	-	-	-	-
	CL7051	AACTTCTAGGGCGCCTTTAACA	GTGGAAGACGGTATCATCAACG	+	+	-	-
	CL7104	ACACGGTGGTGTATCTGCTTTG	ATTTTGAGTGGGTCCGATGC	-	+	-	-
	CL7134	CGAGTTTCCTCAAGACCTGCTT	GCTCTTAAACATAACGGCAGGA	-	+	-	-
	CL7149	GAGACGTGAAGCAAGCAACAAC	TCCATGTAATTGGCGATACCAG	+	+	+	RS2CL7149s
321	CL7046	ACCGGAGTTGAAGCCAGTTTT	CGACGATGCCATTGAAGATG	+	+	-	-
	CL7110	ACGTCGTGACGGATAAGATGAA	GCACGCTTTCGGATTAAACTTC	+	+	+	RS2CL7110s
	CL7163	AGGAAGCTGCAAGAGCTTACGA	TAGTTCCATAGGGTCTGCACCA	+	+	+	RS2CL7163s
	CL7167	ATCTCATGGGTCTCAAAACACC	TCGTCCCACAGATCTTTTCTCC	-	-	-	-
	CL7185	GTTTGAAGTAGCGAGGCCATT	GTAACATCGGCTCCAATTGCT	-	-	-	-
	CL7198	AAGACGACCAAGAGGAAGACGA	TCCAAAAGGACTGCCAAAAGAC	+	+	+	RS2CL7198s
322	CL7049	GGACTCACTTCCCCCAATAAGT	AGTGGAGAATCATCCTGACGAA	-	+	-	-
	CL7094	CCAACAACAGCTCAGAACCATC	CAGCTTTGAGCTTAAGCCATCC	+	+	+	RS2CL7094s
	CL7096	CCAGCCAAAAGCCAGAAGTAGT	TCATCTGGGTTCCACTCTACGA	+	+	-	-
	CL7114	ATCAATCTCCCCCAAATCCCTA	TATGAACATGTCCCCACAGAGC	+	+	-	-
	CL7140	CCGATGATGACTTCAAGGACAC	CTTCACAGCCTCTTCGGTTTCT	+	+	+	RS2CL7140s
	CL7144	GCTAATTTCTCTCGCGATCCAT	AACGACGATACACAATCCAACG	+	+	+	RS2CL7144s
323	CL7217	ACCGGAGAAGATGAAGGTGGTA	CAAACCCTCCTTCAACCTGAAC	+	+	+	RS2CL7217s
	CL7220	TGAGGTACTGCCATTCAGATCC	GGTGTGTTCTGTTTCGTTGGAG	-	-	-	-
	CL7231	TCCCATCCAAAATCTCGATCTC	GATGTCGGAGACATAAGGTCCA	+	+	+	RS2CL7231s
	CL7310	ACTTGGTGGGAGGAAGAAAACA	TATGAAGCAGAAGCGTTGAAGC	+	+	-	-
	CL7318	CACACTTGAGTTGTCCCATCTG	GGGGGCCAAGACTTTTTACAAT	+	+	-	-
	CL7321	GCGGACGATGTAGCTATTGATG	ACCAAACCTCAAAGTGTCACCA	-	-	-	-
324	CL7210	CCTCGTTCAAGTCAAGTTGTGG	AACAAGCATCCAGTCTCCTTCC	-	-	-	-
	CL7221	AATGTGACTGCGGATCATCGTA	GATTATCGCGGGAAAACCAAG	+	+	+	RS2CL7221s
	CL7234	CATTTCATCACTCCGCCAAG	TGTTCTTGAAGCCACGTTTAGG	+	+	+	RS2CL7234s
	CL7235	CCAGGAACACTTTGGGGTAAAC	GTCATGGACTGGTTTCTTCAGC	-	-	-	-
	CL7303	GGAATGTTCAGCAACAGCGTAT	AACACCACCGATAACCTTCGTC	+	+	-	-
	CL7314	CCACGAGATCTCAGCAAGAAGA	CTGTTTCCTAGCACGAGAACGA	+	+	+	RS2CL7314s
325	CL7207	TCTTAGGGGGCTCAATGTTCTG	GATCTGATGACTATGCCCCACA	+	+	+	RS2CL7207s
	CL7257	CCTTGATTATTACCCCCAAGCA	ACTTCGCCGAGGAGAATCATTA	-	-	-	-
	CL7244	GGGGTTTTGCAGAATCTCCTAT	CATGTATGGGTTTGGAGTGGTG	+	+	-	-
	CL7263	TTCGAAAAGACAGTGGTCCTCA	GAGGACCAATAAGCCCAACAAC	+	+	+	RS2CL7263s
	CL7329	TTGGTCTATTGGCTTTGGTGGT	CGATGGAGAGCTTCACGTTAGT	+	+	-	-
	CL7330	CAAGGAAACAGCCACAGAGAAA	ACCTAACGCTTCTGCTGCTTCT	+	-	-	-
326	CL7272	ATCATAGGACATCCCTGGACCT	ACGCAAAGCCTAGCATAAAAGC	+	+	+	RS2CL7272s
	CL7278	ATTCTAGCTGCCGTCGTCTCAT	ATGGCAGTGACCTCATCTTCAC	-	-	-	-
	CL7283	ATAAGTACTGCTGCGCTGTTGA	TACGCCTATAAAGGGGCGTTTT	-	-	-	-
	CL7316	CCAATCTCCAACGTCCTCTCTT	AAGCGCTATAACGGTTTCCAAG	+	+	+	RS2CL7316s
	CL7320	CACACAAACTCAGAACCGGAAC	ACGAATCAGCGTTATGTGTTGC	+	+	-	-
	CL7322	TTTCAAGGTGTTACACGGTGGT	TCAATTTGAGAGGGTCTGATGC	+	+	-	-
327	CL7368	GGAGATCCAGACAGAACCCTTT	AAGGCAGAAGAAGAGGACCAAG	+	+	+	RS2CL7268s
	CL7375	TAATCGAGATGGGCTCGGTTTA	TCGTTTATCGGTGTGTCGATTC	+	+	+	RS2CL7375s

	CL7456	AACATGTTGATGAGTGGCTTGC	TTCCGCTACTCTGGAGGAAGTT	+	+	+	RS2CL7456s
	CL7465	TCAGCACAAGCAAAGGTTGG	CCAATGGGCTGTTTTACAAGG	-	-	-	-
	CL7499	GGATCCAAAATCGGTGGAGTT	GATATTTTTCCCGACGGAGACC	-	-	-	-
	CL7568	ATTGCATCTCCTTCCACTCCAT	GCTTAGGCATTGCGTTTCTAGC	+	+	+	RS2CL7568s
328	CL7410	TCTTTCCACCTATGCCACTTCA	GGGTTAAACGTAAACCCAGACG	+	+	-	-
	CL7452	TGTTCCAACGAGACCAGAAGAG	AAGTTCACGGGACACAACTCAG	+	+	+	RS2CL7452s
	CL7457	TCGTTCCATAAGCGACTCCATA	GTTAATCCGATTGCCTGAGCTT	+	+	+	RS2CL7457s
	CL7475	CGGCTTCTAATAGGACCCTTGT	CTTGCTCTTGCACGATTGCTAC	+	+	-	-
	CL7588	TCTGATGAGCGGATAGAGATCG	AGCTTCAGACTCCTTGCTGCTT	+	+	-	-
	CL7603	AGCTGGAGATGGACCAGAAAAG	TCAGAGCTGTGGTCATTCTCCT	+	+	-	-
329	CL7383	AGTTCTTGACGAATTGCCTTCC	ACTCAAGCCCATCGACTACGTT	+	+	+	RS2CL7383s
	CL7426	TAGTCCCTTCTCCGATAGCACA	GATTCAAGGAGGTGGGATCAT	-	-	-	-
	CL7546	GGAGCTTGTGGCTATGGAAATC	CTTCGGAAAGAGACAGGGACAA	+	+	-	-
	CL7550	CCTCCATCACTAAATCCCCAGT	CAATGATGAGACGCTTTGAAGG	-	-	-	-
	CL7551	CTCATGAGCCAGAGTTGTCAAA	GTGTAAACCCACAACGAGCTGA	+	+	+	RS2CL7551s
	CL7570	TCTCGCAGAAGCTTTCGAATC	GCGTGCATGAACTCAAACAAGT	+	+	-	-
330	CL7347	TGAGTACGAGAGGAAGCAAAGC	GCTACATTGTTGGCAGAAACG	+	+	+	RS2CL7347s
	CL7369	AACGTGAAGGGGGTGTTTGAT	AGTGCAATATTTGGGTCTCAGC	+	+	+	RS2CL7369s
	CL7474	GGGGATCTCTCGATAGCTGAAT	CAGAGCGTGTTCAACCGTACTT	-	-	-	-
	CL7584	CAGCACAGACAACCGATGAAA	CAAAACTTCCTTCTGCGACACA	+	+	+	RS2CL7584s
	CL7593	AAGGCCACAAGGGTAACTTGAT	TATTTGAGGTGGGTCCCGTTAG	+	+	-	-
	CL7599	TTAACCAGCACGAGTGGTCTTC	ACTATCCCACAGCCACAACTGA	+	+	+	RS2CL7599s
331	CL7339	GAAGGCAAACTAACTCCCTCCA	CTTGGGAAGATCCTGACGAGTT	+	+	-	-
	CL7401	ATTCCACAACCGATCCTCCTAA	AAGCTTCACCTCTTCGTGCAAT	+	+	-	-
	CL7479	GATTGTTGGCGATACGACAAAG	AGATGGACACACCATCACCATC	-	+	-	-
	CL7484	CCATAAAAAGCTCACGCAGTTC	CGCTTCAAACTGTATCTGACCA	-	+	-	-
	CL7490	TGACACAAAGGAAAGTGCAAGG	CGATATCGATAGTTCCGCAGAC	-	-	-	-
	CL7525	CCCTTCAAAGATCATCACATGC	GGTGCTAAAACGAATGGGGTAG	+	+	-	-
332	CL7332	CATCATCATCACAGCCTTCTCA	CTGAGCGATCGTATCAAAAGGA	-	-	-	-
	CL7379	TCTCGTGTCGGGAGATTATTTG	CTGTCTGCATGAAACGAGGATA	+	+	-	-
	CL7437	GGTATGGTTGTGACCTTTGCAC	CCAGAACGTCTGTCAATCTTGG	+	+	+	RS2CL7437s
	CL7455	TCTGCAAGTAGGCGAGAACAGA	GTCATGTCGAAACCACAAGCTC	+	+	+	RS2CL7455s
	CL7513	GCAGCCTTGGTAACATGAAAGA	CGATTGCCTCAACAATTCCA	+	+	-	-
	CL7561	GCGATGGCGATAGCTTTTTAG	ATCCAGCCTCTCAAAGAACTCC	-	-	-	-
333	CL7750	CTCTTACGGCAAAGAATCAGCA	CGGTGAAACCAAATAGGAAGGA	+	+	-	-
	CL7752	CAAGATGGAAGGCAAGGAAGAA	GTGCAGTAGACAAGGGCAAAGA	-	-	-	-
	CL7787	CCTGATGGACAAGTCATCACAA	GCTGAGGGATGCAAGGATAGAT	+	+	-	-
	CL7841	TTAGCGAGAAGAGGTGGTGTCA	CCGACAATTTTGCAGAAGCA	-	-	-	-
	CL7919	CAGATGGACAGAGAACCATCCA	TACCCAGTGTAGCCACATCAAA	+	+	-	-
	CL7944	GTGCAGCTAAAGGGCTTGAGTA	TCAAGTTCTGTTCTTCGGTTGG	+	+	-	-
334	CL7647	GGGATCTTAGCGTTTCACAAAG	GCTTGCGGGTATTTGCTCAT	+	+	+	RS2CL7647s
	CL7652	CTCTCGATCTCTTGAGCGATTT	TTCCTAACTCCTGCTTCTCTGC	-	-	-	-
	CL7733	GACCAAAGGATAACCCACCAAA	CCTTTCGCTATCGTGAACTCGT	+	+	-	-
	CL7855	GCTTCTTCTCTCGCAGCTTCTT	CTTCTCCACTTGTTGCTCGTTC	+	+	-	-
	CL7960	CAAGCTCCCCAAGTTAACACAA	ACGGCTTCCGCTGTATCTCTAA	+	+	-	-
	CL7987	ACGATGATTCCCCTTGTCTCTC	TATCGAGTCCGCACAAAAAGAC	+	+	-	-
335	CL7656	CCTCTTTTCTTTGGCCACCATA	GATGCCATCAACGACTGTAGGA	+	+	-	-
	CL7699	TTTGGTCAAGAGGTTGAGTTCC	TTGGCCGAGTCTCTGAAGATAA	+	+	-	-
	CL7725	TTGGGACTCTCTCTGCAACTGT	GGCCGTGAAGCTGTTAGAAAGT	+	+	-	-
	CL7888	AGCGAATCCAAGGAACTGAAAG	CTAAATCCGGGTAAAGCTGTGG	+	-	-	-
	CL7920	TGTGGAGAAGCTACTGCAAAGG	TTTCACCCAAAAGGGTCCAG	+	+	-	-
	CL7928	CCAAATACCCACCCAAGAAGTG	AAGACCATGTGATGCAATGGAG	+	+	+	RS2CL7928s
336	CL7647	GGGATCTTAGCGTTTCACAAAG	GCTTGCGGGTATTTGCTCAT	+	+	+	RS2CL7647s
	CL7694	CTGCCTCATGGATCTTGTTCAG	AACTTCTCCCAGTTGCATTTCC	+	+	-	-
	CL7758	TTCCTAAGACGGTGTCTCAAGC	CTTCTTGATTCAGCTGCGTTTG	-	+	-	-
	CL7765	ACAAGGATGAAAGTTGCAGCAG	TTCTGTTCTCGAGTTGGTTTCG	-	-	-	-
	CL7812	ACTTTTGTGCGGTGGTTCTTTC	TGACAGATTTCCCCCAGGAGTA	+	+	-	-
	CL8002	ATCCGTTGTAGAAAGCACGACA	TCCTAACAGAACAGCCATCACG	+	+	-	-
337	CL7627	GGTGGATCTGGAAGAGAGTCAA	CGGAGAAACGAATTAGGGAGA	-	-	-	-
	CL7658	GAAATGTCCATGGCTGTTGGTA	CTGTTTGGCATCCATCAGCTT	+	+	-	-
	CL7673	ATTGGAACATCCGAGACGACTT	CATGATCGTGGTGGTTATCATC	+	-	-	-
	CL7794	GACTCTGCTTACAACCCAAGCA	TCCTCCAACACTGTAGCTTCCA	-	-	-	-
	CL7843	TTGACTTCCGGCTAATCGTAGA	TGAGGAAGTTGCCACAGTTTTG	+	+	-	-
	CL8000	CCCCCTTCAAGCAACAATCTAA	AAGCCACAGCTTCCAAGAAAAG	-	-	-	-
338	CL7685	CAAGGAGAGATTCATCGGAGGT	TCTCTTGAAAAGCAGAGCAACG	+	+	-	-
	CL7722	ACCTGTGCTTGAAGTTGTTCCA	GCTGGACGCTGAGTTCAGTAAT	+	+	+	RS2CL7722s
	CL7766	GTCGTTGTCGGAGATGGTAATG	AAGTTTCCGAGCGTTTTGGTAG	+	+	+	RS2CL7766s
	CL7785	GGGAGAGGTCAAATGCAGAAAC	TCAGAGTCAGCAACAGGGATGT	+	-	-	-
	CL7859	AGCTCCCCATTGTTTCATTCTC	GCCAAGAGATGAGAAAGGCTGT	+	+	-	-
	CL7983	TGATAGCAAAGCCATGGAAGAG	TACGTACCATTGCAGAAACGTG	-	-	-	-

339	CL8033	CTGCTGATGCCTACATCTTTGC	AACCAAGAATCACCGGTTTCAG	+	+	+	RS2CL8033
	CL8195	CGGCGAAGAAAGAAACTGTCTA	TGGCACCAGATTCTTTGACAAC	-	+	-	-
	CL8219	GAGTTGGCGAACATCTCGTATG	TCTTCAGCAACTTCTGCGGTTA	+	-	-	-
	CL8224	TTGAACCGATGAGCAAGAAGTG	TAAAGCGGGTGGTTACAGTTCC	+	+	-	-
	CL8252	TCAAGCTGTGCAAAGTGAGAAC	CAAGCGTGAACACATTCCCTAA	+	+	+	RS2CL8252s
	CL8267	AGCTACAAGACGCGTGATACGA	AAGCTCCGGGAAACTGTATTGA	+	+	-	RS2CL8267s
340	CL8024	ACGTAACCGAGTGGAAAGGAGA	CTGAGGCTAAAGCTGATGCAGA	+	+	+	RS2CL8024s
	CL8031	GCGTCTCCTGTAAAGGAAGGAT	GATACCAAGACCCGTGCATATC	+	+	-	-
	CL8055	TTTCTCTGTGTGGTTTGTGACG	CATCAGATGGACTGAAGCCAAG	-	-	-	-
	CL8123	GTTGGTGTTATGCGTGGCTAAG	AAGGTCACCATGAAACCATCG	+	+	+	RS2CL8123s
	CL8125	CCGAGCCAGATTACCTCAACAT	CAAATACCCAATCCAACGGTTC	+	+	+	RS2CL8125s
	CL8328	CAGAGCTTGGTCTGAAACAGGA	TTCTCTGTAACCATTCGGACCA	-	-	-	-
341	CL8043	TTGTGCTCATTACCGACTGAGG	CTACGCATGTTGGGAAAAGTCC	+	-	-	-
	CL8082	CTTCTTAAACCACCGAGCCAAC	GATGTCAAATTGCTTGGGGAAC	+	+	+	RS2CL8082s
	CL8112	AGGATGGTGATGATGCTTCAGA	AAGTCGTCATTGCCGTAGAACA	+	+	+	RS2CL8112s
	CL8226	TAAAACCCTTCCCTTCCTCTCC	TTGCTCAAAGAGCATAGCCAAG	+	+	-	-
	CL8245	CACGCCTGCAAAAATCTCAC	ATCTCCAGCCAGGCTTATTCTC	+	+	-	-
	CL8360	GGATGCGATTGTATCGTTGAAG	TACTCGGAGATGTCACGTCGAT	+	+	+	RS2CL8360s
342	CL8038	AGAGCGGGTTCCCTTACAAGAT	TAAACCTGATCAGACCCCAACA	-	-	-	-
	CL8062	CCCATAAAACCACCAACAACAG	AAGCTGAAGCAGAGGAGAAAGC	+	+	-	-
	CL8086	TGAGTCCTAACCGAACGCTAGA	TGGTGCCAAAGAGACTTGAGAA	-	-	-	-
	CL8108	GTGAGCTTGGTTTCGGACAGTA	AGGGAAGAGGTCGGAGAAAAAC	+	+	-	-
	CL8121	AGATTCATGGGTGACACGAAGA	AGATGCTGGTTCCTTGGATGAT	+	+	-	-
	CL8240	CATCAGACCTTGACCAAGATGG	ACATCAGTTCGGATTAGGATCG	+	+	-	-
343	CL8137	AACCTCCCTGTGACTTCCTTCA	ATATTGCCTTGACCACATGCAC	+	+	+	RS2CL8137s
	CL8145	CGGTCACAAAACACAAGCTTTC	ATCTACACTGTTCACCCGGACA	+	+	-	-
	CL8171	AATCCTGGCCTAAACCAGATCC	GTCTTGATCCTCACCAAGACCA	+	+	+	RS2CL8171s
	CL8223	CCCATTTTCCGTTCTTCACA	ATCGACAGTAGTTTTGCCGTTG	+	-	-	-
	CL8237	TGTGCACTGACAAGTTTTGACC	TGTCTGATGCACTTGCTCTCTT	+	+	+	RS2CL8237s
	CL8353	AAACTGCAAAGACGGGTTCAAG	CTTGCAATTGACGGTTAAGCAG	+	+	-	-
344	CL8131	TTCCGACGAGCTGTTCAGTCTA	TTTAGTGGAGGTCGGACACTTG	+	+	-	-
	CL8136	GAGATGCCAGTGGTTCTCATCA	AGGTGTTTTCCATGGCCTGA	+	+	-	-
	CL8162	TCCTTCTCCTCACCAATTCCTC	GTTGTGGACAACGACGGATAAA	+	+	-	-
	CL8250	CAAGGGAAAGAACTGTGCAATG	AGCGATTCCTTCATCATCCTTG	-	-	-	-
	CL8321	CCCACACACAAAAGAACGAAAG	CGGGTGGTTCACGAGAAAAT	+	+	-	-
	CL8362	ATCAAACCGTGACACCCTCCTA	TTGCTGGATCAAGATCAGGACT	+	+	-	-
345	CL8397	AATCAGGCTGTGATTGTTACGC	ATGGTGACAGCTCATGTTGAAG	+	+	+	RS2CL8397s
	CL8487	AGCGATTGGGACCAACCTAA	AAGCTTTGTCTTCGTTGTCGTC	+	+	-	-
	CL8541	GAGAAGAGCTTCCAAAGGCAAC	TCCTTGGTTTCACCCATCTCTT	+	+	-	-
	CL8543	ACCACGAGGAGCAGTTCAATCT	AAGCATTGCTGCATTGACAGAG	+	+	-	-
	CL8580	GAGACGGTGAGTTGGTTTCAGT	AGATTATTATGGCGACCTCACG	+	+	+	RS2CL8580s
	CL8618	TCAGACTGTGAAACTGCGAATG	GTGGGTTTTTGCCACAGGTAAG	-	+	-	-
346	CL8409	AGCCAAATGCATGTCCCTAAAC	CGCATCTTGTTGATCTGGTTG	-	-	-	-
	CL8433	GCAACAACGTCCATTCTCAAAC	AGCATAATCTTCAGCGATCACG	+	+	-	-
	CL8467	CAAACCGGTTCTGTGAAATCTG	CACGCCTCAGAATAGCAATCAA	+	+	+	RS2CL8467s
	CL8479	TCTAAAGGGCGGAGAGTTCTTG	TGTCTCAACTCCACTCCTTCCA	-	-	-	-
	CL8565	TTCAGTAACAAGGGCTCAGTCG	GCACTCGATGATCTGTTGAGGT	+	+	-	-
	CL8624	CCTTCGCACACCTATGACTTTG	ACCCCTTTGCAAACTCACATTC	+	+	-	-
347	CL8419	AAGCAACTACTGCACCAACCAC	ACAAATTCTGAGCCCGAGAGAC	+	+	+	RS2CL8419s
	CL8431	GACCCACCAAACAACACATCTC	CATATATGTCGAGGCGGTCGTA	-	+	-	-
	CL8474	TGGCTTTGCAAATACAGACCTC	CAAGGGATGAAACCAGAGGAAG	-	-	-	-
	CL8476	CAACGCGAGTAACATCACAACA	AAGATTGACCATCGGTGTAGCA	+	+	+	RS2CL8476s
	CL8549	CGAACTCAACGAAGGAGACAGA	CACAAGCTGACGAAGCTTGACT	+	+	-	-
	CL8557	GAAACCATACCGAGAGGAGGAA	TGCGTCTCTGAGCTTCTAACCA	+	+	-	-
348	CL8452	GGCGATTGTATACATTGCACAG	GCGAATAAAACTCGTCCCTGTC	-	-	-	-
	CL8499	GCTTTGACACACGCAACAACTT	TTGAATGATGTGGTCAGTGCAG	+	+	-	-
	CL8530	GTCTTCACAGGAGCAAGTTCCA	ATCAGCAGGTCTCTGGGAAAAA	+	+	-	-
	CL8548	TCGCAGACTCGTAGATCTTGGT	CCATTCAGGCTTGGATTACTGA	+	+	+	RS2CL8548s
	CL8592	CTGCAAACATATCCTCGGTTTC	ATGGATTGGTGTGGATAAGAGG	+	+	-	-
	CL8625	CGCCTTGTTTCTTACGACAATG	TAACCCAAACCGGTGTATCTGA	+	+	-	-
349	CL8378	TGACACAGGAGAAAAACGACCT	AGCGGTTGTTCTGTGGTATGTG	-	-	-	
	CL8475	CTTACGAGAAGGAGCGTCTTGA	TCTTTGTCGGATAAGGACACG	+	+	-	-
	CL8495	CCATGGCATGACAGGAACTTAG	ACGCTTTCCACCTGAGGATTAG	+	+	-	-
	CL8540	TGGTGGTACATCTCTGGCTACA	GTAAGCAACGCCATGAACTGAG	+	+	+	RS2CL8540s
	CL8586	TATCCAAAGAGTGGAGCGACTG	TCGGTAGATTTCGGCAGTGTT	+	+	-	-
	CL8626	CCGATCCCAAAATCAGGATCTA	GACGATGATCTTTTGACGACCA	+	+	-	-
350	CL8667	CCCGGAAAATTCTCAGCTTCTA	CATAACGTTGACGGTCTCTTCC	+	+	-	-
	CL8690	CGCTGCATATCCTTCTTTTCTG	AAGAGGCGAGAGATGAGCAAGT	+	+	-	-
	CL8774	TCACCTGATGTTCTTGCCAATC	CAACAGAAATGCCAAGTTCCAC	+	+	-	-
	CL8831	CAAATGGGTTTGGAGGAAGAAG	TCTTCTGCTCCTGTTTTCGATG	+	+	-	-

	CL8835	AACCAATCCAAAGCAACTCCTG	ACAATTGTTGGGATCTGGTAGG	+	+	+	RS2CL8835s
	CL8853	AACGTGAAGCCCAAGTGCTAAC	GCTTCACTCTGCAACAAGTCCA	-	-	-	-
351	CL8651	AAACGTGGGCCCTATTCAAGA	TGTTTTGTGAACCAGGGACTTG	+	+	-	-
	CL8713	GCTTTCCTCCTTTTCATTCGAC	TTTCTTCCATGAGGAGAACACG	-	-	-	-
	CL8738	ACAATGATGGCAATGGTGGTAG	TGCTTGATTTGACAGCTGAACC	+	+	-	-
	CL8794	TCCACATTCAGTGGGAACAAAC	CGTCGGTTCAAAACGTCTTTCT	+	+	-	-
	CL8800	TCAAAGCCACCGAGAAGGAT	ATGGTTGAAAAGCCAGTTGC	+	+	-	-
	CL8834	CCCCGGGGAAAAATAGAGAT	CAAAACCCCTTCCCAAACTGTA	+	+	+	RS2CL8834s
352	CL8631	CCAAACTTGTCACCACCTGAGA	CCTCTTCAGGACGCTTTTCTTC	-	-	-	-
	CL8665	TCAACAGAACATCCTTCCTCGT	TGTACATGTAAGCACCGCCTTC	+	-	-	-
	CL8689	CCTTCCTTCATTTGACGCTTG	GGAGTTTTCTTCTCTGCCTCCA	+	+	+	RS2CL8689s
	CL8709	TATCAATGAGAGCGACACATCG	GCTCCAACGTCATGCAATAGAT	+	+	+	RS2CL8709s
	CL8779	CAAGAAACATCCCAGGAGGACT	AACCAACACATCACACGACAGA	+	+	+	RS2CL8779s
	CL8832	TCAAACCGTACCCGGTTCTAGT	GGATGTCCGTTTTGGAGTTAGG	+	+	+	RS2CL8832s
353	CL8645	GTCACAACCGGAGCATTCTCTA	GGATCCTTCGTGCATCTTTGTA	+	+	+	RS2CL8645s
	CL8655	TTCTTGAAGCTCCTCTCCCAAG	CAAACGACCAGGTAACGGAGAT	+	+	-	-
	CL8659	TGCAAGTAACCTCAACGAGGAA	GCATAGAATCCAATCGACAGCA	-	-	-	-
	CL8673	TCTCTGAAGCGGTGTATTTCCA	ACTGGCTGAGACAAAAGATCCA	-	-	-	-
	CL8746	CAACCACAATTCCCAATCTCAC	AAGCCTCTGCCTACAACCCTAA	+	+	-	-
	CL8836	CCTTGTCGGCTTTCTTAATCCT	GCAAGAGCCATAACCATGAACA	-	-	-	-
354	CL8648	ATTTCGCCTTCTTCCTTCACAC	CATGTTGGTGCCGTACTCAAT	+	+	+	RS2CL8648s
	CL8762	TCGTCGTCAACGTAGATGGTCT	CAAAGAGCTCGAGATGCAAATG	+	+	-	-
	CL8797	CCGTCGTCTCCTCCTCAATTAT	TTCAACAACTTCCTGCAACTCC	+	+	+	RS2CL8797s
	CL8826	GATGCAGTTCTCGCATGATAGC	CCGGGGTTAGAGAAAGAGTTCA	-	-	-	-
	CL8839	ACGGGAGAGAATTTCAGTCGAT	GCATCGAAGTTATGTCGGAGAG	+	+	-	-
	CL8802	TAGGCCTCCACTTCTTCTTCCA	GAATCGTGTTCTTCCTTGCTTG	+	-	-	-
355	CL8644	GACATCCATTAGCCATCATTCG	TCGAGATCAAGAGAGACGATGC	+	-	-	-
	CL8683	CAGCAAGGCCTTTGGTAACTTT	CGTCCCTGGAAAAAGAGAAGAA	+	-	-	-
	CL8782	TCCAACTCTGCTTCTTCGATCA	AGAACCAAGCCTCTGAAGCAAG	-	-	-	-
	CL8817	TCTCGCTTACTTCCACAACAGG	AGTGGGGTTTCTCCTCAGATCA	+	+	+	RS2CL8817s
	CL8842	CGGTAGCAATAACGTGGAAATG	TTCTCTTCTGGCGGAACTTTGT	+	+	-	-
	CL8851	CCTTCAATAGCAGCAGCCAAA	GACCAGAGCTGCTCATGTTGTT	+	+	-	-
356	CL8904	CCTGTCAATCCCTTTCTGCAAT	CTTCCCAAAATCTTTCCTCTGC	-	-	-	-
	CL9025	GTTGATGTCGGTTTGTGCAAG	CACCACAACGGATCACATCA	+	+	+	RS2CL9025s
	CL9028	GAGGAAGCTGCTATTGCTGGAT	TGCTTCTTCCTCCTCAGGAAAC	+	+	+	RS2CL9028s
	CL675	GAGAAGAAGAAAGGAGGGCACA	AAAAGCACTTCCCACAAGGAGA	+	+	-	-
	CL678	ACTCGGAATCAGGAAACCCTTT	AGACTCGAGGCCAAGATTGAGA	-	-	-	-
	CL681	CATGGCCACAGAAACACAAAAC	CTTCAACGGAGACAACGTCAAC	+	+	+	RS2CL681s
357	CL8917	AAGCCACTTGCCTCGTGTTACT	AAGACGCTGTTGGCTTTCATCT	+	+	+	RS2CL8917s
	CL8966	AACCGTCGACTCATGTTGATTG	GTCCCTTGTCTTGCTTTTGGTT	+	+	-	-
	CL670	ATCGGTGCTTGAAGACGTGATA	TCATGTGTTGTTGAGCTGTGCT	+	-	-	-
	CL683	CTTGTATGTTCCTGTGGCCTTG	TACTGGAACTTGCGGTGTCTGT	+	+	+	RS2CL683s
	CL685	CTTTGTGCTCACAGTGGGTGAT	GCGTCACTCTCATGTTGTCCTT	+	+	+	RS2CL685s
	CL697	AACGCAGTGGTTGGAGATCTTT	GCTTCCTTGTCTGCTTCTCTCA	+	+	-	-
358	CL8939	ATGTGGTGTTCGGGCAAGTTAT	ACTGTTTGCGTCATTAGCAAGG	+	+	-	-
	CL8943	GAAGAATGTGGGGAAAGCAATC	AGTGAACATTTCAGGGTCTTGG	-	-	-	-
	CL672	GCGAGAAAGAGGAAGAAGACGA	AGACGATCCGTGAGAATCTCTG	+	+	-	-
	CL684	GGTTAGCGAATCGGAATCTCAG	CTTCAGCTTGAAGAATGGGAAG	+	+	+	RS2CL684s
	CL701	AGCAGAGAGCAGACAAACGAGA	GGCAATGGCATAAGCTCCTTTA	+	+	-	-
	CL706	CAGCTCCACAAAACTCAGCAAG	TTCCAGAGTTGTGCCACTTCAG	+	+	+	RS2CL706s
359	CL8931	GCAAGTTCGTCAGTGAACAAGA	GCGAACACAGACAAACCAGAGT	-	-	-	-
	CL8952	CCCTCAGCCCTTTCACATAA	GAAAAGAGGAGAGGAAGGACCA	+	+	+	RS2CL8952s
	CL9008	TCGTCTACCAACCAAACCAAGA	AAGAGCAGCGAGAAAGGAAGAA	+	+	-	-
	CL666	CGAAATGTGGCAAAGAAGGACT	ATCCCCACAAACTTTTGCTGTC	+	+	+	RS2CL666s
	CL677	AGTGCCGAAAAACTTGAAGCTC	TAGCATGGCCTAGCTTGTTCTG	-	-	-	-
	CL710	GGAGATTCGCCAATTCTCATTC	CGTCTTCACCGACCCATTATCT	+	+	-	-
360	CL8894	TTTCACAACCACCCCTAAAACC	CGATGTTCTTGAGGTGCCAAT	+	+	-	-
	CL8906	CATTCTAATCCACGGTCTGTGC	ACATCACACAACACAACCTCCA	+	+	+	RS2CL8906s
	CL9003	AACTGTGACATTGGAGGCATCT	GAGAGCTGATTTTGCCACTGAA	-	+	-	-
	CL9014	GCGGGATAGCCTTACACAAAAT	AGAATCATCCGAGTCCAAAACG	+	+	-	-
	CL9038	CGATGCAGGAAGAAGTGAAGAA	GTTGTTTCGTTCATCGCCTAGA	+	+	-	-
	CL698	CCGAAGCGTTTCCTTATCTCCT	CCAAGGATTGTCGCTCCTTAAA	+	+	-	-
361	CL8862	GATGGGTTCTTCTCCTGATTGC	TGGAACAGGTTCTGCTTCTTCA	+	+	-	-
	CL8865	TGCAGACCCAACTATTGGTCAC	ATCTCAGGGCTCTGAAAGCAAA	-	-	-	-
	CL9061	CTTGCTCTGTTTCCAGATGTCG	GGAACAAACAAAGACCCCAAAC	+	+	+	RS2CL9061s
	CL688	CCTCAATGTTGAGTCGGTTTTC	AAAACCAGTGCAGGCGAAAG	+	+	-	-
	CL690	CAAGCTCAAAAGGCTCCGTATC	GCAATCTTCTTGCCGTTCTTG	+	+	-	-
	CL694	CTTCAGTTGGTGACTTGGCATC	AAGGTTGAGTCGCTCTTGAAGG	+	+	+	RS2CL694s
362	CL748	GAAAGAACGGGACTTTCCACAC	ATGTTGAGGGATTCCTTCGGTA	+	+	-	-
	CL757	TTCTCCTTTATCCGAGGAGACG	ATGTTTGAGGATGAGGTGGTCA	+	+	-	-

	CL764	ATCTCTTGCGGATTCCACTCAT	CGGGATTACGAAAGAGCTTGAG	+	-	-	-
	CL767	GTTGGGATCAAGGTTTGCTAGG	GACAAATGGCCATAGACTGCTG	+	-	-	-
	CL778	TACCAAGAGCCAACACACTGGT	GGGAGATTTGTTTGTGGGTGAT	+	-	-	-
	CL795	GTGCTAAACGAGGCAAATCTGA	ACTGAAACTACGAGGCGTCACA	+	+	-	-
363	CL719	AAGAAGAAGAGGCCTCCCAGTT	GATCTTTTGCCTCTCTGGAGGA	-	+	-	-
	CL755	TGGAGATTGCGTCTGTGACTTT	CTCCTCGATAACAGAGCGTTGA	+	+	-	-
	CL763	AGCTCCCAATCACAGTCATTCA	TTCTTCTCTTGGGTTGCTGACA	-	-	-	-
	CL769	GAGGCTATGAGGAATGCTTTGG	GCTAGGCTTCCATGTTTGTTCA	-	-	-	-
	CL809	CTGCTTCCGTGGATCCATAGTT	AAGCAAATCCGGTCTTGATCTC	+	+	+	RS2CL809s
	CL812	CAGCATCAGCTGAAGGTTTCAC	AACCGACCCATATGAGAAGCAT	-	-	-	-
364	CL717	ATCCCTAGTTCCCCACATTCCT	CCTTTCCTTTTGATCGTCCTTG	-	-	-	-
	CL772	AACCGAACCAATGCGATAGTCT	AAAGCCTTGCATTTCCTCTCTG	+	+	-	-
	CL784	AAAAATGGCCTCAACAGCTCTC	GGCAAGAGTAAGGCAAGTCGAT	-	-	-	-
	CL773	AGGGTATCCTCCGGCAAGTTAT	CAAAGCAACAGCAAAGCATACC	+	+	-	-
	CL800	AGAGTTACAATGTCGGCATCCA	TAGCTGCTCCTCCTGTGGAATA	+	+	-	-
	CL806	ACATTGTCCTCGAGAACGTCAA	AGAAGCAAGCTCAACGAGAACC	+	+	+	RS2CL806s
365	CL715	CAAGTTCTTGTGGATGCTGGAG	CGAAGGTTGTAGAAAGCAATGG	+	+	+	RS2CL715s
	CL721	CCTAAATGTCTCTCCCGCAAAT	AAAGCCGTAGACCGATTCTGAG	+	+	-	-
	CL731	TTCAAGTCCATGGTGAGAGACC	AAGCACAGTTGGATGAGCTGAA	+	+	+	RS2CL731s
	CL753	AGTGAGGATGCCAACCAAAGTT	GCCCAAGGATTTAGCAAAACAG	+	+	+	RS2CL753s
	CL781	TGGAGTTTGGGAACCCTAAAGA	GCACAGCAAAGCTCGTTATCAA	+	+	-	-
	CL811	TCTTTGGACGAAGTGCCTTCTA	CGCTAGACGCTCTTTTTGAACA	+	-	-	-
366	CL722	CGGACTTGGAGATGGTGTAATG	AGAACCGCCTACATAACCGAAA	+	+	+	RS2CL722s
	CL737	GAAAACTGTGGCTGCAATACCA	TTCTCTGGCATCCCTTACCCTA	+	+	+	RS2CL737s
	CL768	CCAATCTCTGGTGAAGTCATCG	TAGCGTAGCATGGGTTCTCTTG	+	+	-	-
	CL791	CTTGAAGCAGAACAGAGGCAAA	TTCAAGGTGGTGAAGGTGTCTG	+	+	+	RS2CL791s
	CL808	AAAGTACCTCCATCCCAAGGTG	GCGACCCAATCAATCCTAACTC	+	+	-	-
	CL817	TGAATCAGTGGAGGAAGCCATA	CTGAGTCAAAGCATGCGGAAT	-	-	-	-
367	CL751	GAACAGTTTTGGGAGGAGTTCG	TTGGCCTCGTACACCTTCTTCT	+	+	-	-
	CL758	TCTCTCTCCGGGGATTGATAGT	CTGTCCGCATAGATTGATGTCC	+	+	+	RS2CL758s
	CL782	CCATCATCATCCCATTGCATAC	CCAAGGTTTATCACCCGAATGT	+	+	+	RS2CL782s
	CL785	GCGAGAGGAAAGCGTTTCAAT	ACAAGTTGCAGCATCTGTTCG	+	+	-	-
	CL793	ACTTCCTTCTTGCCCTAACAGC	TTGACTTAGCGCAGATCGTAGG	-	-	-	-
	CL804	ACAAGTACGCCTTGCCTCTTCT	GATTCAGCAGCAACCACTTCAC	+	+	+	RS2CL804s
368	CL840	GAATCAAGAAACCGGAGTTCCA	AGTTAAACAGCGGCTTCAGCTC	-	-	-	-
	CL842	AAAGAAGCATCAGTGCCACAAG	AGGAGATTGCTAAAGCCAAGCA	+	+	+	RS2CL842s
	CL847	AAGTAAGTTGCTTGCGTCGAGA	GCTAGCTGCAAAGCACAATGTA	+	+	-	-
	CL865	TTCCACCAGTGATACGGACAAG	GCGGAACTACAGGAAGAAGGAA	-	+	-	-
	CL868	TACCACCTGCTGAGGAGAAAGA	CCCAGGAGGAGGGTCATATTTT	-	-	-	-
	CL914	CACGATGAGGAAATCCCTTCTA	AACGGCCTAACTTGATTGGAGA	+	+	-	-
369	CL827	AGCTATCGGAGAGCATGAGCTT	CTTCCGGATCAAAGTGACTTCC	-	+	-	-
	CL830	ACGACGAATAGGTGGGTTTGTT	AGCAGCCTAAGCTCTTTGTTGG	-	+	-	-
	CL864	AAGAACGAGAAGTCCCTGTGGA	GATCTTGGGTCCTGCCTTATCA	-	+	-	-
	CL885	CTTTGGTTCGAGCTGAAAGGTT	CAAGCAACTGATCCTAGCGAGA	+	+	-	-
	CL892	ACTGATACTGTGGGCCTTCACA	AATTATGAAAGGCGTGGTGGAC	-	+	-	-
	CL907	CGCCACTAACATACTTCCGTCT	TCCGTACGAGGTGGTCAACTTA	+	+	+	RS2CL907s
370	CL821	GAGTCGGTGCTTTCCGTAGAAT	TGAGTGCAAGTCTCAGCAATGA	+	+	-	-
	CL826	TGAAGAAGGAAGGCATTCACAG	GGTGGCTTGAAGCTTGGATACT	+	+	-	-
	CL831	AGCAAATGGGAACGAGATGACT	GACATCTTGAAGGAGCAGTGGA	+	+	+	RS2CL831s
	CL843	GAGATCCGACCAAATCTGCTTC	TCCGGTTTCAAAAGGAAGTCTC	+	+	-	-
	CL853	CATTCTTTCCTCCCGTAACCAG	GATGTGCTGAAAAAGCTTGGTG	+	+	-	-
	CL881	AACCCCAAGGCCAAATCTTACT	AGCAAAACCACTGTTGTGCCTA	+	-	-	-
371	CL823	CCCGTTTGAATTCCTTCTTGAG	ACCGTATTGAGCCTGTGTACGA	-	-	-	-
	CL832	GGGGGAAAACCAAACGAAATAC	CGGGTTCATTAGGGAAAACTTG	-	-	-	-
	CL836	CTCTCTGGAGATGATGCCAATG	TCCACGGATCAGACTAACTCCA	+	+	-	-
	CL845	CTCAGCAAGAGCCGAAACAATA	TTCACCTTCTTCCTGTCCCTCT	+	+	-	-
	CL848	CGAAAACCTCATTTCTCCTGCT	CCGAACGTTACTCCGAAAAATG	-	-	-	-
	CL905	TGTGATTGGAACCTCCACAAAG	AAATCTCTCAGGGGGAAACACA	-	-	-	-
372	CL852	CTCACAACAGCAACAACACTCG	CCCAATCAAGATTCTCCTCCAA	+	-	-	-
	CL858	GGCAAGCGATGAAACTAAAACC	CGGATCATTCAATCTCAAGCAG	+	+	+	RS2CL858s
	CL863	CCTTTCCAATCCACGTTGAAGT	GCGAAAGCGTTTCCTTTGTACT	+	+	+	RS2CL863s
	CL876	GATCCTGGAGAGGAGGAAACAA	TGCCAGATTCCTTGAAGAGTTG	+	+	-	-
	CL882	GCATAACAAGGGATGGGAAGAA	CTTACACAAAGGCAAACCATCC	+	+	-	-
	CL896	TACTAGCGAAGCGGAGGAAATC	GGAGAACCAGCCAATTTGAGAC	+	+	-	-
373	CL846	TTCCCGGTTTAGATGTGGTTCT	CAGGAATGTTCAAAGCAGGATG	+	+	-	-
	CL874	TATCCTAGCCCGCTCATACTTG	TCCAGTCAGTCAGTTCCTCACA	+	+	+	RS2CL874s
	CL880	AGCCAAGGAGTTGAAAGTGGAG	CGTTCTGAAGCACAGGAGTGAT	+	+	-	-
	CL895	GGGATTGAAGGAAGCAGAGAGA	TTGAGCAGATCATCGCCTAGAG	+	+	+	RS2CL895s
	CL906	ACGAGCAAGAGTTCCGTAGCAT	AGCAGCCGTTCTGAGTTTATCC	-	-	-	-
	CL910	ACACCAGTACAAGCGCAAGAAG	TGGAAACATATCAGACCGCTTG	+	+	+	RS2CL910s

374	CL923	TCCGCACCCTTGAGAGTAAGAT	CTCCAATGGCTCCAAGAAGAAT	+	+	-	-
	CL928	ACCCAACGTAGGAAGAAGAACC	AACTTCTTCGATACCGGGAATG	+	+	-	-
	CL954	GCCCTGTCCAAGAGAAAAAGAA	AATGGTGGTGCTGAAGCTAGTG	-	+	-	-
	CL958	CTCGTTGGCTTGTCTCTCAGAA	GGTGCTCCATCATGCTCAATAG	-	-	-	-
	CL964	TGTTCTCAGCTTCGTCGTCTGT	TAAAGGGCCCAACATAGAGAGC	+	+	+	RS2CL964s
	CL978	GACCAATTGGGTTGTCACAAGA	GTGCGATGTTAAAAGGACGTGA	+	+	-	-
375	CL916	AATTCAACTCTGAGGCTGATGC	GGGAAGTGGAACAAAGCAAAAG	+	+	+	RS2CL916s
	CL929	ATAAGGGCAGCAGAGATGCCTA	TTGGTGAAAGCAACTGGGATAC	+	+	+	RS2CL929s
	CL943	ACCAAAAGCCTCTCCCAAATCT	CATCGACCAACTCCATCTTGAC	+	+	-	-
	CL946	TGGAAGTGGACCAGCGTACATA	AAGTGTTGCACGGAAAGAACCT	+	+	-	-
	CL951	CACTTTTGAAGAGGGGGAGAAA	CCTCAAAGCCAAACGAACTACA	+	+	-	-
	CL957	CAGCAGCAGATGGAAGAGCTAA	GACAACATAAACCCAGGCAGAA	+	+	-	-
376	CL933	GTTGAGTTTGGCAGGAGCTTTT	GTAAAAGCACGAGAGCCCTGTT	+	+	-	-
	CL934	TTGAAAACGTAGGGAGGACGAT	AGCACACATGAAGACAGGATCA	-	-	-	-
	CL939	CCGCTCTTAAACAGCTGGAGAT	TGCATTGTCGTATTGCCTTCTC	-	-	-	-
	CL942	TCAAGATGATGCCTTTCTAGCC	GAACCGTGTCGGTGATTGTTTA	-	+	-	-
	CL955	CATATATCACCACCCGTCATGG	ACAACGGTGGATGGAATGTCTT	-	+	-	-
	CL974	TCTTCACTCAGCTCGTCCATTC	ATCAAGCTAGGCGAGAAGCAGT	-	+	-	-
377	CL979	CCTCATTTCTGCAAGAGCAGTG	CTCTGGAGCAATTGTGATGAGG	+	+	-	-
	CL994	CATACGCCAGTTCTTCCACTTC	ACGAAGATGTTCGATGGGTACA	+	+	+	RS2CL994s
	CL1002	GATGATTTAAGTGGCCGGAAC	CCACTGCTTACAAGTGCCAAAC	+	+	+	RS2CL1002s
	CL1003	GTGCAGCCGTATCAGTTTGTTC	GCTCTGCCATTGCTTCTCAGTA	+	+	+	RS2CL1003s
	CL1006	CTTCTCGGGATGGGAAAATTG	ATAACACTCGGGCATGAGGAAA	+	+	-	-
	CL1015	ATACGACAAATCCCCGATGACT	CTGAGATCCGCGTAAACCTCTT	+	+	+	RS2CL1015s
378	CL982	CTGTTGAAGCCCAACTCAAGAA	TCTCACTCGGATGTTCACACAA	+	+	+	RS2CL982s
	CL983	GCTCCAGCATTGATCGGTTT	AAGCTCCTTTTGCTGATCATGG	+	+	+	RS2CL983s
	CL986	GATGATGAGGCAGTTGGTGAAG	TAGCATTGAGAATGTCGGGATG	+	+	+	RS2CL986s
	CL987	GTCATATGTCCAACCGCATCAC	GAGCTTCAGCTTGCTGGTTTTA	+	+	+	RS2CL987s
	CL993	CAAGTGTTCTCTTCGGACGTGT	ATCTCACGGTGCTCTTGACAAA	+	-	-	-
	CL1001	AGTTGGAGCCTTTTTGTTCTCG	AGCAACAGGCCCAAAATATGAC	+	+	-	-
379	CL1029	TCCGTCTCAAGGCTACCAGAAT	TAATCATTGCTTGTGGGTGAGG	-	+	-	-
	CL1034	ACGGAGATCAAGAGGGATGAAC	GAGTGAAGATCACGATGGCAAG	-	-	-	-
	CL1043	GTCTTGCACCTGAGATTCCTGA	TCTCTAAGCAACAAGGGTGCTG	-	-	-	-
	CL1047	AGCTACCGCCTGATTTTCAAAG	AGACAAACCCCAGACCAGAAAA	+	+	+	RS2CL1047s
	CL1050	CGACTGTCATGAACCATTCTCC	CAAATCAATAGACTCGCGCAAC	-	+	-	-
	CL1066	TCATCGTCACGTAGGACAGACA	TCGTACGTCTGAACAAGCGAAT	-	+	-	-
380	CL1016	AGCTTCTCGTGATGGGAAAATC	CGAGTAAACGACGAGTTCGATG	+	+	-	-
	CL1041	TAATAGGTGGACGGACGAAACA	CATGCACGATGACAGCACTATT	-	-	-	-
	CL1067	TTGTCCTATGATGTCTGCTTGG	TGCCCATTCCATGTTTGCTA	-	-	-	-
	CL1070	GATGCGCTTTTGTTGGTGTATG	GAAGAAGCCTCTCCTCCTTTCA	-	-	-	-
	CL1077	TTATCTTCACCGTTGGCTTCAC	CACGGTAAACGACATGAAGCAT	+	+	+	RS2CL1077s
	CL1083	GAACTTGAGCTGCCATTCATTG	AGCCCATTGTTTCTCGTCTCTT	-	-	-	-
381	CL1017	ACACCAACCCTCCTCAAGACAT	AGGAGGCTTTGCATCATCCTTA	+	-	-	-
	CL1020	GGTTACGATGGAGGCTTTTGTC	GAACGTCTCGGGGATCTTGTAA	+	+	-	-
	CL1040	CAGCATGCATATACCCATCACC	ACCACGTGTTGATCCTCTTCAA	+	+	+	RS2CL1040s
	CL1044	ACTTGCTTGGGCTTCTCTTCAT	AGTCCGACCATAACCGCTAGAA	-	-	-	-
	CL1055	TACTGTGTGGCTCTTGTGGTTG	GGCAGCATATAGCTTGGTGAGA	+	+	-	-
	CL1092	ACTGGAATTGGAGGTGAAGAGC	CTTTGATGTCCGCGGATAAACT	+	+	+	RS2CL1092s
382	CL1022	GGCAACCGTTCAAGTTTCTACC	AAGGCAAGTCAACTCCAGCTTC	+	+	+	RS2CL1022s
	CL1051	ACGTTCAGGAGATGGTTCAACA	TTGTAGACGAAGTGCGATAGCC	+	+	-	-
	CL1057	CTGGCTGTGCAACTCCTCTTTA	AGGAGGAAGTGTCGGAAGGATT	+	+	-	-
	CL1058	TCCTGAGGAACAATGGAACAAG	CTGGACTCCATCCCTTTGAGAT	+	+	+	RS2CL1058s
	CL1063	TGGTGGAAGTCTACAGGCTTGA	CTCACGGTTTCAAAGACCTCCT	+	+	-	-
	CL1089	GTTGTCTGCGAAGCTCAAGAAA	AGTCCCATGTGGTATCGTTGAC	+	+	-	-
383	CL1021	TTCGAAAGGGGACTAAAGGACA	TTTATCATCCAAGGCCTCCAGT	+	+	-	-
	CL1045	GACCGAAAACAACTCCACTAGG	GATGGTTTCTTGGTTGGTGGT	+	+	+	RS2CL1045s
	CL1056	CAACGGTACACGCTACTTTTCA	ATGTCGAACTTTGGAGGAAAGC	-	-	-	-
	CL1064	TAGAGATCCGGAAGTTCCTTGG	ACAGCGGAAGCTCTGTTCTTCT	+	+	-	-
	CL1068	GTGGAGAGTGTAAAGGCCACAA	TCACCCCTGATACAAGCTGAGA	+	+	-	-
	CL1073	CAATCTTCACCAATTCGTCTCG	CAAATCGGATTGAACGAAGGAG	-	-	-	-
384	CL1024	AGAGAGAGGAGCTTCCTCCAAA	AGGCCTACATCCACATAAACCA	+	+	+	RS2CL1024s
	CL1027	AGAGCTCTCATCACAAGGCTCA	GGTTCTTGCTGAAGCCACTTCT	+	+	-	-
	CL1048	CACTCGTTGATTCTGGGGAAAT	AAGGCTCGTTTTGGTGTTCTTC	+	+	-	-
	CL1071	GAACGACCCAAGTGTCATCGTA	CAAGGCTTTTACAGCTCGTCCT	-	-	-	-
	CL1076	CAAATGGAAGCCAGTTCTTCCT	AACGAATAGGGCTTTGGTTCAG	+	+	-	-
	CL1090	GATCAGATCAAGCGGTGGTAGA	ATGGCGGGAAAAGTGTGTATG	-	+	-	-
385	CL1095	GCCTCGTGATCGAAAAGGATAC	TCTGAATTGAGGAAAGCCCCTA	-	-	-	-
	CL1096	GTAACTTTTGGTTTCGGGGTTG	TACCGTCACCATCCATCTCTTG	+	+	+	RS2CL1096s
	CL1098	AGTATTCCCTTTGCCATGGTTG	TTCAGTTGTCGTATCAGCCACA	+	-	+	RS2CL1098s
	CL1157	CAGCTCTGCTGGAGAATCCATA	TCTTGGGTTTAGTGGTGGTTGA	+	+	-	-

	CL1187	CATAGCATTGATGGCTCATCCT	CCGAAAAGAGAGCAAAGAGAGG	+	+	+	RS2CL1187s
	CL1189	TGTCAGAGACACCGATGTCAGA	CTCCAATGCACACAAAGCAAGT	+	+	-	-
386	CL1101	TGACACTGGTTGCTTTGGATTC	TTGCATCATCCTTGGTCACTCT	+	+	-	-
	CL1117	GGCTCAGCAAAGACAAAGGAAA	GCTATAATCGGGGAAAGCCTCT	-	-	-	-
	CL1120	TTTTCTAGGCGACTGGGATCAT	AAATTCAAGGACGGGTACATGG	+	+	+	RS2CL1120s
	CL1162	CAGCTGAAGAAGCTGATGAACG	TCAAAGCCCATTCCATTGGT	+	+	-	-
	CL1178	CACGGTCGACGATTAAATCAGA	GGAAGCAGACCACCTCATTTCT	+	+	-	-
	CL1180	TGCTATCTTTTGCGTCATCCTG	AACGTATGCCTTGCACCCTACT	+	+	+	RS2CL1180s
387	CL1102	CTCTGCGTTCTTGGCCTTATTC	TAGTGAACTCCAAAGCCCATGA	-	+	-	-
	CL1115	GTCTGCGGAGAAGAGAATTGAA	AGCGAAAAGAAATCCCCTCAAC	-	+	-	-
	CL1147	GCATCCCTTATAGCCTTCTCCA	TCTCACAACAATGACAGGCAGA	-	+	-	-
	CL1161	AGCCAAATTCAATCAGCTCTGG	CCGACTTCGACATAACCACAAG	-	+	-	-
	CL1167	AACCCTCAGTTCCATCTGCTTC	AAGGAAACCACCACGCTTACAT	+	+	-	-
	CL1175	CACTCGCATGTTTTTCTCCAAC	AAAATGAGTGGCGTGCGATT	-	-	-	-
388	CL1093	CTTGGCTTGGCTTCAGTACCTT	GCAGTTTCCACATCCAGAAGAG	+	+	-	-
	CL1127	GAAGCTTGGAATGAAACCCATC	TTGAGAGCCTTCACTGCTCTTG	+	+	-	-
	CL1141	GGATCGAGGAAGAATGAGCA	TAGCTTTTCAGCATCGAGTTCC	+	+	+	RS2CL1141s
	CL1142	GGAGGATAGCACATAACGCTGA	CGTCTCCTTTTGCTGATTCCTT	+	+	-	-
	CL1185	CTCTGAACGTTGGAACCCTGTT	AACGCAGGAGAGATGAATCACA	+	+	-	-
	CL1191	AGCTCCCTCCTTTTTGAAGACC	TTCAGTGTCAGCAGCAGTACCA	+	+	-	-
389	CL1097	AACCCTAAAGGGGATGGAGATG	ATCACTAGACGGGGACGATGTT	-	-	-	-
	CL1099	ACAGAGCCACAGGGTATGGAAT	TGTGGTATGCATTCTCCACTGA	-	+	-	-
	CL1110	GCTGCGCCTATCTCTCTATTTG	CCGAGACCTGCAGTTTCTTCTT	+	+	+	RS2CL1110s
	CL1118	ATCCCAGTTCGCTATCTTTTGC	CATGGACACCAACCTTCACAGT	-	-	-	-
	CL1119	CTGCATTGGGACAGTTAGGATG	AAGCCAAAAGCGTAAAGCCTCT	-	+	-	-
	CL1131	ATCCCGGCTTCATACATTGAGT	ATGTAAGCGTCAATCCACACCA	+	+	-	-
390	CL1123	GCATGGTGTGCATCTAAAGGAA	TGTATGGTAACCCTCTGCAACA	+	+	+	RS2CL1123s
	CL1138	GAAGGTCTTGCTTCAGCTGCTT	GACATCGTGCCTAAAGACTGGA	+	+	+	RS2CL1138s
	CL1145	CGATACTTGCCAAGGGCTTTAC	ACGCTCGACTCTTCTCACATTG	+	+	-	-
	CL1149	GAGAAGCGGTCGCTGTAAAGAT	GGGACTTTCTGTGTAGGCTTGA	+	+	+	RS2CL1149s
	CL1154	CATGAGACTTTCCGTGACCAAC	TCTCTCCAGACCAAACTGACCA	+	+	+	RS2CL1154s
	CL1172	AGCAGGGACATGGGTAACAAAT	CTTGACATGAAGCTCAGGGATG	+	+	+	RS2CL1172s
391	CL1203	AGTCGGCAAAGAAAATGAGTCC	AAGAGTGCAGCCTTTCCAGAAC	-	-	-	-
	CL1214	TATGCAGATGGTTCGTGACAGA	TCCAGTGAACCGCATAGTGAGT	-	-	-	-
	CL1215	CGCATTGACTTGTCCGTATAGC	AAATACCCCCAGTGCTCATGTC	+	+	-	-
	CL1219	TCATCTTCTACCCCATCGTCCT	CTATTCCTGAGTTTGCGGCTTT	+	+	-	-
	CL1226	CAACTTCGATTTCCAGCCTTCT	AGAAGCATTCAAGCTCACACGA	+	+	-	-
	CL1292	TGCAGAACATCCTCTGGCTAAA	CGGTTTCACTAGGGGAATGAAG	-	-	-	-
392	CL1208	TTCTGTGATTGAAGCGCCTTAG	CGCTTCTTGAGCTGTCTTGACT	-	-	-	-
	CL1235	GAAGGATACAAGCCGCAGATTT	ACCGATGTATAGCACCGTGGAT	+	+	-	-
	CL1243	CTTAACGGTTGGCCTTAGCAAC	CCTTTGAGACGTGTGAACCAAG	+	+	-	-
	CL1247	GCTTCTTGACGAGCTCGGTATT	TGGTTTCAGAGTTTGTGGATGG	+	+	-	-
	CL1249	CCTTAATGATTCCACCGCTTTC	AGCTACCTGATCCATTCCCAAG	+	-	-	-
	CL1253	GGACCAGAGGCAAGAATGTTTC	AGCCTCATTCTTGGAGACGAA	+	+	+	RS2CL1253s
393	CL1195	AACCAGGCTGGAACCAATTTAG	GAGCAAGCAATGTGGATCTCTG	+	+	-	-
	CL1199	AGAGCCAACCACAGAAGAAACC	CTGTTTCTCTTCCGACACGATG	+	+	+	RS2CL1199s
	CL1209	TGGAGAGGACCCAATATCTGCT	CAGTATTACGCACCACCCAAAA	-	-	-	-
	CL1269	CGCCATCAAAGGACTTAAGGAT	GTGAAACCGGATTTGCTACGTT	+	+	+	RS2CL1269s
	CL1282	CCATGGAAGCATCTCCAATAGT	CCATGTGTTTAAGCCACACCAG	+	+	+	RS2CL1282s
	CL1284	TAGGGTTGCCTGTGATCAAGAA	CCAACAAAGAGCACGACAACAT	+	+	-	-
394	CL1197	GACGTGTACAAAAGCGGTGAAG	TCAACCGGTCTTCAATCGAGTA	-	+	-	-
	CL1211	CATTGATCTCGTCTTCCTCGAA	GGTGTGTGTGAATCAATCAACG	+	+	-	-
	CL1224	CAACCTCAACGTGAGGGTTACA	TGTTCTCTTTCTTGGGGCGTAT	+	+	-	-
	CL1245	AGTTGTCAACCGACCAAACTGA	CTCCATGCGAGTCACAGAGATT	+	+	+	RS2CL1245s
	CL1262	CAGAATCTGATCCCACCTTTCA	CAGCAATGTCAGCTTTCACCAT	+	+	-	-
	CL1276	CTGGGGATTTCCTTAGGGTTCT	AATGGGTAAGAAGGCCAATCCT	+	+	-	-
395	CL1216	ACACGCAATGAATGAGAGTGCT	GTTGAGAAGATCAGGCCAACAA	-	+	-	-
	CL1252	ACTTACCAACTCGGTCCAGGAA	AGATGATGCTAAACCCCGTCAC	+	-	-	-
	CL1265	GAGGAAAGAGGAACGGTGAAAA	CTTTTCAGCCTTGTTGATGCTG	+	+	-	-
	CL1267	CTTCTCGAAGGCATGGCTAATC	TGTCAACGGTTCGACCATAATC	+	+	-	-
	CL1283	GATTCCAAAGCCAACTGCTCTT	GTCCACACCTCGGCAACTTTAT	+	+	+	RS2CL1283s
	CL1289	TCAGGACGAGAACTTCAAGCTG	TCGTTCTTGAGCTCACCACAAT	+	-	-	-
396	CL1213	AGACATGGCAACCACAGAGGTA	AACAACGAGAACAGCTGGGTTT	+	+	+	RS2CL1213s
	CL1217	GCAAACATCGAAATGCTCTCAC	GAAATGATGCATGGGCTTTG	-	-	-	-
	CL1221	ATGCAAATCGCTACAGCTTCTG	TGCTCACTGGCGAAAAGAGTAA	+	+	+	RS2CL1221s
	CL1239	TCGGGTTAAAGCCTAATCTCCA	CGAAGACGCTGAATCTCTTCCT	-	-	-	-
	CL1256	AGCTTTCGCTCTTCTGATCCTC	CGCTTTATCGGGTTTCACATCT	+	+	-	-
	CL1286	GCTTAGCTTCACCGAGGAAAGA	ATGGTCCAGGATTCTCAATGGT	+	+	+	RS2CL1286s
397	CL1305	AAACTCCGAGAGAACCAACGAG	CAATGTTCAGCAGTCACCGTTT	+	+	-	-
	CL1352	GTGCGAGTAAACCCAAGGAATC	CAGCTGCTTCAGCATTTTCTGT	+	+	-	-

	CL1355	CGGATATGACCCTTCCCTTTCT	TGTAGCCACACTTGATCGGTTT	+	+	-	-
	CL1375	GAGACTGAAGAGATCGGCGTTT	CGAGGACAACAGTTTTCGTTCA	+	+	-	-
	CL1388	CCGTTTCTCTCCTTCACTTTCC	TTCCCCTAGTTCGACATCTCCT	+	+	+	RS2CL1388s
	CL1389	TGGTAGGAGACCAGCTTGATGA	GCTGCAGAATTAGCCAGGATTT	+	+	+	RS2CL1389s
398	CL1297	ATTGGCAGCTCAAGTCACAGAG	GAATCTGCAGGTGGTTTCTTCA	+	+	+	RS2CL1297s
	CL1313	AGGAAGTGGCAAACCCAAAGTA	AGCAGCGGAATGGTAGAAAAAG	-	-	-	-
	CL1329	AAGACGATCAAAAGGTCGTTGC	AAAACCAACAGAGTCGCATGAC	+	+	-	-
	CL1331	ATGAAAGATCTCCAGTGGGTTG	GGGGAATCTAGCCATTGATGTA	+	+	+	RS2CL1331s
	CL1342	GATGGTGAGGCTGAAGCAAGTA	AACGATCCAGTTGTTTGCTCCT	-	-	-	-
	CL1383	TTCGACGTTGGTGTTAAGGAGA	GTTGCATGACGACTGACGAACT	-	-	-	-
399	CL1296	ACTTTCTGGCTTAAGGGCAATG	ACCCGGTTTAGACATCTTCAGC	-	-	-	-
	CL1299	ACACCACACCAAACAACCAAAC	ATGCGAGCGTAAACAACGTC	+	+	+	RS2CL1299s
	CL1300	CTTCTCCACGTTCTCTCGTTCA	ACATGCTGCACCAGAATCTCTC	+	+	-	-
	CL1320	TCGGAGTAAAGCTGACTGTCCA	ATCCAAGGCGAAGGTATGTCA	+	+	+	RS2CL1320s
	CL1326	GACCTATGGTTCGTGCTCAACA	AAACCCAACCAAACAACAGACC	-	-	-	-
	CL1327	TTCGAGGAGATATTTGCGAAGC	AGATTCACAGGCGTGCAAGTT	+	+	+	RS2CL1327s
400	CL1301	TGTGATCAGATACGGGCGTAAA	TTGTTTGGGGGTAAGGAACAAG	+	+	+	RS2CL1301s
	CL1337	CCACGAAGCTCTTCTTCACCTT	CCTTTGCCATCAGAGAACAACT	+	-	-	-
	CL1347	TGACAAGCACATGTACCACGAC	GCGCAGAGTAGGTGTTTTGACA	+	+	+	RS2CL1347s
	CL1348	CAGACACATGGAAGAAGCCGTA	GAGAAAGGGGATGGGACACATA	+	+	-	-
	CL1357	TATGGCTACGAAGGCGTGAA	AGCCCACGGGAAATAAATTAGC	+	+	-	-
	CL1372	GCAAATGCACGTTGATCTTCTC	GTGCACACAGAAGCGACAAACT	+	+	-	-
401	CL1333	CAAGATTCCTTACATCGCACCA	CGCCGAACAATAGTTCAAAGAC	+	+	-	-
	CL1339	CAACACCTCTGGATTCTGCAAG	GCTGAGACCAGTGATCCAAATG	+	+	+	RS2CL1339s
	CL1340	GAGATGGGGAAGAAAACTGCAT	CTTCCACCAGCCTCTTCAACTA	-	+	-	-
	CL1358	TTGGTTCATCAAGCAAGTCCTG	ACTACCCAAGCAAACCTCCAAA	-	-	-	-
	CL1368	AGCTGCTTCTGGTTTCTCTGCT	TACCAGTTCTCAAAGCCACGAC	-	-	-	-
	CL1381	CTTGTTGGCCTCGTTTCTCTTT	GATTCCCATGGCTGCTAATCTC	-	+	-	-
402	CL1401	GCTCATTTTTCCCACTGACCTT	ATTGGGAAGCACGAGAAGAGAG	-	+	-	-
	CL1406	CTGAGACCACCACAGCTTCAAT	AAAAACATGGCTTCTCCCTCAG	-	-	-	-
	CL1407	TTCAGCTTCAAGGAGACCAGAG	TCCCCTTGCTCGAGAGAGATAA	+	+	-	-
	CL1409	CCGCGATATTGCAGGAAGTTAT	TCCATCATCACCTTCCTCTTCA	-	+	-	-
	CL1452	AGAATGGCGAAGGAACTAGCAG	GTTCCAACTCGATGGCTTTGTT	+	+	-	-
	CL1468	TGAAGAAGGGAAACCTCTTTCG	CGACATAAAAGCATCCAAGTGC	+	+	-	-
403	CL1390	ATTACACTCGCCCAGGAACACT	GGTGTGGAAGCTGGTAAAAAGG	+	+	+	RS2CL1390s
	CL1399	TAACCTTCTTGAGGCTCCGAAC	GAAGGATCTTGAGCTGGGAAGA	+	+	-	-
	CL1431	TGCACTTGTGGTGGCAAAAT	TAAGTCCCTCTGGTTCCTTTGG	+	+	-	-
	CL1463	CCATCTCAGTTGGATCAGTTGC	GAACGCAGGAGAAAGGAAAAGA	-	+	-	-
	CL1467	CTTTCATGGCAAATCAGTCAGG	CCCGGAAGGATTATGATGAGTC	+	+	-	-
	CL1442	GGAAAGCAGCTAAATCGGAAAG	AGGGATGAGCTCTGGTTCAATC	+	+	-	-
404	CL1403	AGGAGGGAGCTTCTGCTTTCTT	CACATAGAACTCGCACAAATGG	+	+	+	RS2CL1403s
	CL1425	TGGTGGTGATGAAGCTAGTGGT	CCATTTCCATCGCTCTCTCTCT	+	+	+	RS2CL1425s
	CL1459	GCAAACACAGCTCAAAGGTTGT	ACTTTGCTGCTAGGTTCCCAAA	+	+	-	-
	CL1460	CCCGTTTGATCTCATTGACGTA	ATTGAATCGTCTTCGACCTTCC	-	-	-	-
	CL1465	CCAAACACCATTGATTGTCCTG	TTCCTCAGTTCAAGCCTCAAGA	+	+	-	-
	CL1480	GGCACGCTTTTCTACAGTGGAT	AACAGAATCATCAACCGGAGTC	+	+	+	RS2CL1480s
405	CL1396	CTCCTGGAGTATTTCCAAGCAA	GATGGATGGATTGAAGGGATTC	+	+	+	RS2CL1396s
	CL1423	GTTTGCTTCAGTGATCGTCGTC	TGGAGTAGTGGCATCAGTTTTG	+	+	-	-
	CL1444	CTCATCATCACCACTTCCCAAA	GTTAATGTTGGGCAAGACACCA	+	+	+	RS2CL1444s
	CL1462	GTGGCGCGAATTGGATATTAAC	TCAATGGCAATAGAGGAGAACC	+	+	-	-
	CL1464	GCTTCCCCCGAATGCTATAAT	TCCAAACAGGAAAGACACAGGA	-	-	-	-
	CL1473	TCACTTGCAGGGGAACTTGTAA	ACGAGGGTTCCTCTTCATCTTG	+	+	-	-
406	CL1397	ATCCCTGATCCCATTGATAACG	GGAGGGTTTTGACCCTTTTGTT	-	-	-	-
	CL1404	ATGCGACATATCCGAGACTTCA	GAGGGATCCACATTCCATTGAT	+	-	-	-
	CL1412	TCCAAAAGCTTTCGTGAGTCTG	TACGCAAGTCCCATAATCCTCA	-	-	-	-
	CL1430	GCGGCAACAGTGACACTATCAT	AATCTGCCACACGGTTTTCAC	+	+	-	-
	CL1441	CCTCTCCAAATCAAACCAAAGC	GCTCGATATCACCCTTGGAATC	+	-	-	-
	CL1478	GAAGCTGCAGCAAACAACCTAA	ACTTGGAGCACGTGATTTCTGA	+	+	-	-
407	CL1411	TTCCAATCAAACGTGGTTGC	TCCAATGGACTCTCCACTCATT	-	-	-	-
	CL1436	AATGGTTCTTGTTCAGGCCAGT	AAGGCAAGTGGTCTTATGGACA	-	+	-	-
	CL1440	CAGCAGCTTTATGGTGCAAATC	TATCAGCTCATGAAGGCTGGAC	+	-	-	-
	CL1450	CACAATCTTCTCACCGTTCGTC	CTCAATGTAGCGGGTGAAACAG	+	+	+	RS2CL1450s
	CL1456	ACGGCAACAAAGACCGACTA	ATCAAGGGTCGTCTCAACAGGT	-	-	-	-
	CL1470	TTGACGACGAGGAAGACTACGA	CTTGCGACACACAAAAGGAAAG	+	+	-	-
408	CL1488	AACTAGGACAATGGCGGCTATG	AGAAGAAGCCAGTCTCGAAGGA	+	+	-	-
	CL1499	GTCGGACTCCATCATCACAAAG	TGTTGCAGACACAAGCAGAAAG	-	-	-	-
	CL1501	CCAAAAAGGTGAAGGGGAGTCT	TGGTGAACAGATGAAGCATGG	+	+	-	-
	CL1550	GTTCTTGGGTTTGGTTCCATGT	GGATGAAGGAATCAAGCTGGTC	+	+	-	-
	CL1557	ACGCAGATGTGTCAAAGCAGAT	CTCAACGATGAGAGCCTTGAGA	-	-	-	-
	CL1571	ACTTCTTCAGCACAAGGGAAGC	CTTCGGTTTGGTTCGTGGTAAT	+	+	-	-

409	CL1487	CCTGCACACTCCACTGCTTTTA	TAAGCTAAACCCACCACCAACC	+	+	-	-
	CL1507	TTTTTGCGGAGACGCTAATCTC	CTTGTGCTTGATTGGCTTTCAC	+	-	-	-
	CL1514	CATGGCGAAACATGGAAATG	CAGTAGCATTCCTTCCTTCCTG	-	-	-	-
	CL1539	TCATCTCCTTTCGTCGTCTCTC	AGAACAGCAAGACATCCGTCAA	-	-	-	-
	CL1547	ACATGTAATTGGCACTGGCTCA	AGATCAGGGGCATCTGATGATT	+	+	-	-
	CL1569	GAAGAGGGATATACGCCCAAGA	TTGATTCAATCTCCTCGTCTGC	+	-	-	-
410	CL1485	CACGTGAATGACTATTGCGACA	CTCAGGGAGAGGAGGAATGAAA	+	+	-	-
	CL1496	ATGGGAGTGAAGACGAGCAAAT	GGGAAAGAGTGATCGGAAACAT	+	-	-	-
	CL1508	CAACTCTATCTATGGCCGCTGA	CGCTCAAGAAACTCGACAAAGA	+	+	+	RS2CL1508s
	CL1573	ACCACCGTTACTGCTTCTCCAT	ATCTTGGTGGAAGGACCGATTA	+	+	-	-
	CL1575	TCGTTCTGGTACCGGAGAGATT	GCCTTGAGAAGAAGACCAATCA	+	+	-	-
	CL1579	TAATAACCGTTAGGCCCATTGC	ACATGATTTCCTTCGGAAGTGC	+	+	-	-
411	CL1490	GAGGCATATGGAGAGTGCGATA	TGGTCATGAGAACTCCACGATT	-	+	-	-
	CL1503	ACGACCGATGCTCTAAAGAAGG	AAGCTTTGCAGGAACCAAGAAC	+	+	-	-
	CL1517	TCGGAATACCCTTTCTGGAGAC	GCTTAACTACTGACCGCAGCAA	+	+	-	-
	CL1534	CAATCCCTCTAAAGGCACCAGT	GTATTCTCCCTTCGATGGCAAC	+	+	+	RS2CL1534s
	CL1565	AACTTCAAAGGCCATGCTCCTA	TCCAGTATGGCCCAATGATACA	+	+	+	RS2CL1565s
	CL1578	AACCCGCGAATTCACTTACTGT	ATTGTCACGCTTGTTGACCAGT	-	-	-	-
412	CL1482	TTGATCTTTGTGGGGTACATGG	GGAATACGCAAACCAAAGTACG	+	+	+	RS2CL1482s
	CL1484	GAGTTGTCCAACAGAGCAGCAT	CATGGGAAGTAGAAACGGGAAG	+	+	-	-
	CL1510	TCCTCCTTTGGAAGTGGGTTTA	CATGGAACCACGAGTTCTTCTG	+	+	-	-
	CL1537	CAATCGATCACCGAGACAAAAC	TTGAGCACCAGAGGAGTATGGA	+	+	-	-
	CL1558	TTGGAGAAGGGATTCTCTCAGG	TGATGTTCCACCAGGAAAGATG	+	+	-	-
	CL1577	AAGAAACATCCACCCGGTAACT	GATGGGGTTTCGGTTTATGTTC	+	+	+	RS2CL1577s
413	CL1500	AGGAACTCCTTTTGCAGACGAC	AAGATGCGTACCTTGTGCATGT	+	+	+	RS2CL1500s
	CL1511	GTTATTGCCAGCCATGGAGAAT	ATGGACGATTTCGCTTCTGAGT	-	-	-	-
	CL1513	CTCGTTGTCTTCACCACCAACT	TCTTCAACTAGAACGCCACTCG	+	+	+	RS2CL1513s
	CL1520	TGTCACAACGGTTCCAGTTTCT	GTCTGATTGCTTGGATTGTGC	+	+	-	-
	CL1538	TGTCGATAAAGCCTCGCAGAT	AAGCGTTTCGCTGGAATCAT	+	+	+	RS2CL1538s
	CL1549	TCGAACCATCTTGGTGTGAACT	GCTTAAAGAGGTGTGGCATGTG	+	+	+	RS2CL1549s
414	CL1609	AACCTTAAATGGCTGAGGGACA	TGTTACCCCATCTTCCCAACAT	+	+	+	RS2CL1609s
	CL1651	TCTTGTCATGTGCGGCTATCTT	GATACCGGAAACCATTTCTTCG	+	+	-	-
	CL1653	CAACTTGTTGTTCCGTCTTTGC	TTGTCTGGAACGTTCTTGTTGG	+	+	+	RS2CL1653s
	CL1654	GATAGGGCTGTATCCATCAACG	GAAGGAAGAGAACATGGCTGCT	+	+	-	-
	CL1658	CGTGCCTCTCTTTTAAGCACCT	TGTGGTAACGGCTCATTTCGTA	-	-	-	-
	CL1665	TTGTTTGTTCGTCCTCATCGTC	GCACCTTTCCAGTGAACTTGCT	+	+	+	RS2CL1665s
415	CL1598	TAGGAGTGCCATGAATTTGAGC	CGAACATGGCAAGTTTAGCACT	+	+	+	RS2CL1598s
	CL1612	CTTTAGCATCCCCGACGTTTAC	TGTTCTCAACCTCACCACGTCT	+	+	+	RS2CL1612s
	CL1624	AAATGATGGTGGAGGAGAATCG	ACGACATAGAGATTGGCCATGA	-	-	-	-
	CL1638	AACAGAGATGGATCACGAAGCA	GGATATTTGTCTCCCCAGCAAG	-	-	-	-
	CL1641	GGAAACCATGCAAAAGAGGAAG	GAAAGATTTCGATGAGCCTGGT	+	-	-	-
	CL1649	GGGAACATCAAAGACGGAGAAG	CCTGTACAAAAGAAGGCACACG	+	-	-	-
416	CL1588	TTTCTGGTCCAAACGATGAGTC	CGCAAATGAGTCTGTGACGTTT	+	-	-	-
	CL1596	ACGGAAAGCTTAACCACCAGAG	GATCAAAATCCTGGAGGAAGCA	+	+	-	-
	CL1613	CAAGTGAATCCAAGTGGTCGTG	CACATTGAGTTGCATGTCCGTA	+	+	-	-
	CL1621	TCCTTTGCCTTCAGCTTCTCTT	ATCTGACCTTGCTTCTGCAATC	+	-	-	-
	CL1633	GCCGCAAAGCTTATCATCAA	CGATACTCAGCAAGTGCCCATA	+	+	+	RS2CL1633s
	CL1647	CCAAACAGAAAACAACCAGACG	CGGGAAAAGTTATGGCTAAACC	-	-	-	-
417	CL1614	GCTTCATCCACCACCCTTATCT	CTTGATCAAAAACAGCCTCTGC	+	-	-	-
	CL1617	AGTCCTCTCGAATCGTTCCTTC	CAGGACCACAAAAACTCTGCTG	-	-	-	-
	CL1619	AATGGAGCCATTACCTGCTCTC	GACATTTTCCTTCCTCCTGCTC	+	+	-	-
	CL1620	GCCAATTACGGACCTTCTCTGT	TACTCTTGGGTTCCATCGCTTT	+	+	+	RS2CL1620s
	CL1652	CAACTCCATGTGCCGAAAAA	CAAACCCTGATGATCCATTGTC	+	+	-	-
	CL1661	AGTGAACGAGACAAAGGCAACA	ACCAAGCAAGGATTCTGGAAGT	+	+	+	RS2CL1661s
418	CL1608	GAACGCTCCAACCATCTTCTCT	GGATCCTTGCAAATCTGAGTGA	-	-	-	-
	CL1611	GCAAGTGATTTGGAGTCAATGC	CAACATTCCGATCCAATAGCAG	+	+	-	-
	CL1615	CCAAACTGTGCCCTCACTAACA	TGCTCAGAAGCAGAGGATTCAG	+	+	-	-
	CL1630	ATGATGACCTTTTCCTCCTCCA	AGGGTTTGGTGTTCTTCTTCCA	+	-	-	-
	CL1650	ACCTTTCGAGACAAGCCTCATC	ACCCCCAAGGAGAAAGAGAAAG	+	+	-	-
	CL1655	TGTTACGGTCCATGGTTTGTTC	GAGCAGAATCATCAGCGACAGT	+	+	-	-
419	CL1626	TTGAGTACGCTTTGAGGGATGA	CGTAGAGCACATTCCGTAGCAA	+	+	-	-
	CL1627	AAGAAGAAAGGAGGGTGCCTCT	AAAGTAACGGCGCTCATTGG	+	-	-	-
	CL1634	ACTCACAGCCAAACAAAGATGG	CCTGAGACGGTTTGCATAAGTG	+	+	-	-
	CL1639	TGTTGCACAACGCAATGACTAC	CAGTGTGAAGCAAAAGGGAATG	+	+	+	RS2CL1639s
	CL1644	ATAGACATGCATGCCCCTATCA	AGGGTTTGGGTTTACCCCAGTA	-	-	-	-
	CL1645	GTTTCGCCATTGTGCTATTGTC	AGGAGGGATTTGATGCTACTCG	+	+	-	-
420	CL1672	GGGTCACGTGAAAGTTTGGATT	CACGGATCTGCTCAACTTTCTT	+	+	+	RS2CL1672s
	CL1674	TCTCAGTGCACCTTTGTGGAAT	CTTTGACATCTCCCAGTGGTTG	+	+	-	-
	CL1685	GAGATGGAGACTGTGCAAATGG	GGGTGGCTCAAAAGGATTAACA	+	+	+	RS2CL1685s
	CL1692	ACGCAGTCGGTTTGCTATCTTT	ACAGTAGCATCGAGGCTTTGAC	+	+	+	RS2CL1692s

	CL1699	AGCCATCTTGGGATGCATAG	ACACAACAGAAACAGGGACAGC	+	+	-	-
	CL1714	GACGCTTCTTGCAAAGGAAA	TCACTGATAAGCTTGGCCTTCA	-	-	-	-
421	CL1677	AGGAGAACCACAAGCAAGTTGA	ACGTGTGAGTCGAAGAGCCATA	+	-	-	-
	CL1680	TCTTCCTCAAAACTGGAGGTCA	GAGTTTCTAAGCGGCAGTAGCA	-	-	-	-
	CL1683	ATGGGATCAAATCTGAGCAACC	AATGGAGTGGTCAGGGAAAGAG	+	+	-	-
	CL1693	TCAGAGTGATTCCGAGGAGCTA	ATAATCCACAGATGGGGGAAAG	-	-	-	-
	CL1707	GAAAAGACCATGGAAAGCTCGT	GCCTCAATCAATGCCTTCTTCT	+	+	-	-
	CL1737	CAGCCAAACAACCAAACAGACT	TAGGGATGGTTCACAGAGGAAA	+	+	-	-
422	CL1667	TGCCAACTATTAACCGATGGAG	AGCTGGGAAAGTTGGTAAGCTG	+	+	-	-
	CL1689	ACGCTTGCATGTATCAACGAAC	ATACCGGAAGAACGTGATGGAT	-	-	-	-
	CL1698	CTCGCAAAAGGTAAAGGCAAAC	CCATGGCCACAGATGGTTAAA	-	-	-	-
	CL1703	CTATTGCGGATTACAGGCGAAT	GAGATGATCCCGAAGAGGAAAA	-	-	-	-
	CL1730	GCAAGAATCGGCCTTCTCTTTA	CGCTAACGGCTCCAAATAAGAT	+	+	+	RS2CL1730s
	CL1745	AGCGAGCCATTGCTTCTATTTC	CGACTCCGAGATTCAACACAAC	+	-	-	-
423	CL1676	GTCGTCTCGTCGTCATCAATTC	GAGCACAGTCTTTCCACTTCCA	+	+	+	RS2CL1676s
	CL1681	GCTTCTTCCTCTTACGCACCAT	TAGGGAAACATCCTTCCCACTT	-	-	-	-
	CL1695	TCGATCACGTCATCATCTCCTT	CGTAACCTCTTCAAACCCCAAT	+	+	+	RS2CL1695s
	CL1727	GTTTGCGATTTAGCTTGGGAGA	AAGCTCCGTTTTACCAAACTCG	-	-	-	-
	CL1732	CGAACATTGGTTGTTCATGTCC	TCCTTGGGACAAACCAAAAGAC	+	+	-	-
	CL1743	CATGCCATTCTTCCATCATAGC	TTCCAGTACAAGCTGGCCATTA	+	+	+	RS2CL1743s
424	CL1682	CCATCAGAGCAACAATGGAGTC	GGCAAGCACGTCTTTAGAGGAA	+	+	-	-
	CL1694	GATCTGTTCGCCATGGAAGATA	GCATCCGATCAAAAAGTCTCTG	+	+	+	RS2CL1694s
	CL1696	GGGAACCATCAAACCAACAAG	TGTATTTGTTGGGCTCCAGATG	+	+	-	-
	CL1713	CAGATTGTTTGCTTCCTGCAAC	CATTTGCTCAAGATGCTCCAAC	+	+	+	RS2CL1713s
	CL1741	AGGAAAACTTGGCATGGGAAC	CCACGAGGTTACAAAGCCCTTA	+	+	-	-
	CL1742	GAGCAACGAAGGGTTCAAGTTT	AGATTTGGTGAAGCTGGTCGTT	+	-	-	-
425	CL1668	AAAACCAGGAGGGCTTTACCTC	AGAAAGCCTTGTGTTCAGTCCA	+	+	-	-
	CL1670	AACCCATACAAACGGAACTTGG	AAATGGAGGCAGAGAAGACACC	+	+	+	RS2CL1670s
	CL1671	ATGTCCAGAGACGCCGATAGTT	CCTCAGGCAACCAATTCAGATT	+	+	+	RS2CL1671s
	CL1678	ATCCTCACATTCATGTCGAACG	TACTCTGACAACTTCCCGCAGA	+	+	+	RS2CL1678s
	CL1691	TTTGTTCTTCTCGCTTGTGCTC	CGCAATGGATTCCTCTTTTACC	+	+	-	-
	CL1711	TGAGTCTGTTCCTGCACTCAGA	ATTTCCGGAAGGCCAAGACT	-	-	-	-
426	CL1754	GGATGGAGATGTCCCTGACTTT	TGGCTGTTATTCGTGTGTGAGA	+	+	-	-
	CL1762	TGGAGCTGGAGCTAAGGAAACT	GGATCATCCCTCACCTGATCTA	+	+	+	RS2CL1762s
	CL1764	GATTTGCTCGCCTGTAACCTCT	CTTTCCCAAAGGGTTCATCTTG	-	-	-	-
	CL1769	GCTGACATGGGTAATGCAGATG	TAATGAAACGCTTGCTGGTCTG	+	+	-	-
	CL1799	GCTAAAATGACGAGCGTGAGTG	CCTCAGAGATGGGTTTCTGCTT	-	-	-	-
	CL1823	CTCTGATGCAATCGACGAGACT	AGCTGCACAAACCTTCTTCCAT	+	+	-	-
427	CL1751	GCGAAAGAGATTCCATCAGACA	TCCACAATGCCTTCTTTCTCAG	+	+	+	RS2CL1751s
	CL1752	GGAGGAAGAGATCATCAAAGCA	TGTAAGGAAAGGGGGTCAAGAG	+	+	-	-
	CL1773	ATCCACTCCTCATTTTCCCAGA	GCTCCAACAGCCTAAGAGCAAT	-	-	-	-
	CL1791	AAGACGAAGTCTGCATTTGTGC	AGTCGCAGTAACACGTTTTTGG	-	-	-	-
	CL1793	GGATCGTTACAACGCATCTCAA	AGCGTAAACCAGAGCTCTCACA	+	+	+	RS2CL1793s
	CL1803	AAACTGACAACCTCCCCAAATC	AGGAAGCCAGACATAAGCAAGG	-	-	-	-
428	CL1763	TGTTGGATACCAGAGGGAGGTT	GCTTCTAGCCAAGGGTTGTTTG	+	+	-	-
	CL1785	TTTCTGGCAACCGTAAGGCTAT	AGTTTCCTGTACACACCGACCA	+	+	-	-
	CL1792	CAAGTACGATGCCAAGTGGAAG	GAACCCACCAACAAAACAACAG	-	-	-	-
	CL1796	CTAAGCATTGCCAAAGCACAAC	GAAGACCCCGCTTCTGTCTAAA	+	+	-	-
	CL1802	CGGAAAAGCACAGATGAAACAC	CGAATCGATATGGGAAGAAAGC	+	+	+	RS2CL1802s
	CL1806	AAGGAGCGTGTTGAGGAGAAAG	ACAAGAACCACCCCTAAGAGCA	+	+	-	-
429	CL1750	CGAGAAGGTTGAAAGATGGAGA	AGGATGAAGCCTTAATGCCAAC	+	+	-	-
	CL1753	ACAGCAGCAACTCACATGAACA	AGGGAAAGATTCACGATCCAAG	+	+	+	RS2CL1753s
	CL1765	AGCTTGAACCTTTCTTGCCGTA	GGAGGCTCGTTCTATCTTCCAA	-	-	-	-
	CL1772	AGTCCATGATGACTGCAAGCTC	GTGCCGTCACAGCAAACTTTAG	+	+	-	-
	CL1778	TCAGCTGAAGAAGCTGATGACC	AAGCTACCGCCTCATCAAACA	-	-	-	-
	CL1826	ACCACCACCTCTAAAAGCAAGG	CTCACAGGCAAAGAGATCGAAA	+	+	-	-
430	CL1797	GAAGAGAAGCAATGGCAGGTTC	CACCGGTTTTCCTGCATACAT	-	-	-	-
	CL1798	ATCCAATATCGCAAAGGTGGTC	CAAATCGTCCCATCAAGACAGA	+	+	-	-
	CL1800	ACATGTGAGACGGCAATACAGC	GGCCAAAGCCAAGTGATCATA	+	+	-	-
	CL1801	ACTCAACCCGAACCAAGAAGAA	ACACAAACATGCACGCTACACA	+	+	-	-
	CL1812	GCAGTGGTCGTTAAAGCTGTTG	ACTCTGGTCTCCCAAATCGAAA	+	+	-	-
	CL1817	CATGGACAGATGGGGTATGTTC	ACAAGTGAGGCAATGCAGTCAT	+	+	+	RS2CL1817s
431	CL1761	GAGAACCGGCTTATCTGCAAAC	GCTTACCATCAAGCGACCAAGT	+	+	+	RS2CL1761s
	CL1771	CTTGGAGCATTGGTCTATGCAG	TCAGATGCCGTTCAATCGTACT	-	+	-	-
	CL1779	GTGATTGGCTTTGTCTTGTGCT	CAAAGCCTCTCCTCTGATTCAA	+	+	-	-
	CL1794	CTTCGGAGAGAATGGAACAACC	TGTCCCAGACCATGAAACAAGT	-	-	-	-
	CL1818	TTGCTATCCTCTTGCCTCCTCT	CAGTGCAAACACAACAGCGATA	+	+	-	-
	CL1820	TTAGTGCTTCTGCGATGTAGGC	TTCCGTTACCGTTCTTCTCCTT	+	+	-	-
432	CL1840	ACCCAGATTTGCATCTGGTAGG	AACGGTACTTGCTTCCAAACTG	-	+	-	-
	CL1848	AGACTGAATATCGCATCGCACT	CTTGAAACACACATGGGCAAAG	+	+	-	-

	CL1860	ATGGATTTCTCGTCGGTGGT	CCCTTTTTCTCACTGTTGCAGA	-	-	-	-
	CL1883	TTCTTGCCTCTTGATCTTCGTC	TTAACACAAAGCCCGAGAACTG	-	+	-	-
	CL1893	TCACAAGCTCGAAAGAAAGTGG	TGTCTAAGCAGGCTTCCTGTTG	+	+	-	-
	CL1894	CAAGCTTCCATTGTCTGAAGGA	AGCTGAAGTTGGAGTGGTTTCC	+	+	+	RS2CL1894s
433	CL1833	AGTGCAGGTTGTGTATGGGAAG	TGCAGGAAGCTTACTCCTTCAA	+	+	+	RS2CL1833s
	CL1884	AACCGTTCTGCTGAAGTTTTCC	GACAAGCCGGTTGAAGCTAATC	-	-	-	-
	CL1891	GAGCACCGTTTCCTCAGAACTT	TCTGTAGAGGCGCCAGTTCTTT	+	+	-	-
	CL1924	AACTCGGGAAAGGTGATAGCAG	AAGACGAAAGGAACGCTCGTAG	-	+	-	-
	CL1926	GTGCTCGAGTTCTTGAGAGCAA	TCCAGGCACTTGTGATTTCAGT	-	-	-	-
	CL1943	ATGCTGGTGTCATTGTCAATCC	CAAAGCATCCTCAACAGTGACA	+	+	-	-
434	CL1852	AAAGCAGAGAAAGGAGGTTTGC	GCATCACCAACGAGAAGACAAC	+	+	-	-
	CL1862	ATATTTCACGAGGCCTTTGCTC	CGTGAAGACAGAACCAGCTTTG	-	-	-	-
	CL1899	TCAAGCACAGACATTCCAGGAT	TTCTTGATGGCTTCCCTAGGAC	-	+	-	-
	CL1908	GTCATATACGACATGGCCGAAG	TTCTTTTCTTCCCCTGCTTCCT	+	+	-	-
	CL1910	CACCAGAAGGGCAAAAAGATTC	CTTTGGCAGATTCAGCTCTGTT	+	+	-	-
	CL1919	AAAGAAGGAAAGCGCCACAAC	TTAAACCCCTGGGTACCAGCTA	+	+	-	-
435	CL1829	TCATTCGGTAATCGCATGTAGG	TCAGATACAATGGCTGGGTCAC	-	-	-	-
	CL1839	TTGTATGTCCAAGGAGCAAGGA	AAGGCTGTAAGGAAAAGCAACG	-	+	-	-
	CL1892	GAGCGAGGATCTCATTCGTTTT	GAAGGGATAATCGGGAGGAAAG	-	+	-	-
	CL1905	TTCTCGGTCTTCTGAGCAACAG	GCTGAGTGAGAAGGTGAAAGCA	+	+	-	-
	CL1932	GGTAGCAACAGGACATCGCATA	GAGGCTTACAAAAGTCCGGTCA	+	+	-	-
	CL1936	AACGAGTTCCATGACAAGACCA	TATAGCAACATGACGGACACCA	+	+	+	RS2CL1936s
436	CL1836	GAAAAGACAAGAATCCGGTGGT	AGAAGCTTGTCTTCCCATGAGC	+	-	-	-
	CL1844	TCATTCATCAGCGTCTTCGTCT	CTAACTTTTCCTTGGCGGCTTA	-	-	-	-
	CL1898	GGCTTTTGCTTAGGCTGAATCT	AAGATTAACGGTGGCTCCATTG	-	-	-	-
	CL1907	TGGTGAGGAAACTGAAGTTGGA	TTTAACTACCGCGTGCTCTTGA	-	-	-	-
	CL1918	AAAGCAGCTGACTGGTGTGCTA	TCAAATCCACAGTTCAGCAAGC	-	-	-	-
	CL1922	ACTTTCGTCTACCTCGCAAAGC	CGTTCCCTTTCACTGCTTCTTT	+	+	-	-
437	CL1957	GAACCAGGTTTTCTCACGGTTC	ATATTCATACCGCAGACGCAGA	+	+	+	RS2CL1957s
	CL1900	CTCCCGTAGCCATTGTCAACTT	ATGCTTGAAGCGTTGGGTATTC	+	+	-	-
	CL1912	TTCCCTCTCTCCTCTGCCATAA	ACCAGATCAAGGAGTTGGTCGT	-	-	-	-
	CL1923	CAACCAGACTAAGAAGCCCAAA	GAAAAGATGCCAGCAAGGATG	+	+	+	RS2CL1923s
	CL1944	TGGACTTGACACCAAGATTGAG	CCATGTGCTTTACCAATGGATG	+	+	-	-
	CL1951	AGGGGACAACATCAGAACCAAT	ATGGTGTAACTGAAGCCGTTGA	+	+	-	-
438	CL1834	CGGCGTCTGAAAATGTTGACT	TATGCCTGCTGACATCCTTCAG	+	+	-	-
	CL1835	ACAAACCTGAATGTCGAACAGG	CCCAAACACCTGCATAGCATTA	+	+	-	-
	CL1849	TTGATATCGAACCAACCGACAC	AAACACAACCACCACCCCTAAG	+	+	-	-
	CL1888	AGAACACATAAGCAGCGTGGAG	CGAAATAAAGCGCCTCCTTGTA	-	+	-	-
	CL1889	ATCATGATCACACCCATGATCC	GCTCCTCACGGTTCAGATTCTT	+	+	-	-
	CL1911	CGTCATCCCAGTTCGCTATCTT	CATTGTATTTGTTGGGCTCCAG	-	+	-	-
439	CL1952	TCCTCAGCCTCCTTTAGGTTCA	AGTTGGGTCAACTCAGCATCAA	-	-	-	-
	CL1954	GGTGAAGAAGGTGAACGTGACA	AGGACCATAGACATCAGCGTCA	-	-	-	-
	CL1967	CAGCTAGAAAAAGAAGCCGACA	TCAAACTGGTGAAGGAAGATGG	+	-	-	-
	CL1987	CTGTTTTTCTGCTCGTCCTCAA	CAGCCCTGTGGGAGAAAATAAG	+	+	+	RS2CL1987s
	CL2002	TCCAAGTTCATCTCTTGGAGGT	TTGTGGAGAAAGCTGAGCAAGA	+	+	-	-
	CL2033	TGTGGAAGCTGGTTACGTCAAT	ATCTTAGAACCAGCGCGGATAG	+	+	+	RS2CL2033s
440	CL1953	CCTCACAGTGAACTTGGCAAAC	CATTCACCGAACTGGTTCTGAC	-	-	-	-
	CL1969	CAAATCGCACTTTTGAGAGCTG	TATTCAAGGGAGATGCTGCTG	+	+	-	-
	CL1984	CGAAGATCACAGAGGTTGATCG	TGAGTCCGCTTCTTCTTCAATG	+	+	+	RS2CL1984s
	CL1993	GGAAAAACGTAGGGAAACGATG	TCCCACTTCCATGACTGGTTTA	+	+	-	-
	CL1996	CGCAACAGTTCCACCTTGTTTA	CGAAAGAGTGTTTGACGAGCAT	+	-	-	-
	CL2034	TTTTTCAGAGGAGTCCATCACG	AAGAAGGGTTGGGTTCTCTTGA	+	+	+	RS2CL2034s
441	CL1966	AACACGAGCTGTGGACATGTTT	GAAGGAAGCTCTCGGTGATGAT	+	+	+	RS2CL1966s
	CL1974	AGCTTTGTCTCCTCCTCCTGTT	ATGGAGCTCTGATTTGGCATCT	+	+	+	RS2CL1974s
	CL1981	GGTCCAATTATTTTCGCTGGTC	AAACACGATCGTCTCCTGTCCT	+	+	-	-
	CL2012	GGACGCATGTAACAACTCTTCG	CACTGTTTAGGTCCCGATGTCA	+	+	+	RS2CL2012s
	CL2028	TTAGCACTTTCTTCCCCCAAGA	TAACCCAAGAAGCACGACTTTG	-	-	-	-
	CL2037	CATCGATGACAATGACAGCAAC	AGGTGGGACGGTTGAATCAGTA	+	-	-	-
442	CL1957	GAACCAGGTTTTCTCACGGTTC	ATATTCATACCGCAGACGCAGA	+	+	+	RS2CL1957s
	CL1962	AGCTTTTCAGCTTCCTCCAAAC	AGTGGTTCTTGACACTCCCTTG	+	+	-	-
	CL1973	TGTCGTCATCCAAGTTCGCTAT	TTCCTTTCTAAGGACAGCTCCA	+	+	-	-
	CL1995	TAAAGGACGTAGCCAATGCACA	CCTCAAATGCATTCTCAGACG	+	+	+	RS2CL1995s
	CL1997	TCAAAATCTCCGATCGAACTCC	CTTCTTGGCGAGCTCTTTGATT	+	+	+	RS2CL1997s
	CL1999	CGTACGAATACCTCCAAGATGC	ACCTTAACTTCCGGTGTTGCAG	+	+	-	-
443	CL1955	TGGGAAACCACCCTGAAGAT	GTTCACGTTGATGGAAAGCTCA	-	-	-	-
	CL1958	ACTTCAACGCCGTTTCTTCTTC	CGTTTGTTCTTGACCTTGGAGA	+	+	+	RS2CL1958s
	CL1980	TCGTTCGTGTTTCTTGTGTCCT	TTGAGATCTCTCGTCCAGTTCG	+	+	-	-
	CL2001	GATTAAAGTCCACGTCGGCATC	CCACGTTGCTTAATCACAATGC	-	-	-	-
	CL2022	ACCAAACCAAACCAGCAAAGTC	CAACCTCTTGACCAGCCTTCTT	+	+	-	-
	CL2039	GCTTCTTTAACACCCCTCCAGA	CCTTGGAGTAGCGCTGATTCTT	+	+	-	-

444	CL1970	GACGTTGGAGGCAAGATCTCTA	GCTGTCCAGCTAGCCAAAATCT	-	-	-	-
	CL1971	TCACAAACGATCTGGTTGACTC	CCGGAGGACCTCTGAATAAGAA	+	+	+	RS2CL1971s
	CL1977	CGATGAACTTGGAATCCCTTTC	ACAACCGAGCTTAGTGTTTCCA	+	+	+	RS2CL1977s
	CL2003	AAGTCTTGTACTGGCGCTCTCA	GCATTCGCACTACAACTCATCG	+	+	-	-
	CL2010	AAAGGAACATGTGGACCGAGTT	TCGCCTAAACCCATCTTCTCTC	+	+	-	-
	CL2031	ATCCTCATTTCCTCGTCTCTGC	CCAGGAATCATGACAGAAGCAC	+	+	-	-
445	CL2045	TGGGACCAAAGTGTCGAATATG	TTAAGCAGCAACGTCTTTGTCC	+	+	+	RS2CL2045s
	CL2074	CTGACAAACTAAACCGGGATTG	AAGTCGAGCCTACGAACAGGAT	+	-	-	-
	CL2079	AGGAGTTTGCCCAGTTTCAAAG	TCGAGAGAAAACCACAATGGAG	+	+	+	RS2CL2079s
	CL2089	CAACCTGAAGCCAAAGGAAAAG	GAGCCATGGTTGAATAACCACA	-	-	-	-
	CL2092	AACAGTGTCAGAGCCGAAACAA	TGGACAAGCTCTGCTTGAACAT	+	-	-	-
	CL2093	GAGTGTTTATAACGCCGCTCGT	CTGATTAGCCCATGCGAGTTT	+	+	-	-
446	CL2040	ATCGTAGCTGTGCATCAATGGT	CCTAAATGCGACGTTGTAGTCG	-	-	-	-
	CL2052	GTACCCACCCTTCACAATGGTT	TGGTTGGAGTCCTCCTTATGGT	+	+	-	-
	CL2058	AACGGTGATATTGGTCCTTTCC	ACACAGATAGCGGCTGAGACAT	+	+	-	-
	CL2077	GCAGACAAAACTGGGATTGTTC	GTTCGCCTACACTCCCAAAAAC	+	+	+	RS2CL2077s
	CL2097	GGTCCTTGGCTTCCTTCACTAA	AGAGGGAATCTGCAGAGGCTTA	-	-	-	-
	CL2126	CCAAGGCTTTTGCTGCTCTTAT	CAATGCTGTCTTCTCGAGCTTT	+	+	-	-
447	CL2051	TTCTCCTCTGCTTCAATCACCA	ACGAGCCTCCAGCTAAACTTCA	+	+	+	RS2CL2051s
	CL2056	GTTGCCAACCATACTTCGATGA	CTTGCGACAATTTCACGATCC	+	+	-	-
	CL2060	GGACCCAGAAACTCTTGCAAAC	GGGTTTACCAAACACGGGAATA	+	+	-	-
	CL2066	TTCCTGTCGGAACAACAATACG	ACAACTGCTCCAACGTTCTTGA	-	-	-	-
	CL2076	TAGCTTTCTCTTGGGCTCCATC	TTTGCAGATGGATTGGTCTCTC	-	-	-	-
	CL2082	GTTCAGAGAAGAGCAGCATCCA	GTGAACAACACCGAAAACTTGC	+	+	+	RS2CL2082s
448	CL2048	AATGGCTAGCTTCACTGCCTCT	CCAAACACTCCCAAAAGAATCC	+	+	-	-
	CL2059	TGTTCTCTTCCTCCCTCAAAGC	CCTCTTGTCGGATCAAGGAGTT	-	-	-	-
	CL2068	TGTTGGAAGTACCCCTGACTGA	TCGTACTGTCATGACTCCAGCA	+	+	+	RS2CL2068s
	CL2081	ATTCAAACGGAGACACACCGTA	GGACAGACTCCTCTTGCGATTT	+	+	+	RS2CL2081s
	CL2120	TTCAAACTGTGGATGTGGTTCC	CAAACACACTTTTCCCCCAGTT	+	+	-	-
	CL2128	CTGTCGCTTTGCCTAGCTTTTC	CAAAGCCACCAACATAAGGACA	+	+	-	-
449	CL2062	CTGGTTTTTAGTTCGCCCGATA	ACGCAGGACATTGACGATAAGA	-	-	-	-
	CL2063	AGGATGAGGATGATTTGGAGGA	ACCATCTTCATCTTCGCTTTCC	+	+	-	-
	CL2101	CATGGCATCTCCTTCACTTCTG	TCTTGTCTTGACAGGCTTCTGC	+	-	-	-
	CL2106	CTGAAGTGCTTTTGTTCGGTTG	CTGGTTAGATGGAAGGCATGTG	-	-	-	-
	CL2111	GTGGCAAAGCTTCAGTATTTGG	CTGCTGAACGAGCATTCAAGAT	+	-	-	-
	CL2117	GCGACAAATCATACCCTCACTG	TTGAAATCGTGTGGCTGTATCC	+	+	+	RS2CL2117s
450	CL2182	CTACTTCCAAGGGCTGTTTGCT	ACATGAAGAGCAGAGCATCCAA	-	-	-	-
	CL2183	CATCCGCTTTGTCATCTTGGTA	GTGTTACCATGCCGAAGAACAA	+	+	-	-
	CL2199	GTGCAGAAGATTTGGAGAAGGA	ACTGCATCATCAACTCCAGGAA	+	+	-	-
	CL2200	AGAATCTCGTGTCCCATTGTTG	TGGTACACGTTGCCTCTGAAGT	+	+	-	-
	CL2236	ACCATTACCATGTTCGTCATGC	TGATTCCCTTCCCTGGATCTAA	+	+	-	-
	CL2244	TCGAGATTGGTGTGCATAGTCC	ATTGGCCAGGACGTTTTTGTAG	+	+	-	-
451	CL2143	TTGATGACGCGATAGCAGAGTT	TCGACTCGCCAGAAATATGATG	-	-	-	-
	CL2178	GGGGTGCGTTATACGAAGTTGT	TCCAGAGCATGATGTTGTTGTC	+	+	+	RS2CL2178s
	CL2189	AACTCGCGATCCCAGTTACTTC	GAGAATTACTCGGTGGGAATGG	-	-	-	-
	CL2197	AAGTCACTTGAGAGCAGCAACG	ACTACAAAAGCCTCACGTGCTC	+	+	-	-
	CL2206	CAGCTTCCTCACACAAGATTGG	GGAGCAACCGCTAAAACCACTA	+	+	-	-
	CL2249	AAGCCGTCTTGCCCATTATAGA	CACAAACGGGATGAAGTCAGAG	+	+	-	-
452	CL2180	AATGGGAAGTGACTGTGGGATA	ACCGGTACGTAGATCTCTGGAA	-	-	-	-
	CL2184	GTGTATCCAAACAGCGGAGAAA	TTGGAGGAAGAAAGGCTTAACG	-	-	-	-
	CL2188	AGCAGGGTGATTCTGAACCCTA	CAACAACCCCACAAGAGTCATC	-	-	-	-
	CL2214	CTAACCAGACAACAACCCACGA	CTGCACTTTTCGCTTTTGGAG	+	+	-	-
	CL2240	CTGCAGATCCATCTCCACAAAT	ATGGAATCTTCACCAGGAGGAA	+	+	+	RS2CL2240s
	CL2254	CCTCTGATTTCTCAACCAGCAC	AGCTAAGCATCACGCTCCTTCT	+	+	+	RS2CL2254s
453	CL2145	GGAACACCACCACTTGGATGTA	AAGCTCTCTGACTGGGATGGAT	+	+	-	-
	CL2194	ACTCAAGCCGAAAAGGCTATCA	ACGTTTGATGCTCCAACAAGAG	+	+	-	-
	CL2211	TGCCACAAACTAACCACGAAAC	GAGACTCCATTGATCCGAAAGC	+	+	+	RS2CL2211s
	CL2213	TGGAAACAGTAGGCACAAGATG	CGACGCAGTTTTAGCTGAGTGT	+	+	-	-
	CL2220	AGGAGGAAAATGACGACGAGAC	ACATCGATCAACGGAACCTTGT	+	+	-	-
	CL2226	ACCCAACAGAGGAGGAACTTTG	TGGGAGGAATGTTACAACAACG	+	+	-	-
454	CL2150	GGTTCACTTCTCCCTCTCCAAA	ACAGCATCCCTTTTATCCATCC	+	+	-	-
	CL2186	CAAAGACCCCAAAGAAAACACC	ATCGAACTCGTTACGCTTAGCC	+	+	+	RS2CL2186s
	CL2192	GATGTGCTGCAGATGTTCTTGA	GAATCACTTTCCCCAACAATCC	+	+	-	-
	CL2203	TAAAGGGCACAGTCAGCATCAT	TCTCCCGGAGTTCAAACATTTC	+	+	-	-
	CL2210	AAGATTTCACAGCTTCCCAAGG	GTGACATGAATCCTCCCACAAA	+	+	+	RS2CL2210s
	CL2217	GATCTGGTGGTGATGGTTTGAA	GGGAAACAAGAGGTGTTCAAGG	-	-	-	-
455	CL2157	GTCAATCCCACGAGCCTCTTAT	GGCTCCAATCCAAGTTGTTCA	-	-	-	-
	CL2167	CTTTGTCTCCAATGGCGTTC	GTTAACAAAGCCGCCGATCTT	-	-	-	-
	CL2171	AGAGCGTGGTAGCGATGTCATA	ACATTAAGCTCACGAGCCTTGG	+	-	-	-
	CL2196	CATGGGTGGAATGGAAGAGTTT	TCTGGAACGAACAGCAACTTCT	+	-	-	-

	CL2219	AGTTTCCTCTGGACGACCAATC	CGTAAAGGGATGGTCACAACAA	-	-	-	-
	CL2227	TCATAGTGCAAGGTGGTGATCC	GGAGCAGGTGCTAAAGTGATGA	-	-	-	-
456	CL2264	CATGGGTGAGAAAGGTCTGATG	GAAAACAACCGGGAACATCACT	+	+	-	-
	CL2268	TTGAGGCCTGCTGAGTACAAGA	GAAACGTGAAATGTGGACGAGA	+	+	-	-
	CL2280	GATCTTCGTGAAAACCCTGACC	TAGTCCGCAAGGGTTCTACCAT	+	+	-	-
	CL2289	TGAAGCCGTTGCTTTCTACAAG	CTTAACTCCACCGTCAGCTTCA	+	+	+	RS2CL2289s
	CL2302	GACAGCCACATGATGGAAACAG	ATAGCGTGAACTCCTGCAGACA	-	-	-	-
	CL2348	TCCATGCGTGGAAGACTTTG	CAATCGTCGGATCAGCAAATAG	+	+	-	-
457	CL2270	TGCGTCGTCTAGGTAGCTTTGA	TCTCTGCAGGTGGTATCCATTC	+	-	-	-
	CL2290	CAAATGGAGACTCAAGCCCTCT	CGGTCTTAAAGCTCAGGCAACT	+	-	-	-
	CL2293	CTCGGAATCAAGCCAGACAAAT	GTCATCAGGGGGACAAAAGGTA	+	-	-	-
	CL2299	AATAAAGTAATGGGGCGTGTGG	AATCACACCAGGAAGGCTAAGG	-	-	-	-
	CL2331	TGAAGGCAAGCAACACGGTAT	TTTGGACTTGGTGATGGGATG	-	-	-	-
	CL2335	CAGCAGGAGCTGACTTCATCAT	GTCTTGGTCCATGCAGTTCTTG	+	-	-	-
458	CL2283	TAGCTTCCGAGAACGAGAATCC	GGTAGTATCCGTCATCGGGTTT	+	+	-	-
	CL2317	ATCCGGTGTGACTGAAGCAGTA	GATGTTGTGGACTCCCATTTGA	+	+	-	-
	CL2323	AACCAGTGCTTCCAGGATGTTT	GTGAGGAGCGAGTTGTTTGATG	+	+	+	RS2CL2323s
	CL2336	ATGGAGGATCCAGCATGTTTC	ATGTCTCTCCTTGGATCCACCT	+	+	-	-
	CL2337	CGTTCGCCAGACGTAATGTATC	TCTGTGACTCATTTCCGCTTTC	+	+	+	RS2CL2337s
	CL2352	CGATCACGACAATCTCTTCGTC	AACCCAGAGAGTTGTTCCCAGA	+	+	-	-
459	CL2358	CCATCAACATCCATTGCCTCTA	GCAGGTTGTAAAACCATGTGGA	-	-	-	-
	CL2376	GATACCTTGCCTTCCTTGGAGA	CTACTCGTTTCTTTCGCAATGG	-	+	-	-
	CL2284	CCCTCGATTCCCAAACTACTTG	TCAGTGAGAGGCTGCTTGTCTT	+	+	+	RS2CL2284s
	CL2310	GTGGAAGTTGATTGGAAACCTG	GCGTAGCGTTAATTGCTCCTCT	+	+	-	-
	CL2329	CACGACCATCCACTCTTCAATC	GATTTTAGCTACACGCCCAAGG	+	+	-	-
	CL2341	AGGCTCCTGACCACTGAAGAAA	CAGTCTGTTTGGTGCTTGTGTG	+	+	+	RS2CL2341s
460	CL2263	GGAAATTCAACCTGAAGCTGGT	CGCATGTCTTCCATTTCTTCAC	-	-	-	-
	CL2267	GGGAATTTGCACTCCAGAAAGT	AGATGCTCAATGGTGTCGATGA	+	+	+	RS2CL2267s
	CL2271	GCCTTAAAGCTTGCTCCAGATA	TAAAACGTCTCCGGCTAAGCA	+	+	-	-
	CL2272	AATCTGAAACCCCAGAACAGGA	ACTTGAATGCGTCTGCAACAAC	-	+	-	-
	CL2311	AGGCGTACAAATCACAAACACG	AAAACGAAGAAGGCACTGGAAG	+	+	-	-
	CL2338	CTTCTTGTTCCCAAACCTCCAC	GGATCCACCTTTAAATCCCACA	+	+	-	-
461	CL2367	GCGATGGCTTCCATTACTTCTT	ACACAGCCAACCAACAAACAAC	-	+	-	-
	CL2428	ACACTTTGGTGAAGCCTCTTCC	CAGCGTCCATTCAAATCTCAAC	+	-	-	-
	CL2452	GACCCGACAGCTGAAGAAGAAT	AAAATGGTGACGACGAGCACTA	+	+	-	-
	CL2454	TTGACGAAGCTCAGTTCTTTGG	AGCTCTTGCCTTGTCAGAATCC	+	+	+	RS2CL2454s
	CL2465	ATTCGCAATTTCTAGGCGAGAC	CTTAAACGCTTGAAGCAACGTG	-	-	-	-
	CL2466	GTTGCGCTGCTTGATGAATAAC	CATGCGCATAGACGAAACTGAT	+	+	+	RS2CL2466s
462	CL2355	TCTGTATCTGACGAACGGGAAA	ACAAGCATCATCATTGGGACAG	-	-	-	-
	CL2362	ATCTCGACCCCGAGTCTGTTTA	TTTCCCAGACCCATCTCTCAGT	+	+	-	-
	CL2372	CGGAAGAAAGAAAATGGGAGAG	CATCAAAAGCACGAGAGCAATC	+	+	-	-
	CL2399	CGTGAGTTTATCCATGCCAAAG	TGGACTCTTTAAACGACCACGA	+	+	+	RS2CL2399s
	CL2418	GGGAAAAAGTTCTTAGGGCACA	TGCAGTTGTAAACGGGTTCAAG	+	+	+	RS2CL2418s
	CL2443	GACGCTTCATCTTGTGTTGAGG	TTCTTCCTGCAGTTCACAGCTC	+	+	-	-
463	CL2370	AATGGAGGGGAAGAAGATAGGG	GAGCATTGGAAACGTGACAGAC	+	+	+	RS2CL2370s
	CL2386	GGAAGAGGAAAAGGAGGAAAGG	AGTAGCAAACAGCAAACCGAGA	-	-	-	-
	CL2391	GACGTCTCCACCAAGTTCTTCA	TTTTGACGCTCCTAGAGGGAAC	+	+	+	RS2CL2391s
	CL2427	GCGGAAACTCTTGAGCTTTTGT	CATACGCCAACCACCATTGTAA	-	-	-	-
	CL2437	GAAAAATGGCTAGCTTCACTGC	CGAGAGATGAAGTGTGGGTGAA	+	+	-	-
	CL2455	AGAAACCTCGCCAAAGAAACAC	AAGGAACATCTCCGACCAGAAC	+	+	-	-
464	CL2381	AGGAAGAGATAATGGGGGTTCC	GCGCATAGCATGTTTATCAAGG	-	-	-	-
	CL2383	ATGGAAGTGTTGCGGGACTTAT	ATTCGTGTAGCCTGCTTCCTTC	+	+	-	-
	CL2412	CGTCTCTCCTCCGATTTCACTT	CTCGAACAATCCAGTTCCACAG	+	+	+	RS2CL2412s
	CL2417	TCTCTGTTCTTCCCTTCCGACT	TCCCATGGCAAGTAAACCCTTA	-	-	-	-
	CL2448	CAAGCATCCTGATGATCTCTGC	ACAGGGTGTGAACAAGGGTCTT	-	-	-	-
	CL2450	TCATCTTCTCCGCTGACTTCAC	ACTGCACTCCGGAGAGTTTCTT	+	+	+	RS2CL2450s
465	CL2368	CCCTAGCAAAGTTTTGGAGCAT	ACGTTGGTACAAATGAGCCTGA	-	+	-	-
	CL2380	TGGAGGAACAAAGCACACATCT	GCTATCATCAAGTTGGCGAGAA	-	-	-	-
	CL2393	GAACTAGAGCAGGCCATGAAGA	TAAAGAGGGAAGAGGCAAAGCA	-	+	-	-
	CL2406	ACGAAGTGAAGTGCAATCCAAG	ACACTTCCAAGACATGGCACAC	-	+	-	-
	CL2430	CTCAGGAAGGAGGATCAGGAAA	GGATGAGCAGATTCAGTGGCTA	-	-	-	-
	CL2447	AGAACGCAGCAAAAGAAGAAGG	GCAAGGCAAAGATTCCAGAGTT	-	-	-	-
466	CL2374	AAGAGAACCTTGCCACCAGTTC	TCTTCTCCTCCATTTTCCCTCA	+	+	+	RS2CL2374s
	CL2410	GTTTTGGTCCTCGAGATCCTTG	GCGTTCCTAGGAGCTGTTCAAA	+	+	-	-
	CL2429	ACGCTCAAGAAACGCAGTGATA	CGTCAATGGAGAAAACCCTAGC	+	+	-	-
	CL2433	CTTGTCATCATTACCGGCTACA	GATAGCCGAATGCCCATTTT	+	-	-	-
	CL2460	GCCTGCTCATGGAAAAGAAAGA	GAAGAAGAGGCCTCAATTGCTC	+	+	-	-
	CL2464	AACAGATGTCCGATCCCAGAGT	AGATTTAGCTCCCTCCCCATC	+	+	+	RS2CL2464s
467	CL2475	AGGGTAAGATTGTCCCTGAGCA	GTTTCGGATCCGTTGTTGTTCT	+	+	-	-
	CL2508	GAAATGTTTGGCTGAGATGGTG	CTTTCAAAGCTCCGTCTTCCAT	+	+	+	RS2CL2508s

	CL2528	GCGTTTGTTGGATGAGTTTACG	CGATTTCGGTTAGGTTCGGTTA	+	+	-	-
	CL2534	AAAAGCCGCAGCTCTCTACACT	CGGGTTGAGTTTGATGGTTGT	+	+	+	RS2CL2534s
	CL2545	CCCAGATTCGAAAGAAGGTAGC	AGAAACCAGAACCTGAGCCAGT	-	-	-	-
	CL2555	GAGAAACGGTGATTGCACAGA	TACACCTTACTGCGCCAAAAAC	+	+	-	-
468	CL2471	GCGGTGAAGAAGATAGGAATGG	AAAAATGGATGAGCCCACTCTC	+	+	-	-
	CL2483	TGGATTCTCTGTCCTTGTTGGT	AATGTAGGTCGGACAAGGGAAA	+	+	-	-
	CL2493	CCACTGTCTGCTGTTGGTTGAT	GACAGTGAAGAAGAGGCAAACG	+	+	-	-
	CL2509	ATTAGGACACACGGGTAACTGC	GGGTTGAATGAGAAGCAAGTTC	-	-	-	-
	CL2557	ATGGGAGGTGAGCATTTGAAAC	GAGGAGGGAGCAATCAGAAACT	-	-	-	-
	CL2564	GGTTATTGACGGATGACGCTCT	TCCAGGCTGAACAAATTAGTGG	+	+	+	RS2CL2564s
469	CL2478	AATCACTCACTTGCCCAGATCA	CCTTGGCTTGTTTACAGCTTCC	+	+	+	RS2CL2478s
	CL2490	CCCAAGCGATGGAATACATAGG	AGGCTGCGTCTGGTTCTAACAT	+	+	-	-
	CL2514	TATCCAGGAAAATGCCCCAGT	TCAAGTCGAAACCCATACAGTG	+	+	-	-
	CL2522	CTCCGGATTCAAGTGAACAAAG	TTTCTATCAACACCGCACCAAC	-	+	-	-
	CL2562	CCCATCAATTACAAGGTCAAGG	AGCAAAGTCGAAGATGGATGTG	-	-	-	-
	CL2563	TGAATCTAGCAGAGGACCTCCA	AGCACTCCTTAAACCGATCAGA	+	+	-	-
470	CL2498	ACAACGCAGAAAGTCTCCCTCT	ATCGGAGTAGCCATTGGACCTA	-	-	-	-
	CL2501	TTGAACTTCGACTTCGTGCTGT	CATGGATTGAGAAGGCAACATC	-	-	-	-
	CL2518	CCATCTTCTCCAATCAACACCA	GAAAGGGATGGTAGGAATGCTG	-	-	-	-
	CL2530	GTGTTCCAATGTCGTGATGGAT	CTGCTTGTCTGTCTCAACTTCG	+	+	+	RS2CL2530s
	CL2552	GATGTCGTTCGCGATCAAGTAG	AAAGCGGACCGTACTAAAACCA	+	+	-	-
	CL2554	CACCATGCTTCTCTTCTTGTCG	CAAGAGTACATGGCATGCAACA	+	+	-	-
471	CL2473	GATTCAAACCCCAACGACAAAC	CCTCGTAAGCTTCACCAAGATG	-	+	-	-
	CL2477	GGCGGCGATAACCTATAGAATC	GCCTGCCGATGAAATGAAATAC	+	-	-	-
	CL2497	GAGGCACACCTTCAAACACCTT	CGTCATCGTTAGATGCGAGAAG	-	-	-	-
	CL2504	AAAAGCGACGTCTACTCCTTCG	TTTGCTAGAGACAGGGGAAACC	+	+	+	RS2CL2504s
	CL2510	AAGCAAAGGAAGAGGCTGAGAA	ACTGAGATCCCCATCCCAGTAA	+	+	-	-
	CL2527	TCCACCATCACTTGTTTCATCC	ATCCACATAGCATCGCTGAAAG	+	+	-	-
472	CL2610	ATTTCTGGTGCCAGGAGAGTTC	TCAATTTGTACGCAGCCTCAGT	+	+	+	RS2CL2610s
	CL2654	TAGGTCCGGACTCGGTTAAAAA	ACATCAGTTGGCCTCTCATCAA	+	-	-	-
	CL2656	GATCGATGGAAGGGATCGTTAC	GCACGTGATTCAGCATCTTTCT	+	+	-	-
	CL2665	GCCTTATGATTGACCCGGTTAC	GCAGCCTGTTTCAGACAATCAC	-	-	-	-
	CL2674	TCTCAGATTTCCCTCAACTTGG	CCGAAAAGCAAACTCCAGCTAA	+	-	-	-
	CL2676	CCATCTTCACCTCCACTTGAGA	CTGTAAATGCCGTCTCCATGTC	+	+	-	-
473	CL2607	TCCCTTGTGTGGAAGACTTTGA	TTGAGCTCAGGTAACCTTCCAA	+	+	-	-
	CL2609	GGAAATCTCCACCTGCTTTGAA	AAAGATGGTCGAGTCCATTGCT	+	+	-	-
	CL2659	CAAACAGCAACAACAGCTCCTC	ATTATCGGTGTGCTTCGATGG	+	+	-	-
	CL2667	CAACCTGCAAGCCTAGAGATGA	TTTAAGCACCGTGTTCTGTGGT	+	+	-	-
	CL2669	ACAACGTGTGGAACTCTCATCC	TCTGCAGCAGTTGAGACCATAC	+	+	+	RS2CL2669s
	CL2679	AATCCACAAGCTCGAAAAGGAG	ATGTGTGTTGGCTTCTCAGCAT	+	+	-	-
474	CL2572	AGGAAGAAGGAGAATCGTGTGG	TGCCACCAGGAGGAGTAAAAGT	-	+	-	-
	CL2604	TGTCACCCCTTCCACTATAAGC	AATGAAGTCGGCGACAATATCC	+	+	+	RS2CL2604s
	CL2605	TGTGGCCATGGAAGTGATCTTA	CTATCTTCCGTGCGTGCAACTA	-	-	-	-
	CL2623	TGTGTCCGGTGAAGAAGAAGAA	TGCTGAGATCTCTTTCCCCATA	+	-	-	-
	CL2630	GAGAAGGCTATGGCTGTCACAA	TCCAACTCCTTTGAGGAGAAGC	-	+	-	-
	CL2673	CTTAGTGTATGAGCCGCCAAGA	TGCACTGTAACGATCCCATAGA	+	+	-	-
475	CL2589	TCGGAAACCGTTTGGTACAGAT	CTCACGAGCTTACGGTTTTGTG	+	+	-	-
	CL2637	TCGCAAGCTTCAGTTTCTTCTC	AGATCTCGAAGAGAGCCCGTAA	+	+	+	RS2CL2637s
	CL2655	GTCCGAGAAACCGATCACAGTA	GTTAGGCTGCTGCTCTATTTGC	+	+	-	-
	CL2666	AGAACGCCTACCGTTTATCAGC	GCATGGAAAGTTGAGAGACACG	+	+	+	RS2CL2666s
	CL2685	ACCAGCTCTCACATCAAGAGGA	CGTGTGGATTCACTTGACGAAT	+	+	-	-
	CL2691	CTGGCCTATCCTGCTTTGCTAT	AGCTGCATAGATCTGTCCGTCA	+	+	-	-
476	CL2593	GATTCTGTCGCTCCGTTGAGAT	CCCACCGAATCTGTAGTTGAAT	+	+	-	-
	CL2606	AAAGGCTGAATCCATGGAGATG	ACAAGACCGATATGGAGCACAA	-	-	-	-
	CL2612	GTGGTTCACCAAAAGACTGCTG	GAGGGTGCAAAACACATCCATA	+	+	+	RS2CL2612s
	CL2615	AGAACTAGCCGATCCATCGAAA	CTCCTTTTCGGAAGAGAACTCC	-	-	-	-
	CL2636	CGACGACAACCTCTTTGACAAC	AAGTGACCAAACACCGCTATCA	-	-	-	-
	CL2687	GAGCCAAGCATTGGAGATAAGC	TAACAGCACCGTGAGTGTTCCT	-	-	-	-
477	CL2574	GGGTAATGGTTCGAGGAGAGAA	CAGCTGTATCCCACAATCCAAG	-	-	-	-
	CL2596	ATGCAAATGGGTGGTGGATT	GATCACAAGCACCAGCATAACC	-	-	-	-
	CL2597	TGTAGAGGAAGCAACTGTGGTG	TTCAAGCGTAAGGGTTGACG	-	-	-	-
	CL2631	ATCCCACCATGAGTGAGGCTAT	TTCTTTGAGCTCATCCCACATC	-	-	-	-
	CL2660	AACATCACACGGAGATGGACAG	AAACCCAGGAACGTAAGGAACA	+	+	-	-
	CL2684	TATTCAGATAGACGGGCAGCAA	TGAATCCCATCCAATCCTCTCT	-	-	-	-
478	CL2697	ACCAAACCCCATGTTTCTGAAC	CGCCAGATCATTTTCCTCTTCT	-	+	-	-
	CL2709	TTGAAACTGTGGACTGCCTTGT	TGAGTAAGTGGCTACCGGATCA	+	+	-	-
	CL2718	AGGGATCTCCGATCTCAATGAA	GCTGCTGCTGTCATCCAAATTA	+	+	-	-
	CL2728	GGTCGTGTTCTGCGTTTCAGTA	TTATCTTTGCCTCCTCCTCCTC	+	+	-	-
	CL2732	GGGCATGTCATGTGTTCTATTG	ACGGTACTCGTCGAAGAACTGA	+	+	-	-
	CL2737	TTGGGGCAAATCGTGTAATG	AACCAGCAAGTTTCAGGCTCTT	+	+	-	-

479	CL2695	GAGACAACAAGAAGGAGGGTCA	TAGTCTGCATGCATGTCCATTG	+	+	-	-
	CL2702	CGACAGTCAGGAAGCTCAAGAA	GTTCCGACAAACGATCACTGAG	+	+	-	-
	CL2714	ATGAGAACGCGGAGGAGATAAA	CCCTTTAAAGTGGCAGAGCAGT	+	+	-	-
	CL2717	ATCGGTTTGGCAGTAGCTTCA	GCCTTCAAACTACGCAGCTCTT	+	+	-	-
	CL2731	CCGAGTTTAACAACATGCGAGT	TTGGTGACTCACGGGCTTACTA	+	+	-	-
	CL2733	AAAGGAGGAGGAGGAGAAGGAA	CCATGAATGGCTGAGAAAAAGG	+	+	-	-
480	CL2692	GATGTGATATGTCCGCACCAAG	CCCAAGAATATGAAACCCAAGC	+	+	-	-
	CL2699	ATGAAGAAGCAGAACTGCAACG	TGGCTTCTTCCTCTCTGATTCC	+	+	-	-
	CL2700	ACAAAGAGAGCCACAAGCATCA	GTTTAGCTGAATCCATGGCTGA	-	-	-	-
	CL2703	CGGATGGGTGGATATGGTCTAT	TACATGACACAGTTTCGCATCG	+	+	-	-
	CL2705	ACTTGACGACACAAACCAAAGG	CCAAACGCTTTGAGAAAAGGTC	-	-	-	-
	CL2711	TGAGAAACATGGCGTCTTTAGC	GGAGAAGGAGCATCAGATCCAT	+	+	+	RS2CL2711s

Note:'+ ' indicates that primer amplified or SNP detection;'-' means primer not amplified or no SNP detected in this primer.
