# Supplementary material for: QTL Analysis Using SNP Markers Developed by Next-Generation Sequencing for Identification of Candidate Genes Controlling 4-Methylthio-3-Butenyl Glucosinolate Contents in Roots of Radish, Raphanus sativus L
Source: PLoS One. 2013 Jan 7;8(1):e53541. doi: 10.1371/journal.pone.0053541 (PMC3538544; doi:10.1371/journal.pone.0053541)
Supplement: Table S2 — Sequences of primer pairs used for genomic DNA amplification, RT-PCR, and real-time PCR of glucosinolate biosynthesis genes. (PDF) [file pone.0053541.s003.pdf]

Table S2. Sequences of primer pairs used for genomic DNA amplification, RT-PCR, and real-time PCR of glucosinolate biosynthesis genes

Gene name	DNA Fragment amplified primer(5'-3')		RT-PCR Primer sequence (5'-3')		Real-time PCR primer sequence (5'-3')	
	Forward	Reverse	Forward	Reverse	Forward	Reverse
RsBAT5	ATTTTCGGCGTAATTTTGTCG	AACCAGCAACAAAAGCCAAG	-	-	-	-
RsBCAT4	TTAGGATTCGGGTTTTGTCG	ACTCTTCACACCACCGGTTC	CAGGACAGAAGATGGTCGAATTATG	ACAAAACATCAGAGAAACCTTGTGC	AACGCTCTCCGCCTTCAGTC	CCGAGGGACAAGGCATACA
RsIPMDH1	GCAGCTTTACAAACGAACACAC	TGGAGTCCACTGATTTTAGCACT	CAGTTTGACACAATAGTCACCA	ATACACCGAGACTAGCAGATGGA	GGAAAATGCAGATCTCTCAACGA	TGCGGCGCACCTTACTCTAC
RsCYP83A1	AAGATATCATCATCGGGGTGG	TCACTTGCTCACTTTCTC	ACGAGTTCATAACCTACGGGAGAAG	AATCTTCCCCAAAACACTTTGAGTC	-	-
RsCYP79F1	ATGCTCGACTCCGCCTCA	GTCACTTGTGCTTTGATTT	CCTTTGCCAATATAAGCATGTCAAG	AACTCTGTGTCAAAAACCACGGTAA	-	-
RsMAM3	GGTTCCACCGTGGCTGTC	TTAAAATAGTACGGCAATTGTGTTG	CTTCCTTGCTCTCTCTACACCTGAA	TGGTTTTGACTGTTTCGAACTCTTC	CTCAAACCCGTCGTGGAAAG	GTTCTTGTCGGGAAGCTTGTG
RsMAM1	GCTTCTTCACTTCTGACATA	CGTTTGTCATCTTGAGAATA	GTCAGTTTCACCCACTGGATCTTC	AAAACCGACATCCATGACGTCTAC	-	-
Actin	-	-	ATCAGGAAGGACTTGTACGGTAAC	GCTGAGGGAAGCAAGAATGGAACC	ATCAGGAAGGACTTGTACGGTAAC	GCTGAGGGAAGCAAGAATGGAACC
