# Supplementary material for: QTL Analysis Using SNP Markers Developed by Next-Generation Sequencing for Identification of Candidate Genes Controlling 4-Methylthio-3-Butenyl Glucosinolate Contents in Roots of Radish, Raphanus sativus L
Source: PLoS One. 2013 Jan 7;8(1):e53541. doi: 10.1371/journal.pone.0053541 (PMC3538544; doi:10.1371/journal.pone.0053541)
Supplement: Table S3 — Sequences of primer pairs and oligonucleotide probes of SNP markers and the conditions of hybridization and washing. (PDF) [file pone.0053541.s004.pdf]

Table S3. Sequences of primer pairs and oligonucleotide probes of SNP markers and the conditions of hybridization and washing

Marker name	*R. sativus*		Primer sequence (5'-3')			Probe sequence*	Hybridization Condition	
	Linkage Group	Position	Forward	Reverse			TemperATure	SSC
RS2CL1793s	1	0.00	GGATCGTTACAACGCATCTCAA	AGCGTAAACCAGAGCTCTCACA	**TBS**	AAGAGTCAGTGGAAGAA	40	0.5
					**AZ26H**	GAGTCAGCAGTGGAAGA	40	0.5
RS2CL1936s	1	3.70	AACGAGTTCCATGACAAGACCA	TATAGCAACATGACGGACACCA	**TBS**	GGAACTCTTGATGAAGC	40	0.5
					**AZ26H**	GGTACTCTGGATGAAGC	40	0.5
RS2CL1149s	1	10.10	GAGAAGCGGTCGCTGTAAAGAT	GGGACTTTCTGTGTAGGCTTGA	**TBS**	AGACCGGGCTGCAGATC	50	0.1
					**AZ26H**	AGACCGGGATGCAGATC	50	0.1
RS2CL6432s	1	19.90	AAGTCCAAACACATCTCCAAGG	CGACTGACAGGAGGAGTTTGAA	**TBS**	AGTGGGGACCGAACCGT	50	0.1
					**AZ26H**	AGTGGAGATAGAACCGT	50	0.5
RS2CL7263s	1	36.60	TTCGAAAAGACAGTGGTCCTCA	GAGGACCAATAAGCCCAACAAC	**TBS**	GTATAATTCTAGGGACT	45	0.5
					**AZ26H**	GTATAATGTTAGGGATT	40	0.5
RS2CL6218s	1	41.30	CTCCGATAATCCTTCCATCGTC	ATCCTCTTCATTGGTTCGGGTA	**TBS**	ACTTCAGTTGCTCTTGG	40	0.5
					**AZ26H**	ACTTCAGTGGCTCTTGG	40	0.5
RS2CL7011s	1	47.90	AAGCGTAAAGATGCGGTCACTA	GTTTCGCCTTGTCGACTTCATT	**TBS**	GTTCAATCCACTCGGCT	45	0.5
					**AZ26H**	GTTCAATCGACTCGGCT	40	0.5
RS2CL7383s	1	52.40	AGTTCTTGACGAATTGCCTTCC	ACTCAAGCCCATCGACTACGTT	**TBS**	GATTATTTTGAAACGCC	40	0.5
					**AZ26H**	GATTATTCCGAAACGCC	40	0.5
RS2CL2832s	1	59.00	AGCTGGTGAAGGTGAGGTTCTT	GATCCTCCATGTCCAAGCTTTC	**TBS**	GCTGCTATTAAGGCTGC	40	1
					**AZ26H**	GCTGCTATCAAGGCTGC	40	0.5
RS2CL7089s	1	77.20	TTCCAGCCTTCTCTCGAAACTC	TTGTCCATACAAGGCAAGTCGT	**TBS**	CAACTAGGCTTGAAACC	40	0.5
					**AZ26H**	CAAACTAGGTTGAAACC	40	0.5
RS2CL1670s	1	79.80	AACCCATACAAACGGAACTTGG	AAATGGAGGCAGAGAAGACACC	**TBS**	AGTCTCGGTTGGCTAGG	50	0.5
					**AZ26H**	AGTCTCGGCTGGCTAGG	50	0.5
RS2CL1022s	1	89.90	GGCAACCGTTCAAGTTTCTACC	AAGGCAAGTCAACTCCAGCTTC	**TBS**	CAAGTCCTCAGAGGTTT	45	0.5
					**AZ26H**	CAAGTCCTTAGAGGTTT	45	0.5
RS2CL8675s	1	100.80	ACTGAAGGCTTGGTTGAAGAGG	GGCGTTGTGTACAAGCTCAATC	**TBS**	TGGTTGTCTACTTTTTT	40	0.5
					**AZ26H**	TGGTTGTCCACTTTTTT	45	0.5
RS2CL8252s	1	103.70	TCAAGCTGTGCAAAGTGAGAAC	CAAGCGTGAACACATTCCCTAA	**TBS**	TACTCACGGCACCTTTA	45	0.1
					**AZ26H**	TACTCACGTCACCTTTA	45	0.5
RS2CL8726s	1	110.60	GAAAGAGCCGTTGAAGAACCTG	TGTTGCTGTGTGTTTTCCTCCT	**TBS**	CCAATGCATCATCATCC	40	0.5
					**AZ26H**	CCAATGCACCATCATCC	45	0.5
RS2CL1047s	2	9.70	AGCTACCGCCTGATTTTCAAAG	AGACAAACCCCAGACCAGAAAA	**TBS**	TGAAGATGATCAACGAT	45	1
					**AZ26H**	TGAAGATGGTCAACGAT	45	0.5
RS2CL6493s	2	17.10	TGACTCGCCTTTGAAGACTTTG	CTTTCAACAGCACCATCTGAGC	**TBS**	GTGAACATTACAAATAC	40	1
					**AZ26H**	GTGAACATGACAAATAC	40	1
RS2CL1713s	2	27.20	CAGATTGTTTGCTTCCTGCAAC	CATTTGCTCAAGATGCTCCAAC	**TBS**	GAGCTAGCAATGAGACG	40	0.2
					**AZ26H**	GAGCTAGCCATGAGACG	40	0.1
RS2CL1671s	2	26.80	ATGTCCAGAGACGCCGATAGTT	CCTCAGGCAACCAATTCAGATT	**TBS**	AATTCGTTTGAATTTCTC	35	1
					**AZ26H**	AATTCGTTGGAATTTCTC	40	0.5
RS2CL8338s	2	42.00	AGCTGCGTGGACTAGAAGATCA	TGCCATGTTCCTTAATGGTGTC	**TBS**	TCCCGTTCTGATTTCAC	40	0.5
					**AZ26H**	TCCCGTTCGGATTTCAC	45	0.5
RS2CL6248s	2	44.60	GGAGGAGCCAAGACAATAATGG	AGTTGCGTGGAACAAGACAAGA	**TBS**	TGTCCAAAGATAGAAGG	40	0.5
					**AZ26H**	TGTCCAAAAATGGAAGG	40	0.5
RS2CL6306s	2	49.90	TTCCTCGGGATCTCCTCAATAG	TCCTGTTTGACGTGCAGAAATC	**TBS**	GTGATCGTGTTCAAAAA	40	0.5
					**AZ26H**	GTGATCGTCTTCAAAAA	40	0.5
RS2CL1002s	2	51.30	GATGATTTTAAGTGGCCGGAAC	CCACTGCTTACAAGTGCCAAAC	**TBS**	CTCTCCGACATTTTCTC	40	0.5
					**AZ26H**	CTCTCCGATATTTTCTC	40	0.5
RS2CL7437s	2	57.10	GGTATGGTTGTGACCTTTGCAC	CCAGAACGTCTGTCAATCTTGG	**TBS**	GTCTTTATTGTGGAATC	35	0.1
					**AZ26H**	GTCTTTATCGTGGAATC	35	0.1
RS2CL806s	2	68.80	ACATTGTCCTCGAGAACGTCAA	AGAAGCAAGCTCAACGAGAACC	**TBS**	TTTGAAGGGGCTGGAGA	50	0.5
					**AZ26H**	TTTGAAGGTGCTGGAGA	50	0.5
RS2CL8817s	2	70.90	TCTCGCTTACTTCCACAACAGG	AGTGGGGTTTCTCCTCAGATCA	**TBS**	CTTTGGATAATTGCAGC	40	0.5
					**AZ26H**	CTTTGGATGATTGCAGC	40	0.5
RS2CL6006s	2	74.50	AACCATCATCACCCTCTCCATC	CCCGAAGACAAGACTAGCGAAT	**TBS**	AGTGTTTTGAATTTGAGT	35	0.5
					**AZ26H**	GTGTTTTGGATTTGAGT	35	0.5
RS2CL7551s	2	69.60	CTCATGAGCCAGAGTTGTCAAA	GTGTAAACCCACAACGAGCTGA	**TBS**	CTTGGTGCAACCACTGC	50	0.5
					**AZ26H**	CTTGGTGCGACCACTGC	50	0.5
RS2CL1110s	2	87.40	GCTGCGCCTATCTCTCTATTTG	CCGAGACCTGCAGTTTCTTCTT	**TBS**	ATCGGATTGGGCGGAGG	50	0.5
					**AZ26H**	ATCGGATTAGGCGGAGG	50	0.5
RS2CL804s	2	88.40	ACAAGTACGCCTTGCCTCTTCT	GATTCAGCAGCAACCACTTCAC	**TBS**	GGAGACTCTATCGCCTT	50	1
					**AZ26H**	GGAGACTCCATCGCCTT	50	1
RS2CL7457s	2	92.40	TCGTTCCATAAGCGACTCCATA	GTTAATCCGATTGCCTGAGCTT	**TBS**	AGAGATTTTGTAACATA	40	0.5
					**AZ26H**	AGAGATTTGTGTAACAT	40	0.5
RS2CL7234s	2	98.50	CATTTCATCACTCCGCCAAG	TGTTCTTGAAGCCACGTTTAGG	**TBS**	TTGGGAAACAGAGCAGT	45	0.5
					**AZ26H**	TTGGGAAAAAGAGCAGT	45	0.5
RS2CL7712s	2	101.70	ACGAAGCTGCTCTTCGGTTTAG	AGGTAACCGGTTTCGCTAACTG	**TBS**	GATAGAGTACCGTGGAA	45	0.5
					**AZ26H**	GATAGAGTTCCGTGGAA	45	0.5

RS2CL916s	2	112.70	AATTCAACTCTGAGGCTGATGC	GGGAAGTGGAACAAAGCAAAAG	**TBS**	CATGATCTGCTTCACCT	40	1
					**AZ26H**	CATGATCTCCTTCACCT	40	1
RS2CL8027s	2	115.10	ATGGTGCGAAGAAGAGAAAACC	TCGAGAAAGCTGATGAAGATGC	**TBS**	GCTTTTGGACAGAACTT	45	0.1
					**AZ26H**	GCTTTTGGGCAGAACTT	40	0.1
RS2CL7144s	2	120.10	GCTAATTTCTCTCGCGATCCAT	AACGACGATACACAATCCAACG	**TBS**	GCGTCACGGCTCGCGTC	45	0.5
					**AZ26H**	GCGTCACGACTCGCGTC	40	0.5
RS2CL5790s	3	0.00	GTGGAGAGATGAACGGTTTCCT	GACATTGTCTTCATCCCCTGGT	**TBS**	ATTCTGAAGTACTGTCT	35	0.5
					**AZ26H**	ATTCTGAAATACTGTCT	35	0.5
RS2CL842s	3	1.80	AAAGAAGCATCAGTGCCACAAG	AGGAGATTGCTAAAGCCAAGCA	**TBS**	TTGAAATGAAGTCGGCA	50	1
					**AZ26H**	TTGAAATGGAGTCGGCA	50	1
RS2CL7584s	3	3.10	CAGCACAGACAACCGATGAAA	CAAAACTTCCTTCTGCGACACA	**TBS**	TTGACCTCAGGTGGAAA	40	0.1
					**AZ26H**	TTGACCTCTGGTGGAAA	40	0.1
RS2CL8540s	3	10.70	TGGTGGTACATCTCTGGCTACA	GTAAGCAACGCCATGAACTGAG	**TBS**	GCCTCGCTGGCCTTCTT	55	0.5
					**AZ26H**	GCCTCGCTAGCCTTCTT	50	0.5
RS2CL5450s	3	13.80	TAATCCAAGTGGCTCTGCTCTG	CAAACCCAATCTGAATCCTCCT	**TBS**	TCCAAGATAATAAGATT	45	0.5
					**AZ26H**	TCCAAGATGATAAGATT	40	1
RS2CL7828s	3	18.10	TCTTCCAGATGGCAGTAGCTCA	ACTTCACGGAAGGCTTCAGATT	**TBS**	GATTTCTTGTTTTCTGA	45	0.5
					**AZ26H**	GATTTCTTATTTTCTGA	45	1
RS2CL3293s	3	22.10	AAAACTTGTAGTGCCGGTGACA	ACCCACAAACAACAAGTGGACA	**TBS**	CTACTCTCGTCATATGA	45	0.5
					**AZ26H**	CTACTCTCATCATATGA	55	0.5
RS2CL1187s	3	27.70	CATAGCATTGATGGCTCATCCT	CCGAAAAGAGAGCAAAGAGAGG	**TBS**	CTTACGCAGGGTGAAAT	50	1
					**AZ26H**	CTTGCGCATGGTAAAAT	50	1
RS2CL5783s	3	29.40	GAACAACATGGGACGAGATTGA	TATGCGGCTAAGCACTCTTTTG	**TBS**	CTGTGAATTATTTAACT	35	0.5
					**AZ26H**	CTGTGAATATATTTAAC	35	0.5
RsHAO32s	3	34.10	CACATATTGTTGCAACGTCTTGTTA	ACCCATCGTTTGTGCTATGATTC	**TBS**	ATCGGATTGGGCGGAGG	45	0.5
					**AZ26H**	ATCGGATTAGGCGGAGG	40	1
RS2CL6884s	3	34.60	ACGACCAAGTTTCTAGCGAAGC	CCTTCTGTCTCCCACAAAAGAA	**TBS**	GCTGCTATTAAGGCTGC	45	0.5
					**AZ26H**	GCTGCTATCAAGGCTGC	45	0.5
RS2CL8467s	3	44.60	CAAACCGGTTCTGTGAAATCTG	CACGCCTCAGAATAGCAATCAA	**TBS**	GAGGATCTGAGGCTGAA	55	0.5
					**AZ26H**	GAGGATCTCAGGCTGAA	55	0.5
RS2CL9072s	3	49.20	CCTTCACCATCTCATTTCATCG	CGAGCATAGCATTTGAGAGCAT	**TBS**	CTTTCTTTACCTTTCAT	35	0.5
					**AZ26H**	CTTTCTTTTCCTTTCAT	35	0.5
RS2CL3410s	3	58.50	CGATCAACAACGTCTCCTTGTC	CATATCTTAACGCCGTCCATCC	**TBS**	ATAGCTATTGATGTTGC	40	0.5
					**AZ26H**	ATAGCTATCGATGTTGC	35	0.5
RS2CL731s	3	66.90	TTCAAGTCCATGGTGAGAGACC	AAGCACAGTTGGATGAGCTGAA	**TBS**	TAATAAAGAAAAACCAG	40	1
					**AZ26H**	TAATAAAGGAAAACCAG	40	1
RS2CL2598s	3	74.20	GTTTGGAGCTCGAGGTAAGGAA	GTAACAACATTGGGAGCGAGTG	**TBS**	GGACCAAGTCGTAAGAG	45	1
					**AZ26H**	GGACCAAGCCGTAAGAG	50	1
RS2CL8917s	3	80.10	AAGCCACTTGCCTCGTGTTACT	AAGACGCTGTTGGCTTTCATCT	**TBS**	TGTCCAGTCGAGGTCAT	45	1
					**AZ26H**	TGTCCAGTTGAGGTCAT	45	1
RS2CL1743s	3	83.10	CATGCCATTCTTCCATCATAGC	TTCCAGTACAAGCTGGCCATTA	**TBS**	GTGTGGCAATCGTTTAT	50	1
					**AZ26H**	GTGGGGCATTCGTTTAT	50	1
RS2CL6009s	3	92.70	TGTGAGCAAGGTTACCGTCTTG	TTACCATGGCTTCCTCATCTTG	**TBS**	TCCGCTATTGTAAAAGC	40	0.5
					**AZ26H**	TCCGCAATGGTAAAAGC	40	0.5
RS2CL5598s	3	99.90	CCCCAATACCCTATTTCCGTCT	TGGCTTCACAGATTGGGTCATA	**TBS**	GAAATCAATTTTCAAATT	35	0.5
					**AZ26H**	GAAATCAAATTTCAGATT	35	0.5
RS2CL7217s	3	103.60	ACCGGAGAAGATGAAGGTGGTA	CAAACCCTCCTTCAACCTGAAC	**TBS**	AGGTTGTCTACCATTGG	40	0.5
					**AZ26H**	AGGTTGTCGACCATTGG	40	0.5
RS2CL1003s	3	106.90	GTGCAGCCGTATCAGTTTGTTC	GCTCTGCCATTGCTTCTCAGTA	**TBS**	TAGCGAAGATGACTGCG	45	0.5
					**AZ26H**	TAGCGAAGCTGACTGCG	50	0.5
RS2CL983s	3	112.80	GCTCCAGCATTGATCGGTTT	AAGCTCCTTTTGCTGATCATGG	**TBS**	CTGTTTTTTTTTTTTTT	30	0.5
					**AZ26H**	CTGTTTTTATTTTTTTT	30	0.5
RS2CL4891s	3	119.20	TTCGTCCTCTGCTTCGTACTCA	CAGTTGTATCCACCAACGGAGA	**TBS**	GCTCGTCCCCCATCTTC	45	0.5
					**AZ26H**	GCTCGTCCTCCATCTTC	40	1
RS2CL6952s	4	0.00	GAAAGGGAAAGAAAGGAACGTG	AACCAGACTCAGTGTTGTGTCG	**TBS**	TTTCGTTTATACAGAGT	40	0.5
					**AZ26H**	TTTCGTTTTATACAGAG	40	0.5
RS2CL4960s	4	5.80	TGGTCGTCTTGAAGTCCATGAG	CATAAGGGTTTGTTGTCGTTGC	**TBS**	CACCTCCCGAACCGGCC	45	0.5
					**AZ26H**	CACCTCCCTAACCGGCC	40	0.5
RS2CL5071s	4	11.60	GTTTCATCACCAGCAGCAACTC	AGCATCCAATAACCGAGAGAGC	**TBS**	CGTTACTCTGTTCTTGC	40	0.5
					**AZ26H**	CGTTACTCGGTTCTTGC	40	0.5
RS2CL3611s	4	13.70	AATATCGAAAGCACCAGGCTTC	CTTCTTAACCGGAGCAATGACC	**TBS**	CTGAAACGCGGAGCTGA	45	0.5
					**AZ26H**	CTGAAACGGGGAGCTGA	40	0.5
RS2CL2961s	4	17.40	CTCCCAGGGAAGCTAGTGAAAA	TCACCGTGGTTGACAAAAACTC	**TBS**	TATCGACACGATGGTCA	40	1
					**AZ26H**	TATCGACATGATGGTCA	50	0.5
RS2CL3459s	4	22.50	GCCCTTGTAGCTTTTGAAGGAC	ATTCCGCTTGCTAAGGAAGGT	**TBS**	AGCACGTCCAGGCAGAC	40	1
					**AZ26H**	AGCACGTCTAGGCAGAC	35	0.5
RS2CL1390s	4	26.00	ATTACACTCGCCCAGGAACACT	GGTGTGGAAGCTGGTAAAAAGG	**TBS**	TTTACGCTTTGTGAATT	50	0.1
					**AZ26H**	TTTACGCTATGATGTGA	50	0.1
RS2CL1253s	4	31.50	GGACCAGAGGCAAGAATGTTTC	AGCCTCATTCTTGGAGACGAA	**TBS**	TTCTCTAGAAGCATGTT	45	1
					**AZ26H**	TTCTCTAGCAGCATGTT	45	1

RS2CL1620s	4	32.80	GCCAATTACGGACCTTCTCTGT	TACTCTTGGGTTCCATCGCTTT	**TBS**	CATCTACGAATCCTTAT	35	0.5
					**AZ26H**	CATCTACGTATCCTTAT	35	0.5
RS2CL7884s	4	35.10	ATCCCACCGCATATTCTCTCTC	GCTTTAAGGACAGCGCCTTTAG	**TBS**	CTGTTATCCCCCAACTT	45	0.5
					**AZ26H**	CTGTTATCTCCAACTTG	40	0.5
RS2CL831s	4	38.00	AGCAAATGGGAACGAGATGACT	GACATCTTGAAGGAGCAGTGGA	**TBS**	GAATCCTGTCCGTTGGA	40	0.5
					**AZ26H**	GAATCGTGGCCGTTGGA	55	0.5
RS2CL1685s	4	46.90	GAGATGGAGACTGTGCAAATGG	GGGTGGCTCAAAAGGATTAACA	**TBS**	CTAAGATATGCAAATAT	40	1
					**AZ26H**	CTAAGATAAGCAAATAT	40	1
RS2CL986s	4	50.00	GATGATGAGGCAGTTGGTGAAG	TAGCATTGAGAATGTCGGGATG	**TBS**	TGGAACACTTATCCATT	40	0.5
					**AZ26H**	TGGGAAACCTATCCATT	40	0.5
RS2CL6429s	4	57.30	CAAATAGAAAACCACCCACGAC	GGAAACCTGTTGCGTCGACTAT	**TBS**	TGTTGCGAAATTCTTGG	45	0.5
					**AZ26H**	TGTTGCGAGATTCTTGG	45	0.5
RS2CL1695s	5	0.00	TCGATCACGTCATCATCTCCTT	CGTAACCTCTTCAAACCCCAAT	**TBS**	ACTCGATTCGTGTTTAT	40	0.5
					**AZ26H**	ACTCGATTTGTGTTTAT	40	0.5
RS2CL7452s	5	7.40	TGTTCCAACGAGACCAGAAGAG	AAGTTCACGGGACACAACTCAG	**TBS**	TCTTTGATTAGTGGTGA	40	0.5
					**AZ26H**	TCTTTGATCAGTGGAGA	40	0.5
RS2CL1482s	5	13.40	TTGATCTTTGTGGGGTACATGG	GGAATACGCAAACCAAAGTACG	**TBS**	TGACAAACAGGAGGAGC	45	0.5
					**AZ26H**	TGACAAATATGAGGAGC	40	0.5
RS2CL874s	5	18.80	TATCCTAGCCCGCTCATACTTG	TCCAGTCAGTCAGTTCCTCACA	**TBS**	AAGACTAGAGAATAAA	35	0.5
					**AZ26H**	AAAGACTATAGAATAAA	30	0.5
RS2CL5486	5	24.80	CCCCACACCCAAATTTATACCA	AAGACGAGGAGGAAGCAGAAGA	**TBS**	TCCAACTCTTTTAGTCT	40	0.5
					**AZ26H**	TCCAACTCCTTTAGTCT	45	0.5
RS2CL6430s	5	30.30	TCGAGGTACTAACTGGGAAGCA	TATAGCAAAGAAGACGCCGAGA	**TBS**	CTCCACTCTCTCCCCGT	40	0.5
					**AZ26H**	CTCCACTCGCTCCCCGT	40	1
RS2CL1653s	5	34.80	CAACTTGTTGTTCCGTCTTTGC	TTGTCTGGAACGTTCTTGTTGG	**TBS**	CGTACTTTCCGATTCAG	45	0.5
					**AZ26H**	CGTACTTTTCGATTCAG	50	0.5
RS2CL5203s	5	39.10	GAAGGCAGGGGTAAAAGGACTA	TGCAAGCTACTGGGATTTCATC	**TBS**	GAAGGATAAGTATTGTT	45	0.5
					**AZ26H**	GAAGAATAGGTATTGTT	45	1
RS2CL1958s	5	45.00	ACTTCAACGCCGTTTCTTCTTC	CGTTTGTTCTTGACCTTGGAGA	**TBS**	TTGTCATGGCTCGAACC	50	0.5
					**AZ26H**	TTGTCATGTCTCGAACC	45	0.5
RS2CL1347s	5	55.60	TGACAAGCACATGTACCACGAC	GCGCAGAGTAGGTGTTTTGACA	**TBS**	GACGCTGGTCGGAGATT	50	0.5
					**AZ26H**	GACGCTGGCCGGAGATT	50	0.5
RS2CL8045s	5	60.30	TGGAGGGACTAATGAGATTTGG	GAAGAAACGGTGGAGGCATAAG	**TBS**	AACTGGTTAGCTCCGGT	40	1
					**AZ26H**	AACTGGTTGGCTCCGGT	40	1
RS2CL964s	5	63.20	TGTTCTCAGCTTCGTCGTCTGT	TAAAGGGCCCAACATAGAGAGC	**TBS**	GTAATAATAACTCAGGT	30	0.5
					**AZ26H**	TAAATAATCACTCAGGT	30	0.5
RS2CL1092s	5	70.80	ACTGGAATTGGAGGTGAAGAGC	CTTTGATGTCCGCGGATAAACT	**TBS**	GTAGTGTGTCTGATCAT	40	1
					**AZ26H**	GTAGTGTGCCTGATCAT	40	1
RS2CL1172s	5	72.30	AGCAGGGACATGGGTAACAAAT	CTTGACATGAAGCTCAGGGATG	**TBS**	AGTTGATACTAGCAGCT	40	0.5
					**AZ26H**	AGTTGATATTAGCGGCT	40	0.5
RS2CL5504s	5	76.60	GACAAACGGTATGACCCCAAAC	TCGCATCTATTGCTCTTTCAGC	**TBS**	TCACGAGTTCTGTGCCT	45	0.5
					**AZ26H**	TCACGAGTCCTGTGCCT	50	0.5
RS2CL6276s	5	81.20	CATTTACTCGTACGGGATGTGC	CCATGTTTGAAACCAGCTACGA	**TBS**	ACGTTTCTGATTTTCGT	35	0.5
					**AZ26H**	ACGTTTCTTATTTTCGT	35	0.5
RS2CL7928s	5	83.60	CCAAATACCCACCCAAGAAGTG	AAGACCATGTGATGCAATGGAG	**TBS**	GGCGCGCTTCTCAGGGA	50	0.5
					**AZ26H**	GGCGCGCTCCTCAGGGA	50	0.5
RS2CL1098s	5	86.50	AGTATTCCCTTTGCCATGGTTG	TTCAGTTGTCGTATCAGCCACA	**TBS**	TCACCCTTCCCATCCAG	45	1
					**AZ26H**	TCACCCTTACCATCCAG	45	1
RS2CL8711s	5	94.60	CGTGGAAGATGTACCAAGAAGG	AAGGAAGACGTGGAAATGGAAG	**TBS**	CTGGATCGGATCGTTTA	45	0.5
					**AZ26H**	CTGGATCCGATCGTTTA	45	0.5
RS2CL907s	5	96.90	CGCCACTAACATACTTCCGTCT	TCCGTACGAGGTGGTCAACTTA	**TBS**	AAGACCCGTTGACGAAT	45	0.5
					**AZ26H**	AAGAGCCGGTGCCGAAT	50	0.5
RS2CL1549s	5	99.90	TCGAACCATCTTGGTGTGAACT	GCTTAAAGAGGTGTGGCATGTG	**TBS**	AAATGGGGAGACTTCAT	45	0.5
					**AZ26H**	AAATGGGGTGACTTCAT	45	0.5
RS2CL1444s	5	108.10	CTCATCATCACCACTTCCCAAA	GTTAATGTTGGGCAAGACACCA	**TBS**	AATCTCGTTCGAGTAAA	40	1
					**AZ26H**	AATCTCGTCCGAGTAAA	35	1
RS2CL1565s	5	112.90	AACTTCAAAGGCCATGCTCCTA	TCCAGTATGGCCCAATGATACA	**TBS**	GAGACGTTTGAGTGGAG	40	1
					**AZ26H**	GAGACGATGGAGTGGAG	40	1
RS2CL3966s	6	0.00	TGGTTGCTGACAAAGTCATCGT	TCGTTGCTGAGGCTCTTGTAGA	**TBS**	ATATGTGTATAACCACT	40	0.5
					**AZ26H**	ATATGTGTGTAACCACT	40	0.5
RS2CL5433s	6	7.20	CCCATGAGAAAGAATCATCCAG	CGCTCTCTTTCTCTTTGTGTCG	**TBS**	GGTTCCAACTAGTTTGG	45	0.2
					**AZ26H**	GGTTCCAAGTAGTTTGG	40	0.5
RS2CL5207s	6	14.40	CGGTAAGGAGAAGAAGCCTGAA	AAAGCAACTGCGACTTGGAACT	**TBS**	GCCTGGAGATGGATAAG	45	0.5
					**AZ26H**	GCCTGGAGTTGGATAAG	40	0.5
RS2CL5730s	6	14.50	CGATGAAAAGCAACGATGAGAC	CATGGTTTTATACTCGCGGAAC	**TBS**	CCTCCTCAACTCGGAAT	50	1
					**AZ26H**	CCTCCTCATCTCGGAAT	50	1
RS2CL8709s	6	17.20	TATCAATGAGAGCGACACATCG	GCTCCAACGTCATGCAATAGAT	**TBS**	TTCCGTCTGAATTCTCG	40	0.5
					**AZ26H**	TTCGGTCTAAATTCTCG	45	0.5
RS2CL1676s	6	18.70	GTCGTCTCGTCGTCATCAATTC	GAGCACAGTCTTTCCACTTCCA	**TBS**	CTGGTCAAGTTGAATGT	40	0.5
					**AZ26H**	CTGGTGAAACTGAATGT	40	1

RS2CL1245s	6	20.30	AGTTGTCAACCGACCAAACTGA	CTCCATGCGAGTCACAGAGATT	**TBS**	CATTGCTACGAACGCAA	50	1
					**AZ26H**	CACTGCTATGACCGCAA	50	1
RS2CL4292s	6	21.20	TCACCTGTGAGTCAAACCCAAC	GTTTCTGCATGGGAAGCGTAAT	**TBS**	GCTTGAAGGACAAAGGC	40	0.5
					**AZ26H**	GCTTGAAGAACAAAGGC	40	1
RS2CL1388s	6	21.80	CCGTTTCTCTCCTTCACTTTCC	TTCCCCTAGTTCGACATCTCCT	**TBS**	ATTCAGTCTAAGTGGGA	40	0.5
					**AZ26H**	ATTCAGTCCAAGTGGGA	45	0.5
RS2CL9032s	6	24.00	AGATTGGCAACTGATCGTCGT	GGGCTCAAGCAGAAGTCAAAT	**TBS**	TCCGGTTTATCTACCGC	45	0.5
					**AZ26H**	TCCGGTTTGTCTACCGC	50	0.5
RS2CL1450s	6	28.70	CACAATCTTCTCACCGTTCGTC	CTCAATGTAGCGGGTGAAACAG	**TBS**	CGTGGTCACCAGATCAT	40	0.5
					**AZ26H**	CGTGGTCAACAGATCAT	40	0.5
RS2CL4585s	6	32.30	GACATTGAACCCAATGGTGCTA	GGTTCGAAGCCTATCTCACACA	**TBS**	ACTTCTGAGGGCAATGT	50	0.5
					**AZ26H**	ACTTCAGATGGGAATGT	50	0.5
RS2CL5958s	6	34.30	CATAACAAGCCAGTGCAAAGGT	ATTGCAGACATCTGGCTTTCCT	**TBS**	GCTGCAGACGTAAACGC	40	0.5
					**AZ26H**	GCTGCAGATGTAAACGC	45	0.1
RS2CL1196s	6	36.90	ACTGCCTTATCGCTTCCCTCTT	CGTTGCTCGAGGAGAGAATCTT	**TBS**	TGTGTTTTACATAAAGA	30	0.5
					**AZ26H**	GTGTTTTTACATAAAGA	35	0.5
RS2CL1096s	6	41.00	GTAACTTTTGGTTTCGGGGTTG	TACCGTCACCATCCATCTCTTG	**TBS**	TATTCAGAATAATTTTT	30	0.5
					**AZ26H**	TATTCAGATAATTTTTT	30	0.5
RS2CL5384s	6	58.80	CTACTGCGACGAAAACAACAGG	ACTAAGACCCAACCCAAGAGCA	**TBS**	CAAGACAAGAAGATCAT	40	1
					**AZ26H**	CAAGACAAAAAGATCAT	40	1
RS2CL5949s	6	61.30	TGGAGAAACCGAAGAAGAGGAC	AGGTGAAATGCGAAGGTGAATC	**TBS**	CCGTTGATCAAGCTCTC	45	0.1
					**AZ26H**	CCGTTGATTAAGCTCTC	45	0.1
RS2CL5298s	7	0.00	TACTCGACAAATCCGGGAGAAT	CCTTTGGCCATTAGGTTGTGTT	**TBS**	TACTTCCTGTCTCAAGA	45	0.5
					**AZ26H**	TGCTTCCTTTCTCAAGA	45	0.5
RS2CL1983s	7	11.50	GATGTTTTGGACGGTTCAAGTG	TCGGAAGCTTTAGCTCTCCATC	**TBS**	TACGTGACCTTGGACCG	40	1
					**AZ26H**	TACGTGACGTTGGACCG	45	0.5
RS2CL1396s	7	20.30	CTCCTGGAGTATTTCCAAGCAA	GATGGATGGATTGAAGGGATTC	**TBS**	AACAACAATGCCTTGAT	40	0.5
					**AZ26H**	AATAACAACGCCTTGAT	35	0.5
RS2CL6263s	7	26.80	GTTCTGTTGCGTCGTTTCCA	TGGATCAAAGATGAAGGACTCG	**TBS**	AGAAAGGCTGTGACTTT	45	0.5
					**AZ26H**	AGAAAGGCAGTGACTTT	45	0.5
RS2CL3356s	7	33.30	TGACGTGTGGCAATACCTTTGT	CGATATTAAGAACCCCGTCAGG	**TBS**	ATGTATGCCGAGGAGCA	50	0.5
					**AZ26H**	ATGTATGCTGAGGAGCA	50	0.5
RS2CL5288s	7	38.70	GTTCAACTCCAGGGGAGATGTT	CTCCATAATCCCACCATCAAAG	**TBS**	GTGGACCGTTGTCTTCT	45	0.5
					**AZ26H**	GTGGACCGATGTCTTCT	50	1
RS2CL1403s	7	44.20	AGGAGGGAGCTTCTGCTTTCTT	CACATAGAACTCGCACAAATGG	**TBS**	CTTGCACATTATTCTTG	40	0.5
					**AZ26H**	CTTGCACACTATTCTTG	40	1
RS2CL1299s	7	51.10	ACACCACACCAAACAACCAAAC	ATGCGAGCGTAAACAACGTC	**TBS**	GACGATGATGTGGTTGT	50	0.5
					**AZ26H**	GACGATGACGTGGTCGT	55	0.5
RS2CL3940s	7	56.00	AGATTATGAGTGGGGACGCAAG	GAAGAAACCACCGTTGCTAACC	**TBS**	AGAGATTTCGTCGCTCT	40	0.5
					**AZ26H**	AGAGATTTTGTCGTTCT	40	1
RS2CL1923s	7	59.80	CAACCAGACTAAGAAGCCCAAA	GAAAAGATGCCAGCAAGGATG	**TBS**	TCCTCTCTTACAGCTTC	45	0.5
					**AZ26H**	TCCTCTCTCACAGCTTC	45	0.5
RS2CL6221s	7	64.80	ACCCTAATTTCATCCCCCATTC	CACCCACTGACCCAAAACATTA	**TBS**	ATAACACAAGTGAAGTT	45	0.5
					**AZ26H**	ATAACACAGGTGAAGTT	40	1
RS2CL1692s	7	69.80	ACGCAGTCGGTTTGCTATCTTT	ACAGTAGCATCGAGGCTTTGAC	**TBS**	CTGAACCAGAACAGAGT	35	0.5
					**AZ26H**	CTGAACCAAAACAGAGT	35	0.5
RS2CL8607s	7	89.60	GCTTCCGCAGATCCTTGAAT	TCCAGTGTTTGCAATGTCCA	**TBS**	TCTGATTTATCTCCACC	40	1
					**AZ26H**	TCTGATTTGTCTCCACC	40	1
RS2CL1123s	7	99.10	GCATGGTGTGCATCTAAAGGAA	TGTATGGTAACCCTCTGCAACA	**TBS**	AAACTTGGAGCTGCTTC	50	0.5
					**AZ26H**	AAACTTGGTGCTGCTTC	50	0.5
RS2CL6356s	8	0.00	TCAGTACGGAAGACCGCTGTTA	TGCTAGTCGGGAAAGTGTCAAA	**TBS**	ATGTTCAGTTTAAGGAG	50	0.1
					**AZ26H**	ATGTTCAGCCTAAGGAG	45	0.5
RS2CL7272s	8	5.40	ATCATAGGACATCCCTGGACCT	ACGCAAAGCCTAGCATAAAAGC	**TBS**	TGAAGTGTCACATGCTG	45	0.5
					**AZ26H**	TGAAGTGTAACATGCTG	40	0.5
RS2CL8267s	8	11.50	AGCTACAAGACGCGTGATACGA	AAGCTCCGGGAAACTGTATTGA	**TBS**	GATTGATAGAAAAAAAA	35	0.5
					**AZ26H**	GATTGATAAAAAAAAAG	35	1
RS2CL7786s	8	17.90	GGCTTTCCTGAAGTGGTACAGA	ACCTAGAGGTGTTTTCCCCTGA	**TBS**	AAGACACATACAAAGGT	40	0.5
					**AZ26H**	CACACACAGACAAAGGT	40	0.1
RS2CL1854s	8	20.90	CAACATCTCCTCCTGAAGCTGA	TTCTCTGAGCTGATCCAGTTGC	**TBS**	ACCGAGCTGGCTACCAC	50	0.5
					**AZ26H**	ACCGAGCTCGCTACCAC	40	0.5
RS2CL706s	8	23.00	CAGCTCCACAAAACTCAGCAAG	TTCCAGAGTTGTGCCACTTCAG	**TBS**	GAAGATGAATATGACAA	35	0.5
					**AZ26H**	GAAGATGAGTATGACAA	35	0.5
RS2CL6905s	8	27.30	TCGGAGGATTGTGGTTGTTATG	GAAAACGGCGAAGGATACTGAT	**TBS**	GGTGACAATGTGGGATT	45	0.5
					**AZ26H**	GGTGACAACGTGGGATT	45	0.5
RS2CL2122s	8	28.80	AAATCCTCTGATCCCATCAACG	GCTTACAACACCTCGTTTCACG	**TBS**	AAAGAG:TCAGTCTCAAG	50	0.1
					**AZ26H**	AAA:AGCTCTGTCTCAAG	45	0.5
RS2CL6151s	8	35.70	GGACAAGATCGGGAAAAATGG	CTTCTCGACCGGAGTCTGATTT	**TBS**	GCTCACATCATGAACGA	50	1
					**AZ26H**	GCTCACATTATGAACGA	50	1
RS2CL1672s	8	36.80	GGGTCACGTGAAAGTTTGGATT	CACGGATCTGCTCAACTTTCTT	**TBS**	CCACATTTTGTCTAGGT	40	0.1
					**AZ26H**	CCACATTTGTCTAGGTA	40	0.5

RS2CL895s	8	38.40	GGGATTGAAGGAAGCAGAGAGA	TTGAGCAGATCATCGCCTAGAG	**TBS**	AAGTGTTGGCTCAGATT	45	0.5
					**AZ26H**	AAGTGTTGACTCAGATT	45	0.5
RS2CL6126s	8	39.50	GGTTTGTGTTTGAGACCGAGAA	AGACTGTTCAGACACCGAGCAA	**TBS**	AGATGGTTGCACGAGCT	50	0.5
					**AZ26H**	AGATGGTTACACGAGCT	50	0.5
RS2CL5805s	8	46.70	CGTGGTTAGCACGGAAAACATA	CGGTGCAAAGTCATCCTAGAAG	**TBS**	CGATTTGCATCTCTCTC	55	0.5
					**AZ26H**	CGATTTGCTTCCCTCTC	50	0.1
RS2CL8082s	8	48.00	CTTCTTAAACCACCGAGCCAAC	GATGTCAAATTGCTTGGGGAAC	**TBS**	TTTCCTTGTTGAATTTT	40	0.5
					**AZ26H**	TTTCCTTGATGAATTTT	40	0.5
RS2CL1283s	8	50.90	GATTCCAAAGCCAACTGCTCTT	GTCCACACCTCGGCAACTTTAT	**TBS**	ATCCGTGAATGTGCTTA	50	1
					**AZ26H**	ATACGTGACTGTGCTTA	50	1
RS2CL7647s	8	53.40	GGGATCTTAGCGTTTCACAAAG	GCTTGCGGGTATTTGCTCAT	**TBS**	CAAGAACTGTCCGTTCA	40	0.5
					**AZ26H**	CAAGAACTCTCCGTTCA	50	1
RS2CL7978s	8	56.10	TCCGATTCTCCTGTTCCTTCTC	CCGAGAAGCTTGACAATGTGAG	**TBS**	GCCATCTCATCATCACA	40	0.5
					**AZ26H**	GCCATCTCGTCATCACA	45	0.5
RS2CL685s	8	64.80	CTTTGTGCTCACAGTGGGTGAT	GCGTCACTCTCATGTTGTCCTT	**TBS**	CCAGGTTCTAGCGAGTC	45	0.5
					**AZ26H**	CCAGGTTCCAACGATCC	45	0.5
RS2CL8033s	9	0.00	CTGCTGATGCCTACATCTTTGC	AACCAAGAATCACCGGTTTCAG	**TBS**	CGGTTAAGACAAAATGT	35	0.5
					**AZ26H**	CGGTTAAGTCAAAATGT	35	0.5
RS2CL1610s	9	7.40	TACGAGAGACACGACGTGATGA	CAAACGAATACACATGCCCAGT	**TBS**	CTCGAGTCTGGGACAGG	35	0.5
					**AZ26H**	CTCGAGTCAGGGACGGG	40	0.5
RS2CL7086s	9	12.70	AGTGTTTGGGGAAGAGAAGGTG	TCTGCTCGACGTTCTTTCTCAG	**TBS**	TATATGTGGAGCCATAA	40	0.5
					**AZ26H**	TATATGTGCAGCCATAA	40	0.5
RS2CL8124s	9	17.50	ATTGGTCAGCTAGGTTCGCTTC	GTTCAATCGTGTGATGCGTTG	**TBS**	GAGAGATTCCGAATAAT	35	0.5
					**AZ26H**	GAGAGATTACGAATAAT	35	0.5
RS2CL1576s	9	21.60	GAGTTCAATGCATCGAGCAGAT	GTCCTCCTGTTTTCTCCTCCAA	**TBS**	ATCCAGTGCAGTACTAC	40	0.5
					**AZ26H**	ATCCAGTGTAGTACTAC	40	0.5
RS2CL3030s	9	25.40	AGCTTGCTAACCTGGTGGTTGT	ATCTGGTCCCAGTGAGATGGAT	**TBS**	GCCTTTGGTCTGACTGT	40	0.5
					**AZ26H**	GCCTTTGGGTTGACTGT	35	0.5
RS2CL1984s	9	29.80	CGAAGATCACAGAGGTTGATCG	TGAGTCCGCTTCTTCTTCAATG	**TBS**	GGCAAAACATTGAACAC	40	0.5
					**AZ26H**	GGCAAAACGCTGAACAC	40	0.5
RS2CL6027s	9	36.10	TACCTTCCATCGGTGAGAACAG	CAGTCAGAAGAGGAACCCGAAT	**TBS**	TGTGTTTAAGCTCAATT	40	0.5
					**AZ26H**	TGTGTTTAGGCTCAATT	40	0.5
RS2CL5291s	9	46.20	AAAAACCGATCCCGACACACT	GGTTTCTCATCTGTTCCTGCAA	**TBS**	CCTAAACCCCAAAGGTT	50	0.5
					**AZ26H**	CCTAAACCTCAAAGGTT	50	0.5
RS2CL7455s	9	49.70	TCTGCAAGTAGGCGAGAACAGA	GTCATGTCGAAACCACAAGCTC	**TBS**	GTGTCGGTTTACCGTTA	50	0.5
					**AZ26H**	GTGTCGGTGTACCGTTA	50	0.5
RS2CL2344s	9	57.80	AACTGGTACGCGTTTGACAATG	AACAGGGCATGAGGATTTGTCT	**TBS**	TTTCTCTGGTACCTACA	40	0.5
					**AZ26H**	TTTCTCTGATACCTACA	50	0.5
RS2CL1154s	9	60.10	CATGAGACTTTCCGTGACCAAC	TCTCTCCAGACCAAACTGACCA	**TBS**	TCGTTATTCTGAAGTTT	45	0.5
					**AZ26H**	TCGTTATTTTGAAGTTT	40	0.5
RS2CL1297s	9	64.50	ATTGGCAGCTCAAGTCACAGAG	GAATCTGCAGGTGGTTTCTTCA	**TBS**	ATATTGTGCTGTTTCTC	40	0.5
					**AZ26H**	ATATTGTGATGTTTCTT	40	1
RS2CL4290s	9	68.20	CAGAGTCGCTAACCCTTTGACA	ACCCGAGAAAACTGCGTACTTC	**TBS**	TATGCCCTTCACCAGAG	50	0.5
					**AZ26H**	TATGCCATCACCAGAGA	50	0.5
RS2CL6364s	9	78.80	AAATACACTCAAGGGTGCAAGG	AGAGCTGCTCACTGTGGCTAAA	**TBS**	TCACCATCCTGGTTCGT	40	0.5
					**AZ26H**	TCACCATCATGGTTCGT	50	0.5
RS2CL1508s	9	82.40	CAACTCTATCTATGGCCGCTGA	CGCTCAAGAAACTCGACAAAGA	**TBS**	CTTCTTCCGCTTCTTCT	45	0.5
					**AZ26H**	CTTCTTCCTCTTCTTCT	45	0.5
RS2CL1894s	9	87.00	CAAGCTTCCATTGTCTGAAGGA	AGCTGAAGTTGGAGTGGTTTCC	**TBS**	GTAAAGGTCCGGACAGG	40	0.5
					**AZ26H**	GTAAAGGTTCGGAGAGG	40	0.5
RS2CL1633s	9	95.10	GCCGCAAAGCTTATCATCAA	CGATACTCAGCAAGTGCCCATA	**TBS**	AGAAACACTTTCCCATT	35	0.5
					**AZ26H**	AGAAACACCTTCCCATT	35	0.1

* The oligonucleotide probes were designed as bridge probes (Shiokai et al. 2010b). Sequences excluding bridge sequence are shown. A sequence, TATATTTACATTCGCAATTAAGAGGCTTCGT designated as SCR-27, and a sequence, TATATTCCCTCCGTCAGCGGATC designated as SCR-52, were added to allele-specific sequences of 'TBS' and 'AZ26H', respectively.
