# Supplementary material for: QTL Analysis Using SNP Markers Developed by Next-Generation Sequencing for Identification of Candidate Genes Controlling 4-Methylthio-3-Butenyl Glucosinolate Contents in Roots of Radish, Raphanus sativus L
Source: PLoS One. 2013 Jan 7;8(1):e53541. doi: 10.1371/journal.pone.0053541 (PMC3538544; doi:10.1371/journal.pone.0053541)
Supplement: Table S4 — Dot-blot-SNP markers previously mapped with newly identified SNPs. (PDF) [file pone.0053541.s005.pdf]

Table S4. Dot-blot-SNP markers previously mapped with newly identified SNPs

Marker name	*R. sativus*		Primer sequence (5'-3')		Probe sequence*		Hybridization Condition	
	Linkage Group	Position	Forward	Reverse			TemperATure	SSC
RS2CL6241s	1	17.30	CGAGTACGCAGCGAACATAAAG	CCATTTCTCCGATCAGGCTAAC	**TBS**	GAAGACAACCAAGGAGA	50	1
					**AZ26H**	GAAGACAATCAAGGAGA	50	1
RS2CL6824s	1	67.20	ATAGAAGCGATGATGCTGGTCA	GCTTGATCTGCCATCGTAATGA	**TBS**	AGGCCCTTAAAACCTGA	40	1
					**AZ26H**	AGGCCCTTGAAACCTGA	40	1
RS2CL6670s	2	0.00	GGATCCCGAAACACACAAGACT	CCTGATAGCCAAGGTGAAAAGG	**TBS**	TTTGTTACCAAGACGTT	40	1
					**AZ26H**	TTTGTCACTAAGATATT	40	1
RS2CL6094s	2	20.80	CCGTTCACGTTCTGATGAAATC	TAATGGCCCCCTTAGATTGTTG	**TBS**	AACACTACATCTCCTTC	40	0.5
					**AZ26H**	AACACTACCTCTCCTTC	40	0.5
RS2CL6594s	2	25.10	AACCTTGCAACGATCCGAAA	AGCTCTTCCAGCCTGATATCCT	**TBS**	CTAGTGCCACCGCCGCA	45	0.5
					**AZ26H**	CTAGTGCCGCCGCAGCA	45	0.5
RS2CL7602s	2	62.30	GGTGATTCAACGAAGAGGAAGA	TATAGGTTCGACGTCTCCGAGT	**TBS**	CCATCTGCGGCCTCTGG	50	0.5
					**AZ26H**	CCATCTGCTGCCTCTGG	50	0.5
RS2CL4583s	2	120.10	GCTAATTTCTCTCGCGATCCAT	AACGACGATACACAATCCAACG	**TBS**	ATTGTGAATCCGCTATG	50	1
					**AZ26H**	ATTGTGAAGCTGCTATG	50	1
RS2CL5808s	3	65.30	ACGCGTAAACGTGAGCAGAA	CCCAGAAGTTGTAACCGTCGAT	**TBS**	GTTCAATCCACTCGGCT	45	0.5
					**AZ26H**	GTTCAATCGACTCGGCT	45	0.5
RS2CL5873s	3	127.80	GCTTCCCTTTCTCGTTTTCTCA	CAATGTTCTTCAATCCCAGCAC	**TBS**	CGCCTTCTTCTCACAAG	45	0.5
					**AZ26H**	CGCCTTGTCACAAGAAC	45	0.5
RS2CL8169s	4	41.50	ATTGTGGAGGTGATGATCCAGA	CAAGGCCAAGCCATTCTTTAAC	**TBS**	TCAAGAGTCTGAACAAC	45	0.5
					**AZ26H**	TCAAGAGTTCGAACAAC	40	0.5
RS2CL6213s	4	64.30	GGGCTTGTGGACAAGGAATAGA	TACCCCCAAGAAGCTAACTGTG	**TBS**	AGTTTGATTTTGGAAAC	35	0.5
					**AZ26H**	AGTTTGATGTTGGAAAC	35	0.5
RS2CL3438s	5	0.00	TCGATCACGTCATCATCTCCTT	CGTAACCTCTTCAAACCCCAAT	**TBS**	AACACCATTTCAACCTC	40	0.5
					**AZ26H**	AACACCATCTCAACCTC	50	0.5
RS2CL5908s	5	3.90	TCTACGATCAACCAACCCTCCT	GAGGTTCTCGATGATGACGTTG	**TBS**	TTGATCTCCGGCGTCCT	50	0.5
					**AZ26H**	TTGATCTCTGGCGTCCT	50	0.5
RS2CL8415s	5	10.10	CGGATTTAGAATACAGCGATGC	TGAGGAAGCTGTGTCATCAAGG	**TBS**	CAAGACCACATTATATA	40	1
					**AZ26H**	CAAGACCATATCATATA	40	1
RS2CL8092s	5	90.70	AATCATCCCGAGGCATCTTCT	GCGAAAAGTTGTGGATCTGAAG	**TBS**	GTTTCATCGTCCTTCTT	40	0.5
					**AZ26H**	GTTTCATCATCCTTCTT	40	0.5
RS2CL6155s	6	12.00	CGACGAGAGGAGACAACAAAAA	AGGCCTGAAGATGATACCGAAC	**TBS**	CAGATACTGCACGATTG	50	0.5
					**AZ26H**	CAGATACTACACGATTG	45	0.5
RS2CL5594s	7	79.10	GTATGAAGCACCAGGAGCTGTT	AAACTCTAAAGCCCAAGGAACC	**TBS**	GCTGATGCCGTGAGGCA	50	0.1
					**AZ26H**	GCTGATGCGGTGAGGCA	50	0.1
RS2CL5804s	8	14.60	TGGAGGAGTTCATAAGCAGCAA	CTGGAACAAAGACTGAGGCAAA	**TBS**	ATCGGCGTATTTCCTCT	50	0.5
					**AZ26H**	ATCGGCGTGTTTCCTCT	50	0.5
RS2CL6357s	8	26.10	GCGCCTCTCTTCATCTTCTTTC	CAGTGCCATCAGCGAGTCTAAA	**TBS**	TAGATGCGCAGTAGATT	50	0.5
					**AZ26H**	TAGCTGCGTCGTCGATT	50	0.5
RS2CL7214s	8	30.50	AGCTGATCCAGTTCGAGTTGCT	ATTGTGCGTGCAAAGGAAAC	**TBS**	CTCTACTATGCCTGTGT	40	0.5
					**AZ26H**	CTCTACTACGCGTGTGT	35	0.5
RS2CL627s	8	32.00	AAGAAGAAAGGAGGGTGCCTCT	AAAGTAACGGCGCTCATTGG	**TBS**	CTCACCATAACCAGAGC	40	0.5
					**AZ26H**	CTCACCATGACCAGAGC	50	0.5
RS2CL6233s	8	42.50	CCAGATTTTGCCTAATCTCAGC	CTTTGCATTTAACGGGGACAC	**TBS**	ATGACTATGCCGCGCTG	45	0.5
					**AZ26H**	ATGACTATTCCGCGCTG	45	0.5
RS2CL5940s	8	43.70	GAAGAGCTACAAAAGGGGCAGA	ATCGTGCGTCTGCTAGAAACAA	**TBS**	CACTTATACCACTCTCA	40	0.5
					**AZ26H**	CACTTATAGCACTCTCA	40	0.5
RS2CL7231s	8	44.60	TCCCATCCAAAATCTCGATCTC	GATGTCGGAGACATAAGGTCCA	**TBS**	CTTCTCATACGGCTCCA	55	0.1
					**AZ26H**	CTTCTCATCCGGCTCCA	45	0.1
RS2CL5546s	8	46.30	AGTTGTGACATCATCCGCAAAG	GCGGTTTATCAGCAGAAACAGA	**TBS**	ACGTAATACGCAATGTA	50	1
					**AZ26H**	ACGTAATAAGCAATGTA	50	1

* The oligonucleotide probes were designed as bridge probes (Shiokai et al. 2010b). Sequences excluding bridge sequence are shown. A sequence, TATATTTACATTCGCAATTAAGAGGCTTCGT designated as SCR-27, and a sequence, TATATTCCCTCCGTCAGCGGATC designated as SCR-52, were added to allele-specific sequences of 'TBS' and 'AZ26H', respectively.
