# Supplementary material for: QTL Analysis Using SNP Markers Developed by Next-Generation Sequencing for Identification of Candidate Genes Controlling 4-Methylthio-3-Butenyl Glucosinolate Contents in Roots of Radish, Raphanus sativus L
Source: PLoS One. 2013 Jan 7;8(1):e53541. doi: 10.1371/journal.pone.0053541 (PMC3538544; doi:10.1371/journal.pone.0053541)
Supplement: Table S5 — Comparison of 4MTB-GSL contents between different genotypes of SNP markers in GSL-QTL-1 and GSL-QTL-3. (PDF) [file pone.0053541.s006.pdf]

Table S5. Comparison of 4MTB-GSL contents between different genotypes of SNP markers in GSL-QTL-1 and GSL-QTL-3

Group number	Marker genotype		2010 experiment		2011 experiment	
	RS2CL6432s	RS2CL4585s	Number of plants	4MTB-GSL content(μmol/g DW)	Number of plants	4MTB-GSL content(μmol/g DW)
1	AB	BB	11	73.6±7.45 a	25	58.8±3.39 a
2	AB	AB	47	61.7±2.97 ab	42	53.7±2.08 a
3	AB	AA	17	60.2±5.14 abc	20	41.9±3.49 a
4	AA	AB	17	56.9±2.89 abcd	22	46.7±2.23 a
5	AA	BB	8	54.3±3.14 abcd	13	41.5±4.39 a
6	AA	AA	4	53.5±13.10 abcd	10	43.8±3.26 a
7	BB	AA	3	39.4±7.01 abcd	7	17.2±2.33 b
8	BB	BB	6	38.3±7.78 bd	8	41.4± 8.46 a
9	BB	AB	13	35.1±5.87 d	11	17.5±3.70 b

Note:Values followed with same letter within each experiment are not significantly different at the 5% level were determined by Tukey's multiple comparison test.
